# Resistance Training Increases White Matter Density in Frail Elderly Women

**DOI:** 10.3390/jcm12072684

**Published:** 2023-04-04

**Authors:** Marco Bucci, Patricia Iozzo, Harri Merisaari, Ville Huovinen, Heta Lipponen, Katri Räikkönen, Riitta Parkkola, Minna Salonen, Samuel Sandboge, Johan Gunnar Eriksson, Lauri Nummenmaa, Pirjo Nuutila

**Affiliations:** 1 Turku PET Centre, University of Turku, 20520 Turku, Finland; 2 Theme Inflammation and Aging, Karolinska University Hospital, 141 86 Huddinge, Sweden; 3 Department of Neurobiology, Care Sciences and Society, Division of Clinical Geriatrics, Center for Alzheimer Research, Karolinska University, 171 77 Stockholm, Sweden; 4 Institute of Clinical Physiology, National Research Council, 56124 Pisa, Italy; 5 Department of Radiology, Turku University Hospital, University of Turku, 20014 Turku, Finland; 6 Turku Brain and Mind Center, University of Turku, 20014 Turku, Finland; 7 Department of Psychology and Logopedics, University of Helsinki, 00014 Helsinki, Finland; 8 Folkhälsan Research Centre, 00250 Helsinki, Finland; 9 Finnish Institute for Health and Welfare, 00271 Helsinki, Finland; 10 Psychology/Welfare Sciences, Faculty of Social Sciences, University of Tampere, 33014 Tampere, Finland; 11 Department of General Practice and Primary Health Care, University of Helsinki, Helsinki University Hospital, 00290 Helsinki, Finland; 12 Singapore Institute for Clinical Sciences, Agency for Science, Technology, and Research, Singapore 138632, Singapore; 13 Department of Obstetrics & Gynaecology and Human Potential Translational Research Programme, Yong Loo Lin School of Medicine, National University of Singapore, Singapore 119228, Singapore; 14 Department of Endocrinology, Turku University Hospital, 20520 Turku, Finland

**Keywords:** resistance training, frailty, magnetic resonance imaging, VBM, positron-emission tomography, glucose clamp technique, cognitive dysfunction/diagnosis, aged, female, diffusion tensor imaging

## Abstract

We aimed to investigate the effects of maternal obesity on brain structure and metabolism in frail women, and their reversibility in response to exercise. We recruited 37 frail elderly women (20 offspring of lean/normal-weight mothers (OLM) and 17 offspring of obese/overweight mothers (OOM)) and nine non-frail controls to undergo magnetic resonance and diffusion tensor imaging (DTI), positron emission tomography with Fluorine-18-fluorodeoxyglucose (PET), and cognitive function tests (CERAD). Frail women were studied before and after a 4-month resistance training, and controls were studied once. White matter (WM) density (voxel-based morphometry) was higher in OLM than in OOM subjects. Exercise increased WM density in both OLM and OOM in the cerebellum in superior parietal regions in OLM and in cuneal and precuneal regions in OOM. OLM gained more WM density than OOM in response to intervention. No significant results were found from the Freesurfer analysis, nor from PET or DTI images. Exercise has an impact on brain morphology and cognition in elderly frail women.

## 1. Introduction

Frailty is a clinical syndrome that develops as a consequence of age-related decline in many physiological systems including the brain, which collectively results in vulnerability to health status changes triggered by minor stressors [1]. Frailty is also associated with cognitive impairment and/or faster cognitive decline and dementia [1]. The established links between frailty, cognitive decline, and functional impairments prompt the necessity to investigate brain structure in frailty [2]. Voxel based morphometry (VBM) can provide measures of white and gray matter density [3,4,5,6]. Ageing of the brain is not accompanied by significant neuronal loss [7]; whereas, WM volume rapidly declines after 60–70 years of age across multiple regions [8,9].

Maternal obesity during pregnancy affects the development of the growing fetus [10,11,12] and increases offspring risk of morbidities in later life [13]. Offspring of obese mothers have an increased risk to develop neurodevelopmental complications, such as cognitive decline and symptoms of attention deficit hyperactivity disorder in childhood, eating disorders in adolescence, and psychotic disorders in adulthood [14,15]. Maternal obesity was shown to increase the risk of intellectual disability or cognitive deficits by up to 3.6 fold in the offspring [16,17,18,19]. Nonetheless, the effects of maternal adiposity on the offspring’s brain structure remain poorly characterized.

Neurobiological mechanisms suggest that the age-related decline in white matter (WM) affecting the structural integrity of myelin contributes to alterations in cognitive abilities [20]. Diffusion tensor imaging (DTI), in particular fractional anisotropy maps, can provide measures of structural integrity of white matter [3]. One DTI study in 39-week-old infants found that WM integrity was reduced in several brain regions in offspring of obese mothers (OOM) compared to offspring of lean mothers (OLM) [21]. WM integrity abnormalities and cognitive impairments have also been associated with frailty in elderly subjects [22]. To our knowledge, white matter integrity has not investigated in frail offspring of obese and lean mothers.

Brain glucose uptake is intimately associated with brain health and neurodegenerative disease [23,24]. Furthermore, offspring of obese mothers have altered skeletal muscle glucose metabolism in adulthood [25] and, except for a methodological study [26], we are not aware of any studies addressing brain glucose metabolism in vivo in frail OLM or frail OOM subjects. Brain glucose uptake measured via [^18^F]FDG PET in the brain can also be used to measure acute and chronic effects of exercise [27].

Physical activity is beneficial in frail subjects [28,29,30,31], and can have beneficial effects in multiple systems including brain and cognition [32,33,34]. Both aerobic and non-aerobic interventions increased functional connectivity in the brain of older adults after one year, and non-aerobic training was already effective within 6 months [35]. Mild-intensity exercise could prevent prefrontal volume reduction due to ageing and decelerate cognitive decline [36]. However, to our knowledge, no study has investigated the possible volume changes in the brain following a regime of resistance training in frail OLM subjects, as compared to frail OOM subjects.

Therefore, this study was designed to examine the effects of maternal obesity (OLM compared to OOM) on brain structure and metabolism in frail women, and the possible reversible effects of exercise on the alterations evidenced by group comparisons. We hypothesized that frail elderly women have decreased GM and WM density, volume, and metabolism compared to controls not statistically different for age/gender/body mass index (BMI). By conducting a resistance exercise training intervention, we sought to reduce alterations in brain structure and metabolism of frail OOM and OLM.

## 2. Materials and Methods

### 2.1. Study Subjects

Study subjects were enrolled from the Helsinki Birth Cohort Study II (HBSC) [13], the largest and one of the best-characterized longitudinal cohorts spanning the entire lifespan. Details about inclusion and exclusion criteria have been described in our previous study [25]. Briefly, 37 elderly women were selected as frail (F) participants and 9 non-frail elderly women as controls (CTR); BMI and age were not statistically different between the groups. Grip strength measurements were used to stratify frail and non-frail women according to the median of the HBSC clinical sub-cohort obtained in a previous clinical investigation [37,38,39]. To investigate the effect of maternal obesity, the F group was additionally divided into two groups according to maternal BMI at pregnancy: offspring of lean mothers (OLM) with maternal BMI ≤ 26.3 kg/m^2^ (lower half of the HBSC population) and offspring of obese mothers (OOM) with maternal BMI ≥ 28.1 kg/m^2^ (highest quartile of the HBSC population). CTR group individuals were non-frail and born-to-lean mothers (BMI ≤ 26.3 kg/m^2^). We excluded diabetic subjects with insulin treatment and subjects with fasting glucose > 7 mmol/L [25]. The nature and risks of the study were explained, and all subjects gave their written informed consent. The study protocol was approved by the Ethics Committee of the Hospital District of Southwest Finland and conducted according to the principles of the Declaration of Helsinki.

### 2.2. Study Design

The F group (further subdivided into OLM and OOM) and the CTR group were studied at baseline, and the F group again after 4 months of a supervised resistance training intervention program. The intervention consisted of training sessions three times a week, for 60 min. Subjects participated on average in 78.6% of all training sessions and there were no significant between the group differences in adherence to the supervised resistance training program. Details on the exercise program are reported in our previous publication [25]. The examinations (at baseline and after intervention) consisted of brain MR (MRI and DTI) and [^18^F]FDG-PET studies performed in an overnight fasting state.

### 2.3. MRI

Brain imaging involved an T1-weighted scan and a DTI scan with a Philips Gyroscan Intera 1.5T Nova Dual scanner (Philips, Best, The Netherlands) at the Turku PET Centre. T1-weighted images were acquired following parameters: 3D FFE (flip angle 30°), SENSE 2, TR 25 ms, TE 4.6 ms, acquisition voxel size 1.17/1.17/1.00 mm, and reconstructed image size 256 × 256 × 256 with voxel size 1.09/1.09/1.0 (with 0.5 mm of gap between slices) mm. The DTI were acquired with the following parameters: SENSE 2, single-shot spin-echo echo-planar-imaging sequence (half-scan), 32 directions at b = 1000 s/mm^2^, TR 4286 ms, TE 89 ms, acquisition voxel size 2.32/2.36/2.32 mm, and reconstructed image size 122 × 122 × 60 with voxels size isotropic 2.32 mm.

### 2.4. Voxel Based Morphometry (VBM)

T1-weighted images were segmented with VBM8 toolbox (http://dbm.neuro.uni-jena.de/vbm8, accessed on 23 March 2023) in SPM8 (Wellcome Department of Cognitive Neurology, Institute of Neurology, University College London) implemented in MATLAB R2013a (The MathWorks, Inc., Natick, MA, USA). The DARTEL algorithm [40] and non-linear correction for GM and WM that takes into account the changes in brain volume was applied. Finally, the segmented, normalized, and modulated images were smoothed using a Gaussian kernel of 10 mm full width at half maximum (FWHM). After checking the sample homogeneity of GM segmented images using covariance with a VBM8 tool, two images from the frail group were excluded because their covariance was less than 2 SD. GM density and WM density images were further analyzed with the Statistical Parametric Mapping Toolbox (SPM 8) in MATLAB 2013a.

### 2.5. Cortical Thickness Analysis

The brain was segmented to GM and WM with FreeSurfer’s image analysis suite (version 5.3.0, https://surfer.nmr.mgh.harvard.edu/pub/dist/freesurfer/5.3.0/, accessed on 23 March 2023) processing pipeline for measurements of cortex area, thickness, folding index, gaussian curvature, and local gyrification index (LGI). The 33 default cortical ROIs of Freesurfer were analyzed for statistical difference between groups as described in the statistical analysis section. The cerebral cortical surfaces were also analyzed with Surfstat (available at https://www.math.mcgill.ca/keith/surfstat/doc/SurfStat/index.html, accessed on 23 March 2023) to obtain surface-based difference maps between groups as well as before and after the intervention.

### 2.6. DTI and Tract-Based Spatial Statistics (TBSS)

The DTI data was pre-processed with FSL tools (FMRIB Software Library, Version 5.0.4; FMRIB, Oxford, UK) [41] running on OS X, and with in-house bash scripts. The 32 directions were quality controlled with DTIprep tool (version 1.2.4) [42]. The images were corrected for motion and eddy current distortions. After this, the b-vectors (diffusion directions) were rotated correspondingly to the image rotations. BET was used to remove non-brain matter [43]. Next, the diffusion tensors were fitted (using dtifit, FSL 5.0.4) and Fractional Anisotrophy (FA), Mean Diffusivity (MD), and Radial Diffusivity (RD) maps were computed.

Tract-based spatial statistics (TBSS) analysis was performed according to default procedure [44,45]. First, individual images were normalized: aligned to the default template (FMRIB58 at 1 mm resolution) using FLIRT for affine co-registration, followed by FNIRT for non-linear co-registration. Second, the mean FA image was generated and reduced to FA skeleton, which represents the centers of all tracts common to the entire group. The mean FA skeleton was then placed at a threshold FA value of >0.3 to exclude peripheral tracts and minimize partial volume. Finally, each participant’s aligned FA, MD, and RD images were projected onto the mean FA skeleton and the resulting data were fed into voxelwise permutation-based analysis (5000 permutations). We adopted the threshold-free cluster enhancement with family-wise error correction at *p*-values less than 0.05. To investigate the white matter from DTI maps in a region-wise analysis, the individual normalized maps (FA, MD, RD) were masked with the white matter tractography atlas JHU in ICBM space. Group baseline comparisons for F vs. CTR, OLM vs. CTR and OOM vs. CTR, as well as longitudinal comparisons (before and after intervention) for F, OLM and OOM have been tabulated for each ROI. The results, both uncorrected and FDR-corrected, have been reported in the Supplemental Tables.

### 2.7. Positron Emission Tomography (PET)—Brain and Skeletal Muscle Glucose Uptake during Clamp

The PET examination was performed one day before or after the MR examination day. During the day of the PET examination, body fat percentage was measured using a bioelectrical impedance scale (Omron, model HBF-400-E, Kyoto, Japan). BMI was calculated via the formula BMI = body weight (kg)/height (m^2^). During the PET examination, the hyperinsulinemic euglycemic clamp was used to induce an insulin-stimulated state, while maintaining normoglycemia. The technique allows to estimate whole body insulin sensitivity (M-Value) [46] and glucose uptake (GU, [^18^F]FDG-PET) in different organs during insulin stimulation. The protocol for the acquisition of the PET images during clamp has already been described elsewhere [25]. In brief, at the beginning of the study, one catheter was inserted on each arm of the participant: left arm for glucose, insulin, and [^18^F]FDG injection; right arm for saline infusion and blood sampling. Insulin (Actrapid, Novo Nordisk, Copenhagen, Denmark) was infused at rate of 1 mU kg^−1^ min^−1^. Normoglycemia was maintained via a variable infusion rate of 20% glucose and controlled every 5–10 min via plasma glucose determinations from arterialized blood. After 40–50 min, when a steady state condition was achieved, the participant was moved in the PET/CT scanner (Discovery 690, General Electric (GE) Medical systems, Milwaukee, WI, USA). [^18^F]FDG was produced as previously described [25]. The scanning of the different body regions started after the radiotracer ([^18^F]FDG) injection. In order to obtain the first 2 min of the input function from the heart cavity, the chest area was scanned first for 35 min (frames 8 × 15 s, 3 × 60 s, 6 × 300 s). The brain was scanned approximately 77 min after injection for 15 min (frames 5 × 180 s). Images were corrected for dead time, decay, photon attenuation, and motion. Images in Bq/mL units were converted in GU parametric images, via the graphical method (Gjedde-Patlak plot) [47] utilizing an image-derived input function of [^18^F]FDG activity (heart scan, first 2 min) and from the arterialized venous blood samples (collected frequently from the 2nd min during the PET scans), as previously detailed [25]. Parametric images were subsequently co-registered and normalized to the MNI template and fed into SPM.

### 2.8. Cognitive Assessments

We used cognitive measures obtained in a different study involving the subjects enrolled [48]. CERAD (The Consortium to Establish a Registry for Alzheimer’s Disease) tests were performed at two time points: at 3 years (on average) before the baseline imaging study day and at 6 months (on average) after the end of intervention. Some 4 OLM and 2 OOM subjects were excluded because the CERAD tests were performed before the end of the treatment. For these assessments, CTR were also tested twice (at baseline and after 4 months with no treatment); different to the imaging studies, for which only the baseline study is available. Details of the neurocognitive testing are reported elsewhere [48].

### 2.9. Biochemical Analysis

Plasma fasting glucose was measured with an automatized enzymatic assay (Cobas 8000, Roche Diagnostics GmbH, Mannheim, Germany). Serum fasting insulin was measured using automatized electro-chemiluminescence immunoassay (ECLIA; Cobas 8000, Roche Diagnostics GmbH, Mannheim, Germany).

### 2.10. Statistical Analysis

Statistical analysis was performed using SPSS IBM 23.0 statistical program (SPSS Inc., Chicago, IL, USA). Data are reported as mean ± standard error of mean (SEM). The statistical analysis of the basic characteristics was carried out as described previously [41]. VBM results were placed at threshold *p* ≤ 0.05 and FDR corrected with minimum cluster size of 10 mm^3^. SPM was used to compare the brain images (MR, WM density and GM density and PET, GU) of the different study groups (The frail group as a whole (F), as well as OOM, OLM and CTR) with two sample *t*-tests. To evaluate the effect of treatment paired *t*-tests were performed within SPM. Since the Freesurfer analysis was performed on multiple ROIs, statistical significance was raised to *p* ≤ 0.01 (uncorrected *p* values reported) and FDR correction was also performed. Additionally, an analysis of the surface of the cortex, calculated with Freesurfer, was performed with Surfstat, and a *p*-value of less than or equal to 0.05 (FDR corrected) was considered significant.

### 2.11. Correlations

Voxel-by-voxel correlations between PET (GU) and MRI (WM density, GM density) images, after they were re-sliced to match the same image matrix, were performed; a *p*-value of less than or equal to 0.05 (FDR corrected) was considered significant.

Metabolic and cognitive assessments were entered as single covariates in one sample *t*-tests models for the SPM analysis of GU, WM density, and GM density for each separate group (F, CTR, OLM, OOM). Results were placed at threshold *p* ≤ 0.05 and FDR corrected with minimum cluster size of 10 mm^3^. To correct for multiple comparisons, a *p*-value of less than 0.01 was considered statistically significant. GM density correlations were corrected for age, since age resulted related with GM in women [49].

## 3. Results

General characteristics of the study groups at baseline and before and after the intervention are presented in Table 1 and Table 2, respectively. The mean age and BMI of the groups were not statistically different and only women were included. The age range of the groups varied slightly, but all fell within a range of 10 ± 2 years. (More information on the subject characterization can be found in our previous article [25].

### 3.1. Comparison between Frail (F) and Non-Frail Control (CTR) Elderly Women

**Figure 1:**
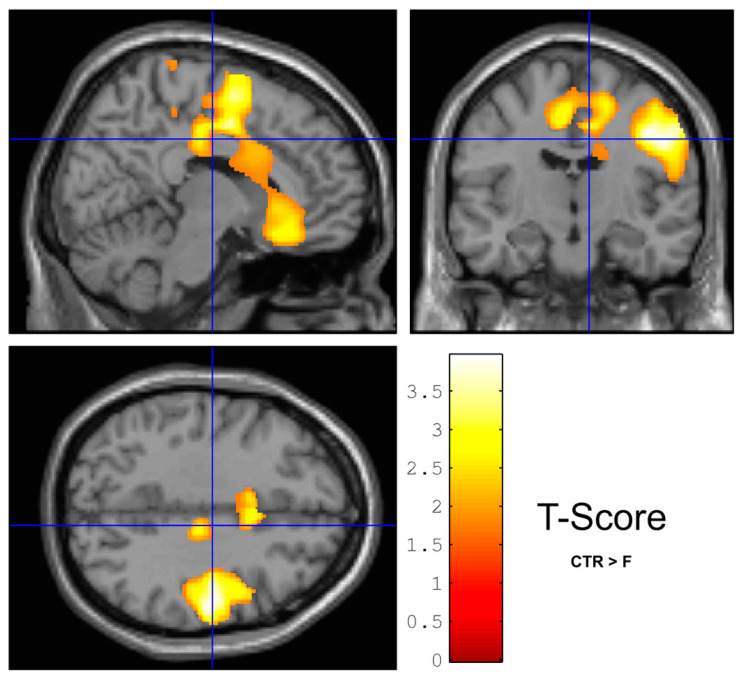
WM density was greater in controls (CTR) than in frails (F) with statistical minima in zones close to the right pre- and post-central regions. The yellow color marks for *t*-value of the statistical test between the groups, with darkest visualized color at *p* = 0.05.

WM density (VBM) in the right pre- and post-central region of the brain was significantly higher in the CTR compared to the F group (Figure 1). GM density (VBM) was not different between frail (F) and non-frail control (CTR) women, when controlling for age. These results were confirmed by the Freesurfer analysis (33 F vs. 8 CTR), which could not establish any significant difference in area, thickness, folding index, or gaussian curvature comparing the two groups in the same regions following FDR correction. LGI was not different among the groups after FDR correction. Cortical thickness was also not different between F and CTR after FDR correction. TBSS and ROI white matter atlas-based (Supplemental Material) analyses (FA, MD and RD maps) did not reveal difference between groups after multiple comparison correction. Brain GU was not different between groups.

Regarding the cognitive assessments, the CERAD questionnaire evidenced that in the verbal fluency and the word recognition tests CTR performed significantly better than F (*p* ≤ 0.05, both).

### 3.2. Comparison between Offspring of Lean Mothers (OLM) and Offspring of Obese Mothers (OOM) (and CTR): The Role of Maternal Obesity

**Figure 2:**
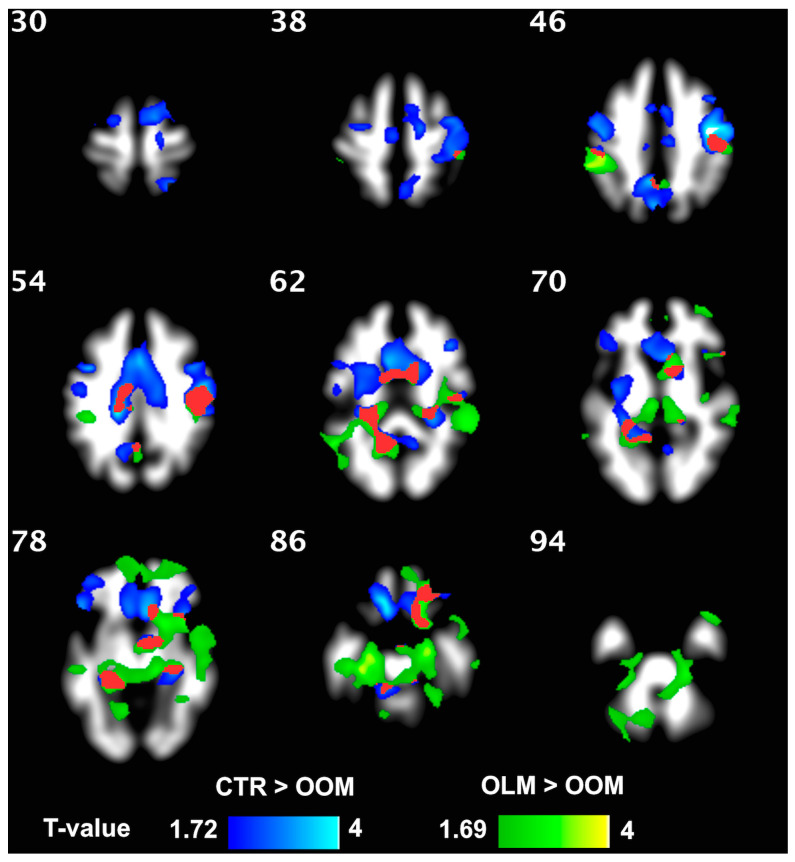
WM density was greater in OLM compared to OOM (*green*) and was greater in CTR than in OOM (*blue*). The two-color scales represent the *t*-values of the statistical test. The lowest visualized *t*-value in the color scales corresponds to *p* = 0.05. The *red* area shows the intersections between the two statistical maps.

WM density was not different between OLM and CTR groups but was higher in OLM compared to OOM and in CTR compared to OOM in several regions, as evidenced from the VBM analysis (Figure 2); significant clusters were found for both comparisons, even after correction for age. WM volume (Freesurfer) in the left caudate was higher in CTR vs. OOM and in OLM vs. OOM (both *p* ≤ 0.01); no difference was found between OLM and CTR.

VBM analysis of GM density did not result in significant differences between the three groups (OLM, OOM, and CTR), after correcting for age.

Freesurfer results confirmed VBM analysis when corrected for FDR, and no difference remained significant. Cortical thickness statistical surface maps, LGI, TBSS, and ROI white matter atlas-based (Supplemental Material) analyses (FA, MD and RD maps), did not show statistical differences between OLM, OOM, and CTR after multiple comparison correction.

Brain glucose uptake was not different between groups.

### 3.3. Effect of Resistance Training on the Brain Structure of Frail Elderly Women

**Figure 3:**
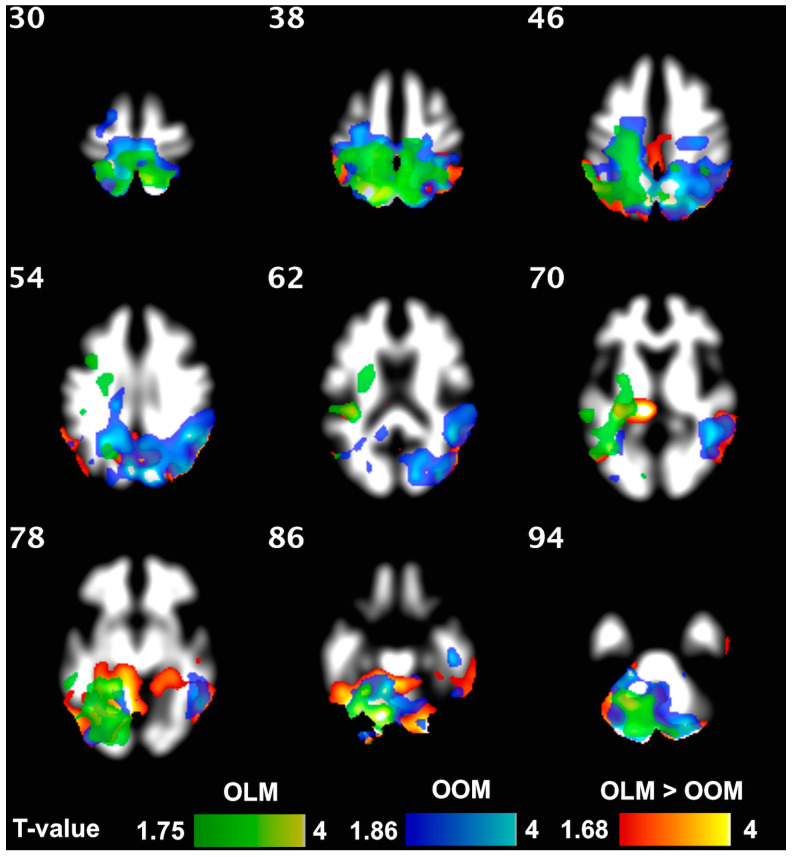
Exercise increased WM density both in OLM (*green*) and OOM (*blue*). The two-color scales represent the *t*-values of the statistical test. The lowest visualized *t*-value in the color scales corresponds to *p* = 0.05. The brain areas where the exercise vs. group interaction resulted in significant effects are highlighted in *orange*. Within these areas, the OLM group exhibited greater increases in WM density than the OOM group following 4 months of training.

A resistance training intervention markedly influenced the brain structure of the F group. WM density increased significantly in the F group, especially in the cerebellum. This was also observed in the OLM and OOM group, separately. Furthermore, OLM showed an increase in WM density in parietal superior regions, while OOM exhibited an increase in both the cuneal and precuneal regions (Figure 3). Taking into account the interaction of the group, OLM resulted to have a greater increase in WM density than OOM, especially in cerebellum (Figure 3), but also in superior parietal regions. The intervention-associated increase in WM density was confirmed by a corresponding increase in the left cortical WM volume in the frail group (*p* = 0.009), while a significant increase in corpus callosum (middle anterior) was observed exclusively in the OLM group.

GM density (VBM) did not change after training when correcting for age. The Freesurfer analysis confirmed the VBM analysis; neither cortical thickness nor LGI changed after intervention in F.

Brain GU did not change after exercise intervention in F, OLM and OOM groups.

### 3.4. Cognitive Functioning

CERAD sum (CS) increased significantly after intervention in F (*p* = 0.022) but also in CTR after re-test (*p* = 0.048) when tested with a paired *t*-test. With a repeated measure general linear model, the effect of resistance training between groups (the interaction between the two factors) was tested, but it was not statistically significant (*p* = 0.094). When checking for the stability of the CS via linear regression of CS at baseline and after treatment [50], there was no significant correlation between the CS variables in the CTR group; hence, there was an indication of instability and random effect causing the increase, while in F, the correlation between CS at baseline and after treatment was positive (ρ = 0.70) and significant (*p* < 0.001), indicating higher stability.

The CS did not increase significantly in OLM nor in OOM, when analyzed separately. When testing the stability of the variable, CS at baseline correlated positively with CS after treatment in OLM (ρ = 0.722, *p* = 0.002), but not in OOM (ρ = 0.422, *p* = 0.298).

## 4. Discussion

Our main finding is that WM density was reduced in frail women (F) compared to control (CTR) women, especially in the right pre- and post-central region. Additionally, main differences between OOM and OLM were found at the level of WM. Furthermore, both VBM and Freesurfer analyses independently confirmed that WM density atrophy was reversed by resistance training in frail elderly women, especially in OLM compared to OOM.

According to a cross-sectional study of 30–90 years old subjects [9], WM volume decreases at a faster rate than GM volume in both cerebrum and cerebellum even during aging. We expanded the study of Jernigan et al. [9], performed on normal volunteers, investigating frail elderly women and we observed a reduced WM density in F compared to CTR. Considering that our groups were not statistically different in age, this novel finding shows that the frail condition per se is an indicator of WM density reduction. Chen et al. reported a reduced GM volume in frail and prefrail populations according to Fried definition of frailty [51,52], clustering weight loss, exhaustion, low physical activity, weakness (grip strength), and slowness (walk time). They found that GM was reduced in the brains of elderly men and women, especially in the cerebellum in relation to slowness, weakness, and low activity. Kant et al. did also find a reduction in total brain volume and GM volume in frail compared to non-frail subjects, but not pre-frail compared to non-frail subjects [53]. In the present study, we have examined women characterized by the weakness component (in some regards less severe than full-blown frailty syndrome) and performed an extensive series of analyses (VBM, Freesurfer and TBSS) and multiple comparisons corrections. We did not detect differences in GM characteristics (GM volume, cortical thickness, cortex area, thickness, folding index, gaussian curvature, and LGI) between the frail and control groups, in line with the report by Kant et al. [53].

Ageing has been associated with lowered fasting brain GU [54]. A study from our group provided evidence that brain glucose uptake in response to insulin, as measured during hyperinsulinemic euglycemic clamp such as in the current study, is associated negatively with age [55]. In the current study, we did not find differences in brain glucose uptake during clamp between any of the groups. Our previous study in minipigs showed that brain insulin resistance in the offspring of high-fat fed mothers was more evident at younger than older ages [56]. Furthermore, in frail subjects from a memory clinic, frailty was associated with presence of deep white matter hyperintensities but not hypometabolism as assessed by PET/SPECT clinical reports (PET scans in fasting condition and not quantitatively assessed) [57].

Maternal obesity affects between 20.2 and 34% of pregnant women and is a major factor influencing offspring health, as shown in animal and human studies [12]. One DTI study in infants showed that WM integrity was reduced in OOM compared to OLM in several regions [21], and another group showed that local and distal functional connectivity is different in infants with different maternal BMI [58]. In line with these findings, observing the subjects in adulthood, we found that WM volume (Freesurfer) and density (VBM) were lower in OOM than CTR and OLM, with no difference between OLM and CTR. This could be due to altered trajectories of longitudinal decline in white matter integrity. Unfortunately, we could not study longitudinal decline due to our study design; however, others have done so in elderly participants finding different clusters of white matter decline [59]. We hypothesized that, together with a WM decline (observed), we would have found alterations in DTI FA and MD; namely, an increase in MD and a decrease in FA in frail subjects according to previous literature [2]. We observed no significant differences in DTI maps (FA, MD, RD) with TBSS or ROI level analysis (JHU atlas, 48 ROIs) after correcting for multiple comparisons; however, some differences were observed uncorrected, and the Supplemental Tables can be of aid in formulating hypotheses on specific regions in future studies with bigger sample sizes.

Exercise programs in frail elderly differ in their content, setting, delivery, duration, and frequency [29,30,60], and uncertainties remain in the intensity level that is effective [28,29]. In this study, weak grip strength-type frail women were enrolled in moderate intensity resistance training, and we sought to find improvement in their health status. In our previous publications, we reported that the intervention improved whole body and skeletal muscle glucose metabolism in the OOM group [25], and the suppression of endogenous glucose production in the whole frail group [61]. In the current study, however, using the same cohort, we found no significant differences between groups in brain GU before and after intervention. In our study, the subjects remained supine during the tracer uptake period to ensure optimal conditions for the hyperinsulinemic euglycemic clamp. The design was chosen due to the nature of our multi-organ study and was possibly suboptimal for the purpose of measuring brain glucose uptake. As suggested by the results of a recent paper, changes in brain GU can be detected after exercise intervention in older adults when the tracer is taken up from the subjects’ bodies during walking [62].

Instead, resistance training increased WM density in F, both OOM and OLM, especially in the cerebellum region, which is a motor area. When comparing the increments of WM density between OLM and OOM, OLM had a higher increase especially in cerebellum. As reviewed by Thomas et al., aerobic exercise increases capillary diameter in the cerebellum in small animals and elderly humans [63]. The changes in brain white matter observed in this study are in line with effects of resistance training in healthy older adults; differently from aerobic training, that is believed to predominantly effect gray matter [64]. In support of this notion, underlying mechanisms of plasticity (such as angiogenesis, synaptogenesis, and neurogenesis) that are associated with brain volumetric changes, have in fact all been associated with exercise training interventions in animals [64].

In this study, the F group performed worse than CTR in two of the CERAD subtests. This confirms what has been previously found; namely, that frailty is characterized by cognitive decline and incident Alzheimer Disease [65]. Cognitive performance was improved in F after exercise but also in CTR (after 4 months without intervention). Possible explanations for an increase in cognitive performance without intervention are: (a) learning effect; (b) stress in the first testing (less stressed by the second session, as the situation is more familiar); (c) active memorization of some tests. However, the stability check (correlation test–retest) [50] highlighted differences. In F, but not in CTR, baseline results predicted post-intervention results as the two measurements were correlated, which is indicative of stability along time, less random effect, or less variability [50,66]. Stability is considered a beneficial indicator of resistance to cognitive decline and to major cognitive change [67]. The improvement of cognition after exercise is in line with results from a recent RCT with 12 weeks of combined exercise intervention (aerobic + resistance + ThaiChi) in frail old adults. An improvement on cognitive functions and brain activations was found, and the authors also found that a marker of neural efficiency was ameliorated [68]. This amelioration in neural efficiency could be speculated to be an effect of our exercise intervention when considered together with the improvement of cognitive performance (in a compatible time frame), and especially, the white matter density findings. It is in fact known that white matter tracts and gray matter are equally essential for the brain connectivity and therefore brain function and cognitive ability [69].

Our study had some limitations. WM integrity, WM density, and GM density have been associated with increased body fat in elderly females [70,71]; however, our study groups F vs. CTR were originally selected to not be statistically different in BMI and age, and OLM and OOM did not differ in BMI and age. Hence, we are unable to validate that association. We recognize that the study sample is relatively small. It must be noted that the study was logistically demanding, since most patients were not living in the local area and underwent repeated imaging studies [25]. Though our sample size was relatively small, it was sufficient to detect relevant differences. On the other hand, TBSS and WM ROI-based analyses did not survive correction for multiple comparisons. Error in ROI sampling of the DTI might have been affected by subject motion and systematic errors, including B-spatial distribution in DTI (BSD-DTI) [72], of which the latter effect can be assessed employing anisotropic phantoms. However, in this study these were not considered due to lack of evidence at the time of the investigations. We enrolled only women to improve homogeneity of the findings, and this makes our conclusions difficult to generalize. Women have higher WM volume than males [8], which may have reinforced our likelihood to observe group differences and changes in this structure. CERAD examinations were performed three years before the imaging studies and six months after the end of the intervention. This might have led to an underestimation of cognitive improvements in the exercise study.

## 5. Conclusions

Frail elderly women had decreased WM density compared to similar age non-frail women. Women born to an overweight compared to a normal weight mother showed lower WM density, suggesting that early programming has long-term effects on brain structure, which may contribute to frailty. A four-month resistance training normalized WM density in both OLM and OOM, and (although assessed with a different timeline) might have contributed to a greater stability in cognitive performance.

## Figures and Tables

**Table 1:** General characteristics at baseline; comparisons between groups.

	Controls (*n* = 9)	Frails (*n* = 37)	*p*	OLM (*n* = 20)	OOM (*n* = 17)	*p*
Age (years)	71.7 ± 1.0	71.8 ± 0.5	0.98 ^	72.2 ± 0.6	71.5 ± 0.9	0.27 ^
BMI (kg/m^2^)	27.8 ± 1.4	27.2 ± 0.8	0.70	26.6 ± 1.1	27.9 ± 1.1	0.40
fP-Glucose (mmol/L)	6.4 ± 0.3	6.0 ± 0.1	0.21 ^	6.0 ± 0.2	5.9 ± 0.2	0.99 ^
fP-Insulin (mU/I)	10.7 ± 2.2	9.5 ± 0.9	0.60 ^	9.6 ± 1.4	9.4 ± 1.2	0.78 ^

Frails (OLM + OOM); OLM = Offspring of lean mothers; OOM = Offspring of obese mothers. Mean ± SEM. ^ Non-parametric Mann Whitney-U test (Exact sig.).

**Table 2:** Effect of exercise on general characteristics in frail groups sub-divided according to maternal BMI.

	Offspring of Lean Mothers			Offspring of Obese Mothers		
	Baseline (*n* = 20)	Treatment (*n* = 19)	*p*	Baseline (*n* = 17)	Treatment (*n* = 16)	*p*
BMI (kg/m^2^)	26.6 ± 1.1	27.1 ± 1.1	0.52	27.9 ± 1.1	27.6 ± 1.2	0.92
fP-Glucose (mmol/L)	6.0 ± 0.2	6.1 ± 0.2	0.43	5.9 ± 0.2	5.8 ± 0.2	0.92
fP-Insulin (mU/I)	9.6 ± 1.4	9.5 ± 1.1	0.70	9.4 ± 1.2	9.7 ± 1.4	0.56

Mean ± SEM.

## Data Availability

The data presented in this study are available on request from the corresponding author. The data are not publicly available due to their sensitive and clinical nature.

## Supplementary Materials

The following supporting information can be downloaded at: https://www.mdpi.com/article/10.3390/jcm12072684/s1, Supplementary Tables of DTI results.

## Abbreviations

BMI, Body mass index; CS, CERAD sum; CTR, Control group; DTI, Diffusion tensor imaging; F, Frail group (including both OLM and OOM groups); GM, gray matter; OLM, Offspring of lean mothers (group); OOM, Offspring of obese mothers (group); PET, Positron emission tomography; WM, white matter
