# Supplementary material for: Resistance Training Increases White Matter Density in Frail Elderly Women
Source: J Clin Med. 2023 Apr 4;12(7):2684. doi: 10.3390/jcm12072684 (PMC10094827; doi:10.3390/jcm12072684)
Supplement: Supplementary file 1 [file jcm-12-02684-s001.zip › jcm-2217024-supplementary.pdf]

Supplementary Tables of DTI results

Fractional anisotropy (FA) (DTI) - Frail vs control subjects - (JHU white-matter tractography atlas) - Uncorrected p-values

	Control (N=9)	Frail (N=35)	Total (N=44)	p value
Middle cerebellar peduncle				0.943
- Mean (SD)	0.406 (0.045)	0.408 (0.056)	0.407 (0.053)	
- Range	0.296 - 0.453	0.263 - 0.478	0.263 - 0.478	
Pontine crossing tract				0.152
- Mean (SD)	0.385 (0.034)	0.406 (0.040)	0.402 (0.039)	
- Range	0.324 - 0.428	0.338 - 0.483	0.324 - 0.483	
Genu of corpus callosum				0.685
- Mean (SD)	0.441 (0.041)	0.436 (0.026)	0.437 (0.029)	
- Range	0.371 - 0.486	0.383 - 0.498	0.371 - 0.498	
Body of corpus callosum				0.300
- Mean (SD)	0.501 (0.043)	0.487 (0.035)	0.490 (0.036)	
- Range	0.425 - 0.554	0.426 - 0.553	0.425 - 0.554	
Splenium of corpus callosum				0.655
- Mean (SD)	0.586 (0.052)	0.592 (0.033)	0.591 (0.037)	
- Range	0.494 - 0.669	0.533 - 0.669	0.494 - 0.669	
Fornix				0.486
- Mean (SD)	0.242 (0.037)	0.251 (0.035)	0.249 (0.035)	
- Range	0.193 - 0.281	0.184 - 0.332	0.184 - 0.332	
Corticospinal tract R				0.198
- Mean (SD)	0.444 (0.042)	0.467 (0.048)	0.463 (0.047)	
- Range	0.369 - 0.499	0.309 - 0.555	0.309 - 0.555	
Corticospinal tract L				0.102
- Mean (SD)	0.446 (0.044)	0.474 (0.046)	0.469 (0.047)	
- Range	0.370 - 0.482	0.350 - 0.598	0.350 - 0.598	
Medial lemniscus R				0.819
- Mean (SD)	0.445 (0.034)	0.449 (0.049)	0.448 (0.046)	
- Range	0.403 - 0.514	0.335 - 0.560	0.335 - 0.560	
Medial lemniscus L				0.884
- Mean (SD)	0.453 (0.036)	0.455 (0.055)	0.455 (0.052)	
- Range	0.404 - 0.506	0.353 - 0.598	0.353 - 0.598	
Inferior cerebellar peduncle R				0.649
- Mean (SD)	0.350 (0.048)	0.358 (0.047)	0.356 (0.047)	
- Range	0.282 - 0.442	0.270 - 0.449	0.270 - 0.449	
Inferior cerebellar peduncle L				0.726
- Mean (SD)	0.361 (0.032)	0.355 (0.045)	0.356 (0.042)	
- Range	0.328 - 0.422	0.230 - 0.432	0.230 - 0.432	
Superior cerebellar peduncle R				0.751
- Mean (SD)	0.471 (0.062)	0.464 (0.066)	0.465 (0.065)	
- Range	0.346 - 0.530	0.298 - 0.568	0.298 - 0.568	
Superior cerebellar peduncle L				0.802
- Mean (SD)	0.447 (0.057)	0.442 (0.051)	0.443 (0.051)	
- Range	0.309 - 0.487	0.308 - 0.521	0.308 - 0.521	
Cerebral peduncle R				0.425
- Mean (SD)	0.569 (0.026)	0.561 (0.027)	0.562 (0.027)	
- Range	0.513 - 0.598	0.495 - 0.625	0.495 - 0.625	
Cerebral peduncle L				0.987
- Mean (SD)	0.572 (0.036)	0.572 (0.028)	0.572 (0.029)	
- Range	0.485 - 0.610	0.491 - 0.615	0.485 - 0.615	
Anterior limb of internal capsule R				0.315
- Mean (SD)	0.444 (0.040)	0.432 (0.030)	0.434 (0.032)	
- Range	0.357 - 0.486	0.341 - 0.482	0.341 - 0.486	
Anterior limb of internal capsule L				0.878
- Mean (SD)	0.442 (0.038)	0.440 (0.030)	0.441 (0.032)	
- Range	0.355 - 0.481	0.389 - 0.491	0.355 - 0.491	
Posterior limb of internal capsule R				0.603
- Mean (SD)	0.533 (0.033)	0.528 (0.026)	0.529 (0.027)	
- Range	0.463 - 0.574	0.472 - 0.586	0.463 - 0.586	
Posterior limb of internal capsule L				0.660
- Mean (SD)	0.543 (0.042)	0.539 (0.024)	0.540 (0.028)	
- Range	0.453 - 0.591	0.478 - 0.595	0.453 - 0.595	

	Control (N=9)	Frail (N=35)	Total (N=44)	p value
Retrolenticular part of internal capsule R				0.648
- Mean (SD)	0.442 (0.037)	0.437 (0.027)	0.438 (0.029)	
- Range	0.375 - 0.501	0.389 - 0.518	0.375 - 0.518	
Retrolenticular part of internal capsule L				0.347
- Mean (SD)	0.459 (0.031)	0.448 (0.030)	0.450 (0.030)	
- Range	0.405 - 0.496	0.402 - 0.522	0.402 - 0.522	
Anterior corona radiata R				0.787
- Mean (SD)	0.334 (0.029)	0.331 (0.029)	0.332 (0.029)	
- Range	0.273 - 0.364	0.263 - 0.383	0.263 - 0.383	
Anterior corona radiata L				0.996
- Mean (SD)	0.334 (0.027)	0.334 (0.029)	0.334 (0.028)	
- Range	0.280 - 0.358	0.270 - 0.392	0.270 - 0.392	
Superior corona radiata R				0.204
- Mean (SD)	0.416 (0.037)	0.400 (0.032)	0.403 (0.033)	
- Range	0.366 - 0.470	0.315 - 0.451	0.315 - 0.470	
Superior corona radiata L				0.527
- Mean (SD)	0.414 (0.031)	0.405 (0.036)	0.407 (0.035)	
- Range	0.356 - 0.452	0.311 - 0.478	0.311 - 0.478	
Posterior corona radiata R				0.144
- Mean (SD)	0.430 (0.047)	0.409 (0.037)	0.413 (0.039)	
- Range	0.373 - 0.513	0.331 - 0.480	0.331 - 0.513	
Posterior corona radiata L				0.476
- Mean (SD)	0.412 (0.034)	0.401 (0.042)	0.403 (0.040)	
- Range	0.368 - 0.466	0.308 - 0.491	0.308 - 0.491	
Posterior thalamic radiation R				0.252
- Mean (SD)	0.472 (0.034)	0.457 (0.035)	0.460 (0.035)	
- Range	0.415 - 0.543	0.372 - 0.529	0.372 - 0.543	
Posterior thalamic radiation L				0.902
- Mean (SD)	0.447 (0.044)	0.448 (0.030)	0.448 (0.032)	
- Range	0.358 - 0.494	0.388 - 0.504	0.358 - 0.504	
Sagittal stratum R				0.118
- Mean (SD)	0.447 (0.032)	0.430 (0.028)	0.433 (0.029)	
- Range	0.406 - 0.506	0.391 - 0.507	0.391 - 0.507	
Sagittal stratum L				0.302
- Mean (SD)	0.404 (0.031)	0.393 (0.026)	0.395 (0.027)	
- Range	0.349 - 0.444	0.346 - 0.464	0.346 - 0.464	
External capsule R				0.398
- Mean (SD)	0.336 (0.026)	0.328 (0.026)	0.329 (0.026)	
- Range	0.295 - 0.366	0.280 - 0.412	0.280 - 0.412	
External capsule L				0.517
- Mean (SD)	0.343 (0.021)	0.337 (0.025)	0.339 (0.024)	
- Range	0.295 - 0.371	0.302 - 0.399	0.295 - 0.399	
Cingulum cingulate gyrus R				0.233
- Mean (SD)	0.368 (0.040)	0.355 (0.024)	0.358 (0.028)	
- Range	0.293 - 0.425	0.303 - 0.414	0.293 - 0.425	
Cingulum cingulate gyrus L				0.213
- Mean (SD)	0.383 (0.041)	0.367 (0.030)	0.370 (0.033)	
- Range	0.329 - 0.430	0.310 - 0.445	0.310 - 0.445	
Cingulum hippocampus R				0.344
- Mean (SD)	0.297 (0.030)	0.284 (0.037)	0.287 (0.036)	
- Range	0.250 - 0.340	0.213 - 0.371	0.213 - 0.371	
Cingulum hippocampus L				0.699
- Mean (SD)	0.273 (0.032)	0.278 (0.033)	0.277 (0.032)	
- Range	0.226 - 0.332	0.204 - 0.366	0.204 - 0.366	
Fornix R				0.247
- Mean (SD)	0.375 (0.031)	0.361 (0.031)	0.364 (0.031)	
- Range	0.317 - 0.412	0.306 - 0.426	0.306 - 0.426	
Fornix L				0.840
- Mean (SD)	0.383 (0.027)	0.380 (0.033)	0.381 (0.031)	
- Range	0.331 - 0.432	0.326 - 0.438	0.326 - 0.438	
Superior longitudinal fasciculus R				0.399
- Mean (SD)	0.395 (0.035)	0.386 (0.024)	0.388 (0.027)	

	Control (N=9)	Frail (N=35)	Total (N=44)	p value
- Range	0.344 - 0.442	0.346 - 0.426	0.344 - 0.442	
Superior longitudinal fasciculus L				0.405
- Mean (SD)	0.399 (0.040)	0.389 (0.029)	0.391 (0.032)	
- Range	0.327 - 0.463	0.320 - 0.437	0.320 - 0.463	
Superior fronto occipital fasciculus R				0.485
- Mean (SD)	0.359 (0.047)	0.348 (0.042)	0.350 (0.043)	
- Range	0.279 - 0.415	0.248 - 0.441	0.248 - 0.441	
Superior fronto occipital fasciculus L				0.584
- Mean (SD)	0.324 (0.053)	0.333 (0.038)	0.331 (0.041)	
- Range	0.234 - 0.389	0.243 - 0.410	0.234 - 0.410	
Uncinate fasciculus R				0.386
- Mean (SD)	0.381 (0.049)	0.393 (0.035)	0.390 (0.038)	
- Range	0.288 - 0.424	0.328 - 0.462	0.288 - 0.462	
Uncinate fasciculus L				0.594
- Mean (SD)	0.385 (0.036)	0.393 (0.041)	0.391 (0.040)	
- Range	0.333 - 0.434	0.319 - 0.462	0.319 - 0.462	
Tapetum R				0.715
- Mean (SD)	0.343 (0.037)	0.338 (0.032)	0.339 (0.033)	
- Range	0.268 - 0.386	0.264 - 0.387	0.264 - 0.387	
Tapetum L				0.503
- Mean (SD)	0.307 (0.018)	0.300 (0.029)	0.301 (0.027)	
- Range	0.278 - 0.332	0.228 - 0.372	0.228 - 0.372	

Fractional anisotropy (FA) (DTI) - Frail vs control subjects - (JHU white-matter tractography atlas) - FDR corrected p-values

	Control (N=9)	Frail (N=35)	Total (N=44)	p value
Middle cerebellar peduncle				0.985
- Mean (SD)	0.406 (0.045)	0.408 (0.056)	0.407 (0.053)	
- Range	0.296 - 0.453	0.263 - 0.478	0.263 - 0.478	
Pontine crossing tract				0.942
- Mean (SD)	0.385 (0.034)	0.406 (0.040)	0.402 (0.039)	
- Range	0.324 - 0.428	0.338 - 0.483	0.324 - 0.483	
Genu of corpus callosum				0.942
- Mean (SD)	0.441 (0.041)	0.436 (0.026)	0.437 (0.029)	
- Range	0.371 - 0.486	0.383 - 0.498	0.371 - 0.498	
Body of corpus callosum				0.942
- Mean (SD)	0.501 (0.043)	0.487 (0.035)	0.490 (0.036)	
- Range	0.425 - 0.554	0.426 - 0.553	0.425 - 0.554	
Splenium of corpus callosum				0.942
- Mean (SD)	0.586 (0.052)	0.592 (0.033)	0.591 (0.037)	
- Range	0.494 - 0.669	0.533 - 0.669	0.494 - 0.669	
Fornix				0.942
- Mean (SD)	0.242 (0.037)	0.251 (0.035)	0.249 (0.035)	
- Range	0.193 - 0.281	0.184 - 0.332	0.184 - 0.332	
Corticospinal tract R				0.942
- Mean (SD)	0.444 (0.042)	0.467 (0.048)	0.463 (0.047)	
- Range	0.369 - 0.499	0.309 - 0.555	0.309 - 0.555	
Corticospinal tract L				0.942
- Mean (SD)	0.446 (0.044)	0.474 (0.046)	0.469 (0.047)	
- Range	0.370 - 0.482	0.350 - 0.598	0.350 - 0.598	
Medial lemniscus R				0.958
- Mean (SD)	0.445 (0.034)	0.449 (0.049)	0.448 (0.046)	
- Range	0.403 - 0.514	0.335 - 0.560	0.335 - 0.560	
Medial lemniscus L				0.962
- Mean (SD)	0.453 (0.036)	0.455 (0.055)	0.455 (0.052)	
- Range	0.404 - 0.506	0.353 - 0.598	0.353 - 0.598	
Inferior cerebellar peduncle R				0.942
- Mean (SD)	0.350 (0.048)	0.358 (0.047)	0.356 (0.047)	
- Range	0.282 - 0.442	0.270 - 0.449	0.270 - 0.449	
Inferior cerebellar peduncle L				0.942
- Mean (SD)	0.361 (0.032)	0.355 (0.045)	0.356 (0.042)	
- Range	0.328 - 0.422	0.230 - 0.432	0.230 - 0.432	
Superior cerebellar peduncle R				0.949
- Mean (SD)	0.471 (0.062)	0.464 (0.066)	0.465 (0.065)	
- Range	0.346 - 0.530	0.298 - 0.568	0.298 - 0.568	
Superior cerebellar peduncle L				0.958
- Mean (SD)	0.447 (0.057)	0.442 (0.051)	0.443 (0.051)	
- Range	0.309 - 0.487	0.308 - 0.521	0.308 - 0.521	
Cerebral peduncle R				0.942
- Mean (SD)	0.569 (0.026)	0.561 (0.027)	0.562 (0.027)	
- Range	0.513 - 0.598	0.495 - 0.625	0.495 - 0.625	
Cerebral peduncle L				0.996
- Mean (SD)	0.572 (0.036)	0.572 (0.028)	0.572 (0.029)	
- Range	0.485 - 0.610	0.491 - 0.615	0.485 - 0.615	
Anterior limb of internal capsule R				0.942
- Mean (SD)	0.444 (0.040)	0.432 (0.030)	0.434 (0.032)	
- Range	0.357 - 0.486	0.341 - 0.482	0.341 - 0.486	
Anterior limb of internal capsule L				0.962
- Mean (SD)	0.442 (0.038)	0.440 (0.030)	0.441 (0.032)	
- Range	0.355 - 0.481	0.389 - 0.491	0.355 - 0.491	
Posterior limb of internal capsule R				0.942
- Mean (SD)	0.533 (0.033)	0.528 (0.026)	0.529 (0.027)	
- Range	0.463 - 0.574	0.472 - 0.586	0.463 - 0.586	
Posterior limb of internal capsule L				0.942
- Mean (SD)	0.543 (0.042)	0.539 (0.024)	0.540 (0.028)	
- Range	0.453 - 0.591	0.478 - 0.595	0.453 - 0.595	

	Control (N=9)	Frail (N=35)	Total (N=44)	p value
Retrolenticular part of internal capsule R				0.942
- Mean (SD)	0.442 (0.037)	0.437 (0.027)	0.438 (0.029)	
- Range	0.375 - 0.501	0.389 - 0.518	0.375 - 0.518	
Retrolenticular part of internal capsule L				0.942
- Mean (SD)	0.459 (0.031)	0.448 (0.030)	0.450 (0.030)	
- Range	0.405 - 0.496	0.402 - 0.522	0.402 - 0.522	
Anterior corona radiata R				0.958
- Mean (SD)	0.334 (0.029)	0.331 (0.029)	0.332 (0.029)	
- Range	0.273 - 0.364	0.263 - 0.383	0.263 - 0.383	
Anterior corona radiata L				0.996
- Mean (SD)	0.334 (0.027)	0.334 (0.029)	0.334 (0.028)	
- Range	0.280 - 0.358	0.270 - 0.392	0.270 - 0.392	
Superior corona radiata R				0.942
- Mean (SD)	0.416 (0.037)	0.400 (0.032)	0.403 (0.033)	
- Range	0.366 - 0.470	0.315 - 0.451	0.315 - 0.470	
Superior corona radiata L				0.942
- Mean (SD)	0.414 (0.031)	0.405 (0.036)	0.407 (0.035)	
- Range	0.356 - 0.452	0.311 - 0.478	0.311 - 0.478	
Posterior corona radiata R				0.942
- Mean (SD)	0.430 (0.047)	0.409 (0.037)	0.413 (0.039)	
- Range	0.373 - 0.513	0.331 - 0.480	0.331 - 0.513	
Posterior corona radiata L				0.942
- Mean (SD)	0.412 (0.034)	0.401 (0.042)	0.403 (0.040)	
- Range	0.368 - 0.466	0.308 - 0.491	0.308 - 0.491	
Posterior thalamic radiation R				0.942
- Mean (SD)	0.472 (0.034)	0.457 (0.035)	0.460 (0.035)	
- Range	0.415 - 0.543	0.372 - 0.529	0.372 - 0.543	
Posterior thalamic radiation L				0.962
- Mean (SD)	0.447 (0.044)	0.448 (0.030)	0.448 (0.032)	
- Range	0.358 - 0.494	0.388 - 0.504	0.358 - 0.504	
Sagittal stratum R				0.942
- Mean (SD)	0.447 (0.032)	0.430 (0.028)	0.433 (0.029)	
- Range	0.406 - 0.506	0.391 - 0.507	0.391 - 0.507	
Sagittal stratum L				0.942
- Mean (SD)	0.404 (0.031)	0.393 (0.026)	0.395 (0.027)	
- Range	0.349 - 0.444	0.346 - 0.464	0.346 - 0.464	
External capsule R				0.942
- Mean (SD)	0.336 (0.026)	0.328 (0.026)	0.329 (0.026)	
- Range	0.295 - 0.366	0.280 - 0.412	0.280 - 0.412	
External capsule L				0.942
- Mean (SD)	0.343 (0.021)	0.337 (0.025)	0.339 (0.024)	
- Range	0.295 - 0.371	0.302 - 0.399	0.295 - 0.399	
Cingulum cingulate gyrus R				0.942
- Mean (SD)	0.368 (0.040)	0.355 (0.024)	0.358 (0.028)	
- Range	0.293 - 0.425	0.303 - 0.414	0.293 - 0.425	
Cingulum cingulate gyrus L				0.942
- Mean (SD)	0.383 (0.041)	0.367 (0.030)	0.370 (0.033)	
- Range	0.329 - 0.430	0.310 - 0.445	0.310 - 0.445	
Cingulum hippocampus R				0.942
- Mean (SD)	0.297 (0.030)	0.284 (0.037)	0.287 (0.036)	
- Range	0.250 - 0.340	0.213 - 0.371	0.213 - 0.371	
Cingulum hippocampus L				0.942
- Mean (SD)	0.273 (0.032)	0.278 (0.033)	0.277 (0.032)	
- Range	0.226 - 0.332	0.204 - 0.366	0.204 - 0.366	
Fornix R				0.942
- Mean (SD)	0.375 (0.031)	0.361 (0.031)	0.364 (0.031)	
- Range	0.317 - 0.412	0.306 - 0.426	0.306 - 0.426	
Fornix L				0.960
- Mean (SD)	0.383 (0.027)	0.380 (0.033)	0.381 (0.031)	
- Range	0.331 - 0.432	0.326 - 0.438	0.326 - 0.438	
Superior longitudinal fasciculus R				0.942
- Mean (SD)	0.395 (0.035)	0.386 (0.024)	0.388 (0.027)	

	Control (N=9)	Frail (N=35)	Total (N=44)	p value
- Range	0.344 - 0.442	0.346 - 0.426	0.344 - 0.442	
Superior longitudinal fasciculus L				0.942
- Mean (SD)	0.399 (0.040)	0.389 (0.029)	0.391 (0.032)	
- Range	0.327 - 0.463	0.320 - 0.437	0.320 - 0.463	
Superior fronto occipital fasciculus R				0.942
- Mean (SD)	0.359 (0.047)	0.348 (0.042)	0.350 (0.043)	
- Range	0.279 - 0.415	0.248 - 0.441	0.248 - 0.441	
Superior fronto occipital fasciculus L				0.942
- Mean (SD)	0.324 (0.053)	0.333 (0.038)	0.331 (0.041)	
- Range	0.234 - 0.389	0.243 - 0.410	0.234 - 0.410	
Uncinate fasciculus R				0.942
- Mean (SD)	0.381 (0.049)	0.393 (0.035)	0.390 (0.038)	
- Range	0.288 - 0.424	0.328 - 0.462	0.288 - 0.462	
Uncinate fasciculus L				0.942
- Mean (SD)	0.385 (0.036)	0.393 (0.041)	0.391 (0.040)	
- Range	0.333 - 0.434	0.319 - 0.462	0.319 - 0.462	
Tapetum R				0.942
- Mean (SD)	0.343 (0.037)	0.338 (0.032)	0.339 (0.033)	
- Range	0.268 - 0.386	0.264 - 0.387	0.264 - 0.387	
Tapetum L				0.942
- Mean (SD)	0.307 (0.018)	0.300 (0.029)	0.301 (0.027)	
- Range	0.278 - 0.332	0.228 - 0.372	0.228 - 0.372	

Mean diffusivity (MD, (10^-3)*(mm^2)/s) (DTI) - Frail vs control subjects - (JHU white-matter tractography atlas) - Uncorrected p-values

	Control (N=9)	Frail (N=35)	Total (N=44)	p value
Middle cerebellar peduncle				0.888
- Mean (SD)	1.089 (0.156)	1.082 (0.133)	1.083 (0.136)	
- Range	0.906 - 1.447	0.857 - 1.433	0.857 - 1.447	
Pontine crossing tract				0.622
- Mean (SD)	0.797 (0.070)	0.815 (0.099)	0.811 (0.093)	
- Range	0.723 - 0.951	0.683 - 1.162	0.683 - 1.162	
Genu of corpus callosum				0.906
- Mean (SD)	1.370 (0.119)	1.364 (0.139)	1.366 (0.134)	
- Range	1.240 - 1.614	1.042 - 1.688	1.042 - 1.688	
Body of corpus callosum				0.787
- Mean (SD)	1.112 (0.116)	1.122 (0.097)	1.120 (0.099)	
- Range	0.990 - 1.319	0.984 - 1.370	0.984 - 1.370	
Splenium of corpus callosum				0.491
- Mean (SD)	1.046 (0.151)	1.018 (0.093)	1.024 (0.106)	
- Range	0.888 - 1.318	0.886 - 1.287	0.886 - 1.318	
Fornix				0.260
- Mean (SD)	2.495 (0.323)	2.369 (0.289)	2.395 (0.297)	
- Range	2.048 - 2.977	1.853 - 2.857	1.853 - 2.977	
Corticospinal tract R				0.790
- Mean (SD)	0.865 (0.094)	0.874 (0.098)	0.872 (0.096)	
- Range	0.754 - 1.012	0.724 - 1.250	0.724 - 1.250	
Corticospinal tract L				0.583
- Mean (SD)	0.824 (0.108)	0.843 (0.087)	0.839 (0.090)	
- Range	0.736 - 1.037	0.723 - 1.159	0.723 - 1.159	
Medial lemniscus R				0.282
- Mean (SD)	0.846 (0.068)	0.887 (0.106)	0.879 (0.100)	
- Range	0.782 - 0.982	0.697 - 1.210	0.697 - 1.210	
Medial lemniscus L				0.227
- Mean (SD)	0.834 (0.046)	0.863 (0.066)	0.857 (0.063)	
- Range	0.783 - 0.935	0.695 - 1.034	0.695 - 1.034	
Inferior cerebellar peduncle R				0.973
- Mean (SD)	1.136 (0.189)	1.133 (0.227)	1.134 (0.217)	
- Range	0.880 - 1.524	0.831 - 1.875	0.831 - 1.875	
Inferior cerebellar peduncle L				0.768
- Mean (SD)	1.057 (0.148)	1.078 (0.190)	1.073 (0.181)	
- Range	0.954 - 1.406	0.864 - 1.748	0.864 - 1.748	
Superior cerebellar peduncle R				0.657
- Mean (SD)	1.209 (0.241)	1.238 (0.154)	1.232 (0.172)	
- Range	1.000 - 1.829	0.890 - 1.532	0.890 - 1.829	
Superior cerebellar peduncle L				0.609
- Mean (SD)	1.265 (0.175)	1.300 (0.178)	1.293 (0.176)	
- Range	1.010 - 1.622	0.913 - 1.623	0.913 - 1.623	
Cerebral peduncle R				0.826
- Mean (SD)	0.874 (0.071)	0.879 (0.047)	0.878 (0.052)	
- Range	0.794 - 1.038	0.785 - 0.971	0.785 - 1.038	
Cerebral peduncle L				0.900
- Mean (SD)	0.842 (0.060)	0.840 (0.046)	0.840 (0.048)	
- Range	0.788 - 0.985	0.733 - 0.927	0.733 - 0.985	
Anterior limb of internal capsule R				0.736
- Mean (SD)	0.846 (0.087)	0.855 (0.063)	0.853 (0.068)	
- Range	0.773 - 1.055	0.774 - 1.020	0.773 - 1.055	
Anterior limb of internal capsule L				0.848
- Mean (SD)	0.877 (0.110)	0.871 (0.074)	0.872 (0.081)	
- Range	0.775 - 1.112	0.753 - 1.040	0.753 - 1.112	
Posterior limb of internal capsule R				0.735
- Mean (SD)	0.770 (0.050)	0.765 (0.034)	0.766 (0.037)	
- Range	0.689 - 0.846	0.705 - 0.857	0.689 - 0.857	
Posterior limb of internal capsule L				0.725
- Mean (SD)	0.770 (0.060)	0.764 (0.033)	0.765 (0.039)	
- Range	0.694 - 0.882	0.703 - 0.847	0.694 - 0.882	

	Control (N=9)	Frail (N=35)	Total (N=44)	p value
Retrolenticular part of internal capsule R				0.433
- Mean (SD)	0.934 (0.097)	0.911 (0.070)	0.916 (0.075)	
- Range	0.827 - 1.105	0.780 - 1.062	0.780 - 1.105	
Retrolenticular part of internal capsule L				0.837
- Mean (SD)	0.936 (0.111)	0.929 (0.070)	0.931 (0.079)	
- Range	0.837 - 1.171	0.814 - 1.088	0.814 - 1.171	
Anterior corona radiata R				0.870
- Mean (SD)	0.940 (0.079)	0.947 (0.107)	0.945 (0.101)	
- Range	0.835 - 1.070	0.790 - 1.248	0.790 - 1.248	
Anterior corona radiata L				0.702
- Mean (SD)	0.927 (0.066)	0.942 (0.115)	0.939 (0.107)	
- Range	0.841 - 1.030	0.791 - 1.298	0.791 - 1.298	
Superior corona radiata R				0.912
- Mean (SD)	0.821 (0.059)	0.818 (0.078)	0.819 (0.074)	
- Range	0.740 - 0.927	0.708 - 1.096	0.708 - 1.096	
Superior corona radiata L				0.959
- Mean (SD)	0.835 (0.068)	0.837 (0.085)	0.836 (0.081)	
- Range	0.743 - 0.949	0.705 - 1.099	0.705 - 1.099	
Posterior corona radiata R				0.671
- Mean (SD)	0.967 (0.131)	0.949 (0.100)	0.953 (0.106)	
- Range	0.818 - 1.163	0.780 - 1.178	0.780 - 1.178	
Posterior corona radiata L				0.393
- Mean (SD)	1.028 (0.189)	0.984 (0.121)	0.993 (0.136)	
- Range	0.871 - 1.331	0.800 - 1.328	0.800 - 1.331	
Posterior thalamic radiation R				0.837
- Mean (SD)	1.002 (0.097)	1.012 (0.131)	1.010 (0.124)	
- Range	0.857 - 1.174	0.828 - 1.510	0.828 - 1.510	
Posterior thalamic radiation L				0.828
- Mean (SD)	1.137 (0.115)	1.124 (0.170)	1.126 (0.159)	
- Range	0.993 - 1.352	0.934 - 1.606	0.934 - 1.606	
Sagittal stratum R				0.790
- Mean (SD)	0.986 (0.089)	0.996 (0.095)	0.994 (0.093)	
- Range	0.833 - 1.078	0.844 - 1.335	0.833 - 1.335	
Sagittal stratum L				0.912
- Mean (SD)	1.074 (0.143)	1.069 (0.111)	1.070 (0.117)	
- Range	0.903 - 1.379	0.881 - 1.305	0.881 - 1.379	
External capsule R				0.462
- Mean (SD)	0.842 (0.067)	0.859 (0.059)	0.856 (0.060)	
- Range	0.764 - 0.953	0.753 - 0.996	0.753 - 0.996	
External capsule L				0.473
- Mean (SD)	0.822 (0.048)	0.837 (0.056)	0.834 (0.054)	
- Range	0.766 - 0.893	0.765 - 0.953	0.765 - 0.953	
Cingulum cingulate gyrus R				0.213
- Mean (SD)	0.950 (0.134)	0.910 (0.067)	0.918 (0.085)	
- Range	0.802 - 1.199	0.782 - 1.082	0.782 - 1.199	
Cingulum cingulate gyrus L				0.904
- Mean (SD)	0.945 (0.100)	0.948 (0.080)	0.948 (0.083)	
- Range	0.835 - 1.085	0.792 - 1.115	0.792 - 1.115	
Cingulum hippocampus R				0.568
- Mean (SD)	1.106 (0.143)	1.076 (0.137)	1.082 (0.137)	
- Range	0.917 - 1.303	0.887 - 1.523	0.887 - 1.523	
Cingulum hippocampus L				0.048
- Mean (SD)	1.256 (0.183)	1.125 (0.170)	1.152 (0.179)	
- Range	1.007 - 1.484	0.849 - 1.734	0.849 - 1.734	
Fornix R				0.570
- Mean (SD)	1.055 (0.138)	1.085 (0.137)	1.079 (0.136)	
- Range	0.866 - 1.261	0.844 - 1.408	0.844 - 1.408	
Fornix L				0.820
- Mean (SD)	1.015 (0.136)	1.026 (0.125)	1.023 (0.126)	
- Range	0.851 - 1.246	0.841 - 1.331	0.841 - 1.331	
Superior longitudinal fasciculus R				0.882
- Mean (SD)	0.805 (0.052)	0.802 (0.050)	0.802 (0.050)	

	Control (N=9)	Frail (N=35)	Total (N=44)	p value
- Range	0.758 - 0.898	0.727 - 0.950	0.727 - 0.950	
Superior longitudinal fasciculus L				0.520
- Mean (SD)	0.781 (0.038)	0.792 (0.047)	0.790 (0.045)	
- Range	0.732 - 0.839	0.728 - 0.904	0.728 - 0.904	
Superior fronto occipital fasciculus R				0.798
- Mean (SD)	0.964 (0.172)	0.944 (0.210)	0.948 (0.201)	
- Range	0.718 - 1.300	0.723 - 1.571	0.718 - 1.571	
Superior fronto occipital fasciculus L				0.762
- Mean (SD)	1.088 (0.246)	1.059 (0.259)	1.065 (0.254)	
- Range	0.836 - 1.504	0.795 - 1.863	0.795 - 1.863	
Uncinate fasciculus R				0.962
- Mean (SD)	0.913 (0.179)	0.910 (0.117)	0.911 (0.130)	
- Range	0.782 - 1.375	0.790 - 1.267	0.782 - 1.375	
Uncinate fasciculus L				0.364
- Mean (SD)	0.846 (0.064)	0.878 (0.102)	0.872 (0.096)	
- Range	0.777 - 0.939	0.780 - 1.287	0.777 - 1.287	
Tapetum R				0.836
- Mean (SD)	2.032 (0.341)	2.061 (0.390)	2.055 (0.377)	
- Range	1.379 - 2.374	1.141 - 2.681	1.141 - 2.681	
Tapetum L				0.557
- Mean (SD)	2.404 (0.471)	2.310 (0.410)	2.329 (0.419)	
- Range	1.674 - 3.197	1.407 - 2.936	1.407 - 3.197	

Mean diffusivity (MD, (10^-3)*(mm^2)/s) (DTI) - Frail vs control subjects - (JHU white-matter tractography atlas) - FDR corrected p-values

	Control (N=9)	Frail (N=35)	Total (N=44)	p value
Middle cerebellar peduncle				0.973
- Mean (SD)	1.089 (0.156)	1.082 (0.133)	1.083 (0.136)	
- Range	0.906 - 1.447	0.857 - 1.433	0.857 - 1.447	
Pontine crossing tract				0.973
- Mean (SD)	0.797 (0.070)	0.815 (0.099)	0.811 (0.093)	
- Range	0.723 - 0.951	0.683 - 1.162	0.683 - 1.162	
Genu of corpus callosum				0.973
- Mean (SD)	1.370 (0.119)	1.364 (0.139)	1.366 (0.134)	
- Range	1.240 - 1.614	1.042 - 1.688	1.042 - 1.688	
Body of corpus callosum				0.973
- Mean (SD)	1.112 (0.116)	1.122 (0.097)	1.120 (0.099)	
- Range	0.990 - 1.319	0.984 - 1.370	0.984 - 1.370	
Splenium of corpus callosum				0.973
- Mean (SD)	1.046 (0.151)	1.018 (0.093)	1.024 (0.106)	
- Range	0.888 - 1.318	0.886 - 1.287	0.886 - 1.318	
Fornix				0.973
- Mean (SD)	2.495 (0.323)	2.369 (0.289)	2.395 (0.297)	
- Range	2.048 - 2.977	1.853 - 2.857	1.853 - 2.977	
Corticospinal tract R				0.973
- Mean (SD)	0.865 (0.094)	0.874 (0.098)	0.872 (0.096)	
- Range	0.754 - 1.012	0.724 - 1.250	0.724 - 1.250	
Corticospinal tract L				0.973
- Mean (SD)	0.824 (0.108)	0.843 (0.087)	0.839 (0.090)	
- Range	0.736 - 1.037	0.723 - 1.159	0.723 - 1.159	
Medial lemniscus R				0.973
- Mean (SD)	0.846 (0.068)	0.887 (0.106)	0.879 (0.100)	
- Range	0.782 - 0.982	0.697 - 1.210	0.697 - 1.210	
Medial lemniscus L				0.973
- Mean (SD)	0.834 (0.046)	0.863 (0.066)	0.857 (0.063)	
- Range	0.783 - 0.935	0.695 - 1.034	0.695 - 1.034	
Inferior cerebellar peduncle R				0.973
- Mean (SD)	1.136 (0.189)	1.133 (0.227)	1.134 (0.217)	
- Range	0.880 - 1.524	0.831 - 1.875	0.831 - 1.875	
Inferior cerebellar peduncle L				0.973
- Mean (SD)	1.057 (0.148)	1.078 (0.190)	1.073 (0.181)	
- Range	0.954 - 1.406	0.864 - 1.748	0.864 - 1.748	
Superior cerebellar peduncle R				0.973
- Mean (SD)	1.209 (0.241)	1.238 (0.154)	1.232 (0.172)	
- Range	1.000 - 1.829	0.890 - 1.532	0.890 - 1.829	
Superior cerebellar peduncle L				0.973
- Mean (SD)	1.265 (0.175)	1.300 (0.178)	1.293 (0.176)	
- Range	1.010 - 1.622	0.913 - 1.623	0.913 - 1.623	
Cerebral peduncle R				0.973
- Mean (SD)	0.874 (0.071)	0.879 (0.047)	0.878 (0.052)	
- Range	0.794 - 1.038	0.785 - 0.971	0.785 - 1.038	
Cerebral peduncle L				0.973
- Mean (SD)	0.842 (0.060)	0.840 (0.046)	0.840 (0.048)	
- Range	0.788 - 0.985	0.733 - 0.927	0.733 - 0.985	
Anterior limb of internal capsule R				0.973
- Mean (SD)	0.846 (0.087)	0.855 (0.063)	0.853 (0.068)	
- Range	0.773 - 1.055	0.774 - 1.020	0.773 - 1.055	
Anterior limb of internal capsule L				0.973
- Mean (SD)	0.877 (0.110)	0.871 (0.074)	0.872 (0.081)	
- Range	0.775 - 1.112	0.753 - 1.040	0.753 - 1.112	
Posterior limb of internal capsule R				0.973
- Mean (SD)	0.770 (0.050)	0.765 (0.034)	0.766 (0.037)	
- Range	0.689 - 0.846	0.705 - 0.857	0.689 - 0.857	
Posterior limb of internal capsule L				0.973
- Mean (SD)	0.770 (0.060)	0.764 (0.033)	0.765 (0.039)	
- Range	0.694 - 0.882	0.703 - 0.847	0.694 - 0.882	

	Control (N=9)	Frail (N=35)	Total (N=44)	p value
Retrolenticular part of internal capsule R				0.973
- Mean (SD)	0.934 (0.097)	0.911 (0.070)	0.916 (0.075)	
- Range	0.827 - 1.105	0.780 - 1.062	0.780 - 1.105	
Retrolenticular part of internal capsule L				0.973
- Mean (SD)	0.936 (0.111)	0.929 (0.070)	0.931 (0.079)	
- Range	0.837 - 1.171	0.814 - 1.088	0.814 - 1.171	
Anterior corona radiata R				0.973
- Mean (SD)	0.940 (0.079)	0.947 (0.107)	0.945 (0.101)	
- Range	0.835 - 1.070	0.790 - 1.248	0.790 - 1.248	
Anterior corona radiata L				0.973
- Mean (SD)	0.927 (0.066)	0.942 (0.115)	0.939 (0.107)	
- Range	0.841 - 1.030	0.791 - 1.298	0.791 - 1.298	
Superior corona radiata R				0.973
- Mean (SD)	0.821 (0.059)	0.818 (0.078)	0.819 (0.074)	
- Range	0.740 - 0.927	0.708 - 1.096	0.708 - 1.096	
Superior corona radiata L				0.973
- Mean (SD)	0.835 (0.068)	0.837 (0.085)	0.836 (0.081)	
- Range	0.743 - 0.949	0.705 - 1.099	0.705 - 1.099	
Posterior corona radiata R				0.973
- Mean (SD)	0.967 (0.131)	0.949 (0.100)	0.953 (0.106)	
- Range	0.818 - 1.163	0.780 - 1.178	0.780 - 1.178	
Posterior corona radiata L				0.973
- Mean (SD)	1.028 (0.189)	0.984 (0.121)	0.993 (0.136)	
- Range	0.871 - 1.331	0.800 - 1.328	0.800 - 1.331	
Posterior thalamic radiation R				0.973
- Mean (SD)	1.002 (0.097)	1.012 (0.131)	1.010 (0.124)	
- Range	0.857 - 1.174	0.828 - 1.510	0.828 - 1.510	
Posterior thalamic radiation L				0.973
- Mean (SD)	1.137 (0.115)	1.124 (0.170)	1.126 (0.159)	
- Range	0.993 - 1.352	0.934 - 1.606	0.934 - 1.606	
Sagittal stratum R				0.973
- Mean (SD)	0.986 (0.089)	0.996 (0.095)	0.994 (0.093)	
- Range	0.833 - 1.078	0.844 - 1.335	0.833 - 1.335	
Sagittal stratum L				0.973
- Mean (SD)	1.074 (0.143)	1.069 (0.111)	1.070 (0.117)	
- Range	0.903 - 1.379	0.881 - 1.305	0.881 - 1.379	
External capsule R				0.973
- Mean (SD)	0.842 (0.067)	0.859 (0.059)	0.856 (0.060)	
- Range	0.764 - 0.953	0.753 - 0.996	0.753 - 0.996	
External capsule L				0.973
- Mean (SD)	0.822 (0.048)	0.837 (0.056)	0.834 (0.054)	
- Range	0.766 - 0.893	0.765 - 0.953	0.765 - 0.953	
Cingulum cingulate gyrus R				0.973
- Mean (SD)	0.950 (0.134)	0.910 (0.067)	0.918 (0.085)	
- Range	0.802 - 1.199	0.782 - 1.082	0.782 - 1.199	
Cingulum cingulate gyrus L				0.973
- Mean (SD)	0.945 (0.100)	0.948 (0.080)	0.948 (0.083)	
- Range	0.835 - 1.085	0.792 - 1.115	0.792 - 1.115	
Cingulum hippocampus R				0.973
- Mean (SD)	1.106 (0.143)	1.076 (0.137)	1.082 (0.137)	
- Range	0.917 - 1.303	0.887 - 1.523	0.887 - 1.523	
Cingulum hippocampus L				0.973
- Mean (SD)	1.256 (0.183)	1.125 (0.170)	1.152 (0.179)	
- Range	1.007 - 1.484	0.849 - 1.734	0.849 - 1.734	
Fornix R				0.973
- Mean (SD)	1.055 (0.138)	1.085 (0.137)	1.079 (0.136)	
- Range	0.866 - 1.261	0.844 - 1.408	0.844 - 1.408	
Fornix L				0.973
- Mean (SD)	1.015 (0.136)	1.026 (0.125)	1.023 (0.126)	
- Range	0.851 - 1.246	0.841 - 1.331	0.841 - 1.331	
Superior longitudinal fasciculus R				0.973
- Mean (SD)	0.805 (0.052)	0.802 (0.050)	0.802 (0.050)	

	Control (N=9)	Frail (N=35)	Total (N=44)	p value
- Range	0.758 - 0.898	0.727 - 0.950	0.727 - 0.950	
Superior longitudinal fasciculus L				0.973
- Mean (SD)	0.781 (0.038)	0.792 (0.047)	0.790 (0.045)	
- Range	0.732 - 0.839	0.728 - 0.904	0.728 - 0.904	
Superior fronto occipital fasciculus R				0.973
- Mean (SD)	0.964 (0.172)	0.944 (0.210)	0.948 (0.201)	
- Range	0.718 - 1.300	0.723 - 1.571	0.718 - 1.571	
Superior fronto occipital fasciculus L				0.973
- Mean (SD)	1.088 (0.246)	1.059 (0.259)	1.065 (0.254)	
- Range	0.836 - 1.504	0.795 - 1.863	0.795 - 1.863	
Uncinate fasciculus R				0.973
- Mean (SD)	0.913 (0.179)	0.910 (0.117)	0.911 (0.130)	
- Range	0.782 - 1.375	0.790 - 1.267	0.782 - 1.375	
Uncinate fasciculus L				0.973
- Mean (SD)	0.846 (0.064)	0.878 (0.102)	0.872 (0.096)	
- Range	0.777 - 0.939	0.780 - 1.287	0.777 - 1.287	
Tapetum R				0.973
- Mean (SD)	2.032 (0.341)	2.061 (0.390)	2.055 (0.377)	
- Range	1.379 - 2.374	1.141 - 2.681	1.141 - 2.681	
Tapetum L				0.973
- Mean (SD)	2.404 (0.471)	2.310 (0.410)	2.329 (0.419)	
- Range	1.674 - 3.197	1.407 - 2.936	1.407 - 3.197	

Radial diffusivity (RD, (10^-3)*(mm^2)/s) (DTI) - Frail vs control subjects - (JHU white-matter tractography atlas) - Uncorrected p-values

	Control (N=9)	Frail (N=35)	Total (N=44)	p value
Middle cerebellar peduncle				0.888
- Mean (SD)	1.089 (0.156)	1.082 (0.133)	1.083 (0.136)	
- Range	0.906 - 1.447	0.857 - 1.433	0.857 - 1.447	
Pontine crossing tract				0.622
- Mean (SD)	0.797 (0.070)	0.815 (0.099)	0.811 (0.093)	
- Range	0.723 - 0.951	0.683 - 1.162	0.683 - 1.162	
Genu of corpus callosum				0.906
- Mean (SD)	1.370 (0.119)	1.364 (0.139)	1.366 (0.134)	
- Range	1.240 - 1.614	1.042 - 1.688	1.042 - 1.688	
Body of corpus callosum				0.787
- Mean (SD)	1.112 (0.116)	1.122 (0.097)	1.120 (0.099)	
- Range	0.990 - 1.319	0.984 - 1.370	0.984 - 1.370	
Splenium of corpus callosum				0.491
- Mean (SD)	1.046 (0.151)	1.018 (0.093)	1.024 (0.106)	
- Range	0.888 - 1.318	0.886 - 1.287	0.886 - 1.318	
Fornix				0.260
- Mean (SD)	2.495 (0.323)	2.369 (0.289)	2.395 (0.297)	
- Range	2.048 - 2.977	1.853 - 2.857	1.853 - 2.977	
Corticospinal tract R				0.790
- Mean (SD)	0.865 (0.094)	0.874 (0.098)	0.872 (0.096)	
- Range	0.754 - 1.012	0.724 - 1.250	0.724 - 1.250	
Corticospinal tract L				0.583
- Mean (SD)	0.824 (0.108)	0.843 (0.087)	0.839 (0.090)	
- Range	0.736 - 1.037	0.723 - 1.159	0.723 - 1.159	
Medial lemniscus R				0.282
- Mean (SD)	0.846 (0.068)	0.887 (0.106)	0.879 (0.100)	
- Range	0.782 - 0.982	0.697 - 1.210	0.697 - 1.210	
Medial lemniscus L				0.227
- Mean (SD)	0.834 (0.046)	0.863 (0.066)	0.857 (0.063)	
- Range	0.783 - 0.935	0.695 - 1.034	0.695 - 1.034	
Inferior cerebellar peduncle R				0.973
- Mean (SD)	1.136 (0.189)	1.133 (0.227)	1.134 (0.217)	
- Range	0.880 - 1.524	0.831 - 1.875	0.831 - 1.875	
Inferior cerebellar peduncle L				0.768
- Mean (SD)	1.057 (0.148)	1.078 (0.190)	1.073 (0.181)	
- Range	0.954 - 1.406	0.864 - 1.748	0.864 - 1.748	
Superior cerebellar peduncle R				0.657
- Mean (SD)	1.209 (0.241)	1.238 (0.154)	1.232 (0.172)	
- Range	1.000 - 1.829	0.890 - 1.532	0.890 - 1.829	
Superior cerebellar peduncle L				0.609
- Mean (SD)	1.265 (0.175)	1.300 (0.178)	1.293 (0.176)	
- Range	1.010 - 1.622	0.913 - 1.623	0.913 - 1.623	
Cerebral peduncle R				0.826
- Mean (SD)	0.874 (0.071)	0.879 (0.047)	0.878 (0.052)	
- Range	0.794 - 1.038	0.785 - 0.971	0.785 - 1.038	
Cerebral peduncle L				0.900
- Mean (SD)	0.842 (0.060)	0.840 (0.046)	0.840 (0.048)	
- Range	0.788 - 0.985	0.733 - 0.927	0.733 - 0.985	
Anterior limb of internal capsule R				0.736
- Mean (SD)	0.846 (0.087)	0.855 (0.063)	0.853 (0.068)	
- Range	0.773 - 1.055	0.774 - 1.020	0.773 - 1.055	
Anterior limb of internal capsule L				0.848
- Mean (SD)	0.877 (0.110)	0.871 (0.074)	0.872 (0.081)	
- Range	0.775 - 1.112	0.753 - 1.040	0.753 - 1.112	
Posterior limb of internal capsule R				0.735
- Mean (SD)	0.770 (0.050)	0.765 (0.034)	0.766 (0.037)	
- Range	0.689 - 0.846	0.705 - 0.857	0.689 - 0.857	
Posterior limb of internal capsule L				0.725
- Mean (SD)	0.770 (0.060)	0.764 (0.033)	0.765 (0.039)	
- Range	0.694 - 0.882	0.703 - 0.847	0.694 - 0.882	

	Control (N=9)	Frail (N=35)	Total (N=44)	p value
Retrolenticular part of internal capsule R				0.433
- Mean (SD)	0.934 (0.097)	0.911 (0.070)	0.916 (0.075)	
- Range	0.827 - 1.105	0.780 - 1.062	0.780 - 1.105	
Retrolenticular part of internal capsule L				0.837
- Mean (SD)	0.936 (0.111)	0.929 (0.070)	0.931 (0.079)	
- Range	0.837 - 1.171	0.814 - 1.088	0.814 - 1.171	
Anterior corona radiata R				0.870
- Mean (SD)	0.940 (0.079)	0.947 (0.107)	0.945 (0.101)	
- Range	0.835 - 1.070	0.790 - 1.248	0.790 - 1.248	
Anterior corona radiata L				0.702
- Mean (SD)	0.927 (0.066)	0.942 (0.115)	0.939 (0.107)	
- Range	0.841 - 1.030	0.791 - 1.298	0.791 - 1.298	
Superior corona radiata R				0.912
- Mean (SD)	0.821 (0.059)	0.818 (0.078)	0.819 (0.074)	
- Range	0.740 - 0.927	0.708 - 1.096	0.708 - 1.096	
Superior corona radiata L				0.959
- Mean (SD)	0.835 (0.068)	0.837 (0.085)	0.836 (0.081)	
- Range	0.743 - 0.949	0.705 - 1.099	0.705 - 1.099	
Posterior corona radiata R				0.671
- Mean (SD)	0.967 (0.131)	0.949 (0.100)	0.953 (0.106)	
- Range	0.818 - 1.163	0.780 - 1.178	0.780 - 1.178	
Posterior corona radiata L				0.393
- Mean (SD)	1.028 (0.189)	0.984 (0.121)	0.993 (0.136)	
- Range	0.871 - 1.331	0.800 - 1.328	0.800 - 1.331	
Posterior thalamic radiation R				0.837
- Mean (SD)	1.002 (0.097)	1.012 (0.131)	1.010 (0.124)	
- Range	0.857 - 1.174	0.828 - 1.510	0.828 - 1.510	
Posterior thalamic radiation L				0.828
- Mean (SD)	1.137 (0.115)	1.124 (0.170)	1.126 (0.159)	
- Range	0.993 - 1.352	0.934 - 1.606	0.934 - 1.606	
Sagittal stratum R				0.790
- Mean (SD)	0.986 (0.089)	0.996 (0.095)	0.994 (0.093)	
- Range	0.833 - 1.078	0.844 - 1.335	0.833 - 1.335	
Sagittal stratum L				0.912
- Mean (SD)	1.074 (0.143)	1.069 (0.111)	1.070 (0.117)	
- Range	0.903 - 1.379	0.881 - 1.305	0.881 - 1.379	
External capsule R				0.462
- Mean (SD)	0.842 (0.067)	0.859 (0.059)	0.856 (0.060)	
- Range	0.764 - 0.953	0.753 - 0.996	0.753 - 0.996	
External capsule L				0.473
- Mean (SD)	0.822 (0.048)	0.837 (0.056)	0.834 (0.054)	
- Range	0.766 - 0.893	0.765 - 0.953	0.765 - 0.953	
Cingulum cingulate gyrus R				0.213
- Mean (SD)	0.950 (0.134)	0.910 (0.067)	0.918 (0.085)	
- Range	0.802 - 1.199	0.782 - 1.082	0.782 - 1.199	
Cingulum cingulate gyrus L				0.904
- Mean (SD)	0.945 (0.100)	0.948 (0.080)	0.948 (0.083)	
- Range	0.835 - 1.085	0.792 - 1.115	0.792 - 1.115	
Cingulum hippocampus R				0.568
- Mean (SD)	1.106 (0.143)	1.076 (0.137)	1.082 (0.137)	
- Range	0.917 - 1.303	0.887 - 1.523	0.887 - 1.523	
Cingulum hippocampus L				0.048
- Mean (SD)	1.256 (0.183)	1.125 (0.170)	1.152 (0.179)	
- Range	1.007 - 1.484	0.849 - 1.734	0.849 - 1.734	
Fornix R				0.570
- Mean (SD)	1.055 (0.138)	1.085 (0.137)	1.079 (0.136)	
- Range	0.866 - 1.261	0.844 - 1.408	0.844 - 1.408	
Fornix L				0.820
- Mean (SD)	1.015 (0.136)	1.026 (0.125)	1.023 (0.126)	
- Range	0.851 - 1.246	0.841 - 1.331	0.841 - 1.331	
Superior longitudinal fasciculus R				0.882
- Mean (SD)	0.805 (0.052)	0.802 (0.050)	0.802 (0.050)	

	Control (N=9)	Frail (N=35)	Total (N=44)	p value
- Range	0.758 - 0.898	0.727 - 0.950	0.727 - 0.950	
Superior longitudinal fasciculus L				0.520
- Mean (SD)	0.781 (0.038)	0.792 (0.047)	0.790 (0.045)	
- Range	0.732 - 0.839	0.728 - 0.904	0.728 - 0.904	
Superior fronto occipital fasciculus R				0.798
- Mean (SD)	0.964 (0.172)	0.944 (0.210)	0.948 (0.201)	
- Range	0.718 - 1.300	0.723 - 1.571	0.718 - 1.571	
Superior fronto occipital fasciculus L				0.762
- Mean (SD)	1.088 (0.246)	1.059 (0.259)	1.065 (0.254)	
- Range	0.836 - 1.504	0.795 - 1.863	0.795 - 1.863	
Uncinate fasciculus R				0.962
- Mean (SD)	0.913 (0.179)	0.910 (0.117)	0.911 (0.130)	
- Range	0.782 - 1.375	0.790 - 1.267	0.782 - 1.375	
Uncinate fasciculus L				0.364
- Mean (SD)	0.846 (0.064)	0.878 (0.102)	0.872 (0.096)	
- Range	0.777 - 0.939	0.780 - 1.287	0.777 - 1.287	
Tapetum R				0.836
- Mean (SD)	2.032 (0.341)	2.061 (0.390)	2.055 (0.377)	
- Range	1.379 - 2.374	1.141 - 2.681	1.141 - 2.681	
Tapetum L				0.557
- Mean (SD)	2.404 (0.471)	2.310 (0.410)	2.329 (0.419)	
- Range	1.674 - 3.197	1.407 - 2.936	1.407 - 3.197	

Radial diffusivity (RD, (10^-3)*(mm^2)/s) (DTI) - Frail vs control subjects - (JHU white-matter tractography atlas) - FDR corrected p-values

	Control (N=9)	Frail (N=35)	Total (N=44)	p value
Middle cerebellar peduncle				0.973
- Mean (SD)	1.089 (0.156)	1.082 (0.133)	1.083 (0.136)	
- Range	0.906 - 1.447	0.857 - 1.433	0.857 - 1.447	
Pontine crossing tract				0.973
- Mean (SD)	0.797 (0.070)	0.815 (0.099)	0.811 (0.093)	
- Range	0.723 - 0.951	0.683 - 1.162	0.683 - 1.162	
Genu of corpus callosum				0.973
- Mean (SD)	1.370 (0.119)	1.364 (0.139)	1.366 (0.134)	
- Range	1.240 - 1.614	1.042 - 1.688	1.042 - 1.688	
Body of corpus callosum				0.973
- Mean (SD)	1.112 (0.116)	1.122 (0.097)	1.120 (0.099)	
- Range	0.990 - 1.319	0.984 - 1.370	0.984 - 1.370	
Splenium of corpus callosum				0.973
- Mean (SD)	1.046 (0.151)	1.018 (0.093)	1.024 (0.106)	
- Range	0.888 - 1.318	0.886 - 1.287	0.886 - 1.318	
Fornix				0.973
- Mean (SD)	2.495 (0.323)	2.369 (0.289)	2.395 (0.297)	
- Range	2.048 - 2.977	1.853 - 2.857	1.853 - 2.977	
Corticospinal tract R				0.973
- Mean (SD)	0.865 (0.094)	0.874 (0.098)	0.872 (0.096)	
- Range	0.754 - 1.012	0.724 - 1.250	0.724 - 1.250	
Corticospinal tract L				0.973
- Mean (SD)	0.824 (0.108)	0.843 (0.087)	0.839 (0.090)	
- Range	0.736 - 1.037	0.723 - 1.159	0.723 - 1.159	
Medial lemniscus R				0.973
- Mean (SD)	0.846 (0.068)	0.887 (0.106)	0.879 (0.100)	
- Range	0.782 - 0.982	0.697 - 1.210	0.697 - 1.210	
Medial lemniscus L				0.973
- Mean (SD)	0.834 (0.046)	0.863 (0.066)	0.857 (0.063)	
- Range	0.783 - 0.935	0.695 - 1.034	0.695 - 1.034	
Inferior cerebellar peduncle R				0.973
- Mean (SD)	1.136 (0.189)	1.133 (0.227)	1.134 (0.217)	
- Range	0.880 - 1.524	0.831 - 1.875	0.831 - 1.875	
Inferior cerebellar peduncle L				0.973
- Mean (SD)	1.057 (0.148)	1.078 (0.190)	1.073 (0.181)	
- Range	0.954 - 1.406	0.864 - 1.748	0.864 - 1.748	
Superior cerebellar peduncle R				0.973
- Mean (SD)	1.209 (0.241)	1.238 (0.154)	1.232 (0.172)	
- Range	1.000 - 1.829	0.890 - 1.532	0.890 - 1.829	
Superior cerebellar peduncle L				0.973
- Mean (SD)	1.265 (0.175)	1.300 (0.178)	1.293 (0.176)	
- Range	1.010 - 1.622	0.913 - 1.623	0.913 - 1.623	
Cerebral peduncle R				0.973
- Mean (SD)	0.874 (0.071)	0.879 (0.047)	0.878 (0.052)	
- Range	0.794 - 1.038	0.785 - 0.971	0.785 - 1.038	
Cerebral peduncle L				0.973
- Mean (SD)	0.842 (0.060)	0.840 (0.046)	0.840 (0.048)	
- Range	0.788 - 0.985	0.733 - 0.927	0.733 - 0.985	
Anterior limb of internal capsule R				0.973
- Mean (SD)	0.846 (0.087)	0.855 (0.063)	0.853 (0.068)	
- Range	0.773 - 1.055	0.774 - 1.020	0.773 - 1.055	
Anterior limb of internal capsule L				0.973
- Mean (SD)	0.877 (0.110)	0.871 (0.074)	0.872 (0.081)	
- Range	0.775 - 1.112	0.753 - 1.040	0.753 - 1.112	
Posterior limb of internal capsule R				0.973
- Mean (SD)	0.770 (0.050)	0.765 (0.034)	0.766 (0.037)	
- Range	0.689 - 0.846	0.705 - 0.857	0.689 - 0.857	
Posterior limb of internal capsule L				0.973
- Mean (SD)	0.770 (0.060)	0.764 (0.033)	0.765 (0.039)	
- Range	0.694 - 0.882	0.703 - 0.847	0.694 - 0.882	

	Control (N=9)	Frail (N=35)	Total (N=44)	p value
Retrolenticular part of internal capsule R				0.973
- Mean (SD)	0.934 (0.097)	0.911 (0.070)	0.916 (0.075)	
- Range	0.827 - 1.105	0.780 - 1.062	0.780 - 1.105	
Retrolenticular part of internal capsule L				0.973
- Mean (SD)	0.936 (0.111)	0.929 (0.070)	0.931 (0.079)	
- Range	0.837 - 1.171	0.814 - 1.088	0.814 - 1.171	
Anterior corona radiata R				0.973
- Mean (SD)	0.940 (0.079)	0.947 (0.107)	0.945 (0.101)	
- Range	0.835 - 1.070	0.790 - 1.248	0.790 - 1.248	
Anterior corona radiata L				0.973
- Mean (SD)	0.927 (0.066)	0.942 (0.115)	0.939 (0.107)	
- Range	0.841 - 1.030	0.791 - 1.298	0.791 - 1.298	
Superior corona radiata R				0.973
- Mean (SD)	0.821 (0.059)	0.818 (0.078)	0.819 (0.074)	
- Range	0.740 - 0.927	0.708 - 1.096	0.708 - 1.096	
Superior corona radiata L				0.973
- Mean (SD)	0.835 (0.068)	0.837 (0.085)	0.836 (0.081)	
- Range	0.743 - 0.949	0.705 - 1.099	0.705 - 1.099	
Posterior corona radiata R				0.973
- Mean (SD)	0.967 (0.131)	0.949 (0.100)	0.953 (0.106)	
- Range	0.818 - 1.163	0.780 - 1.178	0.780 - 1.178	
Posterior corona radiata L				0.973
- Mean (SD)	1.028 (0.189)	0.984 (0.121)	0.993 (0.136)	
- Range	0.871 - 1.331	0.800 - 1.328	0.800 - 1.331	
Posterior thalamic radiation R				0.973
- Mean (SD)	1.002 (0.097)	1.012 (0.131)	1.010 (0.124)	
- Range	0.857 - 1.174	0.828 - 1.510	0.828 - 1.510	
Posterior thalamic radiation L				0.973
- Mean (SD)	1.137 (0.115)	1.124 (0.170)	1.126 (0.159)	
- Range	0.993 - 1.352	0.934 - 1.606	0.934 - 1.606	
Sagittal stratum R				0.973
- Mean (SD)	0.986 (0.089)	0.996 (0.095)	0.994 (0.093)	
- Range	0.833 - 1.078	0.844 - 1.335	0.833 - 1.335	
Sagittal stratum L				0.973
- Mean (SD)	1.074 (0.143)	1.069 (0.111)	1.070 (0.117)	
- Range	0.903 - 1.379	0.881 - 1.305	0.881 - 1.379	
External capsule R				0.973
- Mean (SD)	0.842 (0.067)	0.859 (0.059)	0.856 (0.060)	
- Range	0.764 - 0.953	0.753 - 0.996	0.753 - 0.996	
External capsule L				0.973
- Mean (SD)	0.822 (0.048)	0.837 (0.056)	0.834 (0.054)	
- Range	0.766 - 0.893	0.765 - 0.953	0.765 - 0.953	
Cingulum cingulate gyrus R				0.973
- Mean (SD)	0.950 (0.134)	0.910 (0.067)	0.918 (0.085)	
- Range	0.802 - 1.199	0.782 - 1.082	0.782 - 1.199	
Cingulum cingulate gyrus L				0.973
- Mean (SD)	0.945 (0.100)	0.948 (0.080)	0.948 (0.083)	
- Range	0.835 - 1.085	0.792 - 1.115	0.792 - 1.115	
Cingulum hippocampus R				0.973
- Mean (SD)	1.106 (0.143)	1.076 (0.137)	1.082 (0.137)	
- Range	0.917 - 1.303	0.887 - 1.523	0.887 - 1.523	
Cingulum hippocampus L				0.973
- Mean (SD)	1.256 (0.183)	1.125 (0.170)	1.152 (0.179)	
- Range	1.007 - 1.484	0.849 - 1.734	0.849 - 1.734	
Fornix R				0.973
- Mean (SD)	1.055 (0.138)	1.085 (0.137)	1.079 (0.136)	
- Range	0.866 - 1.261	0.844 - 1.408	0.844 - 1.408	
Fornix L				0.973
- Mean (SD)	1.015 (0.136)	1.026 (0.125)	1.023 (0.126)	
- Range	0.851 - 1.246	0.841 - 1.331	0.841 - 1.331	
Superior longitudinal fasciculus R				0.973
- Mean (SD)	0.805 (0.052)	0.802 (0.050)	0.802 (0.050)	

	Control (N=9)	Frail (N=35)	Total (N=44)	p value
- Range	0.758 - 0.898	0.727 - 0.950	0.727 - 0.950	
Superior longitudinal fasciculus L				0.973
- Mean (SD)	0.781 (0.038)	0.792 (0.047)	0.790 (0.045)	
- Range	0.732 - 0.839	0.728 - 0.904	0.728 - 0.904	
Superior fronto occipital fasciculus R				0.973
- Mean (SD)	0.964 (0.172)	0.944 (0.210)	0.948 (0.201)	
- Range	0.718 - 1.300	0.723 - 1.571	0.718 - 1.571	
Superior fronto occipital fasciculus L				0.973
- Mean (SD)	1.088 (0.246)	1.059 (0.259)	1.065 (0.254)	
- Range	0.836 - 1.504	0.795 - 1.863	0.795 - 1.863	
Uncinate fasciculus R				0.973
- Mean (SD)	0.913 (0.179)	0.910 (0.117)	0.911 (0.130)	
- Range	0.782 - 1.375	0.790 - 1.267	0.782 - 1.375	
Uncinate fasciculus L				0.973
- Mean (SD)	0.846 (0.064)	0.878 (0.102)	0.872 (0.096)	
- Range	0.777 - 0.939	0.780 - 1.287	0.777 - 1.287	
Tapetum R				0.973
- Mean (SD)	2.032 (0.341)	2.061 (0.390)	2.055 (0.377)	
- Range	1.379 - 2.374	1.141 - 2.681	1.141 - 2.681	
Tapetum L				0.973
- Mean (SD)	2.404 (0.471)	2.310 (0.410)	2.329 (0.419)	
- Range	1.674 - 3.197	1.407 - 2.936	1.407 - 3.197	

Fractional anisotropy (FA) (DTI) - Offspring of lean mothers (OLM) vs control subjects - (JHU white-matter tractography atlas) - Uncorrected p-values

	CTR (N=9)	OLM (N=19)	Total (N=28)	p value
Middle cerebellar peduncle				0.735
- Mean (SD)	0.406 (0.045)	0.413 (0.051)	0.411 (0.048)	
- Range	0.296 - 0.453	0.263 - 0.478	0.263 - 0.478	
Pontine crossing tract				0.099
- Mean (SD)	0.385 (0.034)	0.412 (0.041)	0.403 (0.040)	
- Range	0.324 - 0.428	0.349 - 0.483	0.324 - 0.483	
Genu of corpus callosum				0.858
- Mean (SD)	0.441 (0.041)	0.438 (0.029)	0.439 (0.033)	
- Range	0.371 - 0.486	0.383 - 0.498	0.371 - 0.498	
Body of corpus callosum				0.503
- Mean (SD)	0.501 (0.043)	0.490 (0.037)	0.494 (0.038)	
- Range	0.425 - 0.554	0.426 - 0.553	0.425 - 0.554	
Splenium of corpus callosum				0.814
- Mean (SD)	0.586 (0.052)	0.590 (0.038)	0.589 (0.042)	
- Range	0.494 - 0.669	0.533 - 0.669	0.494 - 0.669	
Fornix				0.468
- Mean (SD)	0.242 (0.037)	0.251 (0.028)	0.248 (0.031)	
- Range	0.193 - 0.281	0.197 - 0.303	0.193 - 0.303	
Corticospinal tract R				0.112
- Mean (SD)	0.444 (0.042)	0.470 (0.036)	0.462 (0.040)	
- Range	0.369 - 0.499	0.406 - 0.534	0.369 - 0.534	
Corticospinal tract L				0.036
- Mean (SD)	0.446 (0.044)	0.485 (0.044)	0.473 (0.047)	
- Range	0.370 - 0.482	0.420 - 0.598	0.370 - 0.598	
Medial lemniscus R				0.672
- Mean (SD)	0.445 (0.034)	0.453 (0.055)	0.450 (0.049)	
- Range	0.403 - 0.514	0.335 - 0.560	0.335 - 0.560	
Medial lemniscus L				0.999
- Mean (SD)	0.453 (0.036)	0.453 (0.056)	0.453 (0.049)	
- Range	0.404 - 0.506	0.360 - 0.598	0.360 - 0.598	
Inferior cerebellar peduncle R				0.498
- Mean (SD)	0.350 (0.048)	0.362 (0.045)	0.358 (0.045)	
- Range	0.282 - 0.442	0.272 - 0.437	0.272 - 0.442	
Inferior cerebellar peduncle L				0.497
- Mean (SD)	0.361 (0.032)	0.371 (0.038)	0.368 (0.036)	
- Range	0.328 - 0.422	0.303 - 0.432	0.303 - 0.432	
Superior cerebellar peduncle R				0.830
- Mean (SD)	0.471 (0.062)	0.477 (0.068)	0.475 (0.065)	
- Range	0.346 - 0.530	0.304 - 0.568	0.304 - 0.568	
Superior cerebellar peduncle L				0.712
- Mean (SD)	0.447 (0.057)	0.455 (0.049)	0.452 (0.051)	
- Range	0.309 - 0.487	0.329 - 0.521	0.309 - 0.521	
Cerebral peduncle R				0.383
- Mean (SD)	0.569 (0.026)	0.558 (0.033)	0.561 (0.031)	
- Range	0.513 - 0.598	0.495 - 0.625	0.495 - 0.625	
Cerebral peduncle L				0.974
- Mean (SD)	0.572 (0.036)	0.571 (0.033)	0.571 (0.033)	
- Range	0.485 - 0.610	0.491 - 0.615	0.485 - 0.615	
Anterior limb of internal capsule R				0.324
- Mean (SD)	0.444 (0.040)	0.429 (0.034)	0.434 (0.036)	
- Range	0.357 - 0.486	0.341 - 0.482	0.341 - 0.486	
Anterior limb of internal capsule L				0.833
- Mean (SD)	0.442 (0.038)	0.439 (0.033)	0.440 (0.034)	
- Range	0.355 - 0.481	0.389 - 0.491	0.355 - 0.491	
Posterior limb of internal capsule R				0.549
- Mean (SD)	0.533 (0.033)	0.526 (0.028)	0.529 (0.029)	
- Range	0.463 - 0.574	0.472 - 0.568	0.463 - 0.574	
Posterior limb of internal capsule L				0.674
- Mean (SD)	0.543 (0.042)	0.538 (0.027)	0.540 (0.032)	
- Range	0.453 - 0.591	0.478 - 0.591	0.453 - 0.591	

	CTR (N=9)	OLM (N=19)	Total (N=28)	p value
Retrolenticular part of internal capsule R				0.566
- Mean (SD)	0.442 (0.037)	0.434 (0.032)	0.436 (0.033)	
- Range	0.375 - 0.501	0.389 - 0.518	0.375 - 0.518	
Retrolenticular part of internal capsule L				0.276
- Mean (SD)	0.459 (0.031)	0.445 (0.030)	0.450 (0.031)	
- Range	0.405 - 0.496	0.402 - 0.522	0.402 - 0.522	
Anterior corona radiata R				0.546
- Mean (SD)	0.334 (0.029)	0.326 (0.033)	0.329 (0.032)	
- Range	0.273 - 0.364	0.263 - 0.383	0.263 - 0.383	
Anterior corona radiata L				0.730
- Mean (SD)	0.334 (0.027)	0.330 (0.031)	0.332 (0.030)	
- Range	0.280 - 0.358	0.270 - 0.382	0.270 - 0.382	
Superior corona radiata R				0.275
- Mean (SD)	0.416 (0.037)	0.400 (0.036)	0.405 (0.036)	
- Range	0.366 - 0.470	0.315 - 0.451	0.315 - 0.470	
Superior corona radiata L				0.470
- Mean (SD)	0.414 (0.031)	0.403 (0.039)	0.406 (0.036)	
- Range	0.356 - 0.452	0.311 - 0.478	0.311 - 0.478	
Posterior corona radiata R				0.139
- Mean (SD)	0.430 (0.047)	0.403 (0.042)	0.412 (0.044)	
- Range	0.373 - 0.513	0.331 - 0.480	0.331 - 0.513	
Posterior corona radiata L				0.356
- Mean (SD)	0.412 (0.034)	0.396 (0.045)	0.401 (0.042)	
- Range	0.368 - 0.466	0.308 - 0.491	0.308 - 0.491	
Posterior thalamic radiation R				0.070
- Mean (SD)	0.472 (0.034)	0.445 (0.036)	0.454 (0.037)	
- Range	0.415 - 0.543	0.372 - 0.491	0.372 - 0.543	
Posterior thalamic radiation L				0.700
- Mean (SD)	0.447 (0.044)	0.441 (0.032)	0.443 (0.036)	
- Range	0.358 - 0.494	0.388 - 0.504	0.358 - 0.504	
Sagittal stratum R				0.129
- Mean (SD)	0.447 (0.032)	0.428 (0.027)	0.434 (0.030)	
- Range	0.406 - 0.506	0.396 - 0.504	0.396 - 0.506	
Sagittal stratum L				0.334
- Mean (SD)	0.404 (0.031)	0.392 (0.028)	0.396 (0.029)	
- Range	0.349 - 0.444	0.346 - 0.464	0.346 - 0.464	
External capsule R				0.382
- Mean (SD)	0.336 (0.026)	0.325 (0.031)	0.329 (0.030)	
- Range	0.295 - 0.366	0.280 - 0.412	0.280 - 0.412	
External capsule L				0.544
- Mean (SD)	0.343 (0.021)	0.336 (0.030)	0.339 (0.027)	
- Range	0.295 - 0.371	0.302 - 0.399	0.295 - 0.399	
Cingulum cingulate gyrus R				0.481
- Mean (SD)	0.368 (0.040)	0.358 (0.029)	0.361 (0.032)	
- Range	0.293 - 0.425	0.303 - 0.414	0.293 - 0.425	
Cingulum cingulate gyrus L				0.419
- Mean (SD)	0.383 (0.041)	0.371 (0.033)	0.375 (0.035)	
- Range	0.329 - 0.430	0.310 - 0.445	0.310 - 0.445	
Cingulum hippocampus R				0.361
- Mean (SD)	0.297 (0.030)	0.283 (0.040)	0.288 (0.037)	
- Range	0.250 - 0.340	0.213 - 0.371	0.213 - 0.371	
Cingulum hippocampus L				0.495
- Mean (SD)	0.273 (0.032)	0.284 (0.039)	0.280 (0.036)	
- Range	0.226 - 0.332	0.204 - 0.366	0.204 - 0.366	
Fornix R				0.349
- Mean (SD)	0.375 (0.031)	0.363 (0.031)	0.367 (0.031)	
- Range	0.317 - 0.412	0.319 - 0.426	0.317 - 0.426	
Fornix L				0.813
- Mean (SD)	0.383 (0.027)	0.379 (0.035)	0.380 (0.032)	
- Range	0.331 - 0.432	0.330 - 0.438	0.330 - 0.438	
Superior longitudinal fasciculus R				0.509
- Mean (SD)	0.395 (0.035)	0.387 (0.029)	0.389 (0.031)	

	CTR (N=9)	OLM (N=19)	Total (N=28)	p value
- Range	0.344 - 0.442	0.346 - 0.426	0.344 - 0.442	
Superior longitudinal fasciculus L				0.473
- Mean (SD)	0.399 (0.040)	0.388 (0.033)	0.391 (0.035)	
- Range	0.327 - 0.463	0.320 - 0.431	0.320 - 0.463	
Superior fronto occipital fasciculus R				0.231
- Mean (SD)	0.359 (0.047)	0.336 (0.048)	0.343 (0.048)	
- Range	0.279 - 0.415	0.248 - 0.441	0.248 - 0.441	
Superior fronto occipital fasciculus L				0.963
- Mean (SD)	0.324 (0.053)	0.325 (0.042)	0.325 (0.045)	
- Range	0.234 - 0.389	0.243 - 0.383	0.234 - 0.389	
Uncinate fasciculus R				0.393
- Mean (SD)	0.381 (0.049)	0.395 (0.038)	0.390 (0.042)	
- Range	0.288 - 0.424	0.335 - 0.462	0.288 - 0.462	
Uncinate fasciculus L				0.999
- Mean (SD)	0.385 (0.036)	0.385 (0.038)	0.385 (0.037)	
- Range	0.333 - 0.434	0.319 - 0.439	0.319 - 0.439	
Tapetum R				0.581
- Mean (SD)	0.343 (0.037)	0.335 (0.034)	0.337 (0.035)	
- Range	0.268 - 0.386	0.264 - 0.383	0.264 - 0.386	
Tapetum L				0.223
- Mean (SD)	0.307 (0.018)	0.294 (0.029)	0.298 (0.026)	
- Range	0.278 - 0.332	0.228 - 0.335	0.228 - 0.335	

Fractional anisotropy (FA) (DTI) - Offspring of lean mothers (OLM) vs control subjects - (JHU white-matter tractography atlas) - FDR corrected p-values

	CTR (N=9)	OLM (N=19)	Total (N=28)	p value
Middle cerebellar peduncle				0.904
- Mean (SD)	0.406 (0.045)	0.413 (0.051)	0.411 (0.048)	
- Range	0.296 - 0.453	0.263 - 0.478	0.263 - 0.478	
Pontine crossing tract				0.845
- Mean (SD)	0.385 (0.034)	0.412 (0.041)	0.403 (0.040)	
- Range	0.324 - 0.428	0.349 - 0.483	0.324 - 0.483	
Genu of corpus callosum				0.936
- Mean (SD)	0.441 (0.041)	0.438 (0.029)	0.439 (0.033)	
- Range	0.371 - 0.486	0.383 - 0.498	0.371 - 0.498	
Body of corpus callosum				0.845
- Mean (SD)	0.501 (0.043)	0.490 (0.037)	0.494 (0.038)	
- Range	0.425 - 0.554	0.426 - 0.553	0.425 - 0.554	
Splenium of corpus callosum				0.930
- Mean (SD)	0.586 (0.052)	0.590 (0.038)	0.589 (0.042)	
- Range	0.494 - 0.669	0.533 - 0.669	0.494 - 0.669	
Fornix				0.845
- Mean (SD)	0.242 (0.037)	0.251 (0.028)	0.248 (0.031)	
- Range	0.193 - 0.281	0.197 - 0.303	0.193 - 0.303	
Corticospinal tract R				0.845
- Mean (SD)	0.444 (0.042)	0.470 (0.036)	0.462 (0.040)	
- Range	0.369 - 0.499	0.406 - 0.534	0.369 - 0.534	
Corticospinal tract L				0.845
- Mean (SD)	0.446 (0.044)	0.485 (0.044)	0.473 (0.047)	
- Range	0.370 - 0.482	0.420 - 0.598	0.370 - 0.598	
Medial lemniscus R				0.904
- Mean (SD)	0.445 (0.034)	0.453 (0.055)	0.450 (0.049)	
- Range	0.403 - 0.514	0.335 - 0.560	0.335 - 0.560	
Medial lemniscus L				0.999
- Mean (SD)	0.453 (0.036)	0.453 (0.056)	0.453 (0.049)	
- Range	0.404 - 0.506	0.360 - 0.598	0.360 - 0.598	
Inferior cerebellar peduncle R				0.845
- Mean (SD)	0.350 (0.048)	0.362 (0.045)	0.358 (0.045)	
- Range	0.282 - 0.442	0.272 - 0.437	0.272 - 0.442	
Inferior cerebellar peduncle L				0.845
- Mean (SD)	0.361 (0.032)	0.371 (0.038)	0.368 (0.036)	
- Range	0.328 - 0.422	0.303 - 0.432	0.303 - 0.432	
Superior cerebellar peduncle R				0.930
- Mean (SD)	0.471 (0.062)	0.477 (0.068)	0.475 (0.065)	
- Range	0.346 - 0.530	0.304 - 0.568	0.304 - 0.568	
Superior cerebellar peduncle L				0.904
- Mean (SD)	0.447 (0.057)	0.455 (0.049)	0.452 (0.051)	
- Range	0.309 - 0.487	0.329 - 0.521	0.309 - 0.521	
Cerebral peduncle R				0.845
- Mean (SD)	0.569 (0.026)	0.558 (0.033)	0.561 (0.031)	
- Range	0.513 - 0.598	0.495 - 0.625	0.495 - 0.625	
Cerebral peduncle L				0.999
- Mean (SD)	0.572 (0.036)	0.571 (0.033)	0.571 (0.033)	
- Range	0.485 - 0.610	0.491 - 0.615	0.485 - 0.615	
Anterior limb of internal capsule R				0.845
- Mean (SD)	0.444 (0.040)	0.429 (0.034)	0.434 (0.036)	
- Range	0.357 - 0.486	0.341 - 0.482	0.341 - 0.486	
Anterior limb of internal capsule L				0.930
- Mean (SD)	0.442 (0.038)	0.439 (0.033)	0.440 (0.034)	
- Range	0.355 - 0.481	0.389 - 0.491	0.355 - 0.491	
Posterior limb of internal capsule R				0.845
- Mean (SD)	0.533 (0.033)	0.526 (0.028)	0.529 (0.029)	
- Range	0.463 - 0.574	0.472 - 0.568	0.463 - 0.574	
Posterior limb of internal capsule L				0.904
- Mean (SD)	0.543 (0.042)	0.538 (0.027)	0.540 (0.032)	
- Range	0.453 - 0.591	0.478 - 0.591	0.453 - 0.591	

	CTR (N=9)	OLM (N=19)	Total (N=28)	p value
Retrolenticular part of internal capsule R				0.845
- Mean (SD)	0.442 (0.037)	0.434 (0.032)	0.436 (0.033)	
- Range	0.375 - 0.501	0.389 - 0.518	0.375 - 0.518	
Retrolenticular part of internal capsule L				0.845
- Mean (SD)	0.459 (0.031)	0.445 (0.030)	0.450 (0.031)	
- Range	0.405 - 0.496	0.402 - 0.522	0.402 - 0.522	
Anterior corona radiata R				0.845
- Mean (SD)	0.334 (0.029)	0.326 (0.033)	0.329 (0.032)	
- Range	0.273 - 0.364	0.263 - 0.383	0.263 - 0.383	
Anterior corona radiata L				0.904
- Mean (SD)	0.334 (0.027)	0.330 (0.031)	0.332 (0.030)	
- Range	0.280 - 0.358	0.270 - 0.382	0.270 - 0.382	
Superior corona radiata R				0.845
- Mean (SD)	0.416 (0.037)	0.400 (0.036)	0.405 (0.036)	
- Range	0.366 - 0.470	0.315 - 0.451	0.315 - 0.470	
Superior corona radiata L				0.845
- Mean (SD)	0.414 (0.031)	0.403 (0.039)	0.406 (0.036)	
- Range	0.356 - 0.452	0.311 - 0.478	0.311 - 0.478	
Posterior corona radiata R				0.845
- Mean (SD)	0.430 (0.047)	0.403 (0.042)	0.412 (0.044)	
- Range	0.373 - 0.513	0.331 - 0.480	0.331 - 0.513	
Posterior corona radiata L				0.845
- Mean (SD)	0.412 (0.034)	0.396 (0.045)	0.401 (0.042)	
- Range	0.368 - 0.466	0.308 - 0.491	0.308 - 0.491	
Posterior thalamic radiation R				0.845
- Mean (SD)	0.472 (0.034)	0.445 (0.036)	0.454 (0.037)	
- Range	0.415 - 0.543	0.372 - 0.491	0.372 - 0.543	
Posterior thalamic radiation L				0.904
- Mean (SD)	0.447 (0.044)	0.441 (0.032)	0.443 (0.036)	
- Range	0.358 - 0.494	0.388 - 0.504	0.358 - 0.504	
Sagittal stratum R				0.845
- Mean (SD)	0.447 (0.032)	0.428 (0.027)	0.434 (0.030)	
- Range	0.406 - 0.506	0.396 - 0.504	0.396 - 0.506	
Sagittal stratum L				0.845
- Mean (SD)	0.404 (0.031)	0.392 (0.028)	0.396 (0.029)	
- Range	0.349 - 0.444	0.346 - 0.464	0.346 - 0.464	
External capsule R				0.845
- Mean (SD)	0.336 (0.026)	0.325 (0.031)	0.329 (0.030)	
- Range	0.295 - 0.366	0.280 - 0.412	0.280 - 0.412	
External capsule L				0.845
- Mean (SD)	0.343 (0.021)	0.336 (0.030)	0.339 (0.027)	
- Range	0.295 - 0.371	0.302 - 0.399	0.295 - 0.399	
Cingulum cingulate gyrus R				0.845
- Mean (SD)	0.368 (0.040)	0.358 (0.029)	0.361 (0.032)	
- Range	0.293 - 0.425	0.303 - 0.414	0.293 - 0.425	
Cingulum cingulate gyrus L				0.845
- Mean (SD)	0.383 (0.041)	0.371 (0.033)	0.375 (0.035)	
- Range	0.329 - 0.430	0.310 - 0.445	0.310 - 0.445	
Cingulum hippocampus R				0.845
- Mean (SD)	0.297 (0.030)	0.283 (0.040)	0.288 (0.037)	
- Range	0.250 - 0.340	0.213 - 0.371	0.213 - 0.371	
Cingulum hippocampus L				0.845
- Mean (SD)	0.273 (0.032)	0.284 (0.039)	0.280 (0.036)	
- Range	0.226 - 0.332	0.204 - 0.366	0.204 - 0.366	
Fornix R				0.845
- Mean (SD)	0.375 (0.031)	0.363 (0.031)	0.367 (0.031)	
- Range	0.317 - 0.412	0.319 - 0.426	0.317 - 0.426	
Fornix L				0.930
- Mean (SD)	0.383 (0.027)	0.379 (0.035)	0.380 (0.032)	
- Range	0.331 - 0.432	0.330 - 0.438	0.330 - 0.438	
Superior longitudinal fasciculus R				0.845
- Mean (SD)	0.395 (0.035)	0.387 (0.029)	0.389 (0.031)	

	CTR (N=9)	OLM (N=19)	Total (N=28)	p value
- Range	0.344 - 0.442	0.346 - 0.426	0.344 - 0.442	
Superior longitudinal fasciculus L				0.845
- Mean (SD)	0.399 (0.040)	0.388 (0.033)	0.391 (0.035)	
- Range	0.327 - 0.463	0.320 - 0.431	0.320 - 0.463	
Superior fronto occipital fasciculus R				0.845
- Mean (SD)	0.359 (0.047)	0.336 (0.048)	0.343 (0.048)	
- Range	0.279 - 0.415	0.248 - 0.441	0.248 - 0.441	
Superior fronto occipital fasciculus L				0.999
- Mean (SD)	0.324 (0.053)	0.325 (0.042)	0.325 (0.045)	
- Range	0.234 - 0.389	0.243 - 0.383	0.234 - 0.389	
Uncinate fasciculus R				0.845
- Mean (SD)	0.381 (0.049)	0.395 (0.038)	0.390 (0.042)	
- Range	0.288 - 0.424	0.335 - 0.462	0.288 - 0.462	
Uncinate fasciculus L				0.999
- Mean (SD)	0.385 (0.036)	0.385 (0.038)	0.385 (0.037)	
- Range	0.333 - 0.434	0.319 - 0.439	0.319 - 0.439	
Tapetum R				0.845
- Mean (SD)	0.343 (0.037)	0.335 (0.034)	0.337 (0.035)	
- Range	0.268 - 0.386	0.264 - 0.383	0.264 - 0.386	
Tapetum L				0.845
- Mean (SD)	0.307 (0.018)	0.294 (0.029)	0.298 (0.026)	
- Range	0.278 - 0.332	0.228 - 0.335	0.228 - 0.335	

Mean diffusivity (MD, (10^-3)*(mm^2)/s) (DTI) - Offspring of lean mothers (OLM) vs control subjects - (JHU white-matter tractography atlas) - Uncorrected p-values

	CTR (N=9)	OLM (N=19)	Total (N=28)	p value
Middle cerebellar peduncle				0.340
- Mean (SD)	1.089 (0.156)	1.043 (0.096)	1.058 (0.118)	
- Range	0.906 - 1.447	0.857 - 1.268	0.857 - 1.447	
Pontine crossing tract				0.982
- Mean (SD)	0.797 (0.070)	0.797 (0.074)	0.797 (0.071)	
- Range	0.723 - 0.951	0.683 - 0.974	0.683 - 0.974	
Genu of corpus callosum				0.891
- Mean (SD)	1.370 (0.119)	1.362 (0.158)	1.365 (0.145)	
- Range	1.240 - 1.614	1.042 - 1.688	1.042 - 1.688	
Body of corpus callosum				0.920
- Mean (SD)	1.112 (0.116)	1.108 (0.089)	1.109 (0.096)	
- Range	0.990 - 1.319	0.984 - 1.285	0.984 - 1.319	
Splenium of corpus callosum				0.451
- Mean (SD)	1.046 (0.151)	1.011 (0.090)	1.022 (0.112)	
- Range	0.888 - 1.318	0.894 - 1.221	0.888 - 1.318	
Fornix				0.311
- Mean (SD)	2.495 (0.323)	2.388 (0.222)	2.422 (0.258)	
- Range	2.048 - 2.977	1.956 - 2.841	1.956 - 2.977	
Corticospinal tract R				0.734
- Mean (SD)	0.865 (0.094)	0.853 (0.076)	0.857 (0.080)	
- Range	0.754 - 1.012	0.724 - 1.059	0.724 - 1.059	
Corticospinal tract L				0.748
- Mean (SD)	0.824 (0.108)	0.835 (0.079)	0.832 (0.088)	
- Range	0.736 - 1.037	0.723 - 0.968	0.723 - 1.037	
Medial lemniscus R				0.509
- Mean (SD)	0.846 (0.068)	0.874 (0.113)	0.865 (0.100)	
- Range	0.782 - 0.982	0.697 - 1.165	0.697 - 1.165	
Medial lemniscus L				0.601
- Mean (SD)	0.834 (0.046)	0.847 (0.064)	0.843 (0.058)	
- Range	0.783 - 0.935	0.695 - 0.981	0.695 - 0.981	
Inferior cerebellar peduncle R				0.485
- Mean (SD)	1.136 (0.189)	1.076 (0.217)	1.096 (0.207)	
- Range	0.880 - 1.524	0.831 - 1.875	0.831 - 1.875	
Inferior cerebellar peduncle L				0.620
- Mean (SD)	1.057 (0.148)	1.030 (0.127)	1.039 (0.132)	
- Range	0.954 - 1.406	0.864 - 1.363	0.864 - 1.406	
Superior cerebellar peduncle R				0.830
- Mean (SD)	1.209 (0.241)	1.225 (0.158)	1.220 (0.184)	
- Range	1.000 - 1.829	0.890 - 1.472	0.890 - 1.829	
Superior cerebellar peduncle L				0.777
- Mean (SD)	1.265 (0.175)	1.286 (0.177)	1.279 (0.174)	
- Range	1.010 - 1.622	0.968 - 1.618	0.968 - 1.622	
Cerebral peduncle R				0.927
- Mean (SD)	0.874 (0.071)	0.877 (0.048)	0.876 (0.055)	
- Range	0.794 - 1.038	0.785 - 0.951	0.785 - 1.038	
Cerebral peduncle L				0.981
- Mean (SD)	0.842 (0.060)	0.841 (0.050)	0.842 (0.052)	
- Range	0.788 - 0.985	0.733 - 0.926	0.733 - 0.985	
Anterior limb of internal capsule R				0.569
- Mean (SD)	0.846 (0.087)	0.864 (0.069)	0.858 (0.074)	
- Range	0.773 - 1.055	0.774 - 1.020	0.773 - 1.055	
Anterior limb of internal capsule L				0.975
- Mean (SD)	0.877 (0.110)	0.875 (0.077)	0.876 (0.087)	
- Range	0.775 - 1.112	0.772 - 1.040	0.772 - 1.112	
Posterior limb of internal capsule R				0.931
- Mean (SD)	0.770 (0.050)	0.768 (0.041)	0.768 (0.043)	
- Range	0.689 - 0.846	0.705 - 0.857	0.689 - 0.857	
Posterior limb of internal capsule L				0.787
- Mean (SD)	0.770 (0.060)	0.764 (0.038)	0.766 (0.045)	
- Range	0.694 - 0.882	0.703 - 0.847	0.694 - 0.882	

	CTR (N=9)	OLM (N=19)	Total (N=28)	p value
Retrolenticular part of internal capsule R				0.325
- Mean (SD)	0.934 (0.097)	0.904 (0.058)	0.914 (0.072)	
- Range	0.827 - 1.105	0.793 - 1.009	0.793 - 1.105	
Retrolenticular part of internal capsule L				0.833
- Mean (SD)	0.936 (0.111)	0.929 (0.065)	0.931 (0.081)	
- Range	0.837 - 1.171	0.822 - 1.088	0.822 - 1.171	
Anterior corona radiata R				0.476
- Mean (SD)	0.940 (0.079)	0.968 (0.102)	0.959 (0.094)	
- Range	0.835 - 1.070	0.790 - 1.160	0.790 - 1.160	
Anterior corona radiata L				0.392
- Mean (SD)	0.927 (0.066)	0.966 (0.125)	0.953 (0.110)	
- Range	0.841 - 1.030	0.791 - 1.298	0.791 - 1.298	
Superior corona radiata R				0.780
- Mean (SD)	0.821 (0.059)	0.830 (0.083)	0.827 (0.075)	
- Range	0.740 - 0.927	0.708 - 1.096	0.708 - 1.096	
Superior corona radiata L				0.739
- Mean (SD)	0.835 (0.068)	0.845 (0.078)	0.842 (0.074)	
- Range	0.743 - 0.949	0.705 - 1.052	0.705 - 1.052	
Posterior corona radiata R				0.985
- Mean (SD)	0.967 (0.131)	0.966 (0.076)	0.966 (0.095)	
- Range	0.818 - 1.163	0.852 - 1.178	0.818 - 1.178	
Posterior corona radiata L				0.616
- Mean (SD)	1.028 (0.189)	1.001 (0.098)	1.010 (0.131)	
- Range	0.871 - 1.331	0.813 - 1.192	0.813 - 1.331	
Posterior thalamic radiation R				0.567
- Mean (SD)	1.002 (0.097)	1.024 (0.096)	1.017 (0.095)	
- Range	0.857 - 1.174	0.874 - 1.248	0.857 - 1.248	
Posterior thalamic radiation L				0.902
- Mean (SD)	1.137 (0.115)	1.144 (0.167)	1.142 (0.150)	
- Range	0.993 - 1.352	0.934 - 1.606	0.934 - 1.606	
Sagittal stratum R				0.912
- Mean (SD)	0.986 (0.089)	0.990 (0.065)	0.989 (0.072)	
- Range	0.833 - 1.078	0.880 - 1.091	0.833 - 1.091	
Sagittal stratum L				0.931
- Mean (SD)	1.074 (0.143)	1.078 (0.118)	1.077 (0.124)	
- Range	0.903 - 1.379	0.881 - 1.305	0.881 - 1.379	
External capsule R				0.313
- Mean (SD)	0.842 (0.067)	0.868 (0.060)	0.860 (0.062)	
- Range	0.764 - 0.953	0.784 - 0.996	0.764 - 0.996	
External capsule L				0.420
- Mean (SD)	0.822 (0.048)	0.841 (0.060)	0.835 (0.056)	
- Range	0.766 - 0.893	0.765 - 0.953	0.765 - 0.953	
Cingulum cingulate gyrus R				0.193
- Mean (SD)	0.950 (0.134)	0.898 (0.074)	0.914 (0.098)	
- Range	0.802 - 1.199	0.782 - 1.082	0.782 - 1.199	
Cingulum cingulate gyrus L				0.887
- Mean (SD)	0.945 (0.100)	0.939 (0.086)	0.941 (0.089)	
- Range	0.835 - 1.085	0.792 - 1.115	0.792 - 1.115	
Cingulum hippocampus R				0.143
- Mean (SD)	1.106 (0.143)	1.038 (0.092)	1.060 (0.113)	
- Range	0.917 - 1.303	0.887 - 1.212	0.887 - 1.303	
Cingulum hippocampus L				0.002
- Mean (SD)	1.256 (0.183)	1.061 (0.110)	1.124 (0.163)	
- Range	1.007 - 1.484	0.849 - 1.236	0.849 - 1.484	
Fornix R				0.575
- Mean (SD)	1.055 (0.138)	1.083 (0.114)	1.074 (0.121)	
- Range	0.866 - 1.261	0.844 - 1.309	0.844 - 1.309	
Fornix L				0.641
- Mean (SD)	1.015 (0.136)	1.037 (0.108)	1.030 (0.115)	
- Range	0.851 - 1.246	0.841 - 1.278	0.841 - 1.278	
Superior longitudinal fasciculus R				0.925
- Mean (SD)	0.805 (0.052)	0.803 (0.053)	0.803 (0.052)	

	CTR (N=9)	OLM (N=19)	Total (N=28)	p value
- Range	0.758 - 0.898	0.740 - 0.950	0.740 - 0.950	
Superior longitudinal fasciculus L				0.439
- Mean (SD)	0.781 (0.038)	0.796 (0.049)	0.791 (0.046)	
- Range	0.732 - 0.839	0.730 - 0.904	0.730 - 0.904	
Superior fronto occipital fasciculus R				0.967
- Mean (SD)	0.964 (0.172)	0.967 (0.198)	0.966 (0.187)	
- Range	0.718 - 1.300	0.723 - 1.560	0.718 - 1.560	
Superior fronto occipital fasciculus L				0.883
- Mean (SD)	1.088 (0.246)	1.102 (0.236)	1.098 (0.235)	
- Range	0.836 - 1.504	0.819 - 1.744	0.819 - 1.744	
Uncinate fasciculus R				0.727
- Mean (SD)	0.913 (0.179)	0.895 (0.093)	0.900 (0.124)	
- Range	0.782 - 1.375	0.797 - 1.189	0.782 - 1.375	
Uncinate fasciculus L				0.362
- Mean (SD)	0.846 (0.064)	0.885 (0.119)	0.872 (0.104)	
- Range	0.777 - 0.939	0.780 - 1.287	0.777 - 1.287	
Tapetum R				0.382
- Mean (SD)	2.032 (0.341)	2.157 (0.352)	2.117 (0.347)	
- Range	1.379 - 2.374	1.528 - 2.681	1.379 - 2.681	
Tapetum L				0.967
- Mean (SD)	2.404 (0.471)	2.411 (0.370)	2.408 (0.396)	
- Range	1.674 - 3.197	1.678 - 2.936	1.674 - 3.197	

Mean diffusivity (MD, (10^-3)*(mm^2)/s) (DTI) - Offspring of lean mothers (OLM) vs control subjects - (JHU white-matter tractography atlas) - FDR corrected p-values

	CTR (N=9)	OLM (N=19)	Total (N=28)	p value
Middle cerebellar peduncle				0.985
- Mean (SD)	1.089 (0.156)	1.043 (0.096)	1.058 (0.118)	
- Range	0.906 - 1.447	0.857 - 1.268	0.857 - 1.447	
Pontine crossing tract				0.985
- Mean (SD)	0.797 (0.070)	0.797 (0.074)	0.797 (0.071)	
- Range	0.723 - 0.951	0.683 - 0.974	0.683 - 0.974	
Genu of corpus callosum				0.985
- Mean (SD)	1.370 (0.119)	1.362 (0.158)	1.365 (0.145)	
- Range	1.240 - 1.614	1.042 - 1.688	1.042 - 1.688	
Body of corpus callosum				0.985
- Mean (SD)	1.112 (0.116)	1.108 (0.089)	1.109 (0.096)	
- Range	0.990 - 1.319	0.984 - 1.285	0.984 - 1.319	
Splenium of corpus callosum				0.985
- Mean (SD)	1.046 (0.151)	1.011 (0.090)	1.022 (0.112)	
- Range	0.888 - 1.318	0.894 - 1.221	0.888 - 1.318	
Fornix				0.985
- Mean (SD)	2.495 (0.323)	2.388 (0.222)	2.422 (0.258)	
- Range	2.048 - 2.977	1.956 - 2.841	1.956 - 2.977	
Corticospinal tract R				0.985
- Mean (SD)	0.865 (0.094)	0.853 (0.076)	0.857 (0.080)	
- Range	0.754 - 1.012	0.724 - 1.059	0.724 - 1.059	
Corticospinal tract L				0.985
- Mean (SD)	0.824 (0.108)	0.835 (0.079)	0.832 (0.088)	
- Range	0.736 - 1.037	0.723 - 0.968	0.723 - 1.037	
Medial lemniscus R				0.985
- Mean (SD)	0.846 (0.068)	0.874 (0.113)	0.865 (0.100)	
- Range	0.782 - 0.982	0.697 - 1.165	0.697 - 1.165	
Medial lemniscus L				0.985
- Mean (SD)	0.834 (0.046)	0.847 (0.064)	0.843 (0.058)	
- Range	0.783 - 0.935	0.695 - 0.981	0.695 - 0.981	
Inferior cerebellar peduncle R				0.985
- Mean (SD)	1.136 (0.189)	1.076 (0.217)	1.096 (0.207)	
- Range	0.880 - 1.524	0.831 - 1.875	0.831 - 1.875	
Inferior cerebellar peduncle L				0.985
- Mean (SD)	1.057 (0.148)	1.030 (0.127)	1.039 (0.132)	
- Range	0.954 - 1.406	0.864 - 1.363	0.864 - 1.406	
Superior cerebellar peduncle R				0.985
- Mean (SD)	1.209 (0.241)	1.225 (0.158)	1.220 (0.184)	
- Range	1.000 - 1.829	0.890 - 1.472	0.890 - 1.829	
Superior cerebellar peduncle L				0.985
- Mean (SD)	1.265 (0.175)	1.286 (0.177)	1.279 (0.174)	
- Range	1.010 - 1.622	0.968 - 1.618	0.968 - 1.622	
Cerebral peduncle R				0.985
- Mean (SD)	0.874 (0.071)	0.877 (0.048)	0.876 (0.055)	
- Range	0.794 - 1.038	0.785 - 0.951	0.785 - 1.038	
Cerebral peduncle L				0.985
- Mean (SD)	0.842 (0.060)	0.841 (0.050)	0.842 (0.052)	
- Range	0.788 - 0.985	0.733 - 0.926	0.733 - 0.985	
Anterior limb of internal capsule R				0.985
- Mean (SD)	0.846 (0.087)	0.864 (0.069)	0.858 (0.074)	
- Range	0.773 - 1.055	0.774 - 1.020	0.773 - 1.055	
Anterior limb of internal capsule L				0.985
- Mean (SD)	0.877 (0.110)	0.875 (0.077)	0.876 (0.087)	
- Range	0.775 - 1.112	0.772 - 1.040	0.772 - 1.112	
Posterior limb of internal capsule R				0.985
- Mean (SD)	0.770 (0.050)	0.768 (0.041)	0.768 (0.043)	
- Range	0.689 - 0.846	0.705 - 0.857	0.689 - 0.857	
Posterior limb of internal capsule L				0.985
- Mean (SD)	0.770 (0.060)	0.764 (0.038)	0.766 (0.045)	

	CTR (N=9)	OLM (N=19)	Total (N=28)	p value
- Range	0.694 - 0.882	0.703 - 0.847	0.694 - 0.882	
Retrolenticular part of internal capsule R				0.985
- Mean (SD)	0.934 (0.097)	0.904 (0.058)	0.914 (0.072)	
- Range	0.827 - 1.105	0.793 - 1.009	0.793 - 1.105	
Retrolenticular part of internal capsule L				0.985
- Mean (SD)	0.936 (0.111)	0.929 (0.065)	0.931 (0.081)	
- Range	0.837 - 1.171	0.822 - 1.088	0.822 - 1.171	
Anterior corona radiata R				0.985
- Mean (SD)	0.940 (0.079)	0.968 (0.102)	0.959 (0.094)	
- Range	0.835 - 1.070	0.790 - 1.160	0.790 - 1.160	
Anterior corona radiata L				0.985
- Mean (SD)	0.927 (0.066)	0.966 (0.125)	0.953 (0.110)	
- Range	0.841 - 1.030	0.791 - 1.298	0.791 - 1.298	
Superior corona radiata R				0.985
- Mean (SD)	0.821 (0.059)	0.830 (0.083)	0.827 (0.075)	
- Range	0.740 - 0.927	0.708 - 1.096	0.708 - 1.096	
Superior corona radiata L				0.985
- Mean (SD)	0.835 (0.068)	0.845 (0.078)	0.842 (0.074)	
- Range	0.743 - 0.949	0.705 - 1.052	0.705 - 1.052	
Posterior corona radiata R				0.985
- Mean (SD)	0.967 (0.131)	0.966 (0.076)	0.966 (0.095)	
- Range	0.818 - 1.163	0.852 - 1.178	0.818 - 1.178	
Posterior corona radiata L				0.985
- Mean (SD)	1.028 (0.189)	1.001 (0.098)	1.010 (0.131)	
- Range	0.871 - 1.331	0.813 - 1.192	0.813 - 1.331	
Posterior thalamic radiation R				0.985
- Mean (SD)	1.002 (0.097)	1.024 (0.096)	1.017 (0.095)	
- Range	0.857 - 1.174	0.874 - 1.248	0.857 - 1.248	
Posterior thalamic radiation L				0.985
- Mean (SD)	1.137 (0.115)	1.144 (0.167)	1.142 (0.150)	
- Range	0.993 - 1.352	0.934 - 1.606	0.934 - 1.606	
Sagittal stratum R				0.985
- Mean (SD)	0.986 (0.089)	0.990 (0.065)	0.989 (0.072)	
- Range	0.833 - 1.078	0.880 - 1.091	0.833 - 1.091	
Sagittal stratum L				0.985
- Mean (SD)	1.074 (0.143)	1.078 (0.118)	1.077 (0.124)	
- Range	0.903 - 1.379	0.881 - 1.305	0.881 - 1.379	
External capsule R				0.985
- Mean (SD)	0.842 (0.067)	0.868 (0.060)	0.860 (0.062)	
- Range	0.764 - 0.953	0.784 - 0.996	0.764 - 0.996	
External capsule L				0.985
- Mean (SD)	0.822 (0.048)	0.841 (0.060)	0.835 (0.056)	
- Range	0.766 - 0.893	0.765 - 0.953	0.765 - 0.953	
Cingulum cingulate gyrus R				0.985
- Mean (SD)	0.950 (0.134)	0.898 (0.074)	0.914 (0.098)	
- Range	0.802 - 1.199	0.782 - 1.082	0.782 - 1.199	
Cingulum cingulate gyrus L				0.985
- Mean (SD)	0.945 (0.100)	0.939 (0.086)	0.941 (0.089)	
- Range	0.835 - 1.085	0.792 - 1.115	0.792 - 1.115	
Cingulum hippocampus R				0.985
- Mean (SD)	1.106 (0.143)	1.038 (0.092)	1.060 (0.113)	
- Range	0.917 - 1.303	0.887 - 1.212	0.887 - 1.303	
Cingulum hippocampus L				0.077
- Mean (SD)	1.256 (0.183)	1.061 (0.110)	1.124 (0.163)	
- Range	1.007 - 1.484	0.849 - 1.236	0.849 - 1.484	
Fornix R				0.985
- Mean (SD)	1.055 (0.138)	1.083 (0.114)	1.074 (0.121)	
- Range	0.866 - 1.261	0.844 - 1.309	0.844 - 1.309	
Fornix L				0.985
- Mean (SD)	1.015 (0.136)	1.037 (0.108)	1.030 (0.115)	
- Range	0.851 - 1.246	0.841 - 1.278	0.841 - 1.278	
Superior longitudinal fasciculus R				0.985

	CTR (N=9)	OLM (N=19)	Total (N=28)	p value
- Mean (SD)	0.805 (0.052)	0.803 (0.053)	0.803 (0.052)	
- Range	0.758 - 0.898	0.740 - 0.950	0.740 - 0.950	
Superior longitudinal fasciculus L				0.985
- Mean (SD)	0.781 (0.038)	0.796 (0.049)	0.791 (0.046)	
- Range	0.732 - 0.839	0.730 - 0.904	0.730 - 0.904	
Superior fronto occipital fasciculus R				0.985
- Mean (SD)	0.964 (0.172)	0.967 (0.198)	0.966 (0.187)	
- Range	0.718 - 1.300	0.723 - 1.560	0.718 - 1.560	
Superior fronto occipital fasciculus L				0.985
- Mean (SD)	1.088 (0.246)	1.102 (0.236)	1.098 (0.235)	
- Range	0.836 - 1.504	0.819 - 1.744	0.819 - 1.744	
Uncinate fasciculus R				0.985
- Mean (SD)	0.913 (0.179)	0.895 (0.093)	0.900 (0.124)	
- Range	0.782 - 1.375	0.797 - 1.189	0.782 - 1.375	
Uncinate fasciculus L				0.985
- Mean (SD)	0.846 (0.064)	0.885 (0.119)	0.872 (0.104)	
- Range	0.777 - 0.939	0.780 - 1.287	0.777 - 1.287	
Tapetum R				0.985
- Mean (SD)	2.032 (0.341)	2.157 (0.352)	2.117 (0.347)	
- Range	1.379 - 2.374	1.528 - 2.681	1.379 - 2.681	
Tapetum L				0.985
- Mean (SD)	2.404 (0.471)	2.411 (0.370)	2.408 (0.396)	
- Range	1.674 - 3.197	1.678 - 2.936	1.674 - 3.197	

Radial diffusivity (RD, (10^-3)*(mm^2)/s) (DTI) - Offspring of lean mothers (OLM) vs control subjects - (JHU white-matter tractography atlas) - Uncorrected p-values

	CTR (N=9)	OLM (N=19)	Total (N=28)	p value
Middle cerebellar peduncle				0.340
- Mean (SD)	1.089 (0.156)	1.043 (0.096)	1.058 (0.118)	
- Range	0.906 - 1.447	0.857 - 1.268	0.857 - 1.447	
Pontine crossing tract				0.982
- Mean (SD)	0.797 (0.070)	0.797 (0.074)	0.797 (0.071)	
- Range	0.723 - 0.951	0.683 - 0.974	0.683 - 0.974	
Genu of corpus callosum				0.891
- Mean (SD)	1.370 (0.119)	1.362 (0.158)	1.365 (0.145)	
- Range	1.240 - 1.614	1.042 - 1.688	1.042 - 1.688	
Body of corpus callosum				0.920
- Mean (SD)	1.112 (0.116)	1.108 (0.089)	1.109 (0.096)	
- Range	0.990 - 1.319	0.984 - 1.285	0.984 - 1.319	
Splenium of corpus callosum				0.451
- Mean (SD)	1.046 (0.151)	1.011 (0.090)	1.022 (0.112)	
- Range	0.888 - 1.318	0.894 - 1.221	0.888 - 1.318	
Fornix				0.311
- Mean (SD)	2.495 (0.323)	2.388 (0.222)	2.422 (0.258)	
- Range	2.048 - 2.977	1.956 - 2.841	1.956 - 2.977	
Corticospinal tract R				0.734
- Mean (SD)	0.865 (0.094)	0.853 (0.076)	0.857 (0.080)	
- Range	0.754 - 1.012	0.724 - 1.059	0.724 - 1.059	
Corticospinal tract L				0.748
- Mean (SD)	0.824 (0.108)	0.835 (0.079)	0.832 (0.088)	
- Range	0.736 - 1.037	0.723 - 0.968	0.723 - 1.037	
Medial lemniscus R				0.509
- Mean (SD)	0.846 (0.068)	0.874 (0.113)	0.865 (0.100)	
- Range	0.782 - 0.982	0.697 - 1.165	0.697 - 1.165	
Medial lemniscus L				0.601
- Mean (SD)	0.834 (0.046)	0.847 (0.064)	0.843 (0.058)	
- Range	0.783 - 0.935	0.695 - 0.981	0.695 - 0.981	
Inferior cerebellar peduncle R				0.485
- Mean (SD)	1.136 (0.189)	1.076 (0.217)	1.096 (0.207)	
- Range	0.880 - 1.524	0.831 - 1.875	0.831 - 1.875	
Inferior cerebellar peduncle L				0.620
- Mean (SD)	1.057 (0.148)	1.030 (0.127)	1.039 (0.132)	
- Range	0.954 - 1.406	0.864 - 1.363	0.864 - 1.406	
Superior cerebellar peduncle R				0.830
- Mean (SD)	1.209 (0.241)	1.225 (0.158)	1.220 (0.184)	
- Range	1.000 - 1.829	0.890 - 1.472	0.890 - 1.829	
Superior cerebellar peduncle L				0.777
- Mean (SD)	1.265 (0.175)	1.286 (0.177)	1.279 (0.174)	
- Range	1.010 - 1.622	0.968 - 1.618	0.968 - 1.622	
Cerebral peduncle R				0.927
- Mean (SD)	0.874 (0.071)	0.877 (0.048)	0.876 (0.055)	
- Range	0.794 - 1.038	0.785 - 0.951	0.785 - 1.038	
Cerebral peduncle L				0.981
- Mean (SD)	0.842 (0.060)	0.841 (0.050)	0.842 (0.052)	
- Range	0.788 - 0.985	0.733 - 0.926	0.733 - 0.985	
Anterior limb of internal capsule R				0.569
- Mean (SD)	0.846 (0.087)	0.864 (0.069)	0.858 (0.074)	
- Range	0.773 - 1.055	0.774 - 1.020	0.773 - 1.055	
Anterior limb of internal capsule L				0.975
- Mean (SD)	0.877 (0.110)	0.875 (0.077)	0.876 (0.087)	
- Range	0.775 - 1.112	0.772 - 1.040	0.772 - 1.112	
Posterior limb of internal capsule R				0.931
- Mean (SD)	0.770 (0.050)	0.768 (0.041)	0.768 (0.043)	
- Range	0.689 - 0.846	0.705 - 0.857	0.689 - 0.857	
Posterior limb of internal capsule L				0.787
- Mean (SD)	0.770 (0.060)	0.764 (0.038)	0.766 (0.045)	
- Range	0.694 - 0.882	0.703 - 0.847	0.694 - 0.882	

	CTR (N=9)	OLM (N=19)	Total (N=28)	p value
Retrolenticular part of internal capsule R				0.325
- Mean (SD)	0.934 (0.097)	0.904 (0.058)	0.914 (0.072)	
- Range	0.827 - 1.105	0.793 - 1.009	0.793 - 1.105	
Retrolenticular part of internal capsule L				0.833
- Mean (SD)	0.936 (0.111)	0.929 (0.065)	0.931 (0.081)	
- Range	0.837 - 1.171	0.822 - 1.088	0.822 - 1.171	
Anterior corona radiata R				0.476
- Mean (SD)	0.940 (0.079)	0.968 (0.102)	0.959 (0.094)	
- Range	0.835 - 1.070	0.790 - 1.160	0.790 - 1.160	
Anterior corona radiata L				0.392
- Mean (SD)	0.927 (0.066)	0.966 (0.125)	0.953 (0.110)	
- Range	0.841 - 1.030	0.791 - 1.298	0.791 - 1.298	
Superior corona radiata R				0.780
- Mean (SD)	0.821 (0.059)	0.830 (0.083)	0.827 (0.075)	
- Range	0.740 - 0.927	0.708 - 1.096	0.708 - 1.096	
Superior corona radiata L				0.739
- Mean (SD)	0.835 (0.068)	0.845 (0.078)	0.842 (0.074)	
- Range	0.743 - 0.949	0.705 - 1.052	0.705 - 1.052	
Posterior corona radiata R				0.985
- Mean (SD)	0.967 (0.131)	0.966 (0.076)	0.966 (0.095)	
- Range	0.818 - 1.163	0.852 - 1.178	0.818 - 1.178	
Posterior corona radiata L				0.616
- Mean (SD)	1.028 (0.189)	1.001 (0.098)	1.010 (0.131)	
- Range	0.871 - 1.331	0.813 - 1.192	0.813 - 1.331	
Posterior thalamic radiation R				0.567
- Mean (SD)	1.002 (0.097)	1.024 (0.096)	1.017 (0.095)	
- Range	0.857 - 1.174	0.874 - 1.248	0.857 - 1.248	
Posterior thalamic radiation L				0.902
- Mean (SD)	1.137 (0.115)	1.144 (0.167)	1.142 (0.150)	
- Range	0.993 - 1.352	0.934 - 1.606	0.934 - 1.606	
Sagittal stratum R				0.912
- Mean (SD)	0.986 (0.089)	0.990 (0.065)	0.989 (0.072)	
- Range	0.833 - 1.078	0.880 - 1.091	0.833 - 1.091	
Sagittal stratum L				0.931
- Mean (SD)	1.074 (0.143)	1.078 (0.118)	1.077 (0.124)	
- Range	0.903 - 1.379	0.881 - 1.305	0.881 - 1.379	
External capsule R				0.313
- Mean (SD)	0.842 (0.067)	0.868 (0.060)	0.860 (0.062)	
- Range	0.764 - 0.953	0.784 - 0.996	0.764 - 0.996	
External capsule L				0.420
- Mean (SD)	0.822 (0.048)	0.841 (0.060)	0.835 (0.056)	
- Range	0.766 - 0.893	0.765 - 0.953	0.765 - 0.953	
Cingulum cingulate gyrus R				0.193
- Mean (SD)	0.950 (0.134)	0.898 (0.074)	0.914 (0.098)	
- Range	0.802 - 1.199	0.782 - 1.082	0.782 - 1.199	
Cingulum cingulate gyrus L				0.887
- Mean (SD)	0.945 (0.100)	0.939 (0.086)	0.941 (0.089)	
- Range	0.835 - 1.085	0.792 - 1.115	0.792 - 1.115	
Cingulum hippocampus R				0.143
- Mean (SD)	1.106 (0.143)	1.038 (0.092)	1.060 (0.113)	
- Range	0.917 - 1.303	0.887 - 1.212	0.887 - 1.303	
Cingulum hippocampus L				0.002
- Mean (SD)	1.256 (0.183)	1.061 (0.110)	1.124 (0.163)	
- Range	1.007 - 1.484	0.849 - 1.236	0.849 - 1.484	
Fornix R				0.575
- Mean (SD)	1.055 (0.138)	1.083 (0.114)	1.074 (0.121)	
- Range	0.866 - 1.261	0.844 - 1.309	0.844 - 1.309	
Fornix L				0.641
- Mean (SD)	1.015 (0.136)	1.037 (0.108)	1.030 (0.115)	
- Range	0.851 - 1.246	0.841 - 1.278	0.841 - 1.278	
Superior longitudinal fasciculus R				0.925
- Mean (SD)	0.805 (0.052)	0.803 (0.053)	0.803 (0.052)	

	CTR (N=9)	OLM (N=19)	Total (N=28)	p value
- Range	0.758 - 0.898	0.740 - 0.950	0.740 - 0.950	
Superior longitudinal fasciculus L				0.439
- Mean (SD)	0.781 (0.038)	0.796 (0.049)	0.791 (0.046)	
- Range	0.732 - 0.839	0.730 - 0.904	0.730 - 0.904	
Superior fronto occipital fasciculus R				0.967
- Mean (SD)	0.964 (0.172)	0.967 (0.198)	0.966 (0.187)	
- Range	0.718 - 1.300	0.723 - 1.560	0.718 - 1.560	
Superior fronto occipital fasciculus L				0.883
- Mean (SD)	1.088 (0.246)	1.102 (0.236)	1.098 (0.235)	
- Range	0.836 - 1.504	0.819 - 1.744	0.819 - 1.744	
Uncinate fasciculus R				0.727
- Mean (SD)	0.913 (0.179)	0.895 (0.093)	0.900 (0.124)	
- Range	0.782 - 1.375	0.797 - 1.189	0.782 - 1.375	
Uncinate fasciculus L				0.362
- Mean (SD)	0.846 (0.064)	0.885 (0.119)	0.872 (0.104)	
- Range	0.777 - 0.939	0.780 - 1.287	0.777 - 1.287	
Tapetum R				0.382
- Mean (SD)	2.032 (0.341)	2.157 (0.352)	2.117 (0.347)	
- Range	1.379 - 2.374	1.528 - 2.681	1.379 - 2.681	
Tapetum L				0.967
- Mean (SD)	2.404 (0.471)	2.411 (0.370)	2.408 (0.396)	
- Range	1.674 - 3.197	1.678 - 2.936	1.674 - 3.197	

Radial diffusivity (RD, (10^-3)*(mm^2)/s) (DTI) - Offspring of lean mothers (OLM) vs control subjects - (JHU white-matter tractography atlas) - FDR corrected p-values

	CTR (N=9)	OLM (N=19)	Total (N=28)	p value
Middle cerebellar peduncle				0.985
- Mean (SD)	1.089 (0.156)	1.043 (0.096)	1.058 (0.118)	
- Range	0.906 - 1.447	0.857 - 1.268	0.857 - 1.447	
Pontine crossing tract				0.985
- Mean (SD)	0.797 (0.070)	0.797 (0.074)	0.797 (0.071)	
- Range	0.723 - 0.951	0.683 - 0.974	0.683 - 0.974	
Genu of corpus callosum				0.985
- Mean (SD)	1.370 (0.119)	1.362 (0.158)	1.365 (0.145)	
- Range	1.240 - 1.614	1.042 - 1.688	1.042 - 1.688	
Body of corpus callosum				0.985
- Mean (SD)	1.112 (0.116)	1.108 (0.089)	1.109 (0.096)	
- Range	0.990 - 1.319	0.984 - 1.285	0.984 - 1.319	
Splenium of corpus callosum				0.985
- Mean (SD)	1.046 (0.151)	1.011 (0.090)	1.022 (0.112)	
- Range	0.888 - 1.318	0.894 - 1.221	0.888 - 1.318	
Fornix				0.985
- Mean (SD)	2.495 (0.323)	2.388 (0.222)	2.422 (0.258)	
- Range	2.048 - 2.977	1.956 - 2.841	1.956 - 2.977	
Corticospinal tract R				0.985
- Mean (SD)	0.865 (0.094)	0.853 (0.076)	0.857 (0.080)	
- Range	0.754 - 1.012	0.724 - 1.059	0.724 - 1.059	
Corticospinal tract L				0.985
- Mean (SD)	0.824 (0.108)	0.835 (0.079)	0.832 (0.088)	
- Range	0.736 - 1.037	0.723 - 0.968	0.723 - 1.037	
Medial lemniscus R				0.985
- Mean (SD)	0.846 (0.068)	0.874 (0.113)	0.865 (0.100)	
- Range	0.782 - 0.982	0.697 - 1.165	0.697 - 1.165	
Medial lemniscus L				0.985
- Mean (SD)	0.834 (0.046)	0.847 (0.064)	0.843 (0.058)	
- Range	0.783 - 0.935	0.695 - 0.981	0.695 - 0.981	
Inferior cerebellar peduncle R				0.985
- Mean (SD)	1.136 (0.189)	1.076 (0.217)	1.096 (0.207)	
- Range	0.880 - 1.524	0.831 - 1.875	0.831 - 1.875	
Inferior cerebellar peduncle L				0.985
- Mean (SD)	1.057 (0.148)	1.030 (0.127)	1.039 (0.132)	
- Range	0.954 - 1.406	0.864 - 1.363	0.864 - 1.406	
Superior cerebellar peduncle R				0.985
- Mean (SD)	1.209 (0.241)	1.225 (0.158)	1.220 (0.184)	
- Range	1.000 - 1.829	0.890 - 1.472	0.890 - 1.829	
Superior cerebellar peduncle L				0.985
- Mean (SD)	1.265 (0.175)	1.286 (0.177)	1.279 (0.174)	
- Range	1.010 - 1.622	0.968 - 1.618	0.968 - 1.622	
Cerebral peduncle R				0.985
- Mean (SD)	0.874 (0.071)	0.877 (0.048)	0.876 (0.055)	
- Range	0.794 - 1.038	0.785 - 0.951	0.785 - 1.038	
Cerebral peduncle L				0.985
- Mean (SD)	0.842 (0.060)	0.841 (0.050)	0.842 (0.052)	
- Range	0.788 - 0.985	0.733 - 0.926	0.733 - 0.985	
Anterior limb of internal capsule R				0.985
- Mean (SD)	0.846 (0.087)	0.864 (0.069)	0.858 (0.074)	
- Range	0.773 - 1.055	0.774 - 1.020	0.773 - 1.055	
Anterior limb of internal capsule L				0.985
- Mean (SD)	0.877 (0.110)	0.875 (0.077)	0.876 (0.087)	
- Range	0.775 - 1.112	0.772 - 1.040	0.772 - 1.112	
Posterior limb of internal capsule R				0.985
- Mean (SD)	0.770 (0.050)	0.768 (0.041)	0.768 (0.043)	
- Range	0.689 - 0.846	0.705 - 0.857	0.689 - 0.857	
Posterior limb of internal capsule L				0.985
- Mean (SD)	0.770 (0.060)	0.764 (0.038)	0.766 (0.045)	

	CTR (N=9)	OLM (N=19)	Total (N=28)	p value
- Range	0.694 - 0.882	0.703 - 0.847	0.694 - 0.882	
Retrolenticular part of internal capsule R				0.985
- Mean (SD)	0.934 (0.097)	0.904 (0.058)	0.914 (0.072)	
- Range	0.827 - 1.105	0.793 - 1.009	0.793 - 1.105	
Retrolenticular part of internal capsule L				0.985
- Mean (SD)	0.936 (0.111)	0.929 (0.065)	0.931 (0.081)	
- Range	0.837 - 1.171	0.822 - 1.088	0.822 - 1.171	
Anterior corona radiata R				0.985
- Mean (SD)	0.940 (0.079)	0.968 (0.102)	0.959 (0.094)	
- Range	0.835 - 1.070	0.790 - 1.160	0.790 - 1.160	
Anterior corona radiata L				0.985
- Mean (SD)	0.927 (0.066)	0.966 (0.125)	0.953 (0.110)	
- Range	0.841 - 1.030	0.791 - 1.298	0.791 - 1.298	
Superior corona radiata R				0.985
- Mean (SD)	0.821 (0.059)	0.830 (0.083)	0.827 (0.075)	
- Range	0.740 - 0.927	0.708 - 1.096	0.708 - 1.096	
Superior corona radiata L				0.985
- Mean (SD)	0.835 (0.068)	0.845 (0.078)	0.842 (0.074)	
- Range	0.743 - 0.949	0.705 - 1.052	0.705 - 1.052	
Posterior corona radiata R				0.985
- Mean (SD)	0.967 (0.131)	0.966 (0.076)	0.966 (0.095)	
- Range	0.818 - 1.163	0.852 - 1.178	0.818 - 1.178	
Posterior corona radiata L				0.985
- Mean (SD)	1.028 (0.189)	1.001 (0.098)	1.010 (0.131)	
- Range	0.871 - 1.331	0.813 - 1.192	0.813 - 1.331	
Posterior thalamic radiation R				0.985
- Mean (SD)	1.002 (0.097)	1.024 (0.096)	1.017 (0.095)	
- Range	0.857 - 1.174	0.874 - 1.248	0.857 - 1.248	
Posterior thalamic radiation L				0.985
- Mean (SD)	1.137 (0.115)	1.144 (0.167)	1.142 (0.150)	
- Range	0.993 - 1.352	0.934 - 1.606	0.934 - 1.606	
Sagittal stratum R				0.985
- Mean (SD)	0.986 (0.089)	0.990 (0.065)	0.989 (0.072)	
- Range	0.833 - 1.078	0.880 - 1.091	0.833 - 1.091	
Sagittal stratum L				0.985
- Mean (SD)	1.074 (0.143)	1.078 (0.118)	1.077 (0.124)	
- Range	0.903 - 1.379	0.881 - 1.305	0.881 - 1.379	
External capsule R				0.985
- Mean (SD)	0.842 (0.067)	0.868 (0.060)	0.860 (0.062)	
- Range	0.764 - 0.953	0.784 - 0.996	0.764 - 0.996	
External capsule L				0.985
- Mean (SD)	0.822 (0.048)	0.841 (0.060)	0.835 (0.056)	
- Range	0.766 - 0.893	0.765 - 0.953	0.765 - 0.953	
Cingulum cingulate gyrus R				0.985
- Mean (SD)	0.950 (0.134)	0.898 (0.074)	0.914 (0.098)	
- Range	0.802 - 1.199	0.782 - 1.082	0.782 - 1.199	
Cingulum cingulate gyrus L				0.985
- Mean (SD)	0.945 (0.100)	0.939 (0.086)	0.941 (0.089)	
- Range	0.835 - 1.085	0.792 - 1.115	0.792 - 1.115	
Cingulum hippocampus R				0.985
- Mean (SD)	1.106 (0.143)	1.038 (0.092)	1.060 (0.113)	
- Range	0.917 - 1.303	0.887 - 1.212	0.887 - 1.303	
Cingulum hippocampus L				0.077
- Mean (SD)	1.256 (0.183)	1.061 (0.110)	1.124 (0.163)	
- Range	1.007 - 1.484	0.849 - 1.236	0.849 - 1.484	
Fornix R				0.985
- Mean (SD)	1.055 (0.138)	1.083 (0.114)	1.074 (0.121)	
- Range	0.866 - 1.261	0.844 - 1.309	0.844 - 1.309	
Fornix L				0.985
- Mean (SD)	1.015 (0.136)	1.037 (0.108)	1.030 (0.115)	
- Range	0.851 - 1.246	0.841 - 1.278	0.841 - 1.278	
Superior longitudinal fasciculus R				0.985

	CTR (N=9)	OLM (N=19)	Total (N=28)	p value
- Mean (SD)	0.805 (0.052)	0.803 (0.053)	0.803 (0.052)	
- Range	0.758 - 0.898	0.740 - 0.950	0.740 - 0.950	
Superior longitudinal fasciculus L				0.985
- Mean (SD)	0.781 (0.038)	0.796 (0.049)	0.791 (0.046)	
- Range	0.732 - 0.839	0.730 - 0.904	0.730 - 0.904	
Superior fronto occipital fasciculus R				0.985
- Mean (SD)	0.964 (0.172)	0.967 (0.198)	0.966 (0.187)	
- Range	0.718 - 1.300	0.723 - 1.560	0.718 - 1.560	
Superior fronto occipital fasciculus L				0.985
- Mean (SD)	1.088 (0.246)	1.102 (0.236)	1.098 (0.235)	
- Range	0.836 - 1.504	0.819 - 1.744	0.819 - 1.744	
Uncinate fasciculus R				0.985
- Mean (SD)	0.913 (0.179)	0.895 (0.093)	0.900 (0.124)	
- Range	0.782 - 1.375	0.797 - 1.189	0.782 - 1.375	
Uncinate fasciculus L				0.985
- Mean (SD)	0.846 (0.064)	0.885 (0.119)	0.872 (0.104)	
- Range	0.777 - 0.939	0.780 - 1.287	0.777 - 1.287	
Tapetum R				0.985
- Mean (SD)	2.032 (0.341)	2.157 (0.352)	2.117 (0.347)	
- Range	1.379 - 2.374	1.528 - 2.681	1.379 - 2.681	
Tapetum L				0.985
- Mean (SD)	2.404 (0.471)	2.411 (0.370)	2.408 (0.396)	
- Range	1.674 - 3.197	1.678 - 2.936	1.674 - 3.197	

Fractional anisotropy (FA) (DTI) - Offspring of obese mothers (OOM) vs control subjects - (JHU white-matter tractography atlas) - Uncorrected p-values

	CTR (N=9)	OOM (N=16)	Total (N=25)	p value
Middle cerebellar peduncle				0.837
- Mean (SD)	0.406 (0.045)	0.401 (0.062)	0.403 (0.056)	
- Range	0.296 - 0.453	0.284 - 0.467	0.284 - 0.467	
Pontine crossing tract				0.365
- Mean (SD)	0.385 (0.034)	0.399 (0.038)	0.394 (0.037)	
- Range	0.324 - 0.428	0.338 - 0.469	0.324 - 0.469	
Genu of corpus callosum				0.583
- Mean (SD)	0.441 (0.041)	0.434 (0.021)	0.436 (0.029)	
- Range	0.371 - 0.486	0.391 - 0.476	0.371 - 0.486	
Body of corpus callosum				0.235
- Mean (SD)	0.501 (0.043)	0.482 (0.033)	0.489 (0.037)	
- Range	0.425 - 0.554	0.429 - 0.544	0.425 - 0.554	
Splenium of corpus callosum				0.578
- Mean (SD)	0.586 (0.052)	0.595 (0.027)	0.592 (0.037)	
- Range	0.494 - 0.669	0.555 - 0.662	0.494 - 0.669	
Fornix				0.586
- Mean (SD)	0.242 (0.037)	0.251 (0.043)	0.248 (0.040)	
- Range	0.193 - 0.281	0.184 - 0.332	0.184 - 0.332	
Corticospinal tract R				0.391
- Mean (SD)	0.444 (0.042)	0.464 (0.060)	0.457 (0.054)	
- Range	0.369 - 0.499	0.309 - 0.555	0.309 - 0.555	
Corticospinal tract L				0.417
- Mean (SD)	0.446 (0.044)	0.462 (0.047)	0.456 (0.045)	
- Range	0.370 - 0.482	0.350 - 0.522	0.350 - 0.522	
Medial lemniscus R				0.933
- Mean (SD)	0.445 (0.034)	0.443 (0.042)	0.444 (0.039)	
- Range	0.403 - 0.514	0.375 - 0.518	0.375 - 0.518	
Medial lemniscus L				0.767
- Mean (SD)	0.453 (0.036)	0.459 (0.056)	0.457 (0.049)	
- Range	0.404 - 0.506	0.353 - 0.556	0.353 - 0.556	
Inferior cerebellar peduncle R				0.899
- Mean (SD)	0.350 (0.048)	0.352 (0.051)	0.351 (0.049)	
- Range	0.282 - 0.442	0.270 - 0.449	0.270 - 0.449	
Inferior cerebellar peduncle L				0.179
- Mean (SD)	0.361 (0.032)	0.337 (0.047)	0.345 (0.043)	
- Range	0.328 - 0.422	0.230 - 0.408	0.230 - 0.422	
Superior cerebellar peduncle R				0.365
- Mean (SD)	0.471 (0.062)	0.448 (0.062)	0.456 (0.062)	
- Range	0.346 - 0.530	0.298 - 0.517	0.298 - 0.530	
Superior cerebellar peduncle L				0.369
- Mean (SD)	0.447 (0.057)	0.427 (0.050)	0.434 (0.052)	
- Range	0.309 - 0.487	0.308 - 0.495	0.308 - 0.495	
Cerebral peduncle R				0.614
- Mean (SD)	0.569 (0.026)	0.564 (0.019)	0.566 (0.021)	
- Range	0.513 - 0.598	0.534 - 0.608	0.513 - 0.608	
Cerebral peduncle L				0.937
- Mean (SD)	0.572 (0.036)	0.573 (0.022)	0.572 (0.027)	
- Range	0.485 - 0.610	0.535 - 0.608	0.485 - 0.610	
Anterior limb of internal capsule R				0.479
- Mean (SD)	0.444 (0.040)	0.435 (0.025)	0.438 (0.031)	
- Range	0.357 - 0.486	0.380 - 0.475	0.357 - 0.486	
Anterior limb of internal capsule L				0.969
- Mean (SD)	0.442 (0.038)	0.442 (0.028)	0.442 (0.032)	
- Range	0.355 - 0.481	0.399 - 0.490	0.355 - 0.490	
Posterior limb of internal capsule R				0.793
- Mean (SD)	0.533 (0.033)	0.530 (0.023)	0.532 (0.027)	
- Range	0.463 - 0.574	0.502 - 0.586	0.463 - 0.586	
Posterior limb of internal capsule L				0.771
- Mean (SD)	0.543 (0.042)	0.540 (0.021)	0.541 (0.029)	
- Range	0.453 - 0.591	0.508 - 0.595	0.453 - 0.595	

	CTR (N=9)	OOM (N=16)	Total (N=25)	p value
Retrolenticular part of internal capsule R				0.884
- Mean (SD)	0.442 (0.037)	0.440 (0.022)	0.441 (0.027)	
- Range	0.375 - 0.501	0.402 - 0.482	0.375 - 0.501	
Retrolenticular part of internal capsule L				0.572
- Mean (SD)	0.459 (0.031)	0.452 (0.031)	0.454 (0.030)	
- Range	0.405 - 0.496	0.404 - 0.503	0.404 - 0.503	
Anterior corona radiata R				0.777
- Mean (SD)	0.334 (0.029)	0.337 (0.023)	0.336 (0.025)	
- Range	0.273 - 0.364	0.304 - 0.375	0.273 - 0.375	
Anterior corona radiata L				0.644
- Mean (SD)	0.334 (0.027)	0.340 (0.026)	0.338 (0.026)	
- Range	0.280 - 0.358	0.284 - 0.392	0.280 - 0.392	
Superior corona radiata R				0.250
- Mean (SD)	0.416 (0.037)	0.400 (0.029)	0.406 (0.032)	
- Range	0.366 - 0.470	0.351 - 0.444	0.351 - 0.470	
Superior corona radiata L				0.691
- Mean (SD)	0.414 (0.031)	0.408 (0.034)	0.410 (0.032)	
- Range	0.356 - 0.452	0.362 - 0.461	0.356 - 0.461	
Posterior corona radiata R				0.324
- Mean (SD)	0.430 (0.047)	0.415 (0.030)	0.420 (0.036)	
- Range	0.373 - 0.513	0.369 - 0.463	0.369 - 0.513	
Posterior corona radiata L				0.757
- Mean (SD)	0.412 (0.034)	0.407 (0.038)	0.409 (0.036)	
- Range	0.368 - 0.466	0.340 - 0.487	0.340 - 0.487	
Posterior thalamic radiation R				0.923
- Mean (SD)	0.472 (0.034)	0.471 (0.030)	0.472 (0.031)	
- Range	0.415 - 0.543	0.412 - 0.529	0.412 - 0.543	
Posterior thalamic radiation L				0.467
- Mean (SD)	0.447 (0.044)	0.457 (0.025)	0.453 (0.032)	
- Range	0.358 - 0.494	0.402 - 0.499	0.358 - 0.499	
Sagittal stratum R				0.225
- Mean (SD)	0.447 (0.032)	0.431 (0.029)	0.437 (0.030)	
- Range	0.406 - 0.506	0.391 - 0.507	0.391 - 0.507	
Sagittal stratum L				0.408
- Mean (SD)	0.404 (0.031)	0.394 (0.025)	0.398 (0.027)	
- Range	0.349 - 0.444	0.349 - 0.445	0.349 - 0.445	
External capsule R				0.549
- Mean (SD)	0.336 (0.026)	0.331 (0.018)	0.333 (0.021)	
- Range	0.295 - 0.366	0.305 - 0.381	0.295 - 0.381	
External capsule L				0.545
- Mean (SD)	0.343 (0.021)	0.339 (0.017)	0.340 (0.018)	
- Range	0.295 - 0.371	0.317 - 0.377	0.295 - 0.377	
Cingulum cingulate gyrus R				0.168
- Mean (SD)	0.368 (0.040)	0.351 (0.017)	0.357 (0.028)	
- Range	0.293 - 0.425	0.325 - 0.385	0.293 - 0.425	
Cingulum cingulate gyrus L				0.163
- Mean (SD)	0.383 (0.041)	0.363 (0.028)	0.370 (0.034)	
- Range	0.329 - 0.430	0.313 - 0.416	0.313 - 0.430	
Cingulum hippocampus R				0.417
- Mean (SD)	0.297 (0.030)	0.286 (0.034)	0.290 (0.032)	
- Range	0.250 - 0.340	0.249 - 0.360	0.249 - 0.360	
Cingulum hippocampus L				0.869
- Mean (SD)	0.273 (0.032)	0.271 (0.023)	0.272 (0.026)	
- Range	0.226 - 0.332	0.234 - 0.309	0.226 - 0.332	
Fornix R				0.248
- Mean (SD)	0.375 (0.031)	0.360 (0.031)	0.365 (0.031)	
- Range	0.317 - 0.412	0.306 - 0.421	0.306 - 0.421	
Fornix L				0.905
- Mean (SD)	0.383 (0.027)	0.381 (0.031)	0.382 (0.029)	
- Range	0.331 - 0.432	0.326 - 0.433	0.326 - 0.433	
Superior longitudinal fasciculus R				0.419
- Mean (SD)	0.395 (0.035)	0.386 (0.018)	0.389 (0.025)	

	CTR (N=9)	OOM (N=16)	Total (N=25)	p value
- Range	0.344 - 0.442	0.346 - 0.422	0.344 - 0.442	
Superior longitudinal fasciculus L				0.478
- Mean (SD)	0.399 (0.040)	0.389 (0.024)	0.393 (0.031)	
- Range	0.327 - 0.463	0.350 - 0.437	0.327 - 0.463	
Superior fronto occipital fasciculus R				0.833
- Mean (SD)	0.359 (0.047)	0.363 (0.029)	0.362 (0.036)	
- Range	0.279 - 0.415	0.293 - 0.398	0.279 - 0.415	
Superior fronto occipital fasciculus L				0.309
- Mean (SD)	0.324 (0.053)	0.342 (0.033)	0.335 (0.041)	
- Range	0.234 - 0.389	0.267 - 0.410	0.234 - 0.410	
Uncinate fasciculus R				0.547
- Mean (SD)	0.381 (0.049)	0.391 (0.033)	0.387 (0.039)	
- Range	0.288 - 0.424	0.328 - 0.447	0.288 - 0.447	
Uncinate fasciculus L				0.315
- Mean (SD)	0.385 (0.036)	0.402 (0.044)	0.396 (0.041)	
- Range	0.333 - 0.434	0.328 - 0.462	0.328 - 0.462	
Tapetum R				0.971
- Mean (SD)	0.343 (0.037)	0.342 (0.030)	0.342 (0.032)	
- Range	0.268 - 0.386	0.283 - 0.387	0.268 - 0.387	
Tapetum L				0.964
- Mean (SD)	0.307 (0.018)	0.307 (0.029)	0.307 (0.025)	
- Range	0.278 - 0.332	0.257 - 0.372	0.257 - 0.372	

Fractional anisotropy (FA) (DTI) - Offspring of obese mothers (OOM) vs control subjects - (JHU white-matter tractography atlas) - FDR corrected p-values

	CTR (N=9)	OOM (N=16)	Total (N=25)	p value
Middle cerebellar peduncle				0.971
- Mean (SD)	0.406 (0.045)	0.401 (0.062)	0.403 (0.056)	
- Range	0.296 - 0.453	0.284 - 0.467	0.284 - 0.467	
Pontine crossing tract				0.971
- Mean (SD)	0.385 (0.034)	0.399 (0.038)	0.394 (0.037)	
- Range	0.324 - 0.428	0.338 - 0.469	0.324 - 0.469	
Genu of corpus callosum				0.971
- Mean (SD)	0.441 (0.041)	0.434 (0.021)	0.436 (0.029)	
- Range	0.371 - 0.486	0.391 - 0.476	0.371 - 0.486	
Body of corpus callosum				0.971
- Mean (SD)	0.501 (0.043)	0.482 (0.033)	0.489 (0.037)	
- Range	0.425 - 0.554	0.429 - 0.544	0.425 - 0.554	
Splenium of corpus callosum				0.971
- Mean (SD)	0.586 (0.052)	0.595 (0.027)	0.592 (0.037)	
- Range	0.494 - 0.669	0.555 - 0.662	0.494 - 0.669	
Fornix				0.971
- Mean (SD)	0.242 (0.037)	0.251 (0.043)	0.248 (0.040)	
- Range	0.193 - 0.281	0.184 - 0.332	0.184 - 0.332	
Corticospinal tract R				0.971
- Mean (SD)	0.444 (0.042)	0.464 (0.060)	0.457 (0.054)	
- Range	0.369 - 0.499	0.309 - 0.555	0.309 - 0.555	
Corticospinal tract L				0.971
- Mean (SD)	0.446 (0.044)	0.462 (0.047)	0.456 (0.045)	
- Range	0.370 - 0.482	0.350 - 0.522	0.350 - 0.522	
Medial lemniscus R				0.971
- Mean (SD)	0.445 (0.034)	0.443 (0.042)	0.444 (0.039)	
- Range	0.403 - 0.514	0.375 - 0.518	0.375 - 0.518	
Medial lemniscus L				0.971
- Mean (SD)	0.453 (0.036)	0.459 (0.056)	0.457 (0.049)	
- Range	0.404 - 0.506	0.353 - 0.556	0.353 - 0.556	
Inferior cerebellar peduncle R				0.971
- Mean (SD)	0.350 (0.048)	0.352 (0.051)	0.351 (0.049)	
- Range	0.282 - 0.442	0.270 - 0.449	0.270 - 0.449	
Inferior cerebellar peduncle L				0.971
- Mean (SD)	0.361 (0.032)	0.337 (0.047)	0.345 (0.043)	
- Range	0.328 - 0.422	0.230 - 0.408	0.230 - 0.422	
Superior cerebellar peduncle R				0.971
- Mean (SD)	0.471 (0.062)	0.448 (0.062)	0.456 (0.062)	
- Range	0.346 - 0.530	0.298 - 0.517	0.298 - 0.530	
Superior cerebellar peduncle L				0.971
- Mean (SD)	0.447 (0.057)	0.427 (0.050)	0.434 (0.052)	
- Range	0.309 - 0.487	0.308 - 0.495	0.308 - 0.495	
Cerebral peduncle R				0.971
- Mean (SD)	0.569 (0.026)	0.564 (0.019)	0.566 (0.021)	
- Range	0.513 - 0.598	0.534 - 0.608	0.513 - 0.608	
Cerebral peduncle L				0.971
- Mean (SD)	0.572 (0.036)	0.573 (0.022)	0.572 (0.027)	
- Range	0.485 - 0.610	0.535 - 0.608	0.485 - 0.610	
Anterior limb of internal capsule R				0.971
- Mean (SD)	0.444 (0.040)	0.435 (0.025)	0.438 (0.031)	
- Range	0.357 - 0.486	0.380 - 0.475	0.357 - 0.486	
Anterior limb of internal capsule L				0.971
- Mean (SD)	0.442 (0.038)	0.442 (0.028)	0.442 (0.032)	
- Range	0.355 - 0.481	0.399 - 0.490	0.355 - 0.490	
Posterior limb of internal capsule R				0.971
- Mean (SD)	0.533 (0.033)	0.530 (0.023)	0.532 (0.027)	
- Range	0.463 - 0.574	0.502 - 0.586	0.463 - 0.586	
Posterior limb of internal capsule L				0.971
- Mean (SD)	0.543 (0.042)	0.540 (0.021)	0.541 (0.029)	
- Range	0.453 - 0.591	0.508 - 0.595	0.453 - 0.595	

	CTR (N=9)	OOM (N=16)	Total (N=25)	p value
Retrolenticular part of internal capsule R				0.971
- Mean (SD)	0.442 (0.037)	0.440 (0.022)	0.441 (0.027)	
- Range	0.375 - 0.501	0.402 - 0.482	0.375 - 0.501	
Retrolenticular part of internal capsule L				0.971
- Mean (SD)	0.459 (0.031)	0.452 (0.031)	0.454 (0.030)	
- Range	0.405 - 0.496	0.404 - 0.503	0.404 - 0.503	
Anterior corona radiata R				0.971
- Mean (SD)	0.334 (0.029)	0.337 (0.023)	0.336 (0.025)	
- Range	0.273 - 0.364	0.304 - 0.375	0.273 - 0.375	
Anterior corona radiata L				0.971
- Mean (SD)	0.334 (0.027)	0.340 (0.026)	0.338 (0.026)	
- Range	0.280 - 0.358	0.284 - 0.392	0.280 - 0.392	
Superior corona radiata R				0.971
- Mean (SD)	0.416 (0.037)	0.400 (0.029)	0.406 (0.032)	
- Range	0.366 - 0.470	0.351 - 0.444	0.351 - 0.470	
Superior corona radiata L				0.971
- Mean (SD)	0.414 (0.031)	0.408 (0.034)	0.410 (0.032)	
- Range	0.356 - 0.452	0.362 - 0.461	0.356 - 0.461	
Posterior corona radiata R				0.971
- Mean (SD)	0.430 (0.047)	0.415 (0.030)	0.420 (0.036)	
- Range	0.373 - 0.513	0.369 - 0.463	0.369 - 0.513	
Posterior corona radiata L				0.971
- Mean (SD)	0.412 (0.034)	0.407 (0.038)	0.409 (0.036)	
- Range	0.368 - 0.466	0.340 - 0.487	0.340 - 0.487	
Posterior thalamic radiation R				0.971
- Mean (SD)	0.472 (0.034)	0.471 (0.030)	0.472 (0.031)	
- Range	0.415 - 0.543	0.412 - 0.529	0.412 - 0.543	
Posterior thalamic radiation L				0.971
- Mean (SD)	0.447 (0.044)	0.457 (0.025)	0.453 (0.032)	
- Range	0.358 - 0.494	0.402 - 0.499	0.358 - 0.499	
Sagittal stratum R				0.971
- Mean (SD)	0.447 (0.032)	0.431 (0.029)	0.437 (0.030)	
- Range	0.406 - 0.506	0.391 - 0.507	0.391 - 0.507	
Sagittal stratum L				0.971
- Mean (SD)	0.404 (0.031)	0.394 (0.025)	0.398 (0.027)	
- Range	0.349 - 0.444	0.349 - 0.445	0.349 - 0.445	
External capsule R				0.971
- Mean (SD)	0.336 (0.026)	0.331 (0.018)	0.333 (0.021)	
- Range	0.295 - 0.366	0.305 - 0.381	0.295 - 0.381	
External capsule L				0.971
- Mean (SD)	0.343 (0.021)	0.339 (0.017)	0.340 (0.018)	
- Range	0.295 - 0.371	0.317 - 0.377	0.295 - 0.377	
Cingulum cingulate gyrus R				0.971
- Mean (SD)	0.368 (0.040)	0.351 (0.017)	0.357 (0.028)	
- Range	0.293 - 0.425	0.325 - 0.385	0.293 - 0.425	
Cingulum cingulate gyrus L				0.971
- Mean (SD)	0.383 (0.041)	0.363 (0.028)	0.370 (0.034)	
- Range	0.329 - 0.430	0.313 - 0.416	0.313 - 0.430	
Cingulum hippocampus R				0.971
- Mean (SD)	0.297 (0.030)	0.286 (0.034)	0.290 (0.032)	
- Range	0.250 - 0.340	0.249 - 0.360	0.249 - 0.360	
Cingulum hippocampus L				0.971
- Mean (SD)	0.273 (0.032)	0.271 (0.023)	0.272 (0.026)	
- Range	0.226 - 0.332	0.234 - 0.309	0.226 - 0.332	
Fornix R				0.971
- Mean (SD)	0.375 (0.031)	0.360 (0.031)	0.365 (0.031)	
- Range	0.317 - 0.412	0.306 - 0.421	0.306 - 0.421	
Fornix L				0.971
- Mean (SD)	0.383 (0.027)	0.381 (0.031)	0.382 (0.029)	
- Range	0.331 - 0.432	0.326 - 0.433	0.326 - 0.433	
Superior longitudinal fasciculus R				0.971
- Mean (SD)	0.395 (0.035)	0.386 (0.018)	0.389 (0.025)	

	CTR (N=9)	OOM (N=16)	Total (N=25)	p value
- Range	0.344 - 0.442	0.346 - 0.422	0.344 - 0.442	
Superior longitudinal fasciculus L				0.971
- Mean (SD)	0.399 (0.040)	0.389 (0.024)	0.393 (0.031)	
- Range	0.327 - 0.463	0.350 - 0.437	0.327 - 0.463	
Superior fronto occipital fasciculus R				0.971
- Mean (SD)	0.359 (0.047)	0.363 (0.029)	0.362 (0.036)	
- Range	0.279 - 0.415	0.293 - 0.398	0.279 - 0.415	
Superior fronto occipital fasciculus L				0.971
- Mean (SD)	0.324 (0.053)	0.342 (0.033)	0.335 (0.041)	
- Range	0.234 - 0.389	0.267 - 0.410	0.234 - 0.410	
Uncinate fasciculus R				0.971
- Mean (SD)	0.381 (0.049)	0.391 (0.033)	0.387 (0.039)	
- Range	0.288 - 0.424	0.328 - 0.447	0.288 - 0.447	
Uncinate fasciculus L				0.971
- Mean (SD)	0.385 (0.036)	0.402 (0.044)	0.396 (0.041)	
- Range	0.333 - 0.434	0.328 - 0.462	0.328 - 0.462	
Tapetum R				0.971
- Mean (SD)	0.343 (0.037)	0.342 (0.030)	0.342 (0.032)	
- Range	0.268 - 0.386	0.283 - 0.387	0.268 - 0.387	
Tapetum L				0.971
- Mean (SD)	0.307 (0.018)	0.307 (0.029)	0.307 (0.025)	
- Range	0.278 - 0.332	0.257 - 0.372	0.257 - 0.372	

Mean diffusivity (MD, (10^-3)*(mm^2)/s) (DTI) - Offspring of obese mothers (OOM) vs control subjects - (JHU white-matter tractography atlas) - Uncorrected p-values

	CTR (N=9)	OOM (N=16)	Total (N=25)	p value
Middle cerebellar peduncle				0.556
- Mean (SD)	1.089 (0.156)	1.128 (0.158)	1.114 (0.155)	
- Range	0.906 - 1.447	0.924 - 1.433	0.906 - 1.447	
Pontine crossing tract				0.388
- Mean (SD)	0.797 (0.070)	0.836 (0.121)	0.822 (0.106)	
- Range	0.723 - 0.951	0.698 - 1.162	0.698 - 1.162	
Genu of corpus callosum				0.945
- Mean (SD)	1.370 (0.119)	1.367 (0.116)	1.368 (0.115)	
- Range	1.240 - 1.614	1.231 - 1.664	1.231 - 1.664	
Body of corpus callosum				0.558
- Mean (SD)	1.112 (0.116)	1.139 (0.106)	1.130 (0.108)	
- Range	0.990 - 1.319	1.005 - 1.370	0.990 - 1.370	
Splenium of corpus callosum				0.704
- Mean (SD)	1.046 (0.151)	1.026 (0.099)	1.033 (0.118)	
- Range	0.888 - 1.318	0.886 - 1.287	0.886 - 1.318	
Fornix				0.316
- Mean (SD)	2.495 (0.323)	2.347 (0.359)	2.401 (0.347)	
- Range	2.048 - 2.977	1.853 - 2.857	1.853 - 2.977	
Corticospinal tract R				0.452
- Mean (SD)	0.865 (0.094)	0.899 (0.116)	0.887 (0.108)	
- Range	0.754 - 1.012	0.741 - 1.250	0.741 - 1.250	
Corticospinal tract L				0.522
- Mean (SD)	0.824 (0.108)	0.851 (0.097)	0.841 (0.100)	
- Range	0.736 - 1.037	0.731 - 1.159	0.731 - 1.159	
Medial lemniscus R				0.142
- Mean (SD)	0.846 (0.068)	0.903 (0.099)	0.882 (0.092)	
- Range	0.782 - 0.982	0.812 - 1.210	0.782 - 1.210	
Medial lemniscus L				0.066
- Mean (SD)	0.834 (0.046)	0.882 (0.066)	0.865 (0.063)	
- Range	0.783 - 0.935	0.796 - 1.034	0.783 - 1.034	
Inferior cerebellar peduncle R				0.474
- Mean (SD)	1.136 (0.189)	1.201 (0.226)	1.178 (0.212)	
- Range	0.880 - 1.524	0.927 - 1.639	0.880 - 1.639	
Inferior cerebellar peduncle L				0.392
- Mean (SD)	1.057 (0.148)	1.134 (0.237)	1.106 (0.209)	
- Range	0.954 - 1.406	0.917 - 1.748	0.917 - 1.748	
Superior cerebellar peduncle R				0.580
- Mean (SD)	1.209 (0.241)	1.253 (0.152)	1.237 (0.185)	
- Range	1.000 - 1.829	0.947 - 1.532	0.947 - 1.829	
Superior cerebellar peduncle L				0.510
- Mean (SD)	1.265 (0.175)	1.316 (0.184)	1.298 (0.179)	
- Range	1.010 - 1.622	0.913 - 1.623	0.913 - 1.623	
Cerebral peduncle R				0.770
- Mean (SD)	0.874 (0.071)	0.881 (0.048)	0.879 (0.056)	
- Range	0.794 - 1.038	0.806 - 0.971	0.794 - 1.038	
Cerebral peduncle L				0.829
- Mean (SD)	0.842 (0.060)	0.838 (0.041)	0.839 (0.047)	
- Range	0.788 - 0.985	0.775 - 0.927	0.775 - 0.985	
Anterior limb of internal capsule R				0.948
- Mean (SD)	0.846 (0.087)	0.844 (0.056)	0.845 (0.067)	
- Range	0.773 - 1.055	0.781 - 0.962	0.773 - 1.055	
Anterior limb of internal capsule L				0.751
- Mean (SD)	0.877 (0.110)	0.865 (0.071)	0.869 (0.085)	
- Range	0.775 - 1.112	0.753 - 1.025	0.753 - 1.112	
Posterior limb of internal capsule R				0.562
- Mean (SD)	0.770 (0.050)	0.761 (0.024)	0.764 (0.035)	
- Range	0.689 - 0.846	0.721 - 0.794	0.689 - 0.846	
Posterior limb of internal capsule L				0.754
- Mean (SD)	0.770 (0.060)	0.764 (0.028)	0.766 (0.041)	

	CTR (N=9)	OOM (N=16)	Total (N=25)	p value
- Range	0.694 - 0.882	0.717 - 0.819	0.694 - 0.882	
Retrolenticular part of internal capsule R				0.705
- Mean (SD)	0.934 (0.097)	0.920 (0.083)	0.925 (0.086)	
- Range	0.827 - 1.105	0.780 - 1.062	0.780 - 1.105	
Retrolenticular part of internal capsule L				0.894
- Mean (SD)	0.936 (0.111)	0.931 (0.078)	0.932 (0.089)	
- Range	0.837 - 1.171	0.814 - 1.074	0.814 - 1.171	
Anterior corona radiata R				0.652
- Mean (SD)	0.940 (0.079)	0.921 (0.112)	0.928 (0.100)	
- Range	0.835 - 1.070	0.823 - 1.248	0.823 - 1.248	
Anterior corona radiata L				0.741
- Mean (SD)	0.927 (0.066)	0.915 (0.099)	0.919 (0.087)	
- Range	0.841 - 1.030	0.821 - 1.215	0.821 - 1.215	
Superior corona radiata R				0.546
- Mean (SD)	0.821 (0.059)	0.804 (0.072)	0.810 (0.067)	
- Range	0.740 - 0.927	0.735 - 0.946	0.735 - 0.946	
Superior corona radiata L				0.808
- Mean (SD)	0.835 (0.068)	0.826 (0.094)	0.830 (0.084)	
- Range	0.743 - 0.949	0.733 - 1.099	0.733 - 1.099	
Posterior corona radiata R				0.493
- Mean (SD)	0.967 (0.131)	0.930 (0.123)	0.943 (0.125)	
- Range	0.818 - 1.163	0.780 - 1.146	0.780 - 1.163	
Posterior corona radiata L				0.350
- Mean (SD)	1.028 (0.189)	0.964 (0.145)	0.987 (0.161)	
- Range	0.871 - 1.331	0.800 - 1.328	0.800 - 1.331	
Posterior thalamic radiation R				0.930
- Mean (SD)	1.002 (0.097)	0.996 (0.167)	0.998 (0.143)	
- Range	0.857 - 1.174	0.828 - 1.510	0.828 - 1.510	
Posterior thalamic radiation L				0.568
- Mean (SD)	1.137 (0.115)	1.099 (0.175)	1.113 (0.155)	
- Range	0.993 - 1.352	0.935 - 1.589	0.935 - 1.589	
Sagittal stratum R				0.727
- Mean (SD)	0.986 (0.089)	1.003 (0.124)	0.997 (0.111)	
- Range	0.833 - 1.078	0.844 - 1.335	0.833 - 1.335	
Sagittal stratum L				0.750
- Mean (SD)	1.074 (0.143)	1.057 (0.105)	1.063 (0.118)	
- Range	0.903 - 1.379	0.895 - 1.279	0.895 - 1.379	
External capsule R				0.820
- Mean (SD)	0.842 (0.067)	0.848 (0.057)	0.846 (0.059)	
- Range	0.764 - 0.953	0.753 - 0.955	0.753 - 0.955	
External capsule L				0.642
- Mean (SD)	0.822 (0.048)	0.832 (0.052)	0.829 (0.049)	
- Range	0.766 - 0.893	0.771 - 0.945	0.766 - 0.945	
Cingulum cingulate gyrus R				0.515
- Mean (SD)	0.950 (0.134)	0.924 (0.058)	0.934 (0.090)	
- Range	0.802 - 1.199	0.819 - 1.043	0.802 - 1.199	
Cingulum cingulate gyrus L				0.680
- Mean (SD)	0.945 (0.100)	0.959 (0.074)	0.954 (0.082)	
- Range	0.835 - 1.085	0.839 - 1.108	0.835 - 1.108	
Cingulum hippocampus R				0.822
- Mean (SD)	1.106 (0.143)	1.121 (0.168)	1.115 (0.157)	
- Range	0.917 - 1.303	0.934 - 1.523	0.917 - 1.523	
Cingulum hippocampus L				0.495
- Mean (SD)	1.256 (0.183)	1.200 (0.199)	1.220 (0.192)	
- Range	1.007 - 1.484	0.930 - 1.734	0.930 - 1.734	
Fornix R				0.638
- Mean (SD)	1.055 (0.138)	1.086 (0.163)	1.075 (0.152)	
- Range	0.866 - 1.261	0.884 - 1.408	0.866 - 1.408	
Fornix L				0.965
- Mean (SD)	1.015 (0.136)	1.012 (0.146)	1.013 (0.140)	
- Range	0.851 - 1.246	0.868 - 1.331	0.851 - 1.331	
Superior longitudinal fasciculus R				0.857

	CTR (N=9)	OOM (N=16)	Total (N=25)	p value
- Mean (SD)	0.805 (0.052)	0.801 (0.048)	0.802 (0.048)	
- Range	0.758 - 0.898	0.727 - 0.883	0.727 - 0.898	
Superior longitudinal fasciculus L				0.715
- Mean (SD)	0.781 (0.038)	0.788 (0.045)	0.785 (0.042)	
- Range	0.732 - 0.839	0.728 - 0.865	0.728 - 0.865	
Superior fronto occipital fasciculus R				0.599
- Mean (SD)	0.964 (0.172)	0.917 (0.228)	0.934 (0.207)	
- Range	0.718 - 1.300	0.733 - 1.571	0.718 - 1.571	
Superior fronto occipital fasciculus L				0.480
- Mean (SD)	1.088 (0.246)	1.007 (0.283)	1.036 (0.268)	
- Range	0.836 - 1.504	0.795 - 1.863	0.795 - 1.863	
Uncinate fasciculus R				0.804
- Mean (SD)	0.913 (0.179)	0.929 (0.142)	0.923 (0.153)	
- Range	0.782 - 1.375	0.790 - 1.267	0.782 - 1.375	
Uncinate fasciculus L				0.431
- Mean (SD)	0.846 (0.064)	0.871 (0.082)	0.862 (0.076)	
- Range	0.777 - 0.939	0.784 - 1.136	0.777 - 1.136	
Tapetum R				0.608
- Mean (SD)	2.032 (0.341)	1.948 (0.413)	1.978 (0.383)	
- Range	1.379 - 2.374	1.141 - 2.588	1.141 - 2.588	
Tapetum L				0.267
- Mean (SD)	2.404 (0.471)	2.191 (0.436)	2.268 (0.451)	
- Range	1.674 - 3.197	1.407 - 2.919	1.407 - 3.197	

Mean diffusivity (MD, (10^-3)*(mm^2)/s) (DTI) - Offspring of obese mothers (OOM) vs control subjects - (JHU white-matter tractography atlas) - FDR corrected p-values

	CTR (N=9)	OOM (N=16)	Total (N=25)	p value
Middle cerebellar peduncle				0.948
- Mean (SD)	1.089 (0.156)	1.128 (0.158)	1.114 (0.155)	
- Range	0.906 - 1.447	0.924 - 1.433	0.906 - 1.447	
Pontine crossing tract				0.948
- Mean (SD)	0.797 (0.070)	0.836 (0.121)	0.822 (0.106)	
- Range	0.723 - 0.951	0.698 - 1.162	0.698 - 1.162	
Genu of corpus callosum				0.965
- Mean (SD)	1.370 (0.119)	1.367 (0.116)	1.368 (0.115)	
- Range	1.240 - 1.614	1.231 - 1.664	1.231 - 1.664	
Body of corpus callosum				0.948
- Mean (SD)	1.112 (0.116)	1.139 (0.106)	1.130 (0.108)	
- Range	0.990 - 1.319	1.005 - 1.370	0.990 - 1.370	
Splenium of corpus callosum				0.948
- Mean (SD)	1.046 (0.151)	1.026 (0.099)	1.033 (0.118)	
- Range	0.888 - 1.318	0.886 - 1.287	0.886 - 1.318	
Fornix				0.948
- Mean (SD)	2.495 (0.323)	2.347 (0.359)	2.401 (0.347)	
- Range	2.048 - 2.977	1.853 - 2.857	1.853 - 2.977	
Corticospinal tract R				0.948
- Mean (SD)	0.865 (0.094)	0.899 (0.116)	0.887 (0.108)	
- Range	0.754 - 1.012	0.741 - 1.250	0.741 - 1.250	
Corticospinal tract L				0.948
- Mean (SD)	0.824 (0.108)	0.851 (0.097)	0.841 (0.100)	
- Range	0.736 - 1.037	0.731 - 1.159	0.731 - 1.159	
Medial lemniscus R				0.948
- Mean (SD)	0.846 (0.068)	0.903 (0.099)	0.882 (0.092)	
- Range	0.782 - 0.982	0.812 - 1.210	0.782 - 1.210	
Medial lemniscus L				0.948
- Mean (SD)	0.834 (0.046)	0.882 (0.066)	0.865 (0.063)	
- Range	0.783 - 0.935	0.796 - 1.034	0.783 - 1.034	
Inferior cerebellar peduncle R				0.948
- Mean (SD)	1.136 (0.189)	1.201 (0.226)	1.178 (0.212)	
- Range	0.880 - 1.524	0.927 - 1.639	0.880 - 1.639	
Inferior cerebellar peduncle L				0.948
- Mean (SD)	1.057 (0.148)	1.134 (0.237)	1.106 (0.209)	
- Range	0.954 - 1.406	0.917 - 1.748	0.917 - 1.748	
Superior cerebellar peduncle R				0.948
- Mean (SD)	1.209 (0.241)	1.253 (0.152)	1.237 (0.185)	
- Range	1.000 - 1.829	0.947 - 1.532	0.947 - 1.829	
Superior cerebellar peduncle L				0.948
- Mean (SD)	1.265 (0.175)	1.316 (0.184)	1.298 (0.179)	
- Range	1.010 - 1.622	0.913 - 1.623	0.913 - 1.623	
Cerebral peduncle R				0.948
- Mean (SD)	0.874 (0.071)	0.881 (0.048)	0.879 (0.056)	
- Range	0.794 - 1.038	0.806 - 0.971	0.794 - 1.038	
Cerebral peduncle L				0.948
- Mean (SD)	0.842 (0.060)	0.838 (0.041)	0.839 (0.047)	
- Range	0.788 - 0.985	0.775 - 0.927	0.775 - 0.985	
Anterior limb of internal capsule R				0.965
- Mean (SD)	0.846 (0.087)	0.844 (0.056)	0.845 (0.067)	
- Range	0.773 - 1.055	0.781 - 0.962	0.773 - 1.055	
Anterior limb of internal capsule L				0.948
- Mean (SD)	0.877 (0.110)	0.865 (0.071)	0.869 (0.085)	
- Range	0.775 - 1.112	0.753 - 1.025	0.753 - 1.112	
Posterior limb of internal capsule R				0.948
- Mean (SD)	0.770 (0.050)	0.761 (0.024)	0.764 (0.035)	
- Range	0.689 - 0.846	0.721 - 0.794	0.689 - 0.846	
Posterior limb of internal capsule L				0.948
- Mean (SD)	0.770 (0.060)	0.764 (0.028)	0.766 (0.041)	

	CTR (N=9)	OOM (N=16)	Total (N=25)	p value
- Range	0.694 - 0.882	0.717 - 0.819	0.694 - 0.882	
Retrolenticular part of internal capsule R				0.948
- Mean (SD)	0.934 (0.097)	0.920 (0.083)	0.925 (0.086)	
- Range	0.827 - 1.105	0.780 - 1.062	0.780 - 1.105	
Retrolenticular part of internal capsule L				0.965
- Mean (SD)	0.936 (0.111)	0.931 (0.078)	0.932 (0.089)	
- Range	0.837 - 1.171	0.814 - 1.074	0.814 - 1.171	
Anterior corona radiata R				0.948
- Mean (SD)	0.940 (0.079)	0.921 (0.112)	0.928 (0.100)	
- Range	0.835 - 1.070	0.823 - 1.248	0.823 - 1.248	
Anterior corona radiata L				0.948
- Mean (SD)	0.927 (0.066)	0.915 (0.099)	0.919 (0.087)	
- Range	0.841 - 1.030	0.821 - 1.215	0.821 - 1.215	
Superior corona radiata R				0.948
- Mean (SD)	0.821 (0.059)	0.804 (0.072)	0.810 (0.067)	
- Range	0.740 - 0.927	0.735 - 0.946	0.735 - 0.946	
Superior corona radiata L				0.948
- Mean (SD)	0.835 (0.068)	0.826 (0.094)	0.830 (0.084)	
- Range	0.743 - 0.949	0.733 - 1.099	0.733 - 1.099	
Posterior corona radiata R				0.948
- Mean (SD)	0.967 (0.131)	0.930 (0.123)	0.943 (0.125)	
- Range	0.818 - 1.163	0.780 - 1.146	0.780 - 1.163	
Posterior corona radiata L				0.948
- Mean (SD)	1.028 (0.189)	0.964 (0.145)	0.987 (0.161)	
- Range	0.871 - 1.331	0.800 - 1.328	0.800 - 1.331	
Posterior thalamic radiation R				0.965
- Mean (SD)	1.002 (0.097)	0.996 (0.167)	0.998 (0.143)	
- Range	0.857 - 1.174	0.828 - 1.510	0.828 - 1.510	
Posterior thalamic radiation L				0.948
- Mean (SD)	1.137 (0.115)	1.099 (0.175)	1.113 (0.155)	
- Range	0.993 - 1.352	0.935 - 1.589	0.935 - 1.589	
Sagittal stratum R				0.948
- Mean (SD)	0.986 (0.089)	1.003 (0.124)	0.997 (0.111)	
- Range	0.833 - 1.078	0.844 - 1.335	0.833 - 1.335	
Sagittal stratum L				0.948
- Mean (SD)	1.074 (0.143)	1.057 (0.105)	1.063 (0.118)	
- Range	0.903 - 1.379	0.895 - 1.279	0.895 - 1.379	
External capsule R				0.948
- Mean (SD)	0.842 (0.067)	0.848 (0.057)	0.846 (0.059)	
- Range	0.764 - 0.953	0.753 - 0.955	0.753 - 0.955	
External capsule L				0.948
- Mean (SD)	0.822 (0.048)	0.832 (0.052)	0.829 (0.049)	
- Range	0.766 - 0.893	0.771 - 0.945	0.766 - 0.945	
Cingulum cingulate gyrus R				0.948
- Mean (SD)	0.950 (0.134)	0.924 (0.058)	0.934 (0.090)	
- Range	0.802 - 1.199	0.819 - 1.043	0.802 - 1.199	
Cingulum cingulate gyrus L				0.948
- Mean (SD)	0.945 (0.100)	0.959 (0.074)	0.954 (0.082)	
- Range	0.835 - 1.085	0.839 - 1.108	0.835 - 1.108	
Cingulum hippocampus R				0.948
- Mean (SD)	1.106 (0.143)	1.121 (0.168)	1.115 (0.157)	
- Range	0.917 - 1.303	0.934 - 1.523	0.917 - 1.523	
Cingulum hippocampus L				0.948
- Mean (SD)	1.256 (0.183)	1.200 (0.199)	1.220 (0.192)	
- Range	1.007 - 1.484	0.930 - 1.734	0.930 - 1.734	
Fornix R				0.948
- Mean (SD)	1.055 (0.138)	1.086 (0.163)	1.075 (0.152)	
- Range	0.866 - 1.261	0.884 - 1.408	0.866 - 1.408	
Fornix L				0.965
- Mean (SD)	1.015 (0.136)	1.012 (0.146)	1.013 (0.140)	
- Range	0.851 - 1.246	0.868 - 1.331	0.851 - 1.331	
Superior longitudinal fasciculus R				0.957

	CTR (N=9)	OOM (N=16)	Total (N=25)	p value
- Mean (SD)	0.805 (0.052)	0.801 (0.048)	0.802 (0.048)	
- Range	0.758 - 0.898	0.727 - 0.883	0.727 - 0.898	
Superior longitudinal fasciculus L				0.948
- Mean (SD)	0.781 (0.038)	0.788 (0.045)	0.785 (0.042)	
- Range	0.732 - 0.839	0.728 - 0.865	0.728 - 0.865	
Superior fronto occipital fasciculus R				0.948
- Mean (SD)	0.964 (0.172)	0.917 (0.228)	0.934 (0.207)	
- Range	0.718 - 1.300	0.733 - 1.571	0.718 - 1.571	
Superior fronto occipital fasciculus L				0.948
- Mean (SD)	1.088 (0.246)	1.007 (0.283)	1.036 (0.268)	
- Range	0.836 - 1.504	0.795 - 1.863	0.795 - 1.863	
Uncinate fasciculus R				0.948
- Mean (SD)	0.913 (0.179)	0.929 (0.142)	0.923 (0.153)	
- Range	0.782 - 1.375	0.790 - 1.267	0.782 - 1.375	
Uncinate fasciculus L				0.948
- Mean (SD)	0.846 (0.064)	0.871 (0.082)	0.862 (0.076)	
- Range	0.777 - 0.939	0.784 - 1.136	0.777 - 1.136	
Tapetum R				0.948
- Mean (SD)	2.032 (0.341)	1.948 (0.413)	1.978 (0.383)	
- Range	1.379 - 2.374	1.141 - 2.588	1.141 - 2.588	
Tapetum L				0.948
- Mean (SD)	2.404 (0.471)	2.191 (0.436)	2.268 (0.451)	
- Range	1.674 - 3.197	1.407 - 2.919	1.407 - 3.197	

Radial diffusivity (RD, (10^-3)*(mm^2)/s) (DTI) - Offspring of obese mothers (OOM) vs control subjects - (JHU white-matter tractography atlas) - Uncorrected p-values

	CTR (N=9)	OOM (N=16)	Total (N=25)	p value
Middle cerebellar peduncle				0.556
- Mean (SD)	1.089 (0.156)	1.128 (0.158)	1.114 (0.155)	
- Range	0.906 - 1.447	0.924 - 1.433	0.906 - 1.447	
Pontine crossing tract				0.388
- Mean (SD)	0.797 (0.070)	0.836 (0.121)	0.822 (0.106)	
- Range	0.723 - 0.951	0.698 - 1.162	0.698 - 1.162	
Genu of corpus callosum				0.945
- Mean (SD)	1.370 (0.119)	1.367 (0.116)	1.368 (0.115)	
- Range	1.240 - 1.614	1.231 - 1.664	1.231 - 1.664	
Body of corpus callosum				0.558
- Mean (SD)	1.112 (0.116)	1.139 (0.106)	1.130 (0.108)	
- Range	0.990 - 1.319	1.005 - 1.370	0.990 - 1.370	
Splenium of corpus callosum				0.704
- Mean (SD)	1.046 (0.151)	1.026 (0.099)	1.033 (0.118)	
- Range	0.888 - 1.318	0.886 - 1.287	0.886 - 1.318	
Fornix				0.316
- Mean (SD)	2.495 (0.323)	2.347 (0.359)	2.401 (0.347)	
- Range	2.048 - 2.977	1.853 - 2.857	1.853 - 2.977	
Corticospinal tract R				0.452
- Mean (SD)	0.865 (0.094)	0.899 (0.116)	0.887 (0.108)	
- Range	0.754 - 1.012	0.741 - 1.250	0.741 - 1.250	
Corticospinal tract L				0.522
- Mean (SD)	0.824 (0.108)	0.851 (0.097)	0.841 (0.100)	
- Range	0.736 - 1.037	0.731 - 1.159	0.731 - 1.159	
Medial lemniscus R				0.142
- Mean (SD)	0.846 (0.068)	0.903 (0.099)	0.882 (0.092)	
- Range	0.782 - 0.982	0.812 - 1.210	0.782 - 1.210	
Medial lemniscus L				0.066
- Mean (SD)	0.834 (0.046)	0.882 (0.066)	0.865 (0.063)	
- Range	0.783 - 0.935	0.796 - 1.034	0.783 - 1.034	
Inferior cerebellar peduncle R				0.474
- Mean (SD)	1.136 (0.189)	1.201 (0.226)	1.178 (0.212)	
- Range	0.880 - 1.524	0.927 - 1.639	0.880 - 1.639	
Inferior cerebellar peduncle L				0.392
- Mean (SD)	1.057 (0.148)	1.134 (0.237)	1.106 (0.209)	
- Range	0.954 - 1.406	0.917 - 1.748	0.917 - 1.748	
Superior cerebellar peduncle R				0.580
- Mean (SD)	1.209 (0.241)	1.253 (0.152)	1.237 (0.185)	
- Range	1.000 - 1.829	0.947 - 1.532	0.947 - 1.829	
Superior cerebellar peduncle L				0.510
- Mean (SD)	1.265 (0.175)	1.316 (0.184)	1.298 (0.179)	
- Range	1.010 - 1.622	0.913 - 1.623	0.913 - 1.623	
Cerebral peduncle R				0.770
- Mean (SD)	0.874 (0.071)	0.881 (0.048)	0.879 (0.056)	
- Range	0.794 - 1.038	0.806 - 0.971	0.794 - 1.038	
Cerebral peduncle L				0.829
- Mean (SD)	0.842 (0.060)	0.838 (0.041)	0.839 (0.047)	
- Range	0.788 - 0.985	0.775 - 0.927	0.775 - 0.985	
Anterior limb of internal capsule R				0.948
- Mean (SD)	0.846 (0.087)	0.844 (0.056)	0.845 (0.067)	
- Range	0.773 - 1.055	0.781 - 0.962	0.773 - 1.055	
Anterior limb of internal capsule L				0.751
- Mean (SD)	0.877 (0.110)	0.865 (0.071)	0.869 (0.085)	
- Range	0.775 - 1.112	0.753 - 1.025	0.753 - 1.112	
Posterior limb of internal capsule R				0.562
- Mean (SD)	0.770 (0.050)	0.761 (0.024)	0.764 (0.035)	
- Range	0.689 - 0.846	0.721 - 0.794	0.689 - 0.846	
Posterior limb of internal capsule L				0.754
- Mean (SD)	0.770 (0.060)	0.764 (0.028)	0.766 (0.041)	

	CTR (N=9)	OOM (N=16)	Total (N=25)	p value
- Range	0.694 - 0.882	0.717 - 0.819	0.694 - 0.882	
Retrolenticular part of internal capsule R				0.705
- Mean (SD)	0.934 (0.097)	0.920 (0.083)	0.925 (0.086)	
- Range	0.827 - 1.105	0.780 - 1.062	0.780 - 1.105	
Retrolenticular part of internal capsule L				0.894
- Mean (SD)	0.936 (0.111)	0.931 (0.078)	0.932 (0.089)	
- Range	0.837 - 1.171	0.814 - 1.074	0.814 - 1.171	
Anterior corona radiata R				0.652
- Mean (SD)	0.940 (0.079)	0.921 (0.112)	0.928 (0.100)	
- Range	0.835 - 1.070	0.823 - 1.248	0.823 - 1.248	
Anterior corona radiata L				0.741
- Mean (SD)	0.927 (0.066)	0.915 (0.099)	0.919 (0.087)	
- Range	0.841 - 1.030	0.821 - 1.215	0.821 - 1.215	
Superior corona radiata R				0.546
- Mean (SD)	0.821 (0.059)	0.804 (0.072)	0.810 (0.067)	
- Range	0.740 - 0.927	0.735 - 0.946	0.735 - 0.946	
Superior corona radiata L				0.808
- Mean (SD)	0.835 (0.068)	0.826 (0.094)	0.830 (0.084)	
- Range	0.743 - 0.949	0.733 - 1.099	0.733 - 1.099	
Posterior corona radiata R				0.493
- Mean (SD)	0.967 (0.131)	0.930 (0.123)	0.943 (0.125)	
- Range	0.818 - 1.163	0.780 - 1.146	0.780 - 1.163	
Posterior corona radiata L				0.350
- Mean (SD)	1.028 (0.189)	0.964 (0.145)	0.987 (0.161)	
- Range	0.871 - 1.331	0.800 - 1.328	0.800 - 1.331	
Posterior thalamic radiation R				0.930
- Mean (SD)	1.002 (0.097)	0.996 (0.167)	0.998 (0.143)	
- Range	0.857 - 1.174	0.828 - 1.510	0.828 - 1.510	
Posterior thalamic radiation L				0.568
- Mean (SD)	1.137 (0.115)	1.099 (0.175)	1.113 (0.155)	
- Range	0.993 - 1.352	0.935 - 1.589	0.935 - 1.589	
Sagittal stratum R				0.727
- Mean (SD)	0.986 (0.089)	1.003 (0.124)	0.997 (0.111)	
- Range	0.833 - 1.078	0.844 - 1.335	0.833 - 1.335	
Sagittal stratum L				0.750
- Mean (SD)	1.074 (0.143)	1.057 (0.105)	1.063 (0.118)	
- Range	0.903 - 1.379	0.895 - 1.279	0.895 - 1.379	
External capsule R				0.820
- Mean (SD)	0.842 (0.067)	0.848 (0.057)	0.846 (0.059)	
- Range	0.764 - 0.953	0.753 - 0.955	0.753 - 0.955	
External capsule L				0.642
- Mean (SD)	0.822 (0.048)	0.832 (0.052)	0.829 (0.049)	
- Range	0.766 - 0.893	0.771 - 0.945	0.766 - 0.945	
Cingulum cingulate gyrus R				0.515
- Mean (SD)	0.950 (0.134)	0.924 (0.058)	0.934 (0.090)	
- Range	0.802 - 1.199	0.819 - 1.043	0.802 - 1.199	
Cingulum cingulate gyrus L				0.680
- Mean (SD)	0.945 (0.100)	0.959 (0.074)	0.954 (0.082)	
- Range	0.835 - 1.085	0.839 - 1.108	0.835 - 1.108	
Cingulum hippocampus R				0.822
- Mean (SD)	1.106 (0.143)	1.121 (0.168)	1.115 (0.157)	
- Range	0.917 - 1.303	0.934 - 1.523	0.917 - 1.523	
Cingulum hippocampus L				0.495
- Mean (SD)	1.256 (0.183)	1.200 (0.199)	1.220 (0.192)	
- Range	1.007 - 1.484	0.930 - 1.734	0.930 - 1.734	
Fornix R				0.638
- Mean (SD)	1.055 (0.138)	1.086 (0.163)	1.075 (0.152)	
- Range	0.866 - 1.261	0.884 - 1.408	0.866 - 1.408	
Fornix L				0.965
- Mean (SD)	1.015 (0.136)	1.012 (0.146)	1.013 (0.140)	
- Range	0.851 - 1.246	0.868 - 1.331	0.851 - 1.331	
Superior longitudinal fasciculus R				0.857

	CTR (N=9)	OOM (N=16)	Total (N=25)	p value
- Mean (SD)	0.805 (0.052)	0.801 (0.048)	0.802 (0.048)	
- Range	0.758 - 0.898	0.727 - 0.883	0.727 - 0.898	
Superior longitudinal fasciculus L				0.715
- Mean (SD)	0.781 (0.038)	0.788 (0.045)	0.785 (0.042)	
- Range	0.732 - 0.839	0.728 - 0.865	0.728 - 0.865	
Superior fronto occipital fasciculus R				0.599
- Mean (SD)	0.964 (0.172)	0.917 (0.228)	0.934 (0.207)	
- Range	0.718 - 1.300	0.733 - 1.571	0.718 - 1.571	
Superior fronto occipital fasciculus L				0.480
- Mean (SD)	1.088 (0.246)	1.007 (0.283)	1.036 (0.268)	
- Range	0.836 - 1.504	0.795 - 1.863	0.795 - 1.863	
Uncinate fasciculus R				0.804
- Mean (SD)	0.913 (0.179)	0.929 (0.142)	0.923 (0.153)	
- Range	0.782 - 1.375	0.790 - 1.267	0.782 - 1.375	
Uncinate fasciculus L				0.431
- Mean (SD)	0.846 (0.064)	0.871 (0.082)	0.862 (0.076)	
- Range	0.777 - 0.939	0.784 - 1.136	0.777 - 1.136	
Tapetum R				0.608
- Mean (SD)	2.032 (0.341)	1.948 (0.413)	1.978 (0.383)	
- Range	1.379 - 2.374	1.141 - 2.588	1.141 - 2.588	
Tapetum L				0.267
- Mean (SD)	2.404 (0.471)	2.191 (0.436)	2.268 (0.451)	
- Range	1.674 - 3.197	1.407 - 2.919	1.407 - 3.197	

Radial diffusivity (RD, (10^-3)*(mm^2)/s) (DTI) - Offspring of obese mothers (OOM) vs control subjects - (JHU white-matter tractography atlas) - FDR corrected p-values

	CTR (N=9)	OOM (N=16)	Total (N=25)	p value
Middle cerebellar peduncle				0.948
- Mean (SD)	1.089 (0.156)	1.128 (0.158)	1.114 (0.155)	
- Range	0.906 - 1.447	0.924 - 1.433	0.906 - 1.447	
Pontine crossing tract				0.948
- Mean (SD)	0.797 (0.070)	0.836 (0.121)	0.822 (0.106)	
- Range	0.723 - 0.951	0.698 - 1.162	0.698 - 1.162	
Genu of corpus callosum				0.965
- Mean (SD)	1.370 (0.119)	1.367 (0.116)	1.368 (0.115)	
- Range	1.240 - 1.614	1.231 - 1.664	1.231 - 1.664	
Body of corpus callosum				0.948
- Mean (SD)	1.112 (0.116)	1.139 (0.106)	1.130 (0.108)	
- Range	0.990 - 1.319	1.005 - 1.370	0.990 - 1.370	
Splenium of corpus callosum				0.948
- Mean (SD)	1.046 (0.151)	1.026 (0.099)	1.033 (0.118)	
- Range	0.888 - 1.318	0.886 - 1.287	0.886 - 1.318	
Fornix				0.948
- Mean (SD)	2.495 (0.323)	2.347 (0.359)	2.401 (0.347)	
- Range	2.048 - 2.977	1.853 - 2.857	1.853 - 2.977	
Corticospinal tract R				0.948
- Mean (SD)	0.865 (0.094)	0.899 (0.116)	0.887 (0.108)	
- Range	0.754 - 1.012	0.741 - 1.250	0.741 - 1.250	
Corticospinal tract L				0.948
- Mean (SD)	0.824 (0.108)	0.851 (0.097)	0.841 (0.100)	
- Range	0.736 - 1.037	0.731 - 1.159	0.731 - 1.159	
Medial lemniscus R				0.948
- Mean (SD)	0.846 (0.068)	0.903 (0.099)	0.882 (0.092)	
- Range	0.782 - 0.982	0.812 - 1.210	0.782 - 1.210	
Medial lemniscus L				0.948
- Mean (SD)	0.834 (0.046)	0.882 (0.066)	0.865 (0.063)	
- Range	0.783 - 0.935	0.796 - 1.034	0.783 - 1.034	
Inferior cerebellar peduncle R				0.948
- Mean (SD)	1.136 (0.189)	1.201 (0.226)	1.178 (0.212)	
- Range	0.880 - 1.524	0.927 - 1.639	0.880 - 1.639	
Inferior cerebellar peduncle L				0.948
- Mean (SD)	1.057 (0.148)	1.134 (0.237)	1.106 (0.209)	
- Range	0.954 - 1.406	0.917 - 1.748	0.917 - 1.748	
Superior cerebellar peduncle R				0.948
- Mean (SD)	1.209 (0.241)	1.253 (0.152)	1.237 (0.185)	
- Range	1.000 - 1.829	0.947 - 1.532	0.947 - 1.829	
Superior cerebellar peduncle L				0.948
- Mean (SD)	1.265 (0.175)	1.316 (0.184)	1.298 (0.179)	
- Range	1.010 - 1.622	0.913 - 1.623	0.913 - 1.623	
Cerebral peduncle R				0.948
- Mean (SD)	0.874 (0.071)	0.881 (0.048)	0.879 (0.056)	
- Range	0.794 - 1.038	0.806 - 0.971	0.794 - 1.038	
Cerebral peduncle L				0.948
- Mean (SD)	0.842 (0.060)	0.838 (0.041)	0.839 (0.047)	
- Range	0.788 - 0.985	0.775 - 0.927	0.775 - 0.985	
Anterior limb of internal capsule R				0.965
- Mean (SD)	0.846 (0.087)	0.844 (0.056)	0.845 (0.067)	
- Range	0.773 - 1.055	0.781 - 0.962	0.773 - 1.055	
Anterior limb of internal capsule L				0.948
- Mean (SD)	0.877 (0.110)	0.865 (0.071)	0.869 (0.085)	
- Range	0.775 - 1.112	0.753 - 1.025	0.753 - 1.112	
Posterior limb of internal capsule R				0.948
- Mean (SD)	0.770 (0.050)	0.761 (0.024)	0.764 (0.035)	
- Range	0.689 - 0.846	0.721 - 0.794	0.689 - 0.846	
Posterior limb of internal capsule L				0.948
- Mean (SD)	0.770 (0.060)	0.764 (0.028)	0.766 (0.041)	

	CTR (N=9)	OOM (N=16)	Total (N=25)	p value
- Range	0.694 - 0.882	0.717 - 0.819	0.694 - 0.882	
Retrolenticular part of internal capsule R				0.948
- Mean (SD)	0.934 (0.097)	0.920 (0.083)	0.925 (0.086)	
- Range	0.827 - 1.105	0.780 - 1.062	0.780 - 1.105	
Retrolenticular part of internal capsule L				0.965
- Mean (SD)	0.936 (0.111)	0.931 (0.078)	0.932 (0.089)	
- Range	0.837 - 1.171	0.814 - 1.074	0.814 - 1.171	
Anterior corona radiata R				0.948
- Mean (SD)	0.940 (0.079)	0.921 (0.112)	0.928 (0.100)	
- Range	0.835 - 1.070	0.823 - 1.248	0.823 - 1.248	
Anterior corona radiata L				0.948
- Mean (SD)	0.927 (0.066)	0.915 (0.099)	0.919 (0.087)	
- Range	0.841 - 1.030	0.821 - 1.215	0.821 - 1.215	
Superior corona radiata R				0.948
- Mean (SD)	0.821 (0.059)	0.804 (0.072)	0.810 (0.067)	
- Range	0.740 - 0.927	0.735 - 0.946	0.735 - 0.946	
Superior corona radiata L				0.948
- Mean (SD)	0.835 (0.068)	0.826 (0.094)	0.830 (0.084)	
- Range	0.743 - 0.949	0.733 - 1.099	0.733 - 1.099	
Posterior corona radiata R				0.948
- Mean (SD)	0.967 (0.131)	0.930 (0.123)	0.943 (0.125)	
- Range	0.818 - 1.163	0.780 - 1.146	0.780 - 1.163	
Posterior corona radiata L				0.948
- Mean (SD)	1.028 (0.189)	0.964 (0.145)	0.987 (0.161)	
- Range	0.871 - 1.331	0.800 - 1.328	0.800 - 1.331	
Posterior thalamic radiation R				0.965
- Mean (SD)	1.002 (0.097)	0.996 (0.167)	0.998 (0.143)	
- Range	0.857 - 1.174	0.828 - 1.510	0.828 - 1.510	
Posterior thalamic radiation L				0.948
- Mean (SD)	1.137 (0.115)	1.099 (0.175)	1.113 (0.155)	
- Range	0.993 - 1.352	0.935 - 1.589	0.935 - 1.589	
Sagittal stratum R				0.948
- Mean (SD)	0.986 (0.089)	1.003 (0.124)	0.997 (0.111)	
- Range	0.833 - 1.078	0.844 - 1.335	0.833 - 1.335	
Sagittal stratum L				0.948
- Mean (SD)	1.074 (0.143)	1.057 (0.105)	1.063 (0.118)	
- Range	0.903 - 1.379	0.895 - 1.279	0.895 - 1.379	
External capsule R				0.948
- Mean (SD)	0.842 (0.067)	0.848 (0.057)	0.846 (0.059)	
- Range	0.764 - 0.953	0.753 - 0.955	0.753 - 0.955	
External capsule L				0.948
- Mean (SD)	0.822 (0.048)	0.832 (0.052)	0.829 (0.049)	
- Range	0.766 - 0.893	0.771 - 0.945	0.766 - 0.945	
Cingulum cingulate gyrus R				0.948
- Mean (SD)	0.950 (0.134)	0.924 (0.058)	0.934 (0.090)	
- Range	0.802 - 1.199	0.819 - 1.043	0.802 - 1.199	
Cingulum cingulate gyrus L				0.948
- Mean (SD)	0.945 (0.100)	0.959 (0.074)	0.954 (0.082)	
- Range	0.835 - 1.085	0.839 - 1.108	0.835 - 1.108	
Cingulum hippocampus R				0.948
- Mean (SD)	1.106 (0.143)	1.121 (0.168)	1.115 (0.157)	
- Range	0.917 - 1.303	0.934 - 1.523	0.917 - 1.523	
Cingulum hippocampus L				0.948
- Mean (SD)	1.256 (0.183)	1.200 (0.199)	1.220 (0.192)	
- Range	1.007 - 1.484	0.930 - 1.734	0.930 - 1.734	
Fornix R				0.948
- Mean (SD)	1.055 (0.138)	1.086 (0.163)	1.075 (0.152)	
- Range	0.866 - 1.261	0.884 - 1.408	0.866 - 1.408	
Fornix L				0.965
- Mean (SD)	1.015 (0.136)	1.012 (0.146)	1.013 (0.140)	
- Range	0.851 - 1.246	0.868 - 1.331	0.851 - 1.331	
Superior longitudinal fasciculus R				0.957

	CTR (N=9)	OOM (N=16)	Total (N=25)	p value
- Mean (SD)	0.805 (0.052)	0.801 (0.048)	0.802 (0.048)	
- Range	0.758 - 0.898	0.727 - 0.883	0.727 - 0.898	
Superior longitudinal fasciculus L				0.948
- Mean (SD)	0.781 (0.038)	0.788 (0.045)	0.785 (0.042)	
- Range	0.732 - 0.839	0.728 - 0.865	0.728 - 0.865	
Superior fronto occipital fasciculus R				0.948
- Mean (SD)	0.964 (0.172)	0.917 (0.228)	0.934 (0.207)	
- Range	0.718 - 1.300	0.733 - 1.571	0.718 - 1.571	
Superior fronto occipital fasciculus L				0.948
- Mean (SD)	1.088 (0.246)	1.007 (0.283)	1.036 (0.268)	
- Range	0.836 - 1.504	0.795 - 1.863	0.795 - 1.863	
Uncinate fasciculus R				0.948
- Mean (SD)	0.913 (0.179)	0.929 (0.142)	0.923 (0.153)	
- Range	0.782 - 1.375	0.790 - 1.267	0.782 - 1.375	
Uncinate fasciculus L				0.948
- Mean (SD)	0.846 (0.064)	0.871 (0.082)	0.862 (0.076)	
- Range	0.777 - 0.939	0.784 - 1.136	0.777 - 1.136	
Tapetum R				0.948
- Mean (SD)	2.032 (0.341)	1.948 (0.413)	1.978 (0.383)	
- Range	1.379 - 2.374	1.141 - 2.588	1.141 - 2.588	
Tapetum L				0.948
- Mean (SD)	2.404 (0.471)	2.191 (0.436)	2.268 (0.451)	
- Range	1.674 - 3.197	1.407 - 2.919	1.407 - 3.197	

Fractional anisotropy (FA) (DTI) - Frail (OOM+OLM) subjects pre vs post (paired tests) - (JHU white-matter tractography atlas) - Uncorrected p-values

	Post (N=31)	Pre (N=31)	Difference (N=31)	p value
Middle cerebellar peduncle				0.966
- Mean (SD)	0.403 (0.043)	0.403 (0.057)	0.001 (0.073)	
- Range	0.290 - 0.454	0.262 - 0.480	-0.152 - 0.153	
Pontine crossing tract				0.441
- Mean (SD)	0.396 (0.037)	0.403 (0.037)	0.007 (0.050)	
- Range	0.350 - 0.502	0.337 - 0.483	-0.142 - 0.116	
Genu of corpus callosum				0.615
- Mean (SD)	0.436 (0.023)	0.435 (0.026)	-0.001 (0.014)	
- Range	0.385 - 0.478	0.383 - 0.498	-0.043 - 0.039	
Body of corpus callosum				0.956
- Mean (SD)	0.486 (0.028)	0.486 (0.033)	-0.000 (0.016)	
- Range	0.440 - 0.539	0.426 - 0.553	-0.041 - 0.044	
Splenium of corpus callosum				0.648
- Mean (SD)	0.589 (0.028)	0.588 (0.030)	-0.001 (0.018)	
- Range	0.531 - 0.653	0.533 - 0.669	-0.035 - 0.043	
Fornix				0.042
- Mean (SD)	0.241 (0.042)	0.251 (0.037)	0.010 (0.027)	
- Range	0.179 - 0.340	0.184 - 0.332	-0.063 - 0.078	
Corticospinal tract R				0.307
- Mean (SD)	0.468 (0.038)	0.477 (0.041)	0.008 (0.045)	
- Range	0.389 - 0.553	0.403 - 0.562	-0.072 - 0.095	
Corticospinal tract L				0.848
- Mean (SD)	0.484 (0.044)	0.483 (0.042)	-0.002 (0.054)	
- Range	0.360 - 0.599	0.418 - 0.597	-0.125 - 0.130	
Medial lemniscus R				0.683
- Mean (SD)	0.450 (0.048)	0.446 (0.049)	-0.005 (0.062)	
- Range	0.368 - 0.556	0.335 - 0.560	-0.136 - 0.135	
Medial lemniscus L				0.951
- Mean (SD)	0.454 (0.051)	0.453 (0.057)	-0.001 (0.072)	
- Range	0.380 - 0.569	0.353 - 0.598	-0.143 - 0.163	
Inferior cerebellar peduncle R				0.327
- Mean (SD)	0.344 (0.036)	0.355 (0.044)	0.011 (0.059)	
- Range	0.298 - 0.460	0.270 - 0.437	-0.139 - 0.113	
Inferior cerebellar peduncle L				0.671
- Mean (SD)	0.356 (0.039)	0.352 (0.045)	-0.004 (0.056)	
- Range	0.287 - 0.445	0.230 - 0.422	-0.156 - 0.117	
Superior cerebellar peduncle R				0.885
- Mean (SD)	0.465 (0.050)	0.462 (0.068)	-0.002 (0.093)	
- Range	0.350 - 0.528	0.298 - 0.568	-0.206 - 0.181	
Superior cerebellar peduncle L				0.874
- Mean (SD)	0.442 (0.046)	0.440 (0.052)	-0.002 (0.074)	
- Range	0.311 - 0.500	0.308 - 0.519	-0.154 - 0.148	
Cerebral peduncle R				0.372
- Mean (SD)	0.562 (0.025)	0.559 (0.028)	-0.003 (0.021)	
- Range	0.510 - 0.622	0.495 - 0.625	-0.043 - 0.062	
Cerebral peduncle L				0.525
- Mean (SD)	0.574 (0.025)	0.571 (0.029)	-0.003 (0.023)	
- Range	0.517 - 0.632	0.491 - 0.615	-0.058 - 0.047	
Anterior limb of internal capsule R				0.094
- Mean (SD)	0.434 (0.027)	0.430 (0.031)	-0.004 (0.014)	
- Range	0.360 - 0.476	0.341 - 0.482	-0.042 - 0.020	
Anterior limb of internal capsule L				0.557
- Mean (SD)	0.441 (0.025)	0.439 (0.031)	-0.002 (0.021)	
- Range	0.400 - 0.490	0.389 - 0.491	-0.064 - 0.038	
Posterior limb of internal capsule R				0.275
- Mean (SD)	0.528 (0.020)	0.525 (0.024)	-0.003 (0.014)	
- Range	0.489 - 0.566	0.472 - 0.559	-0.038 - 0.029	
Posterior limb of internal capsule L				0.951
- Mean (SD)	0.535 (0.019)	0.535 (0.021)	0.000 (0.015)	
- Range	0.499 - 0.585	0.478 - 0.573	-0.038 - 0.024	

	Post (N=31)	Pre (N=31)	Difference (N=31)	p value
Retrolenticular part of internal capsule R				0.077
- Mean (SD)	0.437 (0.021)	0.432 (0.023)	-0.005 (0.015)	
- Range	0.402 - 0.477	0.389 - 0.481	-0.030 - 0.025	
Retrolenticular part of internal capsule L				0.512
- Mean (SD)	0.444 (0.024)	0.446 (0.027)	0.003 (0.022)	
- Range	0.406 - 0.489	0.402 - 0.503	-0.044 - 0.063	
Anterior corona radiata R				0.348
- Mean (SD)	0.332 (0.025)	0.330 (0.030)	-0.002 (0.013)	
- Range	0.272 - 0.378	0.263 - 0.383	-0.028 - 0.037	
Anterior corona radiata L				0.870
- Mean (SD)	0.333 (0.025)	0.332 (0.029)	-0.001 (0.017)	
- Range	0.275 - 0.389	0.270 - 0.382	-0.034 - 0.040	
Superior corona radiata R				0.431
- Mean (SD)	0.399 (0.033)	0.397 (0.032)	-0.002 (0.012)	
- Range	0.334 - 0.473	0.315 - 0.451	-0.022 - 0.024	
Superior corona radiata L				0.416
- Mean (SD)	0.401 (0.033)	0.402 (0.036)	0.002 (0.012)	
- Range	0.314 - 0.485	0.311 - 0.478	-0.029 - 0.041	
Posterior corona radiata R				0.173
- Mean (SD)	0.411 (0.035)	0.407 (0.036)	-0.004 (0.017)	
- Range	0.354 - 0.505	0.331 - 0.480	-0.033 - 0.047	
Posterior corona radiata L				0.123
- Mean (SD)	0.395 (0.038)	0.399 (0.039)	0.005 (0.017)	
- Range	0.317 - 0.490	0.308 - 0.491	-0.048 - 0.040	
Posterior thalamic radiation R				0.160
- Mean (SD)	0.455 (0.031)	0.451 (0.032)	-0.004 (0.014)	
- Range	0.370 - 0.502	0.372 - 0.494	-0.030 - 0.027	
Posterior thalamic radiation L				0.453
- Mean (SD)	0.442 (0.026)	0.444 (0.028)	0.002 (0.017)	
- Range	0.379 - 0.482	0.388 - 0.497	-0.035 - 0.030	
Sagittal stratum R				0.618
- Mean (SD)	0.422 (0.021)	0.424 (0.021)	0.002 (0.018)	
- Range	0.379 - 0.467	0.391 - 0.457	-0.025 - 0.035	
Sagittal stratum L				0.738
- Mean (SD)	0.390 (0.017)	0.389 (0.022)	-0.001 (0.022)	
- Range	0.357 - 0.433	0.346 - 0.445	-0.039 - 0.052	
External capsule R				0.796
- Mean (SD)	0.324 (0.019)	0.323 (0.020)	-0.001 (0.017)	
- Range	0.288 - 0.353	0.280 - 0.365	-0.038 - 0.037	
External capsule L				0.328
- Mean (SD)	0.332 (0.018)	0.335 (0.022)	0.003 (0.015)	
- Range	0.301 - 0.369	0.302 - 0.397	-0.051 - 0.037	
Cingulum cingulate gyrus R				0.481
- Mean (SD)	0.355 (0.024)	0.353 (0.022)	-0.002 (0.015)	
- Range	0.315 - 0.411	0.303 - 0.401	-0.030 - 0.035	
Cingulum cingulate gyrus L				0.975
- Mean (SD)	0.366 (0.026)	0.366 (0.027)	0.000 (0.012)	
- Range	0.315 - 0.418	0.310 - 0.421	-0.028 - 0.021	
Cingulum hippocampus R				0.574
- Mean (SD)	0.284 (0.029)	0.280 (0.035)	-0.004 (0.036)	
- Range	0.235 - 0.377	0.213 - 0.371	-0.086 - 0.048	
Cingulum hippocampus L				0.389
- Mean (SD)	0.281 (0.031)	0.275 (0.030)	-0.006 (0.037)	
- Range	0.202 - 0.377	0.204 - 0.326	-0.105 - 0.068	
Fornix R				0.332
- Mean (SD)	0.357 (0.022)	0.361 (0.030)	0.004 (0.024)	
- Range	0.312 - 0.408	0.306 - 0.426	-0.041 - 0.065	
Fornix L				0.454
- Mean (SD)	0.382 (0.032)	0.379 (0.033)	-0.004 (0.026)	
- Range	0.324 - 0.443	0.326 - 0.438	-0.079 - 0.042	
Superior longitudinal fasciculus R				0.770
- Mean (SD)	0.384 (0.026)	0.384 (0.024)	-0.001 (0.015)	

	Post (N=31)	Pre (N=31)	Difference (N=31)	p value
- Range	0.326 - 0.444	0.346 - 0.426	-0.031 - 0.034	
Superior longitudinal fasciculus L				0.993
- Mean (SD)	0.387 (0.028)	0.387 (0.028)	-0.000 (0.016)	
- Range	0.338 - 0.440	0.320 - 0.432	-0.052 - 0.020	
Superior fronto occipital fasciculus R				0.317
- Mean (SD)	0.351 (0.037)	0.345 (0.044)	-0.006 (0.031)	
- Range	0.256 - 0.405	0.248 - 0.441	-0.081 - 0.062	
Superior fronto occipital fasciculus L				0.512
- Mean (SD)	0.332 (0.041)	0.329 (0.038)	-0.003 (0.024)	
- Range	0.254 - 0.410	0.243 - 0.383	-0.060 - 0.065	
Uncinate fasciculus R				0.145
- Mean (SD)	0.379 (0.042)	0.390 (0.034)	0.011 (0.040)	
- Range	0.247 - 0.471	0.328 - 0.462	-0.092 - 0.128	
Uncinate fasciculus L				0.287
- Mean (SD)	0.382 (0.041)	0.389 (0.041)	0.007 (0.036)	
- Range	0.305 - 0.457	0.319 - 0.452	-0.054 - 0.093	
Tapetum R				0.279
- Mean (SD)	0.330 (0.031)	0.335 (0.033)	0.005 (0.024)	
- Range	0.283 - 0.396	0.264 - 0.387	-0.039 - 0.048	
Tapetum L				0.987
- Mean (SD)	0.297 (0.027)	0.297 (0.030)	-0.000 (0.023)	
- Range	0.254 - 0.358	0.228 - 0.372	-0.047 - 0.049	

Fractional anisotropy (FA) (DTI) - Frail (OOM+OLM) subjects pre vs post (paired tests) - (JHU white-matter tractography atlas) - FDR corrected p-values

	Post (N=31)	Pre (N=31)	Difference (N=31)	p value
Middle cerebellar peduncle				0.993
- Mean (SD)	0.403 (0.043)	0.403 (0.057)	0.001 (0.073)	
- Range	0.290 - 0.454	0.262 - 0.480	-0.152 - 0.153	
Pontine crossing tract				0.933
- Mean (SD)	0.396 (0.037)	0.403 (0.037)	0.007 (0.050)	
- Range	0.350 - 0.502	0.337 - 0.483	-0.142 - 0.116	
Genu of corpus callosum				0.958
- Mean (SD)	0.436 (0.023)	0.435 (0.026)	-0.001 (0.014)	
- Range	0.385 - 0.478	0.383 - 0.498	-0.043 - 0.039	
Body of corpus callosum				0.993
- Mean (SD)	0.486 (0.028)	0.486 (0.033)	-0.000 (0.016)	
- Range	0.440 - 0.539	0.426 - 0.553	-0.041 - 0.044	
Splenium of corpus callosum				0.964
- Mean (SD)	0.589 (0.028)	0.588 (0.030)	-0.001 (0.018)	
- Range	0.531 - 0.653	0.533 - 0.669	-0.035 - 0.043	
Fornix				0.933
- Mean (SD)	0.241 (0.042)	0.251 (0.037)	0.010 (0.027)	
- Range	0.179 - 0.340	0.184 - 0.332	-0.063 - 0.078	
Corticospinal tract R				0.933
- Mean (SD)	0.468 (0.038)	0.477 (0.041)	0.008 (0.045)	
- Range	0.389 - 0.553	0.403 - 0.562	-0.072 - 0.095	
Corticospinal tract L				0.993
- Mean (SD)	0.484 (0.044)	0.483 (0.042)	-0.002 (0.054)	
- Range	0.360 - 0.599	0.418 - 0.597	-0.125 - 0.130	
Medial lemniscus R				0.964
- Mean (SD)	0.450 (0.048)	0.446 (0.049)	-0.005 (0.062)	
- Range	0.368 - 0.556	0.335 - 0.560	-0.136 - 0.135	
Medial lemniscus L				0.993
- Mean (SD)	0.454 (0.051)	0.453 (0.057)	-0.001 (0.072)	
- Range	0.380 - 0.569	0.353 - 0.598	-0.143 - 0.163	
Inferior cerebellar peduncle R				0.933
- Mean (SD)	0.344 (0.036)	0.355 (0.044)	0.011 (0.059)	
- Range	0.298 - 0.460	0.270 - 0.437	-0.139 - 0.113	
Inferior cerebellar peduncle L				0.964
- Mean (SD)	0.356 (0.039)	0.352 (0.045)	-0.004 (0.056)	
- Range	0.287 - 0.445	0.230 - 0.422	-0.156 - 0.117	
Superior cerebellar peduncle R				0.993
- Mean (SD)	0.465 (0.050)	0.462 (0.068)	-0.002 (0.093)	
- Range	0.350 - 0.528	0.298 - 0.568	-0.206 - 0.181	
Superior cerebellar peduncle L				0.993
- Mean (SD)	0.442 (0.046)	0.440 (0.052)	-0.002 (0.074)	
- Range	0.311 - 0.500	0.308 - 0.519	-0.154 - 0.148	
Cerebral peduncle R				0.933
- Mean (SD)	0.562 (0.025)	0.559 (0.028)	-0.003 (0.021)	
- Range	0.510 - 0.622	0.495 - 0.625	-0.043 - 0.062	
Cerebral peduncle L				0.933
- Mean (SD)	0.574 (0.025)	0.571 (0.029)	-0.003 (0.023)	
- Range	0.517 - 0.632	0.491 - 0.615	-0.058 - 0.047	
Anterior limb of internal capsule R				0.933
- Mean (SD)	0.434 (0.027)	0.430 (0.031)	-0.004 (0.014)	
- Range	0.360 - 0.476	0.341 - 0.482	-0.042 - 0.020	
Anterior limb of internal capsule L				0.949
- Mean (SD)	0.441 (0.025)	0.439 (0.031)	-0.002 (0.021)	
- Range	0.400 - 0.490	0.389 - 0.491	-0.064 - 0.038	
Posterior limb of internal capsule R				0.933
- Mean (SD)	0.528 (0.020)	0.525 (0.024)	-0.003 (0.014)	
- Range	0.489 - 0.566	0.472 - 0.559	-0.038 - 0.029	
Posterior limb of internal capsule L				0.993
- Mean (SD)	0.535 (0.019)	0.535 (0.021)	0.000 (0.015)	
- Range	0.499 - 0.585	0.478 - 0.573	-0.038 - 0.024	

	Post (N=31)	Pre (N=31)	Difference (N=31)	p value
Retrolenticular part of internal capsule R				0.933
- Mean (SD)	0.437 (0.021)	0.432 (0.023)	-0.005 (0.015)	
- Range	0.402 - 0.477	0.389 - 0.481	-0.030 - 0.025	
Retrolenticular part of internal capsule L				0.933
- Mean (SD)	0.444 (0.024)	0.446 (0.027)	0.003 (0.022)	
- Range	0.406 - 0.489	0.402 - 0.503	-0.044 - 0.063	
Anterior corona radiata R				0.933
- Mean (SD)	0.332 (0.025)	0.330 (0.030)	-0.002 (0.013)	
- Range	0.272 - 0.378	0.263 - 0.383	-0.028 - 0.037	
Anterior corona radiata L				0.993
- Mean (SD)	0.333 (0.025)	0.332 (0.029)	-0.001 (0.017)	
- Range	0.275 - 0.389	0.270 - 0.382	-0.034 - 0.040	
Superior corona radiata R				0.933
- Mean (SD)	0.399 (0.033)	0.397 (0.032)	-0.002 (0.012)	
- Range	0.334 - 0.473	0.315 - 0.451	-0.022 - 0.024	
Superior corona radiata L				0.933
- Mean (SD)	0.401 (0.033)	0.402 (0.036)	0.002 (0.012)	
- Range	0.314 - 0.485	0.311 - 0.478	-0.029 - 0.041	
Posterior corona radiata R				0.933
- Mean (SD)	0.411 (0.035)	0.407 (0.036)	-0.004 (0.017)	
- Range	0.354 - 0.505	0.331 - 0.480	-0.033 - 0.047	
Posterior corona radiata L				0.933
- Mean (SD)	0.395 (0.038)	0.399 (0.039)	0.005 (0.017)	
- Range	0.317 - 0.490	0.308 - 0.491	-0.048 - 0.040	
Posterior thalamic radiation R				0.933
- Mean (SD)	0.455 (0.031)	0.451 (0.032)	-0.004 (0.014)	
- Range	0.370 - 0.502	0.372 - 0.494	-0.030 - 0.027	
Posterior thalamic radiation L				0.933
- Mean (SD)	0.442 (0.026)	0.444 (0.028)	0.002 (0.017)	
- Range	0.379 - 0.482	0.388 - 0.497	-0.035 - 0.030	
Sagittal stratum R				0.958
- Mean (SD)	0.422 (0.021)	0.424 (0.021)	0.002 (0.018)	
- Range	0.379 - 0.467	0.391 - 0.457	-0.025 - 0.035	
Sagittal stratum L				0.993
- Mean (SD)	0.390 (0.017)	0.389 (0.022)	-0.001 (0.022)	
- Range	0.357 - 0.433	0.346 - 0.445	-0.039 - 0.052	
External capsule R				0.993
- Mean (SD)	0.324 (0.019)	0.323 (0.020)	-0.001 (0.017)	
- Range	0.288 - 0.353	0.280 - 0.365	-0.038 - 0.037	
External capsule L				0.933
- Mean (SD)	0.332 (0.018)	0.335 (0.022)	0.003 (0.015)	
- Range	0.301 - 0.369	0.302 - 0.397	-0.051 - 0.037	
Cingulum cingulate gyrus R				0.933
- Mean (SD)	0.355 (0.024)	0.353 (0.022)	-0.002 (0.015)	
- Range	0.315 - 0.411	0.303 - 0.401	-0.030 - 0.035	
Cingulum cingulate gyrus L				0.993
- Mean (SD)	0.366 (0.026)	0.366 (0.027)	0.000 (0.012)	
- Range	0.315 - 0.418	0.310 - 0.421	-0.028 - 0.021	
Cingulum hippocampus R				0.949
- Mean (SD)	0.284 (0.029)	0.280 (0.035)	-0.004 (0.036)	
- Range	0.235 - 0.377	0.213 - 0.371	-0.086 - 0.048	
Cingulum hippocampus L				0.933
- Mean (SD)	0.281 (0.031)	0.275 (0.030)	-0.006 (0.037)	
- Range	0.202 - 0.377	0.204 - 0.326	-0.105 - 0.068	
Fornix R				0.933
- Mean (SD)	0.357 (0.022)	0.361 (0.030)	0.004 (0.024)	
- Range	0.312 - 0.408	0.306 - 0.426	-0.041 - 0.065	
Fornix L				0.933
- Mean (SD)	0.382 (0.032)	0.379 (0.033)	-0.004 (0.026)	
- Range	0.324 - 0.443	0.326 - 0.438	-0.079 - 0.042	
Superior longitudinal fasciculus R				0.993
- Mean (SD)	0.384 (0.026)	0.384 (0.024)	-0.001 (0.015)	

	Post (N=31)	Pre (N=31)	Difference (N=31)	p value
- Range	0.326 - 0.444	0.346 - 0.426	-0.031 - 0.034	
Superior longitudinal fasciculus L				0.993
- Mean (SD)	0.387 (0.028)	0.387 (0.028)	-0.000 (0.016)	
- Range	0.338 - 0.440	0.320 - 0.432	-0.052 - 0.020	
Superior fronto occipital fasciculus R				0.933
- Mean (SD)	0.351 (0.037)	0.345 (0.044)	-0.006 (0.031)	
- Range	0.256 - 0.405	0.248 - 0.441	-0.081 - 0.062	
Superior fronto occipital fasciculus L				0.933
- Mean (SD)	0.332 (0.041)	0.329 (0.038)	-0.003 (0.024)	
- Range	0.254 - 0.410	0.243 - 0.383	-0.060 - 0.065	
Uncinate fasciculus R				0.933
- Mean (SD)	0.379 (0.042)	0.390 (0.034)	0.011 (0.040)	
- Range	0.247 - 0.471	0.328 - 0.462	-0.092 - 0.128	
Uncinate fasciculus L				0.933
- Mean (SD)	0.382 (0.041)	0.389 (0.041)	0.007 (0.036)	
- Range	0.305 - 0.457	0.319 - 0.452	-0.054 - 0.093	
Tapetum R				0.933
- Mean (SD)	0.330 (0.031)	0.335 (0.033)	0.005 (0.024)	
- Range	0.283 - 0.396	0.264 - 0.387	-0.039 - 0.048	
Tapetum L				0.993
- Mean (SD)	0.297 (0.027)	0.297 (0.030)	-0.000 (0.023)	
- Range	0.254 - 0.358	0.228 - 0.372	-0.047 - 0.049	

Mean diffusivity (MD, (10^-3)*(mm^2)/s) (DTI) - Frail (OOM+OLM) subjects pre vs post (paired tests) - (JHU white-matter tractography atlas) - Uncorrected p-values

	Post (N=31)	Pre (N=31)	Difference (N=31)	p value
Middle cerebellar peduncle				0.500
- Mean (SD)	1.094 (0.124)	1.072 (0.117)	-0.022 (0.179)	
- Range	0.874 - 1.380	0.915 - 1.425	-0.454 - 0.414	
Pontine crossing tract				0.606
- Mean (SD)	0.801 (0.062)	0.812 (0.077)	0.011 (0.114)	
- Range	0.681 - 0.967	0.717 - 1.066	-0.200 - 0.342	
Genu of corpus callosum				0.870
- Mean (SD)	1.361 (0.126)	1.363 (0.145)	0.001 (0.046)	
- Range	1.066 - 1.668	1.042 - 1.688	-0.105 - 0.112	
Body of corpus callosum				0.525
- Mean (SD)	1.118 (0.075)	1.124 (0.093)	0.006 (0.052)	
- Range	0.981 - 1.313	0.984 - 1.370	-0.075 - 0.153	
Splenium of corpus callosum				0.069
- Mean (SD)	1.007 (0.060)	1.025 (0.094)	0.019 (0.055)	
- Range	0.901 - 1.157	0.886 - 1.287	-0.061 - 0.157	
Fornix				0.505
- Mean (SD)	2.370 (0.270)	2.362 (0.301)	-0.009 (0.071)	
- Range	1.880 - 2.864	1.853 - 2.857	-0.194 - 0.115	
Corticospinal tract R				0.976
- Mean (SD)	0.845 (0.063)	0.845 (0.065)	0.000 (0.078)	
- Range	0.708 - 0.978	0.710 - 1.012	-0.123 - 0.187	
Corticospinal tract L				0.535
- Mean (SD)	0.812 (0.062)	0.819 (0.059)	0.007 (0.065)	
- Range	0.696 - 0.933	0.720 - 0.951	-0.145 - 0.155	
Medial lemniscus R				0.130
- Mean (SD)	0.862 (0.052)	0.892 (0.107)	0.030 (0.108)	
- Range	0.758 - 0.948	0.742 - 1.210	-0.131 - 0.357	
Medial lemniscus L				0.096
- Mean (SD)	0.843 (0.062)	0.866 (0.060)	0.022 (0.073)	
- Range	0.706 - 0.996	0.744 - 1.034	-0.121 - 0.201	
Inferior cerebellar peduncle R				0.494
- Mean (SD)	1.160 (0.230)	1.119 (0.207)	-0.041 (0.327)	
- Range	0.915 - 1.926	0.927 - 1.875	-0.953 - 0.854	
Inferior cerebellar peduncle L				0.947
- Mean (SD)	1.082 (0.185)	1.078 (0.193)	-0.003 (0.288)	
- Range	0.852 - 1.784	0.885 - 1.748	-0.760 - 0.784	
Superior cerebellar peduncle R				0.741
- Mean (SD)	1.231 (0.172)	1.242 (0.149)	0.012 (0.194)	
- Range	0.971 - 1.547	0.947 - 1.532	-0.557 - 0.428	
Superior cerebellar peduncle L				0.814
- Mean (SD)	1.291 (0.211)	1.300 (0.176)	0.009 (0.203)	
- Range	0.955 - 1.938	0.913 - 1.623	-0.575 - 0.341	
Cerebral peduncle R				0.725
- Mean (SD)	0.882 (0.052)	0.884 (0.046)	0.002 (0.038)	
- Range	0.783 - 1.016	0.785 - 0.971	-0.135 - 0.114	
Cerebral peduncle L				0.923
- Mean (SD)	0.842 (0.042)	0.843 (0.040)	0.001 (0.035)	
- Range	0.749 - 0.915	0.733 - 0.926	-0.087 - 0.071	
Anterior limb of internal capsule R				0.434
- Mean (SD)	0.861 (0.060)	0.857 (0.065)	-0.004 (0.027)	
- Range	0.789 - 1.007	0.774 - 1.020	-0.103 - 0.037	
Anterior limb of internal capsule L				0.470
- Mean (SD)	0.872 (0.069)	0.878 (0.074)	0.006 (0.045)	
- Range	0.765 - 1.022	0.772 - 1.040	-0.044 - 0.195	
Posterior limb of internal capsule R				0.907
- Mean (SD)	0.767 (0.028)	0.767 (0.033)	-0.000 (0.020)	
- Range	0.717 - 0.832	0.718 - 0.857	-0.044 - 0.046	
Posterior limb of internal capsule L				0.055
- Mean (SD)	0.758 (0.029)	0.767 (0.033)	0.009 (0.024)	

	Post (N=31)	Pre (N=31)	Difference (N=31)	p value
- Range	0.711 - 0.831	0.703 - 0.847	-0.023 - 0.087	
Retrolenticular part of internal capsule R				0.693
- Mean (SD)	0.919 (0.053)	0.916 (0.063)	-0.003 (0.037)	
- Range	0.838 - 1.053	0.820 - 1.062	-0.073 - 0.132	
Retrolenticular part of internal capsule L				0.038
- Mean (SD)	0.916 (0.056)	0.933 (0.070)	0.017 (0.043)	
- Range	0.833 - 1.114	0.822 - 1.088	-0.057 - 0.119	
Anterior corona radiata R				0.369
- Mean (SD)	0.945 (0.095)	0.953 (0.112)	0.008 (0.048)	
- Range	0.806 - 1.194	0.790 - 1.248	-0.034 - 0.236	
Anterior corona radiata L				0.181
- Mean (SD)	0.940 (0.104)	0.950 (0.120)	0.010 (0.040)	
- Range	0.775 - 1.179	0.791 - 1.298	-0.027 - 0.176	
Superior corona radiata R				0.655
- Mean (SD)	0.820 (0.082)	0.822 (0.082)	0.003 (0.031)	
- Range	0.724 - 1.109	0.708 - 1.096	-0.072 - 0.121	
Superior corona radiata L				0.274
- Mean (SD)	0.832 (0.073)	0.840 (0.089)	0.008 (0.042)	
- Range	0.720 - 1.069	0.705 - 1.099	-0.044 - 0.169	
Posterior corona radiata R				0.948
- Mean (SD)	0.952 (0.103)	0.951 (0.103)	-0.001 (0.061)	
- Range	0.801 - 1.181	0.780 - 1.178	-0.131 - 0.218	
Posterior corona radiata L				0.131
- Mean (SD)	0.966 (0.101)	0.985 (0.125)	0.018 (0.065)	
- Range	0.810 - 1.160	0.800 - 1.328	-0.091 - 0.202	
Posterior thalamic radiation R				0.669
- Mean (SD)	1.023 (0.163)	1.018 (0.136)	-0.004 (0.058)	
- Range	0.834 - 1.518	0.828 - 1.510	-0.207 - 0.135	
Posterior thalamic radiation L				0.672
- Mean (SD)	1.128 (0.215)	1.138 (0.175)	0.010 (0.125)	
- Range	0.867 - 2.104	0.934 - 1.606	-0.498 - 0.373	
Sagittal stratum R				0.587
- Mean (SD)	1.001 (0.088)	0.996 (0.094)	-0.005 (0.050)	
- Range	0.868 - 1.208	0.880 - 1.335	-0.156 - 0.137	
Sagittal stratum L				0.449
- Mean (SD)	1.083 (0.145)	1.072 (0.113)	-0.011 (0.077)	
- Range	0.898 - 1.571	0.881 - 1.305	-0.317 - 0.163	
External capsule R				0.245
- Mean (SD)	0.860 (0.057)	0.865 (0.058)	0.006 (0.027)	
- Range	0.772 - 1.012	0.784 - 0.996	-0.040 - 0.075	
External capsule L				0.052
- Mean (SD)	0.850 (0.050)	0.839 (0.057)	-0.011 (0.030)	
- Range	0.771 - 0.955	0.765 - 0.953	-0.071 - 0.072	
Cingulum cingulate gyrus R				0.481
- Mean (SD)	0.915 (0.074)	0.910 (0.063)	-0.005 (0.041)	
- Range	0.770 - 1.073	0.792 - 1.082	-0.109 - 0.073	
Cingulum cingulate gyrus L				0.045
- Mean (SD)	0.937 (0.064)	0.954 (0.079)	0.016 (0.044)	
- Range	0.824 - 1.047	0.828 - 1.115	-0.041 - 0.146	
Cingulum hippocampus R				0.426
- Mean (SD)	1.091 (0.129)	1.071 (0.116)	-0.020 (0.138)	
- Range	0.855 - 1.506	0.887 - 1.368	-0.480 - 0.257	
Cingulum hippocampus L				0.709
- Mean (SD)	1.129 (0.109)	1.121 (0.131)	-0.008 (0.123)	
- Range	0.870 - 1.336	0.897 - 1.407	-0.297 - 0.213	
Fornix R				0.079
- Mean (SD)	1.065 (0.110)	1.085 (0.127)	0.020 (0.060)	
- Range	0.847 - 1.298	0.844 - 1.370	-0.072 - 0.273	
Fornix L				0.367
- Mean (SD)	1.015 (0.107)	1.024 (0.118)	0.010 (0.059)	
- Range	0.811 - 1.211	0.841 - 1.313	-0.126 - 0.121	
Superior longitudinal fasciculus R				0.368

	Post (N=31)	Pre (N=31)	Difference (N=31)	p value
- Mean (SD)	0.808 (0.054)	0.803 (0.049)	-0.004 (0.026)	
- Range	0.734 - 0.967	0.734 - 0.950	-0.070 - 0.043	
Superior longitudinal fasciculus L				0.955
- Mean (SD)	0.793 (0.047)	0.793 (0.045)	-0.000 (0.025)	
- Range	0.705 - 0.904	0.730 - 0.904	-0.054 - 0.052	
Superior fronto occipital fasciculus R				0.592
- Mean (SD)	0.945 (0.182)	0.953 (0.222)	0.009 (0.090)	
- Range	0.745 - 1.503	0.723 - 1.571	-0.195 - 0.271	
Superior fronto occipital fasciculus L				0.281
- Mean (SD)	1.053 (0.209)	1.076 (0.268)	0.023 (0.115)	
- Range	0.770 - 1.580	0.795 - 1.863	-0.151 - 0.329	
Uncinate fasciculus R				0.568
- Mean (SD)	0.919 (0.164)	0.906 (0.113)	-0.014 (0.134)	
- Range	0.763 - 1.519	0.790 - 1.267	-0.475 - 0.291	
Uncinate fasciculus L				0.521
- Mean (SD)	0.898 (0.153)	0.883 (0.107)	-0.015 (0.132)	
- Range	0.778 - 1.444	0.780 - 1.287	-0.443 - 0.301	
Tapetum R				0.130
- Mean (SD)	1.996 (0.373)	2.055 (0.403)	0.059 (0.212)	
- Range	1.190 - 2.657	1.141 - 2.681	-0.307 - 0.440	
Tapetum L				0.214
- Mean (SD)	2.274 (0.398)	2.325 (0.423)	0.051 (0.224)	
- Range	1.418 - 3.016	1.407 - 2.936	-0.346 - 0.557	

Mean diffusivity (MD, (10^-3)*(mm^2)/s) (DTI) - Frail (OOM+OLM) subjects pre vs post (paired tests) - (JHU white-matter tractography atlas) - FDR corrected p-values

	Post (N=31)	Pre (N=31)	Difference (N=31)	p value
Middle cerebellar peduncle				0.881
- Mean (SD)	1.094 (0.124)	1.072 (0.117)	-0.022 (0.179)	
- Range	0.874 - 1.380	0.915 - 1.425	-0.454 - 0.414	
Pontine crossing tract				0.881
- Mean (SD)	0.801 (0.062)	0.812 (0.077)	0.011 (0.114)	
- Range	0.681 - 0.967	0.717 - 1.066	-0.200 - 0.342	
Genu of corpus callosum				0.975
- Mean (SD)	1.361 (0.126)	1.363 (0.145)	0.001 (0.046)	
- Range	1.066 - 1.668	1.042 - 1.688	-0.105 - 0.112	
Body of corpus callosum				0.881
- Mean (SD)	1.118 (0.075)	1.124 (0.093)	0.006 (0.052)	
- Range	0.981 - 1.313	0.984 - 1.370	-0.075 - 0.153	
Splenium of corpus callosum				0.626
- Mean (SD)	1.007 (0.060)	1.025 (0.094)	0.019 (0.055)	
- Range	0.901 - 1.157	0.886 - 1.287	-0.061 - 0.157	
Fornix				0.881
- Mean (SD)	2.370 (0.270)	2.362 (0.301)	-0.009 (0.071)	
- Range	1.880 - 2.864	1.853 - 2.857	-0.194 - 0.115	
Corticospinal tract R				0.976
- Mean (SD)	0.845 (0.063)	0.845 (0.065)	0.000 (0.078)	
- Range	0.708 - 0.978	0.710 - 1.012	-0.123 - 0.187	
Corticospinal tract L				0.881
- Mean (SD)	0.812 (0.062)	0.819 (0.059)	0.007 (0.065)	
- Range	0.696 - 0.933	0.720 - 0.951	-0.145 - 0.155	
Medial lemniscus R				0.626
- Mean (SD)	0.862 (0.052)	0.892 (0.107)	0.030 (0.108)	
- Range	0.758 - 0.948	0.742 - 1.210	-0.131 - 0.357	
Medial lemniscus L				0.626
- Mean (SD)	0.843 (0.062)	0.866 (0.060)	0.022 (0.073)	
- Range	0.706 - 0.996	0.744 - 1.034	-0.121 - 0.201	
Inferior cerebellar peduncle R				0.881
- Mean (SD)	1.160 (0.230)	1.119 (0.207)	-0.041 (0.327)	
- Range	0.915 - 1.926	0.927 - 1.875	-0.953 - 0.854	
Inferior cerebellar peduncle L				0.975
- Mean (SD)	1.082 (0.185)	1.078 (0.193)	-0.003 (0.288)	
- Range	0.852 - 1.784	0.885 - 1.748	-0.760 - 0.784	
Superior cerebellar peduncle R				0.889
- Mean (SD)	1.231 (0.172)	1.242 (0.149)	0.012 (0.194)	
- Range	0.971 - 1.547	0.947 - 1.532	-0.557 - 0.428	
Superior cerebellar peduncle L				0.953
- Mean (SD)	1.291 (0.211)	1.300 (0.176)	0.009 (0.203)	
- Range	0.955 - 1.938	0.913 - 1.623	-0.575 - 0.341	
Cerebral peduncle R				0.889
- Mean (SD)	0.882 (0.052)	0.884 (0.046)	0.002 (0.038)	
- Range	0.783 - 1.016	0.785 - 0.971	-0.135 - 0.114	
Cerebral peduncle L				0.975
- Mean (SD)	0.842 (0.042)	0.843 (0.040)	0.001 (0.035)	
- Range	0.749 - 0.915	0.733 - 0.926	-0.087 - 0.071	
Anterior limb of internal capsule R				0.881
- Mean (SD)	0.861 (0.060)	0.857 (0.065)	-0.004 (0.027)	
- Range	0.789 - 1.007	0.774 - 1.020	-0.103 - 0.037	
Anterior limb of internal capsule L				0.881
- Mean (SD)	0.872 (0.069)	0.878 (0.074)	0.006 (0.045)	
- Range	0.765 - 1.022	0.772 - 1.040	-0.044 - 0.195	
Posterior limb of internal capsule R				0.975
- Mean (SD)	0.767 (0.028)	0.767 (0.033)	-0.000 (0.020)	
- Range	0.717 - 0.832	0.718 - 0.857	-0.044 - 0.046	
Posterior limb of internal capsule L				0.626
- Mean (SD)	0.758 (0.029)	0.767 (0.033)	0.009 (0.024)	

	Post (N=31)	Pre (N=31)	Difference (N=31)	p value
- Range	0.711 - 0.831	0.703 - 0.847	-0.023 - 0.087	
Retrolenticular part of internal capsule R				0.889
- Mean (SD)	0.919 (0.053)	0.916 (0.063)	-0.003 (0.037)	
- Range	0.838 - 1.053	0.820 - 1.062	-0.073 - 0.132	
Retrolenticular part of internal capsule L				0.626
- Mean (SD)	0.916 (0.056)	0.933 (0.070)	0.017 (0.043)	
- Range	0.833 - 1.114	0.822 - 1.088	-0.057 - 0.119	
Anterior corona radiata R				0.881
- Mean (SD)	0.945 (0.095)	0.953 (0.112)	0.008 (0.048)	
- Range	0.806 - 1.194	0.790 - 1.248	-0.034 - 0.236	
Anterior corona radiata L				0.792
- Mean (SD)	0.940 (0.104)	0.950 (0.120)	0.010 (0.040)	
- Range	0.775 - 1.179	0.791 - 1.298	-0.027 - 0.176	
Superior corona radiata R				0.889
- Mean (SD)	0.820 (0.082)	0.822 (0.082)	0.003 (0.031)	
- Range	0.724 - 1.109	0.708 - 1.096	-0.072 - 0.121	
Superior corona radiata L				0.881
- Mean (SD)	0.832 (0.073)	0.840 (0.089)	0.008 (0.042)	
- Range	0.720 - 1.069	0.705 - 1.099	-0.044 - 0.169	
Posterior corona radiata R				0.975
- Mean (SD)	0.952 (0.103)	0.951 (0.103)	-0.001 (0.061)	
- Range	0.801 - 1.181	0.780 - 1.178	-0.131 - 0.218	
Posterior corona radiata L				0.626
- Mean (SD)	0.966 (0.101)	0.985 (0.125)	0.018 (0.065)	
- Range	0.810 - 1.160	0.800 - 1.328	-0.091 - 0.202	
Posterior thalamic radiation R				0.889
- Mean (SD)	1.023 (0.163)	1.018 (0.136)	-0.004 (0.058)	
- Range	0.834 - 1.518	0.828 - 1.510	-0.207 - 0.135	
Posterior thalamic radiation L				0.889
- Mean (SD)	1.128 (0.215)	1.138 (0.175)	0.010 (0.125)	
- Range	0.867 - 2.104	0.934 - 1.606	-0.498 - 0.373	
Sagittal stratum R				0.881
- Mean (SD)	1.001 (0.088)	0.996 (0.094)	-0.005 (0.050)	
- Range	0.868 - 1.208	0.880 - 1.335	-0.156 - 0.137	
Sagittal stratum L				0.881
- Mean (SD)	1.083 (0.145)	1.072 (0.113)	-0.011 (0.077)	
- Range	0.898 - 1.571	0.881 - 1.305	-0.317 - 0.163	
External capsule R				0.881
- Mean (SD)	0.860 (0.057)	0.865 (0.058)	0.006 (0.027)	
- Range	0.772 - 1.012	0.784 - 0.996	-0.040 - 0.075	
External capsule L				0.626
- Mean (SD)	0.850 (0.050)	0.839 (0.057)	-0.011 (0.030)	
- Range	0.771 - 0.955	0.765 - 0.953	-0.071 - 0.072	
Cingulum cingulate gyrus R				0.881
- Mean (SD)	0.915 (0.074)	0.910 (0.063)	-0.005 (0.041)	
- Range	0.770 - 1.073	0.792 - 1.082	-0.109 - 0.073	
Cingulum cingulate gyrus L				0.626
- Mean (SD)	0.937 (0.064)	0.954 (0.079)	0.016 (0.044)	
- Range	0.824 - 1.047	0.828 - 1.115	-0.041 - 0.146	
Cingulum hippocampus R				0.881
- Mean (SD)	1.091 (0.129)	1.071 (0.116)	-0.020 (0.138)	
- Range	0.855 - 1.506	0.887 - 1.368	-0.480 - 0.257	
Cingulum hippocampus L				0.889
- Mean (SD)	1.129 (0.109)	1.121 (0.131)	-0.008 (0.123)	
- Range	0.870 - 1.336	0.897 - 1.407	-0.297 - 0.213	
Fornix R				0.626
- Mean (SD)	1.065 (0.110)	1.085 (0.127)	0.020 (0.060)	
- Range	0.847 - 1.298	0.844 - 1.370	-0.072 - 0.273	
Fornix L				0.881
- Mean (SD)	1.015 (0.107)	1.024 (0.118)	0.010 (0.059)	
- Range	0.811 - 1.211	0.841 - 1.313	-0.126 - 0.121	
Superior longitudinal fasciculus R				0.881

	Post (N=31)	Pre (N=31)	Difference (N=31)	p value
- Mean (SD)	0.808 (0.054)	0.803 (0.049)	-0.004 (0.026)	
- Range	0.734 - 0.967	0.734 - 0.950	-0.070 - 0.043	
Superior longitudinal fasciculus L				0.975
- Mean (SD)	0.793 (0.047)	0.793 (0.045)	-0.000 (0.025)	
- Range	0.705 - 0.904	0.730 - 0.904	-0.054 - 0.052	
Superior fronto occipital fasciculus R				0.881
- Mean (SD)	0.945 (0.182)	0.953 (0.222)	0.009 (0.090)	
- Range	0.745 - 1.503	0.723 - 1.571	-0.195 - 0.271	
Superior fronto occipital fasciculus L				0.881
- Mean (SD)	1.053 (0.209)	1.076 (0.268)	0.023 (0.115)	
- Range	0.770 - 1.580	0.795 - 1.863	-0.151 - 0.329	
Uncinate fasciculus R				0.881
- Mean (SD)	0.919 (0.164)	0.906 (0.113)	-0.014 (0.134)	
- Range	0.763 - 1.519	0.790 - 1.267	-0.475 - 0.291	
Uncinate fasciculus L				0.881
- Mean (SD)	0.898 (0.153)	0.883 (0.107)	-0.015 (0.132)	
- Range	0.778 - 1.444	0.780 - 1.287	-0.443 - 0.301	
Tapetum R				0.626
- Mean (SD)	1.996 (0.373)	2.055 (0.403)	0.059 (0.212)	
- Range	1.190 - 2.657	1.141 - 2.681	-0.307 - 0.440	
Tapetum L				0.858
- Mean (SD)	2.274 (0.398)	2.325 (0.423)	0.051 (0.224)	
- Range	1.418 - 3.016	1.407 - 2.936	-0.346 - 0.557	

Radial diffusivity (RD, (10^-3)*(mm^2)/s) (DTI) - Frail (OOM+OLM) subjects pre vs post (paired tests) - (JHU white-matter tractography atlas) - Uncorrected p-values

	Post (N=31)	Pre (N=31)	Difference (N=31)	p value
Middle cerebellar peduncle				0.572
- Mean (SD)	0.868 (0.125)	0.848 (0.120)	-0.019 (0.188)	
- Range	0.663 - 1.171	0.682 - 1.228	-0.410 - 0.447	
Pontine crossing tract				0.752
- Mean (SD)	0.634 (0.059)	0.641 (0.074)	0.006 (0.109)	
- Range	0.522 - 0.792	0.528 - 0.891	-0.195 - 0.327	
Genu of corpus callosum				0.588
- Mean (SD)	1.049 (0.113)	1.052 (0.131)	0.003 (0.034)	
- Range	0.782 - 1.317	0.755 - 1.335	-0.072 - 0.090	
Body of corpus callosum				0.405
- Mean (SD)	0.807 (0.079)	0.813 (0.090)	0.006 (0.041)	
- Range	0.667 - 1.007	0.683 - 1.027	-0.062 - 0.126	
Splenium of corpus callosum				0.071
- Mean (SD)	0.637 (0.059)	0.652 (0.082)	0.015 (0.045)	
- Range	0.516 - 0.750	0.497 - 0.857	-0.057 - 0.121	
Fornix				0.166
- Mean (SD)	2.073 (0.283)	2.055 (0.303)	-0.018 (0.071)	
- Range	1.593 - 2.597	1.568 - 2.596	-0.184 - 0.134	
Corticospinal tract R				0.882
- Mean (SD)	0.623 (0.051)	0.622 (0.057)	-0.002 (0.062)	
- Range	0.521 - 0.709	0.523 - 0.787	-0.129 - 0.172	
Corticospinal tract L				0.469
- Mean (SD)	0.593 (0.052)	0.600 (0.050)	0.007 (0.053)	
- Range	0.513 - 0.743	0.488 - 0.697	-0.134 - 0.098	
Medial lemniscus R				0.099
- Mean (SD)	0.637 (0.048)	0.666 (0.092)	0.029 (0.094)	
- Range	0.570 - 0.743	0.515 - 0.953	-0.128 - 0.332	
Medial lemniscus L				0.156
- Mean (SD)	0.623 (0.058)	0.641 (0.063)	0.018 (0.070)	
- Range	0.509 - 0.777	0.523 - 0.808	-0.095 - 0.163	
Inferior cerebellar peduncle R				0.442
- Mean (SD)	0.959 (0.204)	0.917 (0.191)	-0.042 (0.300)	
- Range	0.744 - 1.601	0.722 - 1.625	-0.825 - 0.786	
Inferior cerebellar peduncle L				0.998
- Mean (SD)	0.887 (0.166)	0.887 (0.179)	-0.000 (0.260)	
- Range	0.668 - 1.471	0.687 - 1.498	-0.627 - 0.761	
Superior cerebellar peduncle R				0.787
- Mean (SD)	0.922 (0.174)	0.933 (0.157)	0.011 (0.228)	
- Range	0.695 - 1.286	0.687 - 1.291	-0.562 - 0.563	
Superior cerebellar peduncle L				0.794
- Mean (SD)	0.993 (0.203)	1.003 (0.167)	0.011 (0.225)	
- Range	0.686 - 1.702	0.713 - 1.378	-0.677 - 0.457	
Cerebral peduncle R				0.437
- Mean (SD)	0.584 (0.047)	0.588 (0.039)	0.004 (0.032)	
- Range	0.515 - 0.703	0.512 - 0.662	-0.096 - 0.075	
Cerebral peduncle L				0.594
- Mean (SD)	0.542 (0.037)	0.545 (0.038)	0.003 (0.028)	
- Range	0.473 - 0.611	0.473 - 0.623	-0.062 - 0.068	
Anterior limb of internal capsule R				0.968
- Mean (SD)	0.642 (0.063)	0.642 (0.069)	0.000 (0.027)	
- Range	0.561 - 0.812	0.568 - 0.837	-0.082 - 0.049	
Anterior limb of internal capsule L				0.427
- Mean (SD)	0.645 (0.067)	0.651 (0.076)	0.006 (0.045)	
- Range	0.536 - 0.785	0.556 - 0.820	-0.045 - 0.181	
Posterior limb of internal capsule R				0.482
- Mean (SD)	0.515 (0.028)	0.517 (0.034)	0.002 (0.017)	
- Range	0.473 - 0.574	0.465 - 0.600	-0.048 - 0.034	
Posterior limb of internal capsule L				0.110
- Mean (SD)	0.505 (0.028)	0.512 (0.033)	0.007 (0.024)	

	Post (N=31)	Pre (N=31)	Difference (N=31)	p value
- Range	0.456 - 0.578	0.448 - 0.603	-0.021 - 0.081	
Retrolenticular part of internal capsule R				0.745
- Mean (SD)	0.689 (0.047)	0.691 (0.057)	0.002 (0.034)	
- Range	0.608 - 0.792	0.589 - 0.797	-0.052 - 0.127	
Retrolenticular part of internal capsule L				0.176
- Mean (SD)	0.685 (0.052)	0.695 (0.063)	0.010 (0.041)	
- Range	0.591 - 0.865	0.572 - 0.826	-0.061 - 0.121	
Anterior corona radiata R				0.234
- Mean (SD)	0.775 (0.088)	0.784 (0.106)	0.009 (0.040)	
- Range	0.636 - 0.989	0.625 - 1.054	-0.031 - 0.204	
Anterior corona radiata L				0.191
- Mean (SD)	0.769 (0.095)	0.778 (0.106)	0.009 (0.036)	
- Range	0.615 - 0.953	0.624 - 1.070	-0.038 - 0.152	
Superior corona radiata R				0.400
- Mean (SD)	0.635 (0.074)	0.639 (0.074)	0.004 (0.026)	
- Range	0.552 - 0.904	0.544 - 0.907	-0.049 - 0.102	
Superior corona radiata L				0.264
- Mean (SD)	0.646 (0.066)	0.652 (0.074)	0.007 (0.032)	
- Range	0.562 - 0.896	0.546 - 0.881	-0.036 - 0.125	
Posterior corona radiata R				0.671
- Mean (SD)	0.728 (0.087)	0.731 (0.090)	0.004 (0.046)	
- Range	0.613 - 0.941	0.598 - 0.964	-0.088 - 0.181	
Posterior corona radiata L				0.203
- Mean (SD)	0.754 (0.086)	0.765 (0.100)	0.011 (0.049)	
- Range	0.626 - 0.959	0.608 - 1.002	-0.069 - 0.139	
Posterior thalamic radiation R				0.845
- Mean (SD)	0.755 (0.127)	0.757 (0.112)	0.001 (0.042)	
- Range	0.601 - 1.130	0.597 - 1.152	-0.133 - 0.108	
Posterior thalamic radiation L				0.736
- Mean (SD)	0.852 (0.180)	0.858 (0.147)	0.006 (0.103)	
- Range	0.647 - 1.665	0.661 - 1.258	-0.407 - 0.306	
Sagittal stratum R				0.596
- Mean (SD)	0.764 (0.072)	0.760 (0.079)	-0.004 (0.046)	
- Range	0.643 - 0.920	0.663 - 1.053	-0.139 - 0.140	
Sagittal stratum L				0.515
- Mean (SD)	0.851 (0.117)	0.844 (0.097)	-0.007 (0.063)	
- Range	0.692 - 1.230	0.685 - 1.037	-0.219 - 0.158	
External capsule R				0.283
- Mean (SD)	0.708 (0.056)	0.714 (0.056)	0.005 (0.028)	
- Range	0.624 - 0.861	0.637 - 0.838	-0.027 - 0.083	
External capsule L				0.051
- Mean (SD)	0.695 (0.047)	0.685 (0.055)	-0.010 (0.027)	
- Range	0.631 - 0.771	0.613 - 0.795	-0.070 - 0.063	
Cingulum cingulate gyrus R				0.753
- Mean (SD)	0.738 (0.070)	0.736 (0.061)	-0.002 (0.039)	
- Range	0.621 - 0.889	0.640 - 0.915	-0.098 - 0.081	
Cingulum cingulate gyrus L				0.055
- Mean (SD)	0.747 (0.060)	0.760 (0.072)	0.013 (0.037)	
- Range	0.637 - 0.865	0.655 - 0.912	-0.032 - 0.130	
Cingulum hippocampus R				0.583
- Mean (SD)	0.932 (0.122)	0.919 (0.113)	-0.013 (0.129)	
- Range	0.714 - 1.327	0.715 - 1.178	-0.453 - 0.261	
Cingulum hippocampus L				0.870
- Mean (SD)	0.970 (0.107)	0.966 (0.122)	-0.003 (0.116)	
- Range	0.738 - 1.161	0.778 - 1.214	-0.266 - 0.199	
Fornix R				0.187
- Mean (SD)	0.865 (0.102)	0.878 (0.121)	0.013 (0.056)	
- Range	0.650 - 1.104	0.642 - 1.144	-0.049 - 0.239	
Fornix L				0.268
- Mean (SD)	0.809 (0.100)	0.820 (0.110)	0.010 (0.051)	
- Range	0.623 - 1.027	0.648 - 1.103	-0.111 - 0.111	
Superior longitudinal fasciculus R				0.562

	Post (N=31)	Pre (N=31)	Difference (N=31)	p value
- Mean (SD)	0.638 (0.056)	0.635 (0.049)	-0.003 (0.028)	
- Range	0.567 - 0.809	0.571 - 0.766	-0.082 - 0.040	
Superior longitudinal fasciculus L				0.932
- Mean (SD)	0.624 (0.046)	0.624 (0.047)	-0.000 (0.023)	
- Range	0.532 - 0.711	0.553 - 0.730	-0.046 - 0.049	
Superior fronto occipital fasciculus R				0.454
- Mean (SD)	0.769 (0.171)	0.779 (0.205)	0.010 (0.072)	
- Range	0.578 - 1.254	0.561 - 1.371	-0.140 - 0.189	
Superior fronto occipital fasciculus L				0.276
- Mean (SD)	0.872 (0.200)	0.893 (0.245)	0.020 (0.102)	
- Range	0.599 - 1.310	0.622 - 1.541	-0.126 - 0.290	
Uncinate fasciculus R				0.463
- Mean (SD)	0.729 (0.160)	0.712 (0.109)	-0.017 (0.129)	
- Range	0.557 - 1.324	0.598 - 1.078	-0.499 - 0.234	
Uncinate fasciculus L				0.408
- Mean (SD)	0.715 (0.148)	0.696 (0.105)	-0.019 (0.127)	
- Range	0.595 - 1.225	0.577 - 1.063	-0.451 - 0.267	
Tapetum R				0.161
- Mean (SD)	1.666 (0.327)	1.707 (0.350)	0.042 (0.162)	
- Range	0.966 - 2.245	0.926 - 2.218	-0.230 - 0.347	
Tapetum L				0.173
- Mean (SD)	1.946 (0.349)	1.993 (0.372)	0.047 (0.188)	
- Range	1.224 - 2.603	1.204 - 2.503	-0.258 - 0.490	

Radial diffusivity (RD, (10^-3)*(mm^2)/s) (DTI) - Frail (OOM+OLM) subjects pre vs post (paired tests) - (JHU white-matter tractography atlas) - FDR corrected p-values

	Post (N=31)	Pre (N=31)	Difference (N=31)	p value
Middle cerebellar peduncle				0.817
- Mean (SD)	0.868 (0.125)	0.848 (0.120)	-0.019 (0.188)	
- Range	0.663 - 1.171	0.682 - 1.228	-0.410 - 0.447	
Pontine crossing tract				0.904
- Mean (SD)	0.634 (0.059)	0.641 (0.074)	0.006 (0.109)	
- Range	0.522 - 0.792	0.528 - 0.891	-0.195 - 0.327	
Genu of corpus callosum				0.817
- Mean (SD)	1.049 (0.113)	1.052 (0.131)	0.003 (0.034)	
- Range	0.782 - 1.317	0.755 - 1.335	-0.072 - 0.090	
Body of corpus callosum				0.817
- Mean (SD)	0.807 (0.079)	0.813 (0.090)	0.006 (0.041)	
- Range	0.667 - 1.007	0.683 - 1.027	-0.062 - 0.126	
Splenium of corpus callosum				0.750
- Mean (SD)	0.637 (0.059)	0.652 (0.082)	0.015 (0.045)	
- Range	0.516 - 0.750	0.497 - 0.857	-0.057 - 0.121	
Fornix				0.750
- Mean (SD)	2.073 (0.283)	2.055 (0.303)	-0.018 (0.071)	
- Range	1.593 - 2.597	1.568 - 2.596	-0.184 - 0.134	
Corticospinal tract R				0.941
- Mean (SD)	0.623 (0.051)	0.622 (0.057)	-0.002 (0.062)	
- Range	0.521 - 0.709	0.523 - 0.787	-0.129 - 0.172	
Corticospinal tract L				0.817
- Mean (SD)	0.593 (0.052)	0.600 (0.050)	0.007 (0.053)	
- Range	0.513 - 0.743	0.488 - 0.697	-0.134 - 0.098	
Medial lemniscus R				0.750
- Mean (SD)	0.637 (0.048)	0.666 (0.092)	0.029 (0.094)	
- Range	0.570 - 0.743	0.515 - 0.953	-0.128 - 0.332	
Medial lemniscus L				0.750
- Mean (SD)	0.623 (0.058)	0.641 (0.063)	0.018 (0.070)	
- Range	0.509 - 0.777	0.523 - 0.808	-0.095 - 0.163	
Inferior cerebellar peduncle R				0.817
- Mean (SD)	0.959 (0.204)	0.917 (0.191)	-0.042 (0.300)	
- Range	0.744 - 1.601	0.722 - 1.625	-0.825 - 0.786	
Inferior cerebellar peduncle L				0.998
- Mean (SD)	0.887 (0.166)	0.887 (0.179)	-0.000 (0.260)	
- Range	0.668 - 1.471	0.687 - 1.498	-0.627 - 0.761	
Superior cerebellar peduncle R				0.908
- Mean (SD)	0.922 (0.174)	0.933 (0.157)	0.011 (0.228)	
- Range	0.695 - 1.286	0.687 - 1.291	-0.562 - 0.563	
Superior cerebellar peduncle L				0.908
- Mean (SD)	0.993 (0.203)	1.003 (0.167)	0.011 (0.225)	
- Range	0.686 - 1.702	0.713 - 1.378	-0.677 - 0.457	
Cerebral peduncle R				0.817
- Mean (SD)	0.584 (0.047)	0.588 (0.039)	0.004 (0.032)	
- Range	0.515 - 0.703	0.512 - 0.662	-0.096 - 0.075	
Cerebral peduncle L				0.817
- Mean (SD)	0.542 (0.037)	0.545 (0.038)	0.003 (0.028)	
- Range	0.473 - 0.611	0.473 - 0.623	-0.062 - 0.068	
Anterior limb of internal capsule R				0.989
- Mean (SD)	0.642 (0.063)	0.642 (0.069)	0.000 (0.027)	
- Range	0.561 - 0.812	0.568 - 0.837	-0.082 - 0.049	
Anterior limb of internal capsule L				0.817
- Mean (SD)	0.645 (0.067)	0.651 (0.076)	0.006 (0.045)	
- Range	0.536 - 0.785	0.556 - 0.820	-0.045 - 0.181	
Posterior limb of internal capsule R				0.817
- Mean (SD)	0.515 (0.028)	0.517 (0.034)	0.002 (0.017)	
- Range	0.473 - 0.574	0.465 - 0.600	-0.048 - 0.034	
Posterior limb of internal capsule L				0.750
- Mean (SD)	0.505 (0.028)	0.512 (0.033)	0.007 (0.024)	

	Post (N=31)	Pre (N=31)	Difference (N=31)	p value
- Range	0.456 - 0.578	0.448 - 0.603	-0.021 - 0.081	
Retrolenticular part of internal capsule R				0.904
- Mean (SD)	0.689 (0.047)	0.691 (0.057)	0.002 (0.034)	
- Range	0.608 - 0.792	0.589 - 0.797	-0.052 - 0.127	
Retrolenticular part of internal capsule L				0.750
- Mean (SD)	0.685 (0.052)	0.695 (0.063)	0.010 (0.041)	
- Range	0.591 - 0.865	0.572 - 0.826	-0.061 - 0.121	
Anterior corona radiata R				0.754
- Mean (SD)	0.775 (0.088)	0.784 (0.106)	0.009 (0.040)	
- Range	0.636 - 0.989	0.625 - 1.054	-0.031 - 0.204	
Anterior corona radiata L				0.750
- Mean (SD)	0.769 (0.095)	0.778 (0.106)	0.009 (0.036)	
- Range	0.615 - 0.953	0.624 - 1.070	-0.038 - 0.152	
Superior corona radiata R				0.817
- Mean (SD)	0.635 (0.074)	0.639 (0.074)	0.004 (0.026)	
- Range	0.552 - 0.904	0.544 - 0.907	-0.049 - 0.102	
Superior corona radiata L				0.754
- Mean (SD)	0.646 (0.066)	0.652 (0.074)	0.007 (0.032)	
- Range	0.562 - 0.896	0.546 - 0.881	-0.036 - 0.125	
Posterior corona radiata R				0.895
- Mean (SD)	0.728 (0.087)	0.731 (0.090)	0.004 (0.046)	
- Range	0.613 - 0.941	0.598 - 0.964	-0.088 - 0.181	
Posterior corona radiata L				0.750
- Mean (SD)	0.754 (0.086)	0.765 (0.100)	0.011 (0.049)	
- Range	0.626 - 0.959	0.608 - 1.002	-0.069 - 0.139	
Posterior thalamic radiation R				0.941
- Mean (SD)	0.755 (0.127)	0.757 (0.112)	0.001 (0.042)	
- Range	0.601 - 1.130	0.597 - 1.152	-0.133 - 0.108	
Posterior thalamic radiation L				0.904
- Mean (SD)	0.852 (0.180)	0.858 (0.147)	0.006 (0.103)	
- Range	0.647 - 1.665	0.661 - 1.258	-0.407 - 0.306	
Sagittal stratum R				0.817
- Mean (SD)	0.764 (0.072)	0.760 (0.079)	-0.004 (0.046)	
- Range	0.643 - 0.920	0.663 - 1.053	-0.139 - 0.140	
Sagittal stratum L				0.817
- Mean (SD)	0.851 (0.117)	0.844 (0.097)	-0.007 (0.063)	
- Range	0.692 - 1.230	0.685 - 1.037	-0.219 - 0.158	
External capsule R				0.754
- Mean (SD)	0.708 (0.056)	0.714 (0.056)	0.005 (0.028)	
- Range	0.624 - 0.861	0.637 - 0.838	-0.027 - 0.083	
External capsule L				0.750
- Mean (SD)	0.695 (0.047)	0.685 (0.055)	-0.010 (0.027)	
- Range	0.631 - 0.771	0.613 - 0.795	-0.070 - 0.063	
Cingulum cingulate gyrus R				0.904
- Mean (SD)	0.738 (0.070)	0.736 (0.061)	-0.002 (0.039)	
- Range	0.621 - 0.889	0.640 - 0.915	-0.098 - 0.081	
Cingulum cingulate gyrus L				0.750
- Mean (SD)	0.747 (0.060)	0.760 (0.072)	0.013 (0.037)	
- Range	0.637 - 0.865	0.655 - 0.912	-0.032 - 0.130	
Cingulum hippocampus R				0.817
- Mean (SD)	0.932 (0.122)	0.919 (0.113)	-0.013 (0.129)	
- Range	0.714 - 1.327	0.715 - 1.178	-0.453 - 0.261	
Cingulum hippocampus L				0.941
- Mean (SD)	0.970 (0.107)	0.966 (0.122)	-0.003 (0.116)	
- Range	0.738 - 1.161	0.778 - 1.214	-0.266 - 0.199	
Fornix R				0.750
- Mean (SD)	0.865 (0.102)	0.878 (0.121)	0.013 (0.056)	
- Range	0.650 - 1.104	0.642 - 1.144	-0.049 - 0.239	
Fornix L				0.754
- Mean (SD)	0.809 (0.100)	0.820 (0.110)	0.010 (0.051)	
- Range	0.623 - 1.027	0.648 - 1.103	-0.111 - 0.111	
Superior longitudinal fasciculus R				0.817

	Post (N=31)	Pre (N=31)	Difference (N=31)	p value
- Mean (SD)	0.638 (0.056)	0.635 (0.049)	-0.003 (0.028)	
- Range	0.567 - 0.809	0.571 - 0.766	-0.082 - 0.040	
Superior longitudinal fasciculus L				0.973
- Mean (SD)	0.624 (0.046)	0.624 (0.047)	-0.000 (0.023)	
- Range	0.532 - 0.711	0.553 - 0.730	-0.046 - 0.049	
Superior fronto occipital fasciculus R				0.817
- Mean (SD)	0.769 (0.171)	0.779 (0.205)	0.010 (0.072)	
- Range	0.578 - 1.254	0.561 - 1.371	-0.140 - 0.189	
Superior fronto occipital fasciculus L				0.754
- Mean (SD)	0.872 (0.200)	0.893 (0.245)	0.020 (0.102)	
- Range	0.599 - 1.310	0.622 - 1.541	-0.126 - 0.290	
Uncinate fasciculus R				0.817
- Mean (SD)	0.729 (0.160)	0.712 (0.109)	-0.017 (0.129)	
- Range	0.557 - 1.324	0.598 - 1.078	-0.499 - 0.234	
Uncinate fasciculus L				0.817
- Mean (SD)	0.715 (0.148)	0.696 (0.105)	-0.019 (0.127)	
- Range	0.595 - 1.225	0.577 - 1.063	-0.451 - 0.267	
Tapetum R				0.750
- Mean (SD)	1.666 (0.327)	1.707 (0.350)	0.042 (0.162)	
- Range	0.966 - 2.245	0.926 - 2.218	-0.230 - 0.347	
Tapetum L				0.750
- Mean (SD)	1.946 (0.349)	1.993 (0.372)	0.047 (0.188)	
- Range	1.224 - 2.603	1.204 - 2.503	-0.258 - 0.490	

Fractional anisotropy (FA) (DTI) - Offspring of lean mothers (OLM) subjects pre vs post (paired tests) - (JHU white-matter tractography atlas) - Uncorrected p-values

	Post (N=18)	Pre (N=18)	Difference (N=18)	p value
Middle cerebellar peduncle				0.493
- Mean (SD)	0.401 (0.042)	0.413 (0.052)	0.011 (0.068)	
- Range	0.290 - 0.447	0.262 - 0.480	-0.138 - 0.121	
Pontine crossing tract				0.142
- Mean (SD)	0.387 (0.042)	0.408 (0.039)	0.021 (0.057)	
- Range	0.350 - 0.502	0.348 - 0.483	-0.142 - 0.116	
Genu of corpus callosum				0.860
- Mean (SD)	0.436 (0.028)	0.437 (0.030)	0.001 (0.016)	
- Range	0.385 - 0.478	0.383 - 0.498	-0.043 - 0.039	
Body of corpus callosum				0.446
- Mean (SD)	0.485 (0.031)	0.488 (0.037)	0.003 (0.017)	
- Range	0.440 - 0.539	0.426 - 0.553	-0.018 - 0.044	
Splenium of corpus callosum				0.796
- Mean (SD)	0.586 (0.034)	0.587 (0.036)	0.001 (0.022)	
- Range	0.531 - 0.653	0.533 - 0.669	-0.035 - 0.043	
Fornix				0.054
- Mean (SD)	0.237 (0.034)	0.252 (0.029)	0.015 (0.031)	
- Range	0.193 - 0.322	0.197 - 0.303	-0.063 - 0.078	
Corticospinal tract R				0.062
- Mean (SD)	0.455 (0.035)	0.476 (0.037)	0.020 (0.044)	
- Range	0.389 - 0.505	0.409 - 0.543	-0.062 - 0.095	
Corticospinal tract L				0.794
- Mean (SD)	0.486 (0.051)	0.490 (0.044)	0.004 (0.059)	
- Range	0.360 - 0.599	0.431 - 0.597	-0.125 - 0.130	
Medial lemniscus R				0.923
- Mean (SD)	0.451 (0.051)	0.450 (0.055)	-0.002 (0.066)	
- Range	0.368 - 0.540	0.335 - 0.560	-0.124 - 0.135	
Medial lemniscus L				0.856
- Mean (SD)	0.453 (0.059)	0.449 (0.055)	-0.003 (0.079)	
- Range	0.380 - 0.569	0.360 - 0.598	-0.143 - 0.163	
Inferior cerebellar peduncle R				0.455
- Mean (SD)	0.347 (0.037)	0.358 (0.042)	0.011 (0.062)	
- Range	0.298 - 0.460	0.272 - 0.429	-0.128 - 0.113	
Inferior cerebellar peduncle L				0.262
- Mean (SD)	0.354 (0.041)	0.368 (0.036)	0.014 (0.051)	
- Range	0.287 - 0.445	0.303 - 0.422	-0.087 - 0.117	
Superior cerebellar peduncle R				0.649
- Mean (SD)	0.463 (0.048)	0.473 (0.067)	0.010 (0.096)	
- Range	0.363 - 0.528	0.304 - 0.568	-0.206 - 0.181	
Superior cerebellar peduncle L				0.668
- Mean (SD)	0.443 (0.041)	0.451 (0.048)	0.007 (0.071)	
- Range	0.354 - 0.497	0.329 - 0.519	-0.146 - 0.148	
Cerebral peduncle R				0.346
- Mean (SD)	0.562 (0.029)	0.557 (0.033)	-0.005 (0.024)	
- Range	0.510 - 0.622	0.495 - 0.625	-0.034 - 0.062	
Cerebral peduncle L				0.437
- Mean (SD)	0.574 (0.028)	0.570 (0.033)	-0.005 (0.026)	
- Range	0.517 - 0.632	0.491 - 0.615	-0.058 - 0.047	
Anterior limb of internal capsule R				0.214
- Mean (SD)	0.433 (0.030)	0.428 (0.034)	-0.004 (0.014)	
- Range	0.360 - 0.474	0.341 - 0.482	-0.026 - 0.020	
Anterior limb of internal capsule L				0.942
- Mean (SD)	0.438 (0.026)	0.438 (0.033)	0.000 (0.021)	
- Range	0.400 - 0.490	0.389 - 0.491	-0.036 - 0.038	
Posterior limb of internal capsule R				0.473
- Mean (SD)	0.527 (0.021)	0.524 (0.027)	-0.003 (0.017)	
- Range	0.489 - 0.558	0.472 - 0.558	-0.038 - 0.029	
Posterior limb of internal capsule L				0.897
- Mean (SD)	0.534 (0.019)	0.535 (0.025)	0.000 (0.015)	

	Post (N=18)	Pre (N=18)	Difference (N=18)	p value
- Range	0.499 - 0.564	0.478 - 0.568	-0.038 - 0.024	
Retrolenticular part of internal capsule R				0.341
- Mean (SD)	0.432 (0.017)	0.429 (0.025)	-0.003 (0.014)	
- Range	0.406 - 0.468	0.389 - 0.481	-0.030 - 0.024	
Retrolenticular part of internal capsule L				0.852
- Mean (SD)	0.440 (0.022)	0.441 (0.025)	0.001 (0.025)	
- Range	0.406 - 0.483	0.402 - 0.501	-0.044 - 0.063	
Anterior corona radiata R				0.349
- Mean (SD)	0.329 (0.028)	0.325 (0.034)	-0.003 (0.015)	
- Range	0.272 - 0.370	0.263 - 0.383	-0.028 - 0.037	
Anterior corona radiata L				0.610
- Mean (SD)	0.327 (0.026)	0.330 (0.032)	0.002 (0.018)	
- Range	0.275 - 0.364	0.270 - 0.382	-0.034 - 0.040	
Superior corona radiata R				0.293
- Mean (SD)	0.401 (0.036)	0.398 (0.035)	-0.003 (0.014)	
- Range	0.334 - 0.473	0.315 - 0.451	-0.022 - 0.024	
Superior corona radiata L				0.587
- Mean (SD)	0.400 (0.037)	0.402 (0.040)	0.002 (0.014)	
- Range	0.314 - 0.485	0.311 - 0.478	-0.029 - 0.041	
Posterior corona radiata R				0.310
- Mean (SD)	0.409 (0.040)	0.404 (0.043)	-0.005 (0.020)	
- Range	0.354 - 0.505	0.331 - 0.480	-0.033 - 0.047	
Posterior corona radiata L				0.918
- Mean (SD)	0.396 (0.046)	0.396 (0.046)	0.000 (0.019)	
- Range	0.317 - 0.490	0.308 - 0.491	-0.048 - 0.040	
Posterior thalamic radiation R				0.018
- Mean (SD)	0.451 (0.038)	0.444 (0.036)	-0.008 (0.013)	
- Range	0.370 - 0.502	0.372 - 0.491	-0.030 - 0.011	
Posterior thalamic radiation L				0.453
- Mean (SD)	0.434 (0.028)	0.438 (0.029)	0.004 (0.020)	
- Range	0.379 - 0.482	0.388 - 0.497	-0.035 - 0.030	
Sagittal stratum R				0.897
- Mean (SD)	0.425 (0.018)	0.424 (0.021)	-0.001 (0.017)	
- Range	0.394 - 0.453	0.396 - 0.455	-0.023 - 0.032	
Sagittal stratum L				0.732
- Mean (SD)	0.390 (0.018)	0.388 (0.023)	-0.002 (0.023)	
- Range	0.357 - 0.431	0.346 - 0.425	-0.038 - 0.052	
External capsule R				0.899
- Mean (SD)	0.320 (0.020)	0.321 (0.024)	0.001 (0.018)	
- Range	0.288 - 0.353	0.280 - 0.365	-0.038 - 0.037	
External capsule L				0.665
- Mean (SD)	0.331 (0.020)	0.333 (0.027)	0.002 (0.019)	
- Range	0.301 - 0.362	0.302 - 0.397	-0.051 - 0.037	
Cingulum cingulate gyrus R				0.715
- Mean (SD)	0.357 (0.025)	0.355 (0.026)	-0.001 (0.016)	
- Range	0.315 - 0.411	0.303 - 0.401	-0.030 - 0.035	
Cingulum cingulate gyrus L				0.631
- Mean (SD)	0.365 (0.023)	0.367 (0.028)	0.002 (0.014)	
- Range	0.326 - 0.414	0.310 - 0.421	-0.028 - 0.021	
Cingulum hippocampus R				0.421
- Mean (SD)	0.288 (0.035)	0.281 (0.040)	-0.008 (0.040)	
- Range	0.235 - 0.377	0.213 - 0.371	-0.086 - 0.044	
Cingulum hippocampus L				0.761
- Mean (SD)	0.282 (0.036)	0.279 (0.034)	-0.003 (0.043)	
- Range	0.202 - 0.377	0.204 - 0.326	-0.105 - 0.068	
Fornix R				0.752
- Mean (SD)	0.358 (0.025)	0.360 (0.030)	0.002 (0.025)	
- Range	0.312 - 0.408	0.319 - 0.426	-0.041 - 0.037	
Fornix L				0.475
- Mean (SD)	0.382 (0.035)	0.376 (0.033)	-0.005 (0.031)	
- Range	0.324 - 0.443	0.330 - 0.438	-0.079 - 0.042	
Superior longitudinal fasciculus R				0.758

	Post (N=18)	Pre (N=18)	Difference (N=18)	p value
- Mean (SD)	0.386 (0.030)	0.385 (0.029)	-0.001 (0.014)	
- Range	0.326 - 0.444	0.346 - 0.426	-0.021 - 0.034	
Superior longitudinal fasciculus L				0.877
- Mean (SD)	0.387 (0.031)	0.386 (0.033)	-0.001 (0.017)	
- Range	0.338 - 0.440	0.320 - 0.431	-0.052 - 0.020	
Superior fronto occipital fasciculus R				0.147
- Mean (SD)	0.346 (0.038)	0.335 (0.050)	-0.011 (0.030)	
- Range	0.256 - 0.400	0.248 - 0.441	-0.081 - 0.041	
Superior fronto occipital fasciculus L				0.169
- Mean (SD)	0.317 (0.036)	0.324 (0.043)	0.007 (0.021)	
- Range	0.254 - 0.370	0.243 - 0.383	-0.023 - 0.065	
Uncinate fasciculus R				0.384
- Mean (SD)	0.384 (0.035)	0.392 (0.037)	0.008 (0.040)	
- Range	0.344 - 0.471	0.335 - 0.462	-0.092 - 0.065	
Uncinate fasciculus L				0.740
- Mean (SD)	0.381 (0.048)	0.384 (0.039)	0.003 (0.036)	
- Range	0.305 - 0.457	0.319 - 0.439	-0.054 - 0.093	
Tapetum R				0.665
- Mean (SD)	0.330 (0.035)	0.333 (0.034)	0.003 (0.026)	
- Range	0.287 - 0.396	0.264 - 0.383	-0.039 - 0.048	
Tapetum L				0.243
- Mean (SD)	0.300 (0.027)	0.293 (0.030)	-0.007 (0.024)	
- Range	0.258 - 0.358	0.228 - 0.335	-0.047 - 0.029	

Fractional anisotropy (FA) (DTI) - Offspring of lean mothers (OLM) subjects pre vs post (paired tests) - (JHU white-matter tractography atlas) - FDR corrected p-values

	Post (N=18)	Pre (N=18)	Difference (N=18)	p value
Middle cerebellar peduncle				0.942
- Mean (SD)	0.401 (0.042)	0.413 (0.052)	0.011 (0.068)	
- Range	0.290 - 0.447	0.262 - 0.480	-0.138 - 0.121	
Pontine crossing tract				0.942
- Mean (SD)	0.387 (0.042)	0.408 (0.039)	0.021 (0.057)	
- Range	0.350 - 0.502	0.348 - 0.483	-0.142 - 0.116	
Genu of corpus callosum				0.942
- Mean (SD)	0.436 (0.028)	0.437 (0.030)	0.001 (0.016)	
- Range	0.385 - 0.478	0.383 - 0.498	-0.043 - 0.039	
Body of corpus callosum				0.942
- Mean (SD)	0.485 (0.031)	0.488 (0.037)	0.003 (0.017)	
- Range	0.440 - 0.539	0.426 - 0.553	-0.018 - 0.044	
Splenium of corpus callosum				0.942
- Mean (SD)	0.586 (0.034)	0.587 (0.036)	0.001 (0.022)	
- Range	0.531 - 0.653	0.533 - 0.669	-0.035 - 0.043	
Fornix				0.942
- Mean (SD)	0.237 (0.034)	0.252 (0.029)	0.015 (0.031)	
- Range	0.193 - 0.322	0.197 - 0.303	-0.063 - 0.078	
Corticospinal tract R				0.942
- Mean (SD)	0.455 (0.035)	0.476 (0.037)	0.020 (0.044)	
- Range	0.389 - 0.505	0.409 - 0.543	-0.062 - 0.095	
Corticospinal tract L				0.942
- Mean (SD)	0.486 (0.051)	0.490 (0.044)	0.004 (0.059)	
- Range	0.360 - 0.599	0.431 - 0.597	-0.125 - 0.130	
Medial lemniscus R				0.942
- Mean (SD)	0.451 (0.051)	0.450 (0.055)	-0.002 (0.066)	
- Range	0.368 - 0.540	0.335 - 0.560	-0.124 - 0.135	
Medial lemniscus L				0.942
- Mean (SD)	0.453 (0.059)	0.449 (0.055)	-0.003 (0.079)	
- Range	0.380 - 0.569	0.360 - 0.598	-0.143 - 0.163	
Inferior cerebellar peduncle R				0.942
- Mean (SD)	0.347 (0.037)	0.358 (0.042)	0.011 (0.062)	
- Range	0.298 - 0.460	0.272 - 0.429	-0.128 - 0.113	
Inferior cerebellar peduncle L				0.942
- Mean (SD)	0.354 (0.041)	0.368 (0.036)	0.014 (0.051)	
- Range	0.287 - 0.445	0.303 - 0.422	-0.087 - 0.117	
Superior cerebellar peduncle R				0.942
- Mean (SD)	0.463 (0.048)	0.473 (0.067)	0.010 (0.096)	
- Range	0.363 - 0.528	0.304 - 0.568	-0.206 - 0.181	
Superior cerebellar peduncle L				0.942
- Mean (SD)	0.443 (0.041)	0.451 (0.048)	0.007 (0.071)	
- Range	0.354 - 0.497	0.329 - 0.519	-0.146 - 0.148	
Cerebral peduncle R				0.942
- Mean (SD)	0.562 (0.029)	0.557 (0.033)	-0.005 (0.024)	
- Range	0.510 - 0.622	0.495 - 0.625	-0.034 - 0.062	
Cerebral peduncle L				0.942
- Mean (SD)	0.574 (0.028)	0.570 (0.033)	-0.005 (0.026)	
- Range	0.517 - 0.632	0.491 - 0.615	-0.058 - 0.047	
Anterior limb of internal capsule R				0.942
- Mean (SD)	0.433 (0.030)	0.428 (0.034)	-0.004 (0.014)	
- Range	0.360 - 0.474	0.341 - 0.482	-0.026 - 0.020	
Anterior limb of internal capsule L				0.942
- Mean (SD)	0.438 (0.026)	0.438 (0.033)	0.000 (0.021)	
- Range	0.400 - 0.490	0.389 - 0.491	-0.036 - 0.038	
Posterior limb of internal capsule R				0.942
- Mean (SD)	0.527 (0.021)	0.524 (0.027)	-0.003 (0.017)	
- Range	0.489 - 0.558	0.472 - 0.558	-0.038 - 0.029	
Posterior limb of internal capsule L				0.942
- Mean (SD)	0.534 (0.019)	0.535 (0.025)	0.000 (0.015)	

	Post (N=18)	Pre (N=18)	Difference (N=18)	p value
- Range	0.499 - 0.564	0.478 - 0.568	-0.038 - 0.024	
Retrolenticular part of internal capsule R				0.942
- Mean (SD)	0.432 (0.017)	0.429 (0.025)	-0.003 (0.014)	
- Range	0.406 - 0.468	0.389 - 0.481	-0.030 - 0.024	
Retrolenticular part of internal capsule L				0.942
- Mean (SD)	0.440 (0.022)	0.441 (0.025)	0.001 (0.025)	
- Range	0.406 - 0.483	0.402 - 0.501	-0.044 - 0.063	
Anterior corona radiata R				0.942
- Mean (SD)	0.329 (0.028)	0.325 (0.034)	-0.003 (0.015)	
- Range	0.272 - 0.370	0.263 - 0.383	-0.028 - 0.037	
Anterior corona radiata L				0.942
- Mean (SD)	0.327 (0.026)	0.330 (0.032)	0.002 (0.018)	
- Range	0.275 - 0.364	0.270 - 0.382	-0.034 - 0.040	
Superior corona radiata R				0.942
- Mean (SD)	0.401 (0.036)	0.398 (0.035)	-0.003 (0.014)	
- Range	0.334 - 0.473	0.315 - 0.451	-0.022 - 0.024	
Superior corona radiata L				0.942
- Mean (SD)	0.400 (0.037)	0.402 (0.040)	0.002 (0.014)	
- Range	0.314 - 0.485	0.311 - 0.478	-0.029 - 0.041	
Posterior corona radiata R				0.942
- Mean (SD)	0.409 (0.040)	0.404 (0.043)	-0.005 (0.020)	
- Range	0.354 - 0.505	0.331 - 0.480	-0.033 - 0.047	
Posterior corona radiata L				0.942
- Mean (SD)	0.396 (0.046)	0.396 (0.046)	0.000 (0.019)	
- Range	0.317 - 0.490	0.308 - 0.491	-0.048 - 0.040	
Posterior thalamic radiation R				0.852
- Mean (SD)	0.451 (0.038)	0.444 (0.036)	-0.008 (0.013)	
- Range	0.370 - 0.502	0.372 - 0.491	-0.030 - 0.011	
Posterior thalamic radiation L				0.942
- Mean (SD)	0.434 (0.028)	0.438 (0.029)	0.004 (0.020)	
- Range	0.379 - 0.482	0.388 - 0.497	-0.035 - 0.030	
Sagittal stratum R				0.942
- Mean (SD)	0.425 (0.018)	0.424 (0.021)	-0.001 (0.017)	
- Range	0.394 - 0.453	0.396 - 0.455	-0.023 - 0.032	
Sagittal stratum L				0.942
- Mean (SD)	0.390 (0.018)	0.388 (0.023)	-0.002 (0.023)	
- Range	0.357 - 0.431	0.346 - 0.425	-0.038 - 0.052	
External capsule R				0.942
- Mean (SD)	0.320 (0.020)	0.321 (0.024)	0.001 (0.018)	
- Range	0.288 - 0.353	0.280 - 0.365	-0.038 - 0.037	
External capsule L				0.942
- Mean (SD)	0.331 (0.020)	0.333 (0.027)	0.002 (0.019)	
- Range	0.301 - 0.362	0.302 - 0.397	-0.051 - 0.037	
Cingulum cingulate gyrus R				0.942
- Mean (SD)	0.357 (0.025)	0.355 (0.026)	-0.001 (0.016)	
- Range	0.315 - 0.411	0.303 - 0.401	-0.030 - 0.035	
Cingulum cingulate gyrus L				0.942
- Mean (SD)	0.365 (0.023)	0.367 (0.028)	0.002 (0.014)	
- Range	0.326 - 0.414	0.310 - 0.421	-0.028 - 0.021	
Cingulum hippocampus R				0.942
- Mean (SD)	0.288 (0.035)	0.281 (0.040)	-0.008 (0.040)	
- Range	0.235 - 0.377	0.213 - 0.371	-0.086 - 0.044	
Cingulum hippocampus L				0.942
- Mean (SD)	0.282 (0.036)	0.279 (0.034)	-0.003 (0.043)	
- Range	0.202 - 0.377	0.204 - 0.326	-0.105 - 0.068	
Fornix R				0.942
- Mean (SD)	0.358 (0.025)	0.360 (0.030)	0.002 (0.025)	
- Range	0.312 - 0.408	0.319 - 0.426	-0.041 - 0.037	
Fornix L				0.942
- Mean (SD)	0.382 (0.035)	0.376 (0.033)	-0.005 (0.031)	
- Range	0.324 - 0.443	0.330 - 0.438	-0.079 - 0.042	
Superior longitudinal fasciculus R				0.942

	Post (N=18)	Pre (N=18)	Difference (N=18)	p value
- Mean (SD)	0.386 (0.030)	0.385 (0.029)	-0.001 (0.014)	
- Range	0.326 - 0.444	0.346 - 0.426	-0.021 - 0.034	
Superior longitudinal fasciculus L				0.942
- Mean (SD)	0.387 (0.031)	0.386 (0.033)	-0.001 (0.017)	
- Range	0.338 - 0.440	0.320 - 0.431	-0.052 - 0.020	
Superior fronto occipital fasciculus R				0.942
- Mean (SD)	0.346 (0.038)	0.335 (0.050)	-0.011 (0.030)	
- Range	0.256 - 0.400	0.248 - 0.441	-0.081 - 0.041	
Superior fronto occipital fasciculus L				0.942
- Mean (SD)	0.317 (0.036)	0.324 (0.043)	0.007 (0.021)	
- Range	0.254 - 0.370	0.243 - 0.383	-0.023 - 0.065	
Uncinate fasciculus R				0.942
- Mean (SD)	0.384 (0.035)	0.392 (0.037)	0.008 (0.040)	
- Range	0.344 - 0.471	0.335 - 0.462	-0.092 - 0.065	
Uncinate fasciculus L				0.942
- Mean (SD)	0.381 (0.048)	0.384 (0.039)	0.003 (0.036)	
- Range	0.305 - 0.457	0.319 - 0.439	-0.054 - 0.093	
Tapetum R				0.942
- Mean (SD)	0.330 (0.035)	0.333 (0.034)	0.003 (0.026)	
- Range	0.287 - 0.396	0.264 - 0.383	-0.039 - 0.048	
Tapetum L				0.942
- Mean (SD)	0.300 (0.027)	0.293 (0.030)	-0.007 (0.024)	
- Range	0.258 - 0.358	0.228 - 0.335	-0.047 - 0.029	

Mean diffusivity (MD, (10^-3)*(mm^2)/s) (DTI) - Offspring of lean mothers (OLM) subjects pre vs post (paired tests) - (JHU white-matter tractography atlas) - Uncorrected p-values

	Post (N=18)	Pre (N=18)	Difference (N=18)	p value
Middle cerebellar peduncle				0.123
- Mean (SD)	1.101 (0.112)	1.040 (0.088)	-0.061 (0.159)	
- Range	0.964 - 1.369	0.915 - 1.257	-0.454 - 0.211	
Pontine crossing tract				0.749
- Mean (SD)	0.811 (0.056)	0.804 (0.070)	-0.007 (0.096)	
- Range	0.738 - 0.906	0.717 - 0.975	-0.159 - 0.211	
Genu of corpus callosum				0.438
- Mean (SD)	1.372 (0.150)	1.365 (0.162)	-0.007 (0.040)	
- Range	1.066 - 1.668	1.042 - 1.688	-0.105 - 0.066	
Body of corpus callosum				0.555
- Mean (SD)	1.120 (0.086)	1.114 (0.087)	-0.006 (0.040)	
- Range	0.981 - 1.313	0.984 - 1.285	-0.075 - 0.057	
Splenium of corpus callosum				0.411
- Mean (SD)	1.005 (0.058)	1.017 (0.089)	0.012 (0.059)	
- Range	0.901 - 1.119	0.894 - 1.221	-0.061 - 0.157	
Fornix				0.539
- Mean (SD)	2.390 (0.194)	2.379 (0.225)	-0.011 (0.072)	
- Range	2.119 - 2.832	1.956 - 2.841	-0.194 - 0.092	
Corticospinal tract R				0.377
- Mean (SD)	0.852 (0.053)	0.837 (0.070)	-0.015 (0.070)	
- Range	0.771 - 0.922	0.710 - 1.012	-0.123 - 0.159	
Corticospinal tract L				0.939
- Mean (SD)	0.821 (0.067)	0.823 (0.069)	0.001 (0.073)	
- Range	0.727 - 0.933	0.720 - 0.951	-0.145 - 0.136	
Medial lemniscus R				0.689
- Mean (SD)	0.873 (0.048)	0.883 (0.107)	0.010 (0.105)	
- Range	0.758 - 0.948	0.742 - 1.165	-0.131 - 0.284	
Medial lemniscus L				0.519
- Mean (SD)	0.864 (0.055)	0.855 (0.054)	-0.009 (0.058)	
- Range	0.755 - 0.996	0.744 - 0.981	-0.121 - 0.107	
Inferior cerebellar peduncle R				0.402
- Mean (SD)	1.158 (0.241)	1.090 (0.215)	-0.068 (0.335)	
- Range	0.915 - 1.926	0.931 - 1.875	-0.953 - 0.854	
Inferior cerebellar peduncle L				0.482
- Mean (SD)	1.069 (0.115)	1.039 (0.124)	-0.030 (0.175)	
- Range	0.854 - 1.291	0.885 - 1.363	-0.406 - 0.509	
Superior cerebellar peduncle R				0.750
- Mean (SD)	1.231 (0.169)	1.244 (0.140)	0.013 (0.167)	
- Range	0.981 - 1.530	1.013 - 1.472	-0.290 - 0.428	
Superior cerebellar peduncle L				0.266
- Mean (SD)	1.263 (0.167)	1.304 (0.164)	0.041 (0.149)	
- Range	0.988 - 1.650	1.064 - 1.618	-0.248 - 0.341	
Cerebral peduncle R				0.982
- Mean (SD)	0.881 (0.056)	0.881 (0.044)	-0.000 (0.042)	
- Range	0.783 - 1.016	0.785 - 0.951	-0.135 - 0.051	
Cerebral peduncle L				0.446
- Mean (SD)	0.840 (0.047)	0.847 (0.046)	0.007 (0.039)	
- Range	0.749 - 0.915	0.733 - 0.926	-0.087 - 0.071	
Anterior limb of internal capsule R				0.439
- Mean (SD)	0.871 (0.066)	0.865 (0.071)	-0.005 (0.029)	
- Range	0.789 - 1.007	0.774 - 1.020	-0.103 - 0.027	
Anterior limb of internal capsule L				0.694
- Mean (SD)	0.873 (0.075)	0.878 (0.079)	0.005 (0.052)	
- Range	0.765 - 1.022	0.772 - 1.040	-0.044 - 0.195	
Posterior limb of internal capsule R				0.858
- Mean (SD)	0.772 (0.033)	0.771 (0.039)	-0.001 (0.023)	
- Range	0.717 - 0.832	0.718 - 0.857	-0.044 - 0.046	
Posterior limb of internal capsule L				0.195
- Mean (SD)	0.760 (0.035)	0.767 (0.037)	0.008 (0.024)	

	Post (N=18)	Pre (N=18)	Difference (N=18)	p value
- Range	0.711 - 0.831	0.703 - 0.847	-0.023 - 0.083	
Retrolenticular part of internal capsule R				0.036
- Mean (SD)	0.926 (0.052)	0.911 (0.053)	-0.015 (0.028)	
- Range	0.838 - 1.053	0.820 - 1.009	-0.073 - 0.030	
Retrolenticular part of internal capsule L				0.302
- Mean (SD)	0.923 (0.060)	0.932 (0.065)	0.009 (0.037)	
- Range	0.852 - 1.114	0.822 - 1.088	-0.030 - 0.119	
Anterior corona radiata R				0.757
- Mean (SD)	0.968 (0.107)	0.970 (0.105)	0.002 (0.023)	
- Range	0.806 - 1.194	0.790 - 1.160	-0.034 - 0.042	
Anterior corona radiata L				0.412
- Mean (SD)	0.960 (0.108)	0.970 (0.128)	0.010 (0.048)	
- Range	0.775 - 1.158	0.791 - 1.298	-0.027 - 0.176	
Superior corona radiata R				0.192
- Mean (SD)	0.838 (0.094)	0.831 (0.086)	-0.008 (0.024)	
- Range	0.724 - 1.109	0.708 - 1.096	-0.072 - 0.038	
Superior corona radiata L				0.833
- Mean (SD)	0.848 (0.079)	0.847 (0.080)	-0.001 (0.027)	
- Range	0.747 - 1.069	0.705 - 1.052	-0.044 - 0.058	
Posterior corona radiata R				0.277
- Mean (SD)	0.978 (0.098)	0.963 (0.077)	-0.015 (0.056)	
- Range	0.845 - 1.181	0.852 - 1.178	-0.131 - 0.093	
Posterior corona radiata L				0.508
- Mean (SD)	0.993 (0.096)	1.002 (0.101)	0.009 (0.058)	
- Range	0.838 - 1.160	0.813 - 1.192	-0.091 - 0.159	
Posterior thalamic radiation R				0.555
- Mean (SD)	1.032 (0.143)	1.022 (0.098)	-0.010 (0.068)	
- Range	0.857 - 1.455	0.874 - 1.248	-0.207 - 0.135	
Posterior thalamic radiation L				0.589
- Mean (SD)	1.167 (0.257)	1.150 (0.170)	-0.017 (0.130)	
- Range	0.965 - 2.104	0.934 - 1.606	-0.498 - 0.130	
Sagittal stratum R				0.107
- Mean (SD)	1.009 (0.083)	0.991 (0.067)	-0.018 (0.046)	
- Range	0.868 - 1.208	0.880 - 1.091	-0.156 - 0.035	
Sagittal stratum L				0.204
- Mean (SD)	1.105 (0.165)	1.079 (0.121)	-0.026 (0.084)	
- Range	0.910 - 1.571	0.881 - 1.305	-0.317 - 0.085	
External capsule R				0.588
- Mean (SD)	0.869 (0.063)	0.872 (0.060)	0.003 (0.025)	
- Range	0.785 - 1.012	0.784 - 0.996	-0.040 - 0.069	
External capsule L				0.412
- Mean (SD)	0.851 (0.053)	0.844 (0.060)	-0.007 (0.033)	
- Range	0.771 - 0.955	0.765 - 0.953	-0.058 - 0.072	
Cingulum cingulate gyrus R				0.822
- Mean (SD)	0.902 (0.077)	0.904 (0.070)	0.002 (0.031)	
- Range	0.770 - 1.073	0.792 - 1.082	-0.063 - 0.058	
Cingulum cingulate gyrus L				0.111
- Mean (SD)	0.935 (0.063)	0.948 (0.080)	0.013 (0.032)	
- Range	0.860 - 1.047	0.828 - 1.115	-0.041 - 0.087	
Cingulum hippocampus R				0.199
- Mean (SD)	1.079 (0.100)	1.043 (0.092)	-0.035 (0.112)	
- Range	0.929 - 1.219	0.887 - 1.212	-0.185 - 0.257	
Cingulum hippocampus L				0.039
- Mean (SD)	1.135 (0.088)	1.073 (0.100)	-0.062 (0.117)	
- Range	0.994 - 1.307	0.897 - 1.236	-0.297 - 0.084	
Fornix R				0.544
- Mean (SD)	1.081 (0.106)	1.087 (0.117)	0.006 (0.040)	
- Range	0.847 - 1.267	0.844 - 1.309	-0.072 - 0.085	
Fornix L				0.706
- Mean (SD)	1.035 (0.095)	1.040 (0.110)	0.005 (0.053)	
- Range	0.811 - 1.211	0.841 - 1.278	-0.097 - 0.091	
Superior longitudinal fasciculus R				0.227

	Post (N=18)	Pre (N=18)	Difference (N=18)	p value
- Mean (SD)	0.813 (0.064)	0.805 (0.054)	-0.009 (0.029)	
- Range	0.734 - 0.967	0.740 - 0.950	-0.070 - 0.031	
Superior longitudinal fasciculus L				0.540
- Mean (SD)	0.803 (0.053)	0.799 (0.048)	-0.004 (0.027)	
- Range	0.705 - 0.904	0.730 - 0.904	-0.054 - 0.052	
Superior fronto occipital fasciculus R				0.228
- Mean (SD)	0.988 (0.190)	0.969 (0.203)	-0.019 (0.066)	
- Range	0.748 - 1.503	0.723 - 1.560	-0.195 - 0.100	
Superior fronto occipital fasciculus L				0.765
- Mean (SD)	1.095 (0.199)	1.103 (0.243)	0.008 (0.111)	
- Range	0.802 - 1.457	0.819 - 1.744	-0.151 - 0.329	
Uncinate fasciculus R				0.496
- Mean (SD)	0.880 (0.049)	0.896 (0.096)	0.016 (0.099)	
- Range	0.763 - 0.948	0.797 - 1.189	-0.099 - 0.291	
Uncinate fasciculus L				0.122
- Mean (SD)	0.944 (0.186)	0.888 (0.121)	-0.056 (0.147)	
- Range	0.794 - 1.444	0.780 - 1.287	-0.443 - 0.223	
Tapetum R				0.433
- Mean (SD)	2.095 (0.304)	2.137 (0.350)	0.042 (0.221)	
- Range	1.554 - 2.657	1.528 - 2.681	-0.307 - 0.440	
Tapetum L				0.281
- Mean (SD)	2.336 (0.287)	2.398 (0.376)	0.062 (0.237)	
- Range	1.890 - 2.834	1.678 - 2.936	-0.346 - 0.557	

Mean diffusivity (MD, (10^-3)*(mm^2)/s) (DTI) - Offspring of lean mothers (OLM) subjects pre vs post (paired tests) - (JHU white-matter tractography atlas) - FDR corrected p-values

	Post (N=18)	Pre (N=18)	Difference (N=18)	p value
Middle cerebellar peduncle				0.783
- Mean (SD)	1.101 (0.112)	1.040 (0.088)	-0.061 (0.159)	
- Range	0.964 - 1.369	0.915 - 1.257	-0.454 - 0.211	
Pontine crossing tract				0.853
- Mean (SD)	0.811 (0.056)	0.804 (0.070)	-0.007 (0.096)	
- Range	0.738 - 0.906	0.717 - 0.975	-0.159 - 0.211	
Genu of corpus callosum				0.783
- Mean (SD)	1.372 (0.150)	1.365 (0.162)	-0.007 (0.040)	
- Range	1.066 - 1.668	1.042 - 1.688	-0.105 - 0.066	
Body of corpus callosum				0.783
- Mean (SD)	1.120 (0.086)	1.114 (0.087)	-0.006 (0.040)	
- Range	0.981 - 1.313	0.984 - 1.285	-0.075 - 0.057	
Splenium of corpus callosum				0.783
- Mean (SD)	1.005 (0.058)	1.017 (0.089)	0.012 (0.059)	
- Range	0.901 - 1.119	0.894 - 1.221	-0.061 - 0.157	
Fornix				0.783
- Mean (SD)	2.390 (0.194)	2.379 (0.225)	-0.011 (0.072)	
- Range	2.119 - 2.832	1.956 - 2.841	-0.194 - 0.092	
Corticospinal tract R				0.783
- Mean (SD)	0.852 (0.053)	0.837 (0.070)	-0.015 (0.070)	
- Range	0.771 - 0.922	0.710 - 1.012	-0.123 - 0.159	
Corticospinal tract L				0.959
- Mean (SD)	0.821 (0.067)	0.823 (0.069)	0.001 (0.073)	
- Range	0.727 - 0.933	0.720 - 0.951	-0.145 - 0.136	
Medial lemniscus R				0.853
- Mean (SD)	0.873 (0.048)	0.883 (0.107)	0.010 (0.105)	
- Range	0.758 - 0.948	0.742 - 1.165	-0.131 - 0.284	
Medial lemniscus L				0.783
- Mean (SD)	0.864 (0.055)	0.855 (0.054)	-0.009 (0.058)	
- Range	0.755 - 0.996	0.744 - 0.981	-0.121 - 0.107	
Inferior cerebellar peduncle R				0.783
- Mean (SD)	1.158 (0.241)	1.090 (0.215)	-0.068 (0.335)	
- Range	0.915 - 1.926	0.931 - 1.875	-0.953 - 0.854	
Inferior cerebellar peduncle L				0.783
- Mean (SD)	1.069 (0.115)	1.039 (0.124)	-0.030 (0.175)	
- Range	0.854 - 1.291	0.885 - 1.363	-0.406 - 0.509	
Superior cerebellar peduncle R				0.853
- Mean (SD)	1.231 (0.169)	1.244 (0.140)	0.013 (0.167)	
- Range	0.981 - 1.530	1.013 - 1.472	-0.290 - 0.428	
Superior cerebellar peduncle L				0.783
- Mean (SD)	1.263 (0.167)	1.304 (0.164)	0.041 (0.149)	
- Range	0.988 - 1.650	1.064 - 1.618	-0.248 - 0.341	
Cerebral peduncle R				0.982
- Mean (SD)	0.881 (0.056)	0.881 (0.044)	-0.000 (0.042)	
- Range	0.783 - 1.016	0.785 - 0.951	-0.135 - 0.051	
Cerebral peduncle L				0.783
- Mean (SD)	0.840 (0.047)	0.847 (0.046)	0.007 (0.039)	
- Range	0.749 - 0.915	0.733 - 0.926	-0.087 - 0.071	
Anterior limb of internal capsule R				0.783
- Mean (SD)	0.871 (0.066)	0.865 (0.071)	-0.005 (0.029)	
- Range	0.789 - 1.007	0.774 - 1.020	-0.103 - 0.027	
Anterior limb of internal capsule L				0.853
- Mean (SD)	0.873 (0.075)	0.878 (0.079)	0.005 (0.052)	
- Range	0.765 - 1.022	0.772 - 1.040	-0.044 - 0.195	
Posterior limb of internal capsule R				0.895
- Mean (SD)	0.772 (0.033)	0.771 (0.039)	-0.001 (0.023)	
- Range	0.717 - 0.832	0.718 - 0.857	-0.044 - 0.046	
Posterior limb of internal capsule L				0.783
- Mean (SD)	0.760 (0.035)	0.767 (0.037)	0.008 (0.024)	

	Post (N=18)	Pre (N=18)	Difference (N=18)	p value
- Range	0.711 - 0.831	0.703 - 0.847	-0.023 - 0.083	
Retrolenticular part of internal capsule R				0.783
- Mean (SD)	0.926 (0.052)	0.911 (0.053)	-0.015 (0.028)	
- Range	0.838 - 1.053	0.820 - 1.009	-0.073 - 0.030	
Retrolenticular part of internal capsule L				0.783
- Mean (SD)	0.923 (0.060)	0.932 (0.065)	0.009 (0.037)	
- Range	0.852 - 1.114	0.822 - 1.088	-0.030 - 0.119	
Anterior corona radiata R				0.853
- Mean (SD)	0.968 (0.107)	0.970 (0.105)	0.002 (0.023)	
- Range	0.806 - 1.194	0.790 - 1.160	-0.034 - 0.042	
Anterior corona radiata L				0.783
- Mean (SD)	0.960 (0.108)	0.970 (0.128)	0.010 (0.048)	
- Range	0.775 - 1.158	0.791 - 1.298	-0.027 - 0.176	
Superior corona radiata R				0.783
- Mean (SD)	0.838 (0.094)	0.831 (0.086)	-0.008 (0.024)	
- Range	0.724 - 1.109	0.708 - 1.096	-0.072 - 0.038	
Superior corona radiata L				0.888
- Mean (SD)	0.848 (0.079)	0.847 (0.080)	-0.001 (0.027)	
- Range	0.747 - 1.069	0.705 - 1.052	-0.044 - 0.058	
Posterior corona radiata R				0.783
- Mean (SD)	0.978 (0.098)	0.963 (0.077)	-0.015 (0.056)	
- Range	0.845 - 1.181	0.852 - 1.178	-0.131 - 0.093	
Posterior corona radiata L				0.783
- Mean (SD)	0.993 (0.096)	1.002 (0.101)	0.009 (0.058)	
- Range	0.838 - 1.160	0.813 - 1.192	-0.091 - 0.159	
Posterior thalamic radiation R				0.783
- Mean (SD)	1.032 (0.143)	1.022 (0.098)	-0.010 (0.068)	
- Range	0.857 - 1.455	0.874 - 1.248	-0.207 - 0.135	
Posterior thalamic radiation L				0.785
- Mean (SD)	1.167 (0.257)	1.150 (0.170)	-0.017 (0.130)	
- Range	0.965 - 2.104	0.934 - 1.606	-0.498 - 0.130	
Sagittal stratum R				0.783
- Mean (SD)	1.009 (0.083)	0.991 (0.067)	-0.018 (0.046)	
- Range	0.868 - 1.208	0.880 - 1.091	-0.156 - 0.035	
Sagittal stratum L				0.783
- Mean (SD)	1.105 (0.165)	1.079 (0.121)	-0.026 (0.084)	
- Range	0.910 - 1.571	0.881 - 1.305	-0.317 - 0.085	
External capsule R				0.785
- Mean (SD)	0.869 (0.063)	0.872 (0.060)	0.003 (0.025)	
- Range	0.785 - 1.012	0.784 - 0.996	-0.040 - 0.069	
External capsule L				0.783
- Mean (SD)	0.851 (0.053)	0.844 (0.060)	-0.007 (0.033)	
- Range	0.771 - 0.955	0.765 - 0.953	-0.058 - 0.072	
Cingulum cingulate gyrus R				0.888
- Mean (SD)	0.902 (0.077)	0.904 (0.070)	0.002 (0.031)	
- Range	0.770 - 1.073	0.792 - 1.082	-0.063 - 0.058	
Cingulum cingulate gyrus L				0.783
- Mean (SD)	0.935 (0.063)	0.948 (0.080)	0.013 (0.032)	
- Range	0.860 - 1.047	0.828 - 1.115	-0.041 - 0.087	
Cingulum hippocampus R				0.783
- Mean (SD)	1.079 (0.100)	1.043 (0.092)	-0.035 (0.112)	
- Range	0.929 - 1.219	0.887 - 1.212	-0.185 - 0.257	
Cingulum hippocampus L				0.783
- Mean (SD)	1.135 (0.088)	1.073 (0.100)	-0.062 (0.117)	
- Range	0.994 - 1.307	0.897 - 1.236	-0.297 - 0.084	
Fornix R				0.783
- Mean (SD)	1.081 (0.106)	1.087 (0.117)	0.006 (0.040)	
- Range	0.847 - 1.267	0.844 - 1.309	-0.072 - 0.085	
Fornix L				0.853
- Mean (SD)	1.035 (0.095)	1.040 (0.110)	0.005 (0.053)	
- Range	0.811 - 1.211	0.841 - 1.278	-0.097 - 0.091	
Superior longitudinal fasciculus R				0.783

	Post (N=18)	Pre (N=18)	Difference (N=18)	p value
- Mean (SD)	0.813 (0.064)	0.805 (0.054)	-0.009 (0.029)	
- Range	0.734 - 0.967	0.740 - 0.950	-0.070 - 0.031	
Superior longitudinal fasciculus L				0.783
- Mean (SD)	0.803 (0.053)	0.799 (0.048)	-0.004 (0.027)	
- Range	0.705 - 0.904	0.730 - 0.904	-0.054 - 0.052	
Superior fronto occipital fasciculus R				0.783
- Mean (SD)	0.988 (0.190)	0.969 (0.203)	-0.019 (0.066)	
- Range	0.748 - 1.503	0.723 - 1.560	-0.195 - 0.100	
Superior fronto occipital fasciculus L				0.853
- Mean (SD)	1.095 (0.199)	1.103 (0.243)	0.008 (0.111)	
- Range	0.802 - 1.457	0.819 - 1.744	-0.151 - 0.329	
Uncinate fasciculus R				0.783
- Mean (SD)	0.880 (0.049)	0.896 (0.096)	0.016 (0.099)	
- Range	0.763 - 0.948	0.797 - 1.189	-0.099 - 0.291	
Uncinate fasciculus L				0.783
- Mean (SD)	0.944 (0.186)	0.888 (0.121)	-0.056 (0.147)	
- Range	0.794 - 1.444	0.780 - 1.287	-0.443 - 0.223	
Tapetum R				0.783
- Mean (SD)	2.095 (0.304)	2.137 (0.350)	0.042 (0.221)	
- Range	1.554 - 2.657	1.528 - 2.681	-0.307 - 0.440	
Tapetum L				0.783
- Mean (SD)	2.336 (0.287)	2.398 (0.376)	0.062 (0.237)	
- Range	1.890 - 2.834	1.678 - 2.936	-0.346 - 0.557	

Radial diffusivity (RD, (10^-3)*(mm^2)/s) (DTI) - Offspring of lean mothers (OLM) subjects pre vs post (paired tests) - (JHU white-matter tractography atlas) - Uncorrected p-values

	Post (N=18)	Pre (N=18)	Difference (N=18)	p value
Middle cerebellar peduncle				0.173
- Mean (SD)	0.873 (0.109)	0.817 (0.095)	-0.057 (0.169)	
- Range	0.737 - 1.092	0.682 - 1.038	-0.410 - 0.264	
Pontine crossing tract				0.537
- Mean (SD)	0.644 (0.055)	0.630 (0.065)	-0.014 (0.094)	
- Range	0.545 - 0.730	0.528 - 0.792	-0.144 - 0.247	
Genu of corpus callosum				0.472
- Mean (SD)	1.058 (0.133)	1.054 (0.147)	-0.004 (0.026)	
- Range	0.782 - 1.317	0.755 - 1.335	-0.051 - 0.051	
Body of corpus callosum				0.454
- Mean (SD)	0.812 (0.091)	0.806 (0.090)	-0.006 (0.033)	
- Range	0.667 - 1.007	0.683 - 0.974	-0.062 - 0.048	
Splenium of corpus callosum				0.544
- Mean (SD)	0.641 (0.065)	0.648 (0.083)	0.007 (0.048)	
- Range	0.516 - 0.730	0.497 - 0.822	-0.057 - 0.121	
Fornix				0.165
- Mean (SD)	2.094 (0.203)	2.067 (0.224)	-0.026 (0.076)	
- Range	1.795 - 2.540	1.678 - 2.567	-0.184 - 0.082	
Corticospinal tract R				0.225
- Mean (SD)	0.634 (0.049)	0.617 (0.060)	-0.018 (0.060)	
- Range	0.550 - 0.709	0.523 - 0.787	-0.129 - 0.107	
Corticospinal tract L				0.955
- Mean (SD)	0.596 (0.056)	0.597 (0.056)	0.001 (0.062)	
- Range	0.522 - 0.743	0.488 - 0.697	-0.134 - 0.098	
Medial lemniscus R				0.554
- Mean (SD)	0.645 (0.052)	0.657 (0.094)	0.012 (0.085)	
- Range	0.570 - 0.743	0.515 - 0.953	-0.128 - 0.210	
Medial lemniscus L				0.892
- Mean (SD)	0.638 (0.058)	0.636 (0.059)	-0.002 (0.062)	
- Range	0.546 - 0.777	0.523 - 0.791	-0.095 - 0.108	
Inferior cerebellar peduncle R				0.419
- Mean (SD)	0.952 (0.211)	0.893 (0.199)	-0.059 (0.304)	
- Range	0.744 - 1.601	0.761 - 1.625	-0.825 - 0.786	
Inferior cerebellar peduncle L				0.437
- Mean (SD)	0.875 (0.107)	0.845 (0.113)	-0.030 (0.162)	
- Range	0.684 - 1.072	0.687 - 1.125	-0.385 - 0.441	
Superior cerebellar peduncle R				0.925
- Mean (SD)	0.922 (0.159)	0.926 (0.151)	0.005 (0.202)	
- Range	0.728 - 1.196	0.687 - 1.291	-0.365 - 0.563	
Superior cerebellar peduncle L				0.410
- Mean (SD)	0.963 (0.141)	0.997 (0.157)	0.033 (0.168)	
- Range	0.686 - 1.281	0.757 - 1.288	-0.331 - 0.457	
Cerebral peduncle R				0.636
- Mean (SD)	0.584 (0.050)	0.588 (0.040)	0.004 (0.036)	
- Range	0.515 - 0.703	0.512 - 0.662	-0.096 - 0.054	
Cerebral peduncle L				0.258
- Mean (SD)	0.541 (0.041)	0.549 (0.042)	0.008 (0.028)	
- Range	0.473 - 0.611	0.473 - 0.623	-0.039 - 0.068	
Anterior limb of internal capsule R				0.836
- Mean (SD)	0.651 (0.069)	0.649 (0.074)	-0.001 (0.026)	
- Range	0.570 - 0.812	0.568 - 0.837	-0.082 - 0.037	
Anterior limb of internal capsule L				0.825
- Mean (SD)	0.649 (0.072)	0.652 (0.081)	0.003 (0.049)	
- Range	0.536 - 0.785	0.556 - 0.820	-0.045 - 0.181	
Posterior limb of internal capsule R				0.682
- Mean (SD)	0.519 (0.033)	0.521 (0.041)	0.002 (0.020)	
- Range	0.473 - 0.574	0.465 - 0.600	-0.048 - 0.034	
Posterior limb of internal capsule L				0.297
- Mean (SD)	0.507 (0.032)	0.512 (0.038)	0.005 (0.022)	

	Post (N=18)	Pre (N=18)	Difference (N=18)	p value
- Range	0.456 - 0.578	0.448 - 0.603	-0.020 - 0.074	
Retrolenticular part of internal capsule R				0.138
- Mean (SD)	0.698 (0.049)	0.688 (0.054)	-0.009 (0.025)	
- Range	0.609 - 0.792	0.589 - 0.781	-0.052 - 0.041	
Retrolenticular part of internal capsule L				0.577
- Mean (SD)	0.693 (0.056)	0.698 (0.056)	0.005 (0.038)	
- Range	0.621 - 0.865	0.605 - 0.826	-0.039 - 0.121	
Anterior corona radiata R				0.294
- Mean (SD)	0.796 (0.099)	0.801 (0.102)	0.004 (0.017)	
- Range	0.636 - 0.989	0.625 - 0.983	-0.029 - 0.033	
Anterior corona radiata L				0.488
- Mean (SD)	0.790 (0.100)	0.797 (0.117)	0.007 (0.042)	
- Range	0.615 - 0.952	0.624 - 1.070	-0.038 - 0.152	
Superior corona radiata R				0.430
- Mean (SD)	0.649 (0.088)	0.645 (0.084)	-0.003 (0.018)	
- Range	0.552 - 0.904	0.544 - 0.907	-0.049 - 0.024	
Superior corona radiata L				0.870
- Mean (SD)	0.659 (0.076)	0.658 (0.076)	-0.001 (0.021)	
- Range	0.576 - 0.896	0.546 - 0.881	-0.036 - 0.044	
Posterior corona radiata R				0.378
- Mean (SD)	0.750 (0.087)	0.742 (0.077)	-0.008 (0.038)	
- Range	0.625 - 0.941	0.649 - 0.964	-0.088 - 0.043	
Posterior corona radiata L				0.481
- Mean (SD)	0.775 (0.086)	0.782 (0.086)	0.008 (0.045)	
- Range	0.627 - 0.959	0.620 - 0.995	-0.069 - 0.139	
Posterior thalamic radiation R				0.897
- Mean (SD)	0.765 (0.111)	0.763 (0.080)	-0.002 (0.050)	
- Range	0.617 - 1.044	0.641 - 0.911	-0.133 - 0.108	
Posterior thalamic radiation L				0.529
- Mean (SD)	0.888 (0.215)	0.872 (0.143)	-0.016 (0.106)	
- Range	0.704 - 1.665	0.661 - 1.258	-0.407 - 0.084	
Sagittal stratum R				0.179
- Mean (SD)	0.769 (0.070)	0.756 (0.057)	-0.014 (0.042)	
- Range	0.643 - 0.920	0.664 - 0.850	-0.139 - 0.041	
Sagittal stratum L				0.217
- Mean (SD)	0.870 (0.136)	0.850 (0.104)	-0.020 (0.064)	
- Range	0.692 - 1.230	0.686 - 1.037	-0.219 - 0.059	
External capsule R				0.665
- Mean (SD)	0.718 (0.064)	0.721 (0.061)	0.003 (0.026)	
- Range	0.647 - 0.861	0.637 - 0.838	-0.027 - 0.076	
External capsule L				0.411
- Mean (SD)	0.697 (0.049)	0.691 (0.060)	-0.006 (0.029)	
- Range	0.631 - 0.771	0.613 - 0.795	-0.052 - 0.063	
Cingulum cingulate gyrus R				0.786
- Mean (SD)	0.726 (0.070)	0.728 (0.067)	0.002 (0.033)	
- Range	0.621 - 0.880	0.640 - 0.915	-0.072 - 0.064	
Cingulum cingulate gyrus L				0.184
- Mean (SD)	0.745 (0.059)	0.754 (0.070)	0.009 (0.027)	
- Range	0.666 - 0.865	0.655 - 0.891	-0.032 - 0.068	
Cingulum hippocampus R				0.386
- Mean (SD)	0.919 (0.097)	0.897 (0.097)	-0.022 (0.104)	
- Range	0.765 - 1.050	0.715 - 1.079	-0.178 - 0.261	
Cingulum hippocampus L				0.073
- Mean (SD)	0.973 (0.093)	0.922 (0.096)	-0.051 (0.113)	
- Range	0.810 - 1.159	0.780 - 1.097	-0.266 - 0.094	
Fornix R				0.669
- Mean (SD)	0.876 (0.097)	0.880 (0.113)	0.004 (0.038)	
- Range	0.650 - 1.042	0.642 - 1.087	-0.040 - 0.077	
Fornix L				0.530
- Mean (SD)	0.826 (0.087)	0.833 (0.101)	0.007 (0.046)	
- Range	0.623 - 0.970	0.648 - 1.065	-0.088 - 0.095	
Superior longitudinal fasciculus R				0.313

	Post (N=18)	Pre (N=18)	Difference (N=18)	p value
- Mean (SD)	0.642 (0.067)	0.635 (0.054)	-0.008 (0.031)	
- Range	0.567 - 0.809	0.571 - 0.766	-0.082 - 0.034	
Superior longitudinal fasciculus L				0.583
- Mean (SD)	0.633 (0.052)	0.630 (0.052)	-0.003 (0.022)	
- Range	0.532 - 0.711	0.575 - 0.730	-0.046 - 0.043	
Superior fronto occipital fasciculus R				0.557
- Mean (SD)	0.808 (0.176)	0.799 (0.199)	-0.009 (0.065)	
- Range	0.578 - 1.254	0.561 - 1.371	-0.140 - 0.117	
Superior fronto occipital fasciculus L				0.936
- Mean (SD)	0.919 (0.192)	0.921 (0.232)	0.002 (0.101)	
- Range	0.651 - 1.243	0.644 - 1.533	-0.126 - 0.290	
Uncinate fasciculus R				0.744
- Mean (SD)	0.693 (0.055)	0.700 (0.091)	0.007 (0.090)	
- Range	0.557 - 0.753	0.598 - 0.957	-0.078 - 0.234	
Uncinate fasciculus L				0.138
- Mean (SD)	0.755 (0.183)	0.703 (0.113)	-0.053 (0.144)	
- Range	0.595 - 1.225	0.590 - 1.063	-0.451 - 0.214	
Tapetum R				0.449
- Mean (SD)	1.748 (0.272)	1.779 (0.295)	0.030 (0.166)	
- Range	1.237 - 2.245	1.231 - 2.218	-0.230 - 0.311	
Tapetum L				0.172
- Mean (SD)	1.996 (0.247)	2.062 (0.329)	0.066 (0.196)	
- Range	1.570 - 2.375	1.405 - 2.503	-0.239 - 0.490	

Radial diffusivity (RD, (10^-3)*(mm^2)/s) (DTI) - Offspring of lean mothers (OLM) subjects pre vs post (paired tests) - (JHU white-matter tractography atlas) - FDR corrected p-values

	Post (N=18)	Pre (N=18)	Difference (N=18)	p value
Middle cerebellar peduncle				0.823
- Mean (SD)	0.873 (0.109)	0.817 (0.095)	-0.057 (0.169)	
- Range	0.737 - 1.092	0.682 - 1.038	-0.410 - 0.264	
Pontine crossing tract				0.823
- Mean (SD)	0.644 (0.055)	0.630 (0.065)	-0.014 (0.094)	
- Range	0.545 - 0.730	0.528 - 0.792	-0.144 - 0.247	
Genu of corpus callosum				0.823
- Mean (SD)	1.058 (0.133)	1.054 (0.147)	-0.004 (0.026)	
- Range	0.782 - 1.317	0.755 - 1.335	-0.051 - 0.051	
Body of corpus callosum				0.823
- Mean (SD)	0.812 (0.091)	0.806 (0.090)	-0.006 (0.033)	
- Range	0.667 - 1.007	0.683 - 0.974	-0.062 - 0.048	
Splenium of corpus callosum				0.823
- Mean (SD)	0.641 (0.065)	0.648 (0.083)	0.007 (0.048)	
- Range	0.516 - 0.730	0.497 - 0.822	-0.057 - 0.121	
Fornix				0.823
- Mean (SD)	2.094 (0.203)	2.067 (0.224)	-0.026 (0.076)	
- Range	1.795 - 2.540	1.678 - 2.567	-0.184 - 0.082	
Corticospinal tract R				0.823
- Mean (SD)	0.634 (0.049)	0.617 (0.060)	-0.018 (0.060)	
- Range	0.550 - 0.709	0.523 - 0.787	-0.129 - 0.107	
Corticospinal tract L				0.955
- Mean (SD)	0.596 (0.056)	0.597 (0.056)	0.001 (0.062)	
- Range	0.522 - 0.743	0.488 - 0.697	-0.134 - 0.098	
Medial lemniscus R				0.823
- Mean (SD)	0.645 (0.052)	0.657 (0.094)	0.012 (0.085)	
- Range	0.570 - 0.743	0.515 - 0.953	-0.128 - 0.210	
Medial lemniscus L				0.955
- Mean (SD)	0.638 (0.058)	0.636 (0.059)	-0.002 (0.062)	
- Range	0.546 - 0.777	0.523 - 0.791	-0.095 - 0.108	
Inferior cerebellar peduncle R				0.823
- Mean (SD)	0.952 (0.211)	0.893 (0.199)	-0.059 (0.304)	
- Range	0.744 - 1.601	0.761 - 1.625	-0.825 - 0.786	
Inferior cerebellar peduncle L				0.823
- Mean (SD)	0.875 (0.107)	0.845 (0.113)	-0.030 (0.162)	
- Range	0.684 - 1.072	0.687 - 1.125	-0.385 - 0.441	
Superior cerebellar peduncle R				0.955
- Mean (SD)	0.922 (0.159)	0.926 (0.151)	0.005 (0.202)	
- Range	0.728 - 1.196	0.687 - 1.291	-0.365 - 0.563	
Superior cerebellar peduncle L				0.823
- Mean (SD)	0.963 (0.141)	0.997 (0.157)	0.033 (0.168)	
- Range	0.686 - 1.281	0.757 - 1.288	-0.331 - 0.457	
Cerebral peduncle R				0.861
- Mean (SD)	0.584 (0.050)	0.588 (0.040)	0.004 (0.036)	
- Range	0.515 - 0.703	0.512 - 0.662	-0.096 - 0.054	
Cerebral peduncle L				0.823
- Mean (SD)	0.541 (0.041)	0.549 (0.042)	0.008 (0.028)	
- Range	0.473 - 0.611	0.473 - 0.623	-0.039 - 0.068	
Anterior limb of internal capsule R				0.955
- Mean (SD)	0.651 (0.069)	0.649 (0.074)	-0.001 (0.026)	
- Range	0.570 - 0.812	0.568 - 0.837	-0.082 - 0.037	
Anterior limb of internal capsule L				0.955
- Mean (SD)	0.649 (0.072)	0.652 (0.081)	0.003 (0.049)	
- Range	0.536 - 0.785	0.556 - 0.820	-0.045 - 0.181	
Posterior limb of internal capsule R				0.861
- Mean (SD)	0.519 (0.033)	0.521 (0.041)	0.002 (0.020)	
- Range	0.473 - 0.574	0.465 - 0.600	-0.048 - 0.034	
Posterior limb of internal capsule L				0.823
- Mean (SD)	0.507 (0.032)	0.512 (0.038)	0.005 (0.022)	

	Post (N=18)	Pre (N=18)	Difference (N=18)	p value
- Range	0.456 - 0.578	0.448 - 0.603	-0.020 - 0.074	
Retrolenticular part of internal capsule R				0.823
- Mean (SD)	0.698 (0.049)	0.688 (0.054)	-0.009 (0.025)	
- Range	0.609 - 0.792	0.589 - 0.781	-0.052 - 0.041	
Retrolenticular part of internal capsule L				0.823
- Mean (SD)	0.693 (0.056)	0.698 (0.056)	0.005 (0.038)	
- Range	0.621 - 0.865	0.605 - 0.826	-0.039 - 0.121	
Anterior corona radiata R				0.823
- Mean (SD)	0.796 (0.099)	0.801 (0.102)	0.004 (0.017)	
- Range	0.636 - 0.989	0.625 - 0.983	-0.029 - 0.033	
Anterior corona radiata L				0.823
- Mean (SD)	0.790 (0.100)	0.797 (0.117)	0.007 (0.042)	
- Range	0.615 - 0.952	0.624 - 1.070	-0.038 - 0.152	
Superior corona radiata R				0.823
- Mean (SD)	0.649 (0.088)	0.645 (0.084)	-0.003 (0.018)	
- Range	0.552 - 0.904	0.544 - 0.907	-0.049 - 0.024	
Superior corona radiata L				0.955
- Mean (SD)	0.659 (0.076)	0.658 (0.076)	-0.001 (0.021)	
- Range	0.576 - 0.896	0.546 - 0.881	-0.036 - 0.044	
Posterior corona radiata R				0.823
- Mean (SD)	0.750 (0.087)	0.742 (0.077)	-0.008 (0.038)	
- Range	0.625 - 0.941	0.649 - 0.964	-0.088 - 0.043	
Posterior corona radiata L				0.823
- Mean (SD)	0.775 (0.086)	0.782 (0.086)	0.008 (0.045)	
- Range	0.627 - 0.959	0.620 - 0.995	-0.069 - 0.139	
Posterior thalamic radiation R				0.955
- Mean (SD)	0.765 (0.111)	0.763 (0.080)	-0.002 (0.050)	
- Range	0.617 - 1.044	0.641 - 0.911	-0.133 - 0.108	
Posterior thalamic radiation L				0.823
- Mean (SD)	0.888 (0.215)	0.872 (0.143)	-0.016 (0.106)	
- Range	0.704 - 1.665	0.661 - 1.258	-0.407 - 0.084	
Sagittal stratum R				0.823
- Mean (SD)	0.769 (0.070)	0.756 (0.057)	-0.014 (0.042)	
- Range	0.643 - 0.920	0.664 - 0.850	-0.139 - 0.041	
Sagittal stratum L				0.823
- Mean (SD)	0.870 (0.136)	0.850 (0.104)	-0.020 (0.064)	
- Range	0.692 - 1.230	0.686 - 1.037	-0.219 - 0.059	
External capsule R				0.861
- Mean (SD)	0.718 (0.064)	0.721 (0.061)	0.003 (0.026)	
- Range	0.647 - 0.861	0.637 - 0.838	-0.027 - 0.076	
External capsule L				0.823
- Mean (SD)	0.697 (0.049)	0.691 (0.060)	-0.006 (0.029)	
- Range	0.631 - 0.771	0.613 - 0.795	-0.052 - 0.063	
Cingulum cingulate gyrus R				0.944
- Mean (SD)	0.726 (0.070)	0.728 (0.067)	0.002 (0.033)	
- Range	0.621 - 0.880	0.640 - 0.915	-0.072 - 0.064	
Cingulum cingulate gyrus L				0.823
- Mean (SD)	0.745 (0.059)	0.754 (0.070)	0.009 (0.027)	
- Range	0.666 - 0.865	0.655 - 0.891	-0.032 - 0.068	
Cingulum hippocampus R				0.823
- Mean (SD)	0.919 (0.097)	0.897 (0.097)	-0.022 (0.104)	
- Range	0.765 - 1.050	0.715 - 1.079	-0.178 - 0.261	
Cingulum hippocampus L				0.823
- Mean (SD)	0.973 (0.093)	0.922 (0.096)	-0.051 (0.113)	
- Range	0.810 - 1.159	0.780 - 1.097	-0.266 - 0.094	
Fornix R				0.861
- Mean (SD)	0.876 (0.097)	0.880 (0.113)	0.004 (0.038)	
- Range	0.650 - 1.042	0.642 - 1.087	-0.040 - 0.077	
Fornix L				0.823
- Mean (SD)	0.826 (0.087)	0.833 (0.101)	0.007 (0.046)	
- Range	0.623 - 0.970	0.648 - 1.065	-0.088 - 0.095	
Superior longitudinal fasciculus R				0.823

	Post (N=18)	Pre (N=18)	Difference (N=18)	p value
- Mean (SD)	0.642 (0.067)	0.635 (0.054)	-0.008 (0.031)	
- Range	0.567 - 0.809	0.571 - 0.766	-0.082 - 0.034	
Superior longitudinal fasciculus L				0.823
- Mean (SD)	0.633 (0.052)	0.630 (0.052)	-0.003 (0.022)	
- Range	0.532 - 0.711	0.575 - 0.730	-0.046 - 0.043	
Superior fronto occipital fasciculus R				0.823
- Mean (SD)	0.808 (0.176)	0.799 (0.199)	-0.009 (0.065)	
- Range	0.578 - 1.254	0.561 - 1.371	-0.140 - 0.117	
Superior fronto occipital fasciculus L				0.955
- Mean (SD)	0.919 (0.192)	0.921 (0.232)	0.002 (0.101)	
- Range	0.651 - 1.243	0.644 - 1.533	-0.126 - 0.290	
Uncinate fasciculus R				0.916
- Mean (SD)	0.693 (0.055)	0.700 (0.091)	0.007 (0.090)	
- Range	0.557 - 0.753	0.598 - 0.957	-0.078 - 0.234	
Uncinate fasciculus L				0.823
- Mean (SD)	0.755 (0.183)	0.703 (0.113)	-0.053 (0.144)	
- Range	0.595 - 1.225	0.590 - 1.063	-0.451 - 0.214	
Tapetum R				0.823
- Mean (SD)	1.748 (0.272)	1.779 (0.295)	0.030 (0.166)	
- Range	1.237 - 2.245	1.231 - 2.218	-0.230 - 0.311	
Tapetum L				0.823
- Mean (SD)	1.996 (0.247)	2.062 (0.329)	0.066 (0.196)	
- Range	1.570 - 2.375	1.405 - 2.503	-0.239 - 0.490	

Fractional anisotropy (FA) (DTI) - Offspring of obese mothers (OOM) subjects pre vs post (paired tests) - (JHU white-matter tractography atlas) - Uncorrected p-values

	Post (N=13)	Pre (N=13)	Difference (N=13)	p value
Middle cerebellar peduncle				0.539
- Mean (SD)	0.404 (0.047)	0.390 (0.063)	-0.014 (0.081)	
- Range	0.293 - 0.454	0.284 - 0.451	-0.152 - 0.153	
Pontine crossing tract				0.204
- Mean (SD)	0.408 (0.025)	0.396 (0.035)	-0.012 (0.032)	
- Range	0.359 - 0.449	0.337 - 0.467	-0.078 - 0.026	
Genu of corpus callosum				0.191
- Mean (SD)	0.436 (0.017)	0.432 (0.020)	-0.004 (0.010)	
- Range	0.408 - 0.459	0.391 - 0.461	-0.021 - 0.011	
Body of corpus callosum				0.239
- Mean (SD)	0.488 (0.025)	0.483 (0.027)	-0.005 (0.013)	
- Range	0.450 - 0.529	0.441 - 0.532	-0.041 - 0.017	
Splenium of corpus callosum				0.050
- Mean (SD)	0.595 (0.016)	0.589 (0.020)	-0.005 (0.009)	
- Range	0.570 - 0.622	0.555 - 0.618	-0.019 - 0.012	
Fornix				0.518
- Mean (SD)	0.246 (0.053)	0.249 (0.047)	0.003 (0.018)	
- Range	0.179 - 0.340	0.184 - 0.332	-0.036 - 0.035	
Corticospinal tract R				0.491
- Mean (SD)	0.486 (0.035)	0.477 (0.048)	-0.008 (0.043)	
- Range	0.437 - 0.553	0.403 - 0.562	-0.072 - 0.088	
Corticospinal tract L				0.481
- Mean (SD)	0.482 (0.035)	0.472 (0.038)	-0.010 (0.048)	
- Range	0.442 - 0.553	0.418 - 0.530	-0.085 - 0.053	
Medial lemniscus R				0.594
- Mean (SD)	0.449 (0.046)	0.441 (0.041)	-0.009 (0.058)	
- Range	0.400 - 0.556	0.375 - 0.490	-0.136 - 0.071	
Medial lemniscus L				0.879
- Mean (SD)	0.456 (0.041)	0.459 (0.060)	0.003 (0.066)	
- Range	0.397 - 0.537	0.353 - 0.556	-0.099 - 0.105	
Inferior cerebellar peduncle R				0.552
- Mean (SD)	0.340 (0.035)	0.350 (0.048)	0.010 (0.058)	
- Range	0.298 - 0.409	0.270 - 0.437	-0.139 - 0.091	
Inferior cerebellar peduncle L				0.068
- Mean (SD)	0.359 (0.036)	0.329 (0.047)	-0.030 (0.053)	
- Range	0.309 - 0.426	0.230 - 0.399	-0.156 - 0.028	
Superior cerebellar peduncle R				0.431
- Mean (SD)	0.467 (0.054)	0.447 (0.068)	-0.020 (0.090)	
- Range	0.350 - 0.512	0.298 - 0.517	-0.181 - 0.134	
Superior cerebellar peduncle L				0.491
- Mean (SD)	0.439 (0.054)	0.424 (0.055)	-0.015 (0.077)	
- Range	0.311 - 0.500	0.308 - 0.495	-0.154 - 0.139	
Cerebral peduncle R				0.912
- Mean (SD)	0.562 (0.021)	0.562 (0.018)	-0.000 (0.016)	
- Range	0.534 - 0.600	0.534 - 0.608	-0.043 - 0.022	
Cerebral peduncle L				0.953
- Mean (SD)	0.572 (0.019)	0.573 (0.022)	0.000 (0.019)	
- Range	0.536 - 0.608	0.535 - 0.608	-0.037 - 0.030	
Anterior limb of internal capsule R				0.292
- Mean (SD)	0.437 (0.024)	0.432 (0.026)	-0.005 (0.015)	
- Range	0.387 - 0.476	0.380 - 0.466	-0.042 - 0.016	
Anterior limb of internal capsule L				0.333
- Mean (SD)	0.446 (0.023)	0.440 (0.029)	-0.006 (0.021)	
- Range	0.403 - 0.486	0.399 - 0.490	-0.064 - 0.017	
Posterior limb of internal capsule R				0.375
- Mean (SD)	0.530 (0.021)	0.527 (0.019)	-0.003 (0.011)	
- Range	0.500 - 0.566	0.502 - 0.559	-0.018 - 0.018	
Posterior limb of internal capsule L				0.958
- Mean (SD)	0.536 (0.021)	0.536 (0.016)	-0.000 (0.016)	

	Post (N=13)	Pre (N=13)	Difference (N=13)	p value
- Range	0.516 - 0.585	0.508 - 0.573	-0.033 - 0.023	
Retrolenticular part of internal capsule R				0.141
- Mean (SD)	0.443 (0.025)	0.436 (0.020)	-0.007 (0.017)	
- Range	0.402 - 0.477	0.402 - 0.481	-0.029 - 0.025	
Retrolenticular part of internal capsule L				0.323
- Mean (SD)	0.449 (0.026)	0.453 (0.029)	0.005 (0.016)	
- Range	0.408 - 0.489	0.404 - 0.503	-0.023 - 0.034	
Anterior corona radiata R				0.805
- Mean (SD)	0.336 (0.022)	0.336 (0.023)	-0.001 (0.012)	
- Range	0.296 - 0.378	0.304 - 0.375	-0.021 - 0.018	
Anterior corona radiata L				0.368
- Mean (SD)	0.341 (0.023)	0.336 (0.025)	-0.004 (0.016)	
- Range	0.303 - 0.389	0.284 - 0.378	-0.034 - 0.026	
Superior corona radiata R				0.820
- Mean (SD)	0.395 (0.028)	0.396 (0.028)	0.001 (0.010)	
- Range	0.348 - 0.447	0.351 - 0.444	-0.016 - 0.021	
Superior corona radiata L				0.504
- Mean (SD)	0.401 (0.029)	0.403 (0.032)	0.002 (0.009)	
- Range	0.363 - 0.455	0.362 - 0.461	-0.014 - 0.019	
Posterior corona radiata R				0.326
- Mean (SD)	0.414 (0.026)	0.411 (0.024)	-0.003 (0.011)	
- Range	0.371 - 0.458	0.370 - 0.455	-0.016 - 0.015	
Posterior corona radiata L				0.004
- Mean (SD)	0.393 (0.027)	0.404 (0.028)	0.011 (0.011)	
- Range	0.345 - 0.434	0.344 - 0.439	-0.011 - 0.029	
Posterior thalamic radiation R				0.667
- Mean (SD)	0.460 (0.016)	0.462 (0.025)	0.002 (0.015)	
- Range	0.433 - 0.486	0.412 - 0.494	-0.021 - 0.027	
Posterior thalamic radiation L				0.871
- Mean (SD)	0.453 (0.019)	0.454 (0.024)	0.001 (0.013)	
- Range	0.416 - 0.475	0.402 - 0.492	-0.017 - 0.026	
Sagittal stratum R				0.392
- Mean (SD)	0.419 (0.025)	0.423 (0.021)	0.005 (0.018)	
- Range	0.379 - 0.467	0.391 - 0.457	-0.025 - 0.035	
Sagittal stratum L				0.928
- Mean (SD)	0.390 (0.016)	0.389 (0.022)	-0.001 (0.021)	
- Range	0.373 - 0.433	0.349 - 0.445	-0.039 - 0.050	
External capsule R				0.573
- Mean (SD)	0.329 (0.015)	0.326 (0.014)	-0.003 (0.017)	
- Range	0.304 - 0.348	0.305 - 0.346	-0.035 - 0.017	
External capsule L				0.197
- Mean (SD)	0.334 (0.017)	0.338 (0.014)	0.004 (0.010)	
- Range	0.303 - 0.369	0.317 - 0.363	-0.016 - 0.018	
Cingulum cingulate gyrus R				0.514
- Mean (SD)	0.353 (0.023)	0.350 (0.016)	-0.003 (0.014)	
- Range	0.322 - 0.402	0.325 - 0.385	-0.022 - 0.023	
Cingulum cingulate gyrus L				0.438
- Mean (SD)	0.366 (0.029)	0.364 (0.027)	-0.002 (0.009)	
- Range	0.315 - 0.418	0.313 - 0.416	-0.019 - 0.016	
Cingulum hippocampus R				0.833
- Mean (SD)	0.277 (0.017)	0.279 (0.030)	0.002 (0.032)	
- Range	0.252 - 0.305	0.249 - 0.334	-0.055 - 0.048	
Cingulum hippocampus L				0.253
- Mean (SD)	0.278 (0.024)	0.268 (0.021)	-0.010 (0.029)	
- Range	0.233 - 0.312	0.234 - 0.309	-0.053 - 0.038	
Fornix R				0.275
- Mean (SD)	0.355 (0.018)	0.363 (0.032)	0.008 (0.024)	
- Range	0.314 - 0.383	0.306 - 0.421	-0.034 - 0.065	
Fornix L				0.838
- Mean (SD)	0.383 (0.028)	0.382 (0.034)	-0.001 (0.017)	
- Range	0.332 - 0.423	0.326 - 0.433	-0.025 - 0.024	
Superior longitudinal fasciculus R				0.931

	Post (N=13)	Pre (N=13)	Difference (N=13)	p value
- Mean (SD)	0.382 (0.020)	0.382 (0.015)	-0.000 (0.016)	
- Range	0.355 - 0.424	0.346 - 0.403	-0.031 - 0.032	
Superior longitudinal fasciculus L				0.853
- Mean (SD)	0.389 (0.024)	0.390 (0.021)	0.001 (0.015)	
- Range	0.355 - 0.438	0.350 - 0.432	-0.028 - 0.018	
Superior fronto occipital fasciculus R				0.848
- Mean (SD)	0.358 (0.035)	0.359 (0.030)	0.002 (0.031)	
- Range	0.283 - 0.405	0.293 - 0.398	-0.064 - 0.062	
Superior fronto occipital fasciculus L				0.013
- Mean (SD)	0.353 (0.041)	0.336 (0.029)	-0.017 (0.021)	
- Range	0.277 - 0.410	0.267 - 0.371	-0.060 - 0.014	
Uncinate fasciculus R				0.248
- Mean (SD)	0.373 (0.051)	0.387 (0.030)	0.014 (0.042)	
- Range	0.247 - 0.424	0.328 - 0.427	-0.051 - 0.128	
Uncinate fasciculus L				0.237
- Mean (SD)	0.382 (0.031)	0.395 (0.044)	0.013 (0.037)	
- Range	0.334 - 0.432	0.328 - 0.452	-0.034 - 0.081	
Tapetum R				0.238
- Mean (SD)	0.331 (0.026)	0.338 (0.031)	0.008 (0.022)	
- Range	0.283 - 0.369	0.283 - 0.387	-0.029 - 0.047	
Tapetum L				0.114
- Mean (SD)	0.294 (0.027)	0.303 (0.030)	0.009 (0.020)	
- Range	0.254 - 0.341	0.257 - 0.372	-0.016 - 0.049	

Fractional anisotropy (FA) (DTI) - Offspring of obese mothers (OOM) subjects pre vs post (paired tests) - (JHU white-matter tractography atlas) - FDR corrected p-values

	Post (N=13)	Pre (N=13)	Difference (N=13)	p value
Middle cerebellar peduncle				0.828
- Mean (SD)	0.404 (0.047)	0.390 (0.063)	-0.014 (0.081)	
- Range	0.293 - 0.454	0.284 - 0.451	-0.152 - 0.153	
Pontine crossing tract				0.828
- Mean (SD)	0.408 (0.025)	0.396 (0.035)	-0.012 (0.032)	
- Range	0.359 - 0.449	0.337 - 0.467	-0.078 - 0.026	
Genu of corpus callosum				0.828
- Mean (SD)	0.436 (0.017)	0.432 (0.020)	-0.004 (0.010)	
- Range	0.408 - 0.459	0.391 - 0.461	-0.021 - 0.011	
Body of corpus callosum				0.828
- Mean (SD)	0.488 (0.025)	0.483 (0.027)	-0.005 (0.013)	
- Range	0.450 - 0.529	0.441 - 0.532	-0.041 - 0.017	
Splenium of corpus callosum				0.793
- Mean (SD)	0.595 (0.016)	0.589 (0.020)	-0.005 (0.009)	
- Range	0.570 - 0.622	0.555 - 0.618	-0.019 - 0.012	
Fornix				0.828
- Mean (SD)	0.246 (0.053)	0.249 (0.047)	0.003 (0.018)	
- Range	0.179 - 0.340	0.184 - 0.332	-0.036 - 0.035	
Corticospinal tract R				0.828
- Mean (SD)	0.486 (0.035)	0.477 (0.048)	-0.008 (0.043)	
- Range	0.437 - 0.553	0.403 - 0.562	-0.072 - 0.088	
Corticospinal tract L				0.828
- Mean (SD)	0.482 (0.035)	0.472 (0.038)	-0.010 (0.048)	
- Range	0.442 - 0.553	0.418 - 0.530	-0.085 - 0.053	
Medial lemniscus R				0.839
- Mean (SD)	0.449 (0.046)	0.441 (0.041)	-0.009 (0.058)	
- Range	0.400 - 0.556	0.375 - 0.490	-0.136 - 0.071	
Medial lemniscus L				0.958
- Mean (SD)	0.456 (0.041)	0.459 (0.060)	0.003 (0.066)	
- Range	0.397 - 0.537	0.353 - 0.556	-0.099 - 0.105	
Inferior cerebellar peduncle R				0.828
- Mean (SD)	0.340 (0.035)	0.350 (0.048)	0.010 (0.058)	
- Range	0.298 - 0.409	0.270 - 0.437	-0.139 - 0.091	
Inferior cerebellar peduncle L				0.815
- Mean (SD)	0.359 (0.036)	0.329 (0.047)	-0.030 (0.053)	
- Range	0.309 - 0.426	0.230 - 0.399	-0.156 - 0.028	
Superior cerebellar peduncle R				0.828
- Mean (SD)	0.467 (0.054)	0.447 (0.068)	-0.020 (0.090)	
- Range	0.350 - 0.512	0.298 - 0.517	-0.181 - 0.134	
Superior cerebellar peduncle L				0.828
- Mean (SD)	0.439 (0.054)	0.424 (0.055)	-0.015 (0.077)	
- Range	0.311 - 0.500	0.308 - 0.495	-0.154 - 0.139	
Cerebral peduncle R				0.958
- Mean (SD)	0.562 (0.021)	0.562 (0.018)	-0.000 (0.016)	
- Range	0.534 - 0.600	0.534 - 0.608	-0.043 - 0.022	
Cerebral peduncle L				0.958
- Mean (SD)	0.572 (0.019)	0.573 (0.022)	0.000 (0.019)	
- Range	0.536 - 0.608	0.535 - 0.608	-0.037 - 0.030	
Anterior limb of internal capsule R				0.828
- Mean (SD)	0.437 (0.024)	0.432 (0.026)	-0.005 (0.015)	
- Range	0.387 - 0.476	0.380 - 0.466	-0.042 - 0.016	
Anterior limb of internal capsule L				0.828
- Mean (SD)	0.446 (0.023)	0.440 (0.029)	-0.006 (0.021)	
- Range	0.403 - 0.486	0.399 - 0.490	-0.064 - 0.017	
Posterior limb of internal capsule R				0.828
- Mean (SD)	0.530 (0.021)	0.527 (0.019)	-0.003 (0.011)	
- Range	0.500 - 0.566	0.502 - 0.559	-0.018 - 0.018	
Posterior limb of internal capsule L				0.958
- Mean (SD)	0.536 (0.021)	0.536 (0.016)	-0.000 (0.016)	

	Post (N=13)	Pre (N=13)	Difference (N=13)	p value
- Range	0.516 - 0.585	0.508 - 0.573	-0.033 - 0.023	
Retrolenticular part of internal capsule R				0.828
- Mean (SD)	0.443 (0.025)	0.436 (0.020)	-0.007 (0.017)	
- Range	0.402 - 0.477	0.402 - 0.481	-0.029 - 0.025	
Retrolenticular part of internal capsule L				0.828
- Mean (SD)	0.449 (0.026)	0.453 (0.029)	0.005 (0.016)	
- Range	0.408 - 0.489	0.404 - 0.503	-0.023 - 0.034	
Anterior corona radiata R				0.958
- Mean (SD)	0.336 (0.022)	0.336 (0.023)	-0.001 (0.012)	
- Range	0.296 - 0.378	0.304 - 0.375	-0.021 - 0.018	
Anterior corona radiata L				0.828
- Mean (SD)	0.341 (0.023)	0.336 (0.025)	-0.004 (0.016)	
- Range	0.303 - 0.389	0.284 - 0.378	-0.034 - 0.026	
Superior corona radiata R				0.958
- Mean (SD)	0.395 (0.028)	0.396 (0.028)	0.001 (0.010)	
- Range	0.348 - 0.447	0.351 - 0.444	-0.016 - 0.021	
Superior corona radiata L				0.828
- Mean (SD)	0.401 (0.029)	0.403 (0.032)	0.002 (0.009)	
- Range	0.363 - 0.455	0.362 - 0.461	-0.014 - 0.019	
Posterior corona radiata R				0.828
- Mean (SD)	0.414 (0.026)	0.411 (0.024)	-0.003 (0.011)	
- Range	0.371 - 0.458	0.370 - 0.455	-0.016 - 0.015	
Posterior corona radiata L				0.208
- Mean (SD)	0.393 (0.027)	0.404 (0.028)	0.011 (0.011)	
- Range	0.345 - 0.434	0.344 - 0.439	-0.011 - 0.029	
Posterior thalamic radiation R				0.915
- Mean (SD)	0.460 (0.016)	0.462 (0.025)	0.002 (0.015)	
- Range	0.433 - 0.486	0.412 - 0.494	-0.021 - 0.027	
Posterior thalamic radiation L				0.958
- Mean (SD)	0.453 (0.019)	0.454 (0.024)	0.001 (0.013)	
- Range	0.416 - 0.475	0.402 - 0.492	-0.017 - 0.026	
Sagittal stratum R				0.828
- Mean (SD)	0.419 (0.025)	0.423 (0.021)	0.005 (0.018)	
- Range	0.379 - 0.467	0.391 - 0.457	-0.025 - 0.035	
Sagittal stratum L				0.958
- Mean (SD)	0.390 (0.016)	0.389 (0.022)	-0.001 (0.021)	
- Range	0.373 - 0.433	0.349 - 0.445	-0.039 - 0.050	
External capsule R				0.834
- Mean (SD)	0.329 (0.015)	0.326 (0.014)	-0.003 (0.017)	
- Range	0.304 - 0.348	0.305 - 0.346	-0.035 - 0.017	
External capsule L				0.828
- Mean (SD)	0.334 (0.017)	0.338 (0.014)	0.004 (0.010)	
- Range	0.303 - 0.369	0.317 - 0.363	-0.016 - 0.018	
Cingulum cingulate gyrus R				0.828
- Mean (SD)	0.353 (0.023)	0.350 (0.016)	-0.003 (0.014)	
- Range	0.322 - 0.402	0.325 - 0.385	-0.022 - 0.023	
Cingulum cingulate gyrus L				0.828
- Mean (SD)	0.366 (0.029)	0.364 (0.027)	-0.002 (0.009)	
- Range	0.315 - 0.418	0.313 - 0.416	-0.019 - 0.016	
Cingulum hippocampus R				0.958
- Mean (SD)	0.277 (0.017)	0.279 (0.030)	0.002 (0.032)	
- Range	0.252 - 0.305	0.249 - 0.334	-0.055 - 0.048	
Cingulum hippocampus L				0.828
- Mean (SD)	0.278 (0.024)	0.268 (0.021)	-0.010 (0.029)	
- Range	0.233 - 0.312	0.234 - 0.309	-0.053 - 0.038	
Fornix R				0.828
- Mean (SD)	0.355 (0.018)	0.363 (0.032)	0.008 (0.024)	
- Range	0.314 - 0.383	0.306 - 0.421	-0.034 - 0.065	
Fornix L				0.958
- Mean (SD)	0.383 (0.028)	0.382 (0.034)	-0.001 (0.017)	
- Range	0.332 - 0.423	0.326 - 0.433	-0.025 - 0.024	
Superior longitudinal fasciculus R				0.958

	Post (N=13)	Pre (N=13)	Difference (N=13)	p value
- Mean (SD)	0.382 (0.020)	0.382 (0.015)	-0.000 (0.016)	
- Range	0.355 - 0.424	0.346 - 0.403	-0.031 - 0.032	
Superior longitudinal fasciculus L				0.958
- Mean (SD)	0.389 (0.024)	0.390 (0.021)	0.001 (0.015)	
- Range	0.355 - 0.438	0.350 - 0.432	-0.028 - 0.018	
Superior fronto occipital fasciculus R				0.958
- Mean (SD)	0.358 (0.035)	0.359 (0.030)	0.002 (0.031)	
- Range	0.283 - 0.405	0.293 - 0.398	-0.064 - 0.062	
Superior fronto occipital fasciculus L				0.302
- Mean (SD)	0.353 (0.041)	0.336 (0.029)	-0.017 (0.021)	
- Range	0.277 - 0.410	0.267 - 0.371	-0.060 - 0.014	
Uncinate fasciculus R				0.828
- Mean (SD)	0.373 (0.051)	0.387 (0.030)	0.014 (0.042)	
- Range	0.247 - 0.424	0.328 - 0.427	-0.051 - 0.128	
Uncinate fasciculus L				0.828
- Mean (SD)	0.382 (0.031)	0.395 (0.044)	0.013 (0.037)	
- Range	0.334 - 0.432	0.328 - 0.452	-0.034 - 0.081	
Tapetum R				0.828
- Mean (SD)	0.331 (0.026)	0.338 (0.031)	0.008 (0.022)	
- Range	0.283 - 0.369	0.283 - 0.387	-0.029 - 0.047	
Tapetum L				0.828
- Mean (SD)	0.294 (0.027)	0.303 (0.030)	0.009 (0.020)	
- Range	0.254 - 0.341	0.257 - 0.372	-0.016 - 0.049	

Mean diffusivity (MD, (10^-3)*(mm^2)/s) (DTI) - Offspring of obese mothers (OOM) subjects pre vs post (paired tests) - (JHU white-matter tractography atlas) - Uncorrected p-values

	Post (N=13)	Pre (N=13)	Difference (N=13)	p value
Middle cerebellar peduncle				0.573
- Mean (SD)	1.083 (0.144)	1.115 (0.141)	0.032 (0.198)	
- Range	0.874 - 1.380	0.986 - 1.425	-0.271 - 0.414	
Pontine crossing tract				0.359
- Mean (SD)	0.787 (0.070)	0.823 (0.088)	0.036 (0.134)	
- Range	0.681 - 0.967	0.724 - 1.066	-0.200 - 0.342	
Genu of corpus callosum				0.368
- Mean (SD)	1.346 (0.086)	1.360 (0.123)	0.014 (0.052)	
- Range	1.270 - 1.583	1.231 - 1.664	-0.089 - 0.112	
Body of corpus callosum				0.231
- Mean (SD)	1.116 (0.058)	1.138 (0.103)	0.022 (0.064)	
- Range	1.030 - 1.226	1.007 - 1.370	-0.050 - 0.153	
Splenium of corpus callosum				0.063
- Mean (SD)	1.009 (0.064)	1.037 (0.103)	0.028 (0.050)	
- Range	0.904 - 1.157	0.886 - 1.287	-0.018 - 0.130	
Fornix				0.776
- Mean (SD)	2.343 (0.357)	2.337 (0.391)	-0.006 (0.073)	
- Range	1.880 - 2.864	1.853 - 2.857	-0.125 - 0.115	
Corticospinal tract R				0.381
- Mean (SD)	0.835 (0.076)	0.857 (0.057)	0.022 (0.086)	
- Range	0.708 - 0.978	0.750 - 0.946	-0.107 - 0.187	
Corticospinal tract L				0.321
- Mean (SD)	0.799 (0.053)	0.815 (0.044)	0.016 (0.055)	
- Range	0.696 - 0.891	0.746 - 0.885	-0.052 - 0.155	
Medial lemniscus R				0.082
- Mean (SD)	0.846 (0.054)	0.904 (0.109)	0.058 (0.111)	
- Range	0.777 - 0.932	0.812 - 1.210	-0.104 - 0.357	
Medial lemniscus L				0.006
- Mean (SD)	0.815 (0.062)	0.881 (0.066)	0.066 (0.071)	
- Range	0.706 - 0.942	0.796 - 1.034	-0.056 - 0.201	
Inferior cerebellar peduncle R				0.974
- Mean (SD)	1.163 (0.223)	1.160 (0.198)	-0.003 (0.325)	
- Range	0.965 - 1.790	0.927 - 1.639	-0.614 - 0.623	
Inferior cerebellar peduncle L				0.773
- Mean (SD)	1.099 (0.258)	1.132 (0.257)	0.033 (0.402)	
- Range	0.852 - 1.784	0.917 - 1.748	-0.760 - 0.784	
Superior cerebellar peduncle R				0.879
- Mean (SD)	1.230 (0.184)	1.240 (0.167)	0.010 (0.235)	
- Range	0.971 - 1.547	0.947 - 1.532	-0.557 - 0.402	
Superior cerebellar peduncle L				0.632
- Mean (SD)	1.330 (0.262)	1.295 (0.197)	-0.035 (0.260)	
- Range	0.955 - 1.938	0.913 - 1.623	-0.575 - 0.327	
Cerebral peduncle R				0.537
- Mean (SD)	0.882 (0.047)	0.888 (0.049)	0.006 (0.035)	
- Range	0.808 - 0.960	0.806 - 0.971	-0.017 - 0.114	
Cerebral peduncle L				0.285
- Mean (SD)	0.845 (0.035)	0.836 (0.033)	-0.009 (0.028)	
- Range	0.784 - 0.890	0.791 - 0.883	-0.065 - 0.045	
Anterior limb of internal capsule R				0.804
- Mean (SD)	0.848 (0.050)	0.846 (0.058)	-0.002 (0.026)	
- Range	0.793 - 0.934	0.792 - 0.962	-0.057 - 0.037	
Anterior limb of internal capsule L				0.473
- Mean (SD)	0.870 (0.064)	0.877 (0.070)	0.007 (0.036)	
- Range	0.781 - 0.985	0.789 - 1.025	-0.041 - 0.080	
Posterior limb of internal capsule R				0.926
- Mean (SD)	0.760 (0.019)	0.760 (0.021)	0.000 (0.015)	
- Range	0.731 - 0.797	0.722 - 0.788	-0.030 - 0.024	
Posterior limb of internal capsule L				0.177
- Mean (SD)	0.756 (0.018)	0.767 (0.027)	0.010 (0.026)	

	Post (N=13)	Pre (N=13)	Difference (N=13)	p value
- Range	0.726 - 0.788	0.726 - 0.819	-0.008 - 0.087	
Retrolenticular part of internal capsule R				0.220
- Mean (SD)	0.909 (0.056)	0.924 (0.077)	0.015 (0.041)	
- Range	0.843 - 1.031	0.845 - 1.062	-0.021 - 0.132	
Retrolenticular part of internal capsule L				0.074
- Mean (SD)	0.906 (0.051)	0.933 (0.078)	0.027 (0.050)	
- Range	0.833 - 0.998	0.825 - 1.074	-0.057 - 0.118	
Anterior corona radiata R				0.414
- Mean (SD)	0.913 (0.065)	0.929 (0.122)	0.016 (0.070)	
- Range	0.843 - 1.054	0.823 - 1.248	-0.029 - 0.236	
Anterior corona radiata L				0.199
- Mean (SD)	0.911 (0.095)	0.922 (0.106)	0.010 (0.027)	
- Range	0.821 - 1.179	0.829 - 1.215	-0.023 - 0.078	
Superior corona radiata R				0.116
- Mean (SD)	0.794 (0.057)	0.811 (0.077)	0.017 (0.036)	
- Range	0.728 - 0.893	0.735 - 0.946	-0.011 - 0.121	
Superior corona radiata L				0.172
- Mean (SD)	0.810 (0.059)	0.832 (0.103)	0.022 (0.054)	
- Range	0.720 - 0.930	0.733 - 1.099	-0.015 - 0.169	
Posterior corona radiata R				0.310
- Mean (SD)	0.916 (0.103)	0.934 (0.132)	0.019 (0.064)	
- Range	0.801 - 1.123	0.780 - 1.146	-0.048 - 0.218	
Posterior corona radiata L				0.166
- Mean (SD)	0.930 (0.100)	0.961 (0.153)	0.031 (0.075)	
- Range	0.810 - 1.126	0.800 - 1.328	-0.051 - 0.202	
Posterior thalamic radiation R				0.808
- Mean (SD)	1.010 (0.193)	1.013 (0.180)	0.003 (0.040)	
- Range	0.834 - 1.518	0.828 - 1.510	-0.067 - 0.094	
Posterior thalamic radiation L				0.165
- Mean (SD)	1.075 (0.130)	1.121 (0.188)	0.046 (0.113)	
- Range	0.867 - 1.274	0.935 - 1.589	-0.093 - 0.373	
Sagittal stratum R				0.374
- Mean (SD)	0.990 (0.097)	1.003 (0.126)	0.013 (0.053)	
- Range	0.875 - 1.198	0.889 - 1.335	-0.070 - 0.137	
Sagittal stratum L				0.530
- Mean (SD)	1.051 (0.108)	1.062 (0.104)	0.011 (0.062)	
- Range	0.898 - 1.209	0.913 - 1.279	-0.093 - 0.163	
External capsule R				0.296
- Mean (SD)	0.847 (0.047)	0.856 (0.057)	0.009 (0.031)	
- Range	0.772 - 0.947	0.791 - 0.955	-0.025 - 0.075	
External capsule L				0.028
- Mean (SD)	0.848 (0.048)	0.831 (0.052)	-0.017 (0.024)	
- Range	0.792 - 0.939	0.771 - 0.945	-0.071 - 0.013	
Cingulum cingulate gyrus R				0.320
- Mean (SD)	0.933 (0.068)	0.918 (0.053)	-0.015 (0.051)	
- Range	0.810 - 1.067	0.819 - 0.979	-0.109 - 0.073	
Cingulum cingulate gyrus L				0.203
- Mean (SD)	0.941 (0.067)	0.962 (0.080)	0.021 (0.057)	
- Range	0.824 - 1.035	0.839 - 1.108	-0.036 - 0.146	
Cingulum hippocampus R				0.977
- Mean (SD)	1.107 (0.164)	1.109 (0.138)	0.001 (0.170)	
- Range	0.855 - 1.506	0.934 - 1.368	-0.480 - 0.204	
Cingulum hippocampus L				0.023
- Mean (SD)	1.121 (0.136)	1.187 (0.143)	0.066 (0.091)	
- Range	0.870 - 1.336	0.930 - 1.407	-0.049 - 0.213	
Fornix R				0.098
- Mean (SD)	1.044 (0.116)	1.082 (0.146)	0.038 (0.077)	
- Range	0.859 - 1.298	0.884 - 1.370	-0.029 - 0.273	
Fornix L				0.399
- Mean (SD)	0.986 (0.118)	1.003 (0.129)	0.017 (0.068)	
- Range	0.817 - 1.209	0.868 - 1.313	-0.126 - 0.121	
Superior longitudinal fasciculus R				0.772

	Post (N=13)	Pre (N=13)	Difference (N=13)	p value
- Mean (SD)	0.800 (0.040)	0.801 (0.045)	0.002 (0.021)	
- Range	0.751 - 0.881	0.734 - 0.883	-0.046 - 0.043	
Superior longitudinal fasciculus L				0.466
- Mean (SD)	0.779 (0.035)	0.784 (0.040)	0.005 (0.023)	
- Range	0.741 - 0.847	0.738 - 0.865	-0.028 - 0.050	
Superior fronto occipital fasciculus R				0.132
- Mean (SD)	0.884 (0.157)	0.932 (0.252)	0.048 (0.107)	
- Range	0.745 - 1.300	0.733 - 1.571	-0.049 - 0.271	
Superior fronto occipital fasciculus L				0.228
- Mean (SD)	0.995 (0.217)	1.039 (0.306)	0.043 (0.123)	
- Range	0.770 - 1.580	0.795 - 1.863	-0.106 - 0.327	
Uncinate fasciculus R				0.251
- Mean (SD)	0.975 (0.242)	0.919 (0.136)	-0.055 (0.166)	
- Range	0.803 - 1.519	0.790 - 1.267	-0.475 - 0.132	
Uncinate fasciculus L				0.102
- Mean (SD)	0.835 (0.041)	0.876 (0.089)	0.041 (0.084)	
- Range	0.778 - 0.926	0.784 - 1.136	-0.022 - 0.301	
Tapetum R				0.169
- Mean (SD)	1.858 (0.425)	1.941 (0.457)	0.083 (0.205)	
- Range	1.190 - 2.620	1.141 - 2.588	-0.201 - 0.437	
Tapetum L				0.559
- Mean (SD)	2.188 (0.516)	2.223 (0.477)	0.036 (0.213)	
- Range	1.418 - 3.016	1.407 - 2.919	-0.255 - 0.370	

Mean diffusivity (MD, (10^-3)*(mm^2)/s) (DTI) - Offspring of obese mothers (OOM) subjects pre vs post (paired tests) - (JHU white-matter tractography atlas) - FDR corrected p-values

	Post (N=13)	Pre (N=13)	Difference (N=13)	p value
Middle cerebellar peduncle				0.724
- Mean (SD)	1.083 (0.144)	1.115 (0.141)	0.032 (0.198)	
- Range	0.874 - 1.380	0.986 - 1.425	-0.271 - 0.414	
Pontine crossing tract				0.609
- Mean (SD)	0.787 (0.070)	0.823 (0.088)	0.036 (0.134)	
- Range	0.681 - 0.967	0.724 - 1.066	-0.200 - 0.342	
Genu of corpus callosum				0.609
- Mean (SD)	1.346 (0.086)	1.360 (0.123)	0.014 (0.052)	
- Range	1.270 - 1.583	1.231 - 1.664	-0.089 - 0.112	
Body of corpus callosum				0.553
- Mean (SD)	1.116 (0.058)	1.138 (0.103)	0.022 (0.064)	
- Range	1.030 - 1.226	1.007 - 1.370	-0.050 - 0.153	
Splenium of corpus callosum				0.553
- Mean (SD)	1.009 (0.064)	1.037 (0.103)	0.028 (0.050)	
- Range	0.904 - 1.157	0.886 - 1.287	-0.018 - 0.130	
Fornix				0.882
- Mean (SD)	2.343 (0.357)	2.337 (0.391)	-0.006 (0.073)	
- Range	1.880 - 2.864	1.853 - 2.857	-0.125 - 0.115	
Corticospinal tract R				0.609
- Mean (SD)	0.835 (0.076)	0.857 (0.057)	0.022 (0.086)	
- Range	0.708 - 0.978	0.750 - 0.946	-0.107 - 0.187	
Corticospinal tract L				0.592
- Mean (SD)	0.799 (0.053)	0.815 (0.044)	0.016 (0.055)	
- Range	0.696 - 0.891	0.746 - 0.885	-0.052 - 0.155	
Medial lemniscus R				0.553
- Mean (SD)	0.846 (0.054)	0.904 (0.109)	0.058 (0.111)	
- Range	0.777 - 0.932	0.812 - 1.210	-0.104 - 0.357	
Medial lemniscus L				0.265
- Mean (SD)	0.815 (0.062)	0.881 (0.066)	0.066 (0.071)	
- Range	0.706 - 0.942	0.796 - 1.034	-0.056 - 0.201	
Inferior cerebellar peduncle R				0.977
- Mean (SD)	1.163 (0.223)	1.160 (0.198)	-0.003 (0.325)	
- Range	0.965 - 1.790	0.927 - 1.639	-0.614 - 0.623	
Inferior cerebellar peduncle L				0.882
- Mean (SD)	1.099 (0.258)	1.132 (0.257)	0.033 (0.402)	
- Range	0.852 - 1.784	0.917 - 1.748	-0.760 - 0.784	
Superior cerebellar peduncle R				0.937
- Mean (SD)	1.230 (0.184)	1.240 (0.167)	0.010 (0.235)	
- Range	0.971 - 1.547	0.947 - 1.532	-0.557 - 0.402	
Superior cerebellar peduncle L				0.778
- Mean (SD)	1.330 (0.262)	1.295 (0.197)	-0.035 (0.260)	
- Range	0.955 - 1.938	0.913 - 1.623	-0.575 - 0.327	
Cerebral peduncle R				0.716
- Mean (SD)	0.882 (0.047)	0.888 (0.049)	0.006 (0.035)	
- Range	0.808 - 0.960	0.806 - 0.971	-0.017 - 0.114	
Cerebral peduncle L				0.592
- Mean (SD)	0.845 (0.035)	0.836 (0.033)	-0.009 (0.028)	
- Range	0.784 - 0.890	0.791 - 0.883	-0.065 - 0.045	
Anterior limb of internal capsule R				0.882
- Mean (SD)	0.848 (0.050)	0.846 (0.058)	-0.002 (0.026)	
- Range	0.793 - 0.934	0.792 - 0.962	-0.057 - 0.037	
Anterior limb of internal capsule L				0.668
- Mean (SD)	0.870 (0.064)	0.877 (0.070)	0.007 (0.036)	
- Range	0.781 - 0.985	0.789 - 1.025	-0.041 - 0.080	
Posterior limb of internal capsule R				0.967
- Mean (SD)	0.760 (0.019)	0.760 (0.021)	0.000 (0.015)	
- Range	0.731 - 0.797	0.722 - 0.788	-0.030 - 0.024	
Posterior limb of internal capsule L				0.553
- Mean (SD)	0.756 (0.018)	0.767 (0.027)	0.010 (0.026)	

	Post (N=13)	Pre (N=13)	Difference (N=13)	p value
- Range	0.726 - 0.788	0.726 - 0.819	-0.008 - 0.087	
Retrolenticular part of internal capsule R				0.553
- Mean (SD)	0.909 (0.056)	0.924 (0.077)	0.015 (0.041)	
- Range	0.843 - 1.031	0.845 - 1.062	-0.021 - 0.132	
Retrolenticular part of internal capsule L				0.553
- Mean (SD)	0.906 (0.051)	0.933 (0.078)	0.027 (0.050)	
- Range	0.833 - 0.998	0.825 - 1.074	-0.057 - 0.118	
Anterior corona radiata R				0.621
- Mean (SD)	0.913 (0.065)	0.929 (0.122)	0.016 (0.070)	
- Range	0.843 - 1.054	0.823 - 1.248	-0.029 - 0.236	
Anterior corona radiata L				0.553
- Mean (SD)	0.911 (0.095)	0.922 (0.106)	0.010 (0.027)	
- Range	0.821 - 1.179	0.829 - 1.215	-0.023 - 0.078	
Superior corona radiata R				0.553
- Mean (SD)	0.794 (0.057)	0.811 (0.077)	0.017 (0.036)	
- Range	0.728 - 0.893	0.735 - 0.946	-0.011 - 0.121	
Superior corona radiata L				0.553
- Mean (SD)	0.810 (0.059)	0.832 (0.103)	0.022 (0.054)	
- Range	0.720 - 0.930	0.733 - 1.099	-0.015 - 0.169	
Posterior corona radiata R				0.592
- Mean (SD)	0.916 (0.103)	0.934 (0.132)	0.019 (0.064)	
- Range	0.801 - 1.123	0.780 - 1.146	-0.048 - 0.218	
Posterior corona radiata L				0.553
- Mean (SD)	0.930 (0.100)	0.961 (0.153)	0.031 (0.075)	
- Range	0.810 - 1.126	0.800 - 1.328	-0.051 - 0.202	
Posterior thalamic radiation R				0.882
- Mean (SD)	1.010 (0.193)	1.013 (0.180)	0.003 (0.040)	
- Range	0.834 - 1.518	0.828 - 1.510	-0.067 - 0.094	
Posterior thalamic radiation L				0.553
- Mean (SD)	1.075 (0.130)	1.121 (0.188)	0.046 (0.113)	
- Range	0.867 - 1.274	0.935 - 1.589	-0.093 - 0.373	
Sagittal stratum R				0.609
- Mean (SD)	0.990 (0.097)	1.003 (0.126)	0.013 (0.053)	
- Range	0.875 - 1.198	0.889 - 1.335	-0.070 - 0.137	
Sagittal stratum L				0.716
- Mean (SD)	1.051 (0.108)	1.062 (0.104)	0.011 (0.062)	
- Range	0.898 - 1.209	0.913 - 1.279	-0.093 - 0.163	
External capsule R				0.592
- Mean (SD)	0.847 (0.047)	0.856 (0.057)	0.009 (0.031)	
- Range	0.772 - 0.947	0.791 - 0.955	-0.025 - 0.075	
External capsule L				0.451
- Mean (SD)	0.848 (0.048)	0.831 (0.052)	-0.017 (0.024)	
- Range	0.792 - 0.939	0.771 - 0.945	-0.071 - 0.013	
Cingulum cingulate gyrus R				0.592
- Mean (SD)	0.933 (0.068)	0.918 (0.053)	-0.015 (0.051)	
- Range	0.810 - 1.067	0.819 - 0.979	-0.109 - 0.073	
Cingulum cingulate gyrus L				0.553
- Mean (SD)	0.941 (0.067)	0.962 (0.080)	0.021 (0.057)	
- Range	0.824 - 1.035	0.839 - 1.108	-0.036 - 0.146	
Cingulum hippocampus R				0.977
- Mean (SD)	1.107 (0.164)	1.109 (0.138)	0.001 (0.170)	
- Range	0.855 - 1.506	0.934 - 1.368	-0.480 - 0.204	
Cingulum hippocampus L				0.451
- Mean (SD)	1.121 (0.136)	1.187 (0.143)	0.066 (0.091)	
- Range	0.870 - 1.336	0.930 - 1.407	-0.049 - 0.213	
Fornix R				0.553
- Mean (SD)	1.044 (0.116)	1.082 (0.146)	0.038 (0.077)	
- Range	0.859 - 1.298	0.884 - 1.370	-0.029 - 0.273	
Fornix L				0.617
- Mean (SD)	0.986 (0.118)	1.003 (0.129)	0.017 (0.068)	
- Range	0.817 - 1.209	0.868 - 1.313	-0.126 - 0.121	
Superior longitudinal fasciculus R				0.882

	Post (N=13)	Pre (N=13)	Difference (N=13)	p value
- Mean (SD)	0.800 (0.040)	0.801 (0.045)	0.002 (0.021)	
- Range	0.751 - 0.881	0.734 - 0.883	-0.046 - 0.043	
Superior longitudinal fasciculus L				0.668
- Mean (SD)	0.779 (0.035)	0.784 (0.040)	0.005 (0.023)	
- Range	0.741 - 0.847	0.738 - 0.865	-0.028 - 0.050	
Superior fronto occipital fasciculus R				0.553
- Mean (SD)	0.884 (0.157)	0.932 (0.252)	0.048 (0.107)	
- Range	0.745 - 1.300	0.733 - 1.571	-0.049 - 0.271	
Superior fronto occipital fasciculus L				0.553
- Mean (SD)	0.995 (0.217)	1.039 (0.306)	0.043 (0.123)	
- Range	0.770 - 1.580	0.795 - 1.863	-0.106 - 0.327	
Uncinate fasciculus R				0.574
- Mean (SD)	0.975 (0.242)	0.919 (0.136)	-0.055 (0.166)	
- Range	0.803 - 1.519	0.790 - 1.267	-0.475 - 0.132	
Uncinate fasciculus L				0.553
- Mean (SD)	0.835 (0.041)	0.876 (0.089)	0.041 (0.084)	
- Range	0.778 - 0.926	0.784 - 1.136	-0.022 - 0.301	
Tapetum R				0.553
- Mean (SD)	1.858 (0.425)	1.941 (0.457)	0.083 (0.205)	
- Range	1.190 - 2.620	1.141 - 2.588	-0.201 - 0.437	
Tapetum L				0.724
- Mean (SD)	2.188 (0.516)	2.223 (0.477)	0.036 (0.213)	
- Range	1.418 - 3.016	1.407 - 2.919	-0.255 - 0.370	

Radial diffusivity (RD, (10^-3)*(mm^2)/s) (DTI) - Offspring of obese mothers (OOM) subjects pre vs post (paired tests) - (JHU white-matter tractography atlas) - Uncorrected p-values

	Post (N=13)	Pre (N=13)	Difference (N=13)	p value
Middle cerebellar peduncle				0.586
- Mean (SD)	0.860 (0.147)	0.892 (0.140)	0.032 (0.208)	
- Range	0.663 - 1.171	0.763 - 1.228	-0.289 - 0.447	
Pontine crossing tract				0.349
- Mean (SD)	0.621 (0.063)	0.655 (0.085)	0.034 (0.126)	
- Range	0.522 - 0.792	0.542 - 0.891	-0.195 - 0.327	
Genu of corpus callosum				0.252
- Mean (SD)	1.036 (0.080)	1.050 (0.110)	0.014 (0.043)	
- Range	0.975 - 1.265	0.916 - 1.324	-0.072 - 0.090	
Body of corpus callosum				0.097
- Mean (SD)	0.800 (0.063)	0.823 (0.092)	0.023 (0.046)	
- Range	0.717 - 0.929	0.718 - 1.027	-0.024 - 0.126	
Splenium of corpus callosum				0.032
- Mean (SD)	0.632 (0.052)	0.658 (0.084)	0.026 (0.039)	
- Range	0.550 - 0.750	0.532 - 0.857	-0.018 - 0.107	
Fornix				0.698
- Mean (SD)	2.044 (0.374)	2.037 (0.397)	-0.007 (0.064)	
- Range	1.593 - 2.597	1.568 - 2.596	-0.112 - 0.134	
Corticospinal tract R				0.252
- Mean (SD)	0.608 (0.050)	0.629 (0.055)	0.021 (0.062)	
- Range	0.521 - 0.685	0.538 - 0.747	-0.065 - 0.172	
Corticospinal tract L				0.162
- Mean (SD)	0.588 (0.047)	0.604 (0.043)	0.015 (0.037)	
- Range	0.513 - 0.675	0.535 - 0.668	-0.066 - 0.075	
Medial lemniscus R				0.097
- Mean (SD)	0.627 (0.043)	0.678 (0.092)	0.051 (0.103)	
- Range	0.570 - 0.714	0.593 - 0.932	-0.106 - 0.332	
Medial lemniscus L				0.042
- Mean (SD)	0.602 (0.052)	0.648 (0.070)	0.046 (0.073)	
- Range	0.509 - 0.703	0.550 - 0.808	-0.070 - 0.163	
Inferior cerebellar peduncle R				0.834
- Mean (SD)	0.967 (0.203)	0.949 (0.181)	-0.018 (0.304)	
- Range	0.796 - 1.529	0.722 - 1.375	-0.593 - 0.525	
Inferior cerebellar peduncle L				0.682
- Mean (SD)	0.903 (0.228)	0.945 (0.235)	0.042 (0.358)	
- Range	0.668 - 1.471	0.781 - 1.498	-0.627 - 0.761	
Superior cerebellar peduncle R				0.789
- Mean (SD)	0.922 (0.201)	0.942 (0.171)	0.020 (0.267)	
- Range	0.695 - 1.286	0.724 - 1.264	-0.562 - 0.459	
Superior cerebellar peduncle L				0.801
- Mean (SD)	1.033 (0.267)	1.012 (0.186)	-0.021 (0.292)	
- Range	0.694 - 1.702	0.713 - 1.378	-0.677 - 0.393	
Cerebral peduncle R				0.495
- Mean (SD)	0.584 (0.044)	0.589 (0.039)	0.005 (0.026)	
- Range	0.522 - 0.662	0.532 - 0.653	-0.021 - 0.075	
Cerebral peduncle L				0.559
- Mean (SD)	0.544 (0.031)	0.540 (0.033)	-0.004 (0.026)	
- Range	0.496 - 0.591	0.488 - 0.595	-0.062 - 0.037	
Anterior limb of internal capsule R				0.789
- Mean (SD)	0.630 (0.052)	0.632 (0.062)	0.002 (0.029)	
- Range	0.561 - 0.734	0.574 - 0.761	-0.051 - 0.049	
Anterior limb of internal capsule L				0.294
- Mean (SD)	0.638 (0.063)	0.650 (0.073)	0.012 (0.039)	
- Range	0.553 - 0.758	0.558 - 0.800	-0.026 - 0.105	
Posterior limb of internal capsule R				0.511
- Mean (SD)	0.510 (0.020)	0.512 (0.023)	0.003 (0.014)	
- Range	0.480 - 0.550	0.476 - 0.546	-0.031 - 0.025	
Posterior limb of internal capsule L				0.249
- Mean (SD)	0.503 (0.022)	0.512 (0.026)	0.009 (0.027)	

	Post (N=13)	Pre (N=13)	Difference (N=13)	p value
- Range	0.462 - 0.536	0.481 - 0.553	-0.021 - 0.081	
Retrolenticular part of internal capsule R				0.126
- Mean (SD)	0.677 (0.044)	0.695 (0.063)	0.018 (0.039)	
- Range	0.608 - 0.753	0.607 - 0.797	-0.018 - 0.127	
Retrolenticular part of internal capsule L				0.199
- Mean (SD)	0.674 (0.046)	0.691 (0.074)	0.018 (0.047)	
- Range	0.591 - 0.752	0.572 - 0.818	-0.061 - 0.094	
Anterior corona radiata R				0.388
- Mean (SD)	0.745 (0.062)	0.760 (0.111)	0.015 (0.060)	
- Range	0.670 - 0.870	0.653 - 1.054	-0.031 - 0.204	
Anterior corona radiata L				0.177
- Mean (SD)	0.740 (0.082)	0.751 (0.087)	0.011 (0.028)	
- Range	0.651 - 0.953	0.667 - 0.963	-0.019 - 0.076	
Superior corona radiata R				0.128
- Mean (SD)	0.616 (0.044)	0.630 (0.061)	0.014 (0.032)	
- Range	0.576 - 0.697	0.574 - 0.755	-0.015 - 0.102	
Superior corona radiata L				0.171
- Mean (SD)	0.627 (0.044)	0.643 (0.074)	0.017 (0.041)	
- Range	0.562 - 0.696	0.580 - 0.821	-0.018 - 0.125	
Posterior corona radiata R				0.211
- Mean (SD)	0.696 (0.080)	0.716 (0.107)	0.020 (0.053)	
- Range	0.613 - 0.837	0.598 - 0.889	-0.035 - 0.181	
Posterior corona radiata L				0.298
- Mean (SD)	0.725 (0.080)	0.741 (0.116)	0.017 (0.055)	
- Range	0.626 - 0.869	0.608 - 1.002	-0.052 - 0.133	
Posterior thalamic radiation R				0.472
- Mean (SD)	0.742 (0.150)	0.747 (0.149)	0.006 (0.028)	
- Range	0.601 - 1.130	0.597 - 1.152	-0.035 - 0.062	
Posterior thalamic radiation L				0.174
- Mean (SD)	0.803 (0.107)	0.840 (0.157)	0.037 (0.093)	
- Range	0.647 - 0.976	0.695 - 1.220	-0.073 - 0.306	
Sagittal stratum R				0.538
- Mean (SD)	0.757 (0.076)	0.766 (0.104)	0.009 (0.049)	
- Range	0.681 - 0.913	0.663 - 1.053	-0.061 - 0.140	
Sagittal stratum L				0.576
- Mean (SD)	0.825 (0.085)	0.834 (0.090)	0.009 (0.058)	
- Range	0.709 - 0.963	0.685 - 1.037	-0.084 - 0.158	
External capsule R				0.299
- Mean (SD)	0.695 (0.042)	0.704 (0.051)	0.009 (0.031)	
- Range	0.624 - 0.774	0.642 - 0.793	-0.018 - 0.083	
External capsule L				0.038
- Mean (SD)	0.692 (0.046)	0.676 (0.050)	-0.015 (0.024)	
- Range	0.638 - 0.768	0.613 - 0.778	-0.070 - 0.010	
Cingulum cingulate gyrus R				0.532
- Mean (SD)	0.754 (0.068)	0.746 (0.054)	-0.008 (0.047)	
- Range	0.637 - 0.889	0.653 - 0.812	-0.098 - 0.081	
Cingulum cingulate gyrus L				0.171
- Mean (SD)	0.750 (0.065)	0.770 (0.076)	0.020 (0.049)	
- Range	0.637 - 0.845	0.668 - 0.912	-0.023 - 0.130	
Cingulum hippocampus R				0.988
- Mean (SD)	0.950 (0.152)	0.949 (0.130)	-0.001 (0.162)	
- Range	0.714 - 1.327	0.772 - 1.178	-0.453 - 0.211	
Cingulum hippocampus L				0.022
- Mean (SD)	0.964 (0.128)	1.027 (0.131)	0.063 (0.086)	
- Range	0.738 - 1.161	0.778 - 1.214	-0.069 - 0.199	
Fornix R				0.212
- Mean (SD)	0.849 (0.110)	0.876 (0.136)	0.027 (0.073)	
- Range	0.687 - 1.104	0.715 - 1.144	-0.049 - 0.239	
Fornix L				0.378
- Mean (SD)	0.786 (0.115)	0.802 (0.123)	0.015 (0.060)	
- Range	0.643 - 1.027	0.675 - 1.103	-0.111 - 0.111	
Superior longitudinal fasciculus R				0.591

	Post (N=13)	Pre (N=13)	Difference (N=13)	p value
- Mean (SD)	0.632 (0.036)	0.635 (0.042)	0.003 (0.023)	
- Range	0.584 - 0.709	0.581 - 0.709	-0.051 - 0.040	
Superior longitudinal fasciculus L				0.641
- Mean (SD)	0.612 (0.035)	0.615 (0.039)	0.003 (0.024)	
- Range	0.566 - 0.680	0.553 - 0.697	-0.032 - 0.049	
Superior fronto occipital fasciculus R				0.112
- Mean (SD)	0.714 (0.153)	0.750 (0.218)	0.036 (0.076)	
- Range	0.590 - 1.119	0.570 - 1.308	-0.050 - 0.189	
Superior fronto occipital fasciculus L				0.129
- Mean (SD)	0.808 (0.199)	0.853 (0.267)	0.046 (0.101)	
- Range	0.599 - 1.310	0.622 - 1.541	-0.097 - 0.279	
Uncinate fasciculus R				0.296
- Mean (SD)	0.779 (0.234)	0.729 (0.131)	-0.051 (0.167)	
- Range	0.607 - 1.324	0.605 - 1.078	-0.499 - 0.129	
Uncinate fasciculus L				0.247
- Mean (SD)	0.659 (0.040)	0.687 (0.096)	0.028 (0.082)	
- Range	0.595 - 0.720	0.577 - 0.950	-0.047 - 0.267	
Tapetum R				0.221
- Mean (SD)	1.551 (0.372)	1.608 (0.406)	0.057 (0.160)	
- Range	0.966 - 2.175	0.926 - 2.204	-0.133 - 0.347	
Tapetum L				0.675
- Mean (SD)	1.876 (0.457)	1.898 (0.420)	0.022 (0.182)	
- Range	1.224 - 2.603	1.204 - 2.476	-0.258 - 0.323	

Radial diffusivity (RD, (10^-3)*(mm^2)/s) (DTI) - Offspring of obese mothers (OOM) subjects pre vs post (paired tests) - (JHU white-matter tractography atlas) - FDR corrected p-values

	Post (N=13)	Pre (N=13)	Difference (N=13)	p value
Middle cerebellar peduncle				0.728
- Mean (SD)	0.860 (0.147)	0.892 (0.140)	0.032 (0.208)	
- Range	0.663 - 1.171	0.763 - 1.228	-0.289 - 0.447	
Pontine crossing tract				0.598
- Mean (SD)	0.621 (0.063)	0.655 (0.085)	0.034 (0.126)	
- Range	0.522 - 0.792	0.542 - 0.891	-0.195 - 0.327	
Genu of corpus callosum				0.526
- Mean (SD)	1.036 (0.080)	1.050 (0.110)	0.014 (0.043)	
- Range	0.975 - 1.265	0.916 - 1.324	-0.072 - 0.090	
Body of corpus callosum				0.526
- Mean (SD)	0.800 (0.063)	0.823 (0.092)	0.023 (0.046)	
- Range	0.717 - 0.929	0.718 - 1.027	-0.024 - 0.126	
Splenium of corpus callosum				0.505
- Mean (SD)	0.632 (0.052)	0.658 (0.084)	0.026 (0.039)	
- Range	0.550 - 0.750	0.532 - 0.857	-0.018 - 0.107	
Fornix				0.780
- Mean (SD)	2.044 (0.374)	2.037 (0.397)	-0.007 (0.064)	
- Range	1.593 - 2.597	1.568 - 2.596	-0.112 - 0.134	
Corticospinal tract R				0.526
- Mean (SD)	0.608 (0.050)	0.629 (0.055)	0.021 (0.062)	
- Range	0.521 - 0.685	0.538 - 0.747	-0.065 - 0.172	
Corticospinal tract L				0.526
- Mean (SD)	0.588 (0.047)	0.604 (0.043)	0.015 (0.037)	
- Range	0.513 - 0.675	0.535 - 0.668	-0.066 - 0.075	
Medial lemniscus R				0.526
- Mean (SD)	0.627 (0.043)	0.678 (0.092)	0.051 (0.103)	
- Range	0.570 - 0.714	0.593 - 0.932	-0.106 - 0.332	
Medial lemniscus L				0.505
- Mean (SD)	0.602 (0.052)	0.648 (0.070)	0.046 (0.073)	
- Range	0.509 - 0.703	0.550 - 0.808	-0.070 - 0.163	
Inferior cerebellar peduncle R				0.852
- Mean (SD)	0.967 (0.203)	0.949 (0.181)	-0.018 (0.304)	
- Range	0.796 - 1.529	0.722 - 1.375	-0.593 - 0.525	
Inferior cerebellar peduncle L				0.779
- Mean (SD)	0.903 (0.228)	0.945 (0.235)	0.042 (0.358)	
- Range	0.668 - 1.471	0.781 - 1.498	-0.627 - 0.761	
Superior cerebellar peduncle R				0.836
- Mean (SD)	0.922 (0.201)	0.942 (0.171)	0.020 (0.267)	
- Range	0.695 - 1.286	0.724 - 1.264	-0.562 - 0.459	
Superior cerebellar peduncle L				0.836
- Mean (SD)	1.033 (0.267)	1.012 (0.186)	-0.021 (0.292)	
- Range	0.694 - 1.702	0.713 - 1.378	-0.677 - 0.393	
Cerebral peduncle R				0.728
- Mean (SD)	0.584 (0.044)	0.589 (0.039)	0.005 (0.026)	
- Range	0.522 - 0.662	0.532 - 0.653	-0.021 - 0.075	
Cerebral peduncle L				0.728
- Mean (SD)	0.544 (0.031)	0.540 (0.033)	-0.004 (0.026)	
- Range	0.496 - 0.591	0.488 - 0.595	-0.062 - 0.037	
Anterior limb of internal capsule R				0.836
- Mean (SD)	0.630 (0.052)	0.632 (0.062)	0.002 (0.029)	
- Range	0.561 - 0.734	0.574 - 0.761	-0.051 - 0.049	
Anterior limb of internal capsule L				0.532
- Mean (SD)	0.638 (0.063)	0.650 (0.073)	0.012 (0.039)	
- Range	0.553 - 0.758	0.558 - 0.800	-0.026 - 0.105	
Posterior limb of internal capsule R				0.728
- Mean (SD)	0.510 (0.020)	0.512 (0.023)	0.003 (0.014)	
- Range	0.480 - 0.550	0.476 - 0.546	-0.031 - 0.025	
Posterior limb of internal capsule L				0.526
- Mean (SD)	0.503 (0.022)	0.512 (0.026)	0.009 (0.027)	

	Post (N=13)	Pre (N=13)	Difference (N=13)	p value
- Range	0.462 - 0.536	0.481 - 0.553	-0.021 - 0.081	
Retrolenticular part of internal capsule R				0.526
- Mean (SD)	0.677 (0.044)	0.695 (0.063)	0.018 (0.039)	
- Range	0.608 - 0.753	0.607 - 0.797	-0.018 - 0.127	
Retrolenticular part of internal capsule L				0.526
- Mean (SD)	0.674 (0.046)	0.691 (0.074)	0.018 (0.047)	
- Range	0.591 - 0.752	0.572 - 0.818	-0.061 - 0.094	
Anterior corona radiata R				0.621
- Mean (SD)	0.745 (0.062)	0.760 (0.111)	0.015 (0.060)	
- Range	0.670 - 0.870	0.653 - 1.054	-0.031 - 0.204	
Anterior corona radiata L				0.526
- Mean (SD)	0.740 (0.082)	0.751 (0.087)	0.011 (0.028)	
- Range	0.651 - 0.953	0.667 - 0.963	-0.019 - 0.076	
Superior corona radiata R				0.526
- Mean (SD)	0.616 (0.044)	0.630 (0.061)	0.014 (0.032)	
- Range	0.576 - 0.697	0.574 - 0.755	-0.015 - 0.102	
Superior corona radiata L				0.526
- Mean (SD)	0.627 (0.044)	0.643 (0.074)	0.017 (0.041)	
- Range	0.562 - 0.696	0.580 - 0.821	-0.018 - 0.125	
Posterior corona radiata R				0.526
- Mean (SD)	0.696 (0.080)	0.716 (0.107)	0.020 (0.053)	
- Range	0.613 - 0.837	0.598 - 0.889	-0.035 - 0.181	
Posterior corona radiata L				0.532
- Mean (SD)	0.725 (0.080)	0.741 (0.116)	0.017 (0.055)	
- Range	0.626 - 0.869	0.608 - 1.002	-0.052 - 0.133	
Posterior thalamic radiation R				0.728
- Mean (SD)	0.742 (0.150)	0.747 (0.149)	0.006 (0.028)	
- Range	0.601 - 1.130	0.597 - 1.152	-0.035 - 0.062	
Posterior thalamic radiation L				0.526
- Mean (SD)	0.803 (0.107)	0.840 (0.157)	0.037 (0.093)	
- Range	0.647 - 0.976	0.695 - 1.220	-0.073 - 0.306	
Sagittal stratum R				0.728
- Mean (SD)	0.757 (0.076)	0.766 (0.104)	0.009 (0.049)	
- Range	0.681 - 0.913	0.663 - 1.053	-0.061 - 0.140	
Sagittal stratum L				0.728
- Mean (SD)	0.825 (0.085)	0.834 (0.090)	0.009 (0.058)	
- Range	0.709 - 0.963	0.685 - 1.037	-0.084 - 0.158	
External capsule R				0.532
- Mean (SD)	0.695 (0.042)	0.704 (0.051)	0.009 (0.031)	
- Range	0.624 - 0.774	0.642 - 0.793	-0.018 - 0.083	
External capsule L				0.505
- Mean (SD)	0.692 (0.046)	0.676 (0.050)	-0.015 (0.024)	
- Range	0.638 - 0.768	0.613 - 0.778	-0.070 - 0.010	
Cingulum cingulate gyrus R				0.728
- Mean (SD)	0.754 (0.068)	0.746 (0.054)	-0.008 (0.047)	
- Range	0.637 - 0.889	0.653 - 0.812	-0.098 - 0.081	
Cingulum cingulate gyrus L				0.526
- Mean (SD)	0.750 (0.065)	0.770 (0.076)	0.020 (0.049)	
- Range	0.637 - 0.845	0.668 - 0.912	-0.023 - 0.130	
Cingulum hippocampus R				0.988
- Mean (SD)	0.950 (0.152)	0.949 (0.130)	-0.001 (0.162)	
- Range	0.714 - 1.327	0.772 - 1.178	-0.453 - 0.211	
Cingulum hippocampus L				0.505
- Mean (SD)	0.964 (0.128)	1.027 (0.131)	0.063 (0.086)	
- Range	0.738 - 1.161	0.778 - 1.214	-0.069 - 0.199	
Fornix R				0.526
- Mean (SD)	0.849 (0.110)	0.876 (0.136)	0.027 (0.073)	
- Range	0.687 - 1.104	0.715 - 1.144	-0.049 - 0.239	
Fornix L				0.621
- Mean (SD)	0.786 (0.115)	0.802 (0.123)	0.015 (0.060)	
- Range	0.643 - 1.027	0.675 - 1.103	-0.111 - 0.111	
Superior longitudinal fasciculus R				0.728

	Post (N=13)	Pre (N=13)	Difference (N=13)	p value
- Mean (SD)	0.632 (0.036)	0.635 (0.042)	0.003 (0.023)	
- Range	0.584 - 0.709	0.581 - 0.709	-0.051 - 0.040	
Superior longitudinal fasciculus L				0.769
- Mean (SD)	0.612 (0.035)	0.615 (0.039)	0.003 (0.024)	
- Range	0.566 - 0.680	0.553 - 0.697	-0.032 - 0.049	
Superior fronto occipital fasciculus R				0.526
- Mean (SD)	0.714 (0.153)	0.750 (0.218)	0.036 (0.076)	
- Range	0.590 - 1.119	0.570 - 1.308	-0.050 - 0.189	
Superior fronto occipital fasciculus L				0.526
- Mean (SD)	0.808 (0.199)	0.853 (0.267)	0.046 (0.101)	
- Range	0.599 - 1.310	0.622 - 1.541	-0.097 - 0.279	
Uncinate fasciculus R				0.532
- Mean (SD)	0.779 (0.234)	0.729 (0.131)	-0.051 (0.167)	
- Range	0.607 - 1.324	0.605 - 1.078	-0.499 - 0.129	
Uncinate fasciculus L				0.526
- Mean (SD)	0.659 (0.040)	0.687 (0.096)	0.028 (0.082)	
- Range	0.595 - 0.720	0.577 - 0.950	-0.047 - 0.267	
Tapetum R				0.526
- Mean (SD)	1.551 (0.372)	1.608 (0.406)	0.057 (0.160)	
- Range	0.966 - 2.175	0.926 - 2.204	-0.133 - 0.347	
Tapetum L				0.779
- Mean (SD)	1.876 (0.457)	1.898 (0.420)	0.022 (0.182)	
- Range	1.224 - 2.603	1.204 - 2.476	-0.258 - 0.323	
